# Differential Gene Expression at the Maternal-Fetal Interface in Preeclampsia Is Influenced by Gestational Age

**DOI:** 10.1371/journal.pone.0069848

**Published:** 2013-07-31

**Authors:** Ingrid A. Lian, Mette Langaas, Eric Moses, Åsa Johansson

**Affiliations:** 1 Department of Cancer Research and Molecular Medicine, Norwegian University of Science and Technology (NTNU), Trondheim, Norway; 2 Department of Mathematical Sciences, NTNU, Trondheim, Norway; 3 Centre for Genetic Epidemiology and Biostatistics, University of Western Australia, Perth, Australia; 4 Department of Immunology, Genetics and Pathology, Uppsala University, Uppsala, Sweden; 5 Uppsala Clinical Research Center, Uppsala University, Uppsala, Sweden; National Institute of Environmental and Health Sciences, United States of America

## Abstract

Genome-wide transcription data of utero-placental tissue has been used to identify altered gene expression associated with preeclampsia (PE). As many women with PE deliver preterm, there is often a difference in gestational age between PE women and healthy pregnant controls. This may pose a potential bias since gestational age has been shown to dramatically influence gene expression in utero-placental tissue. By pooling data from three genome-wide transcription studies of the maternal-fetal interface, we have evaluated the relative effect of gestational age and PE on gene expression. A total of 18,180 transcripts were evaluated in 49 PE cases and 105 controls, with gestational age ranging from week 14 to 42. A total of 22 transcripts were associated with PE, whereas 92 transcripts with gestational age (nominal *P* value <1.51*10^−6^, Bonferroni adjusted *P* value <0.05). Our results indicate that gestational age has a great influence on gene expression both in normal and PE-complicated pregnancies. This effect might introduce serious bias in data analyses and needs to be carefully assessed in future genome-wide transcription studies.

## Introduction

Preeclampsia (PE) is one of the leading causes of perinatal mortality and deaths of pregnant women worldwide [1], [2]. PE is a pregnancy-specific disorder, diagnosed by *de novo* onset of hypertension and proteinuria in the latter half of pregnancy [2]–[4]. To date, there are few reliable predictive tests or any effective treatment available, except delivery of the baby and the placenta. Consequently, PE accounts for approximately 20% of all preterm births [5].

The aetiology of PE is not completely understood, but it is generally considered that disturbed interactions between the invading (fetal) trophoblasts and maternal cells causing defective trophoblast invasion are important pathophysiological events. The subsequent impaired spiral artery remodelling and reduced placental perfusion is proposed to create oxidative stress and a release of inflammatory factors into the maternal circulation, causing overt PE [6]. Gene expression analyses may provide further insight in mechanisms of disease and function as preventive, predictive or therapeutic measures. As the molecular mechanisms behind impaired trophoblast invasion are preferentially reflected at the maternal-fetal interface, attempts to identify aberrant gene expression associated with preeclamptic pregnancies at this site have been made. So far, a number of genome-wide transcription analyses of decidual and placental bed tissue have been performed using a limited number of samples [7]–[9]. Findings in these studies have been inconsistent, probably reflecting lack of power in each individual study in combination with the complexity of the disease.

Women with PE often deliver preterm, due to medical indications or the condition itself. Transcriptional comparisons of gene expression in utero-placental tissue from women with PE and women with normal pregnancies are therefore often hampered by relatively large differences in gestational age. It has been shown that gene expression in utero-placental tissue differs dramatically over gestation [10], [11]. However, most studies aiming to identify altered utero-placental expression in PE have failed to properly assess changes in transcription levels caused by such differences in gestational age.

In this study, we have pooled data from three different genome-wide transcription studies [8], [9], [11] of tissue from the maternal-fetal interface to assess differential gene expression associated with both PE and gestational age. This study is the first to include both variables (PE and gestational age), but also the first to combine data from different genome-wide platforms to analyse transcription profiles. In addition, with 154 samples analysed, this is so far the largest study performed to identify differences in expression patterns at the maternal-fetal interface.

## Materials and Methods

### Microarray Datasets

In this study, we have utilised three publicly available gene expression datasets from tissue from the maternal-fetal interface (Table 1). The first dataset consist of analyses of basal plate biopsies from 36 second- and third trimester singleton pregnancies, including elective surgical terminations and normal, uncomplicated term pregnancies [11]. None of these 36 samples were from women with pregnancies complicated by PE, fetal anomalies, hypertension, diabetes, infections or other significant maternal health issues. The dataset is available at the Gene Expression Omnibus (www.ncbi.nlm.nih.gov/geo/), accession no. GSE5999. The second dataset consists of 23 basal plate biopsies from women who developed severe PE (n = 12) or had preterm labour (n = 11) [9]. Approximately one third of the cases with preterm labour resulted from cervical insufficiency, and 5 of the PE pregnancies were complicated by fetal growth restriction. Pregnancies complicated by fetal anomalies, premature rupture of the membranes, infections, diabetes, autoimmune diseases or pregnancies with multiple gestations were excluded. The dataset is available at Gene Expression Omnibus (www.ncbi.nlm.nih.gov/geo/), accession no. GSE14722. The third dataset consists of 95 decidua basalis samples from women who developed PE (n = 37) and women with normal, uncomplicated term pregnancies (n = 58) [8]. Among the 37 PE cases, 30 had severe PE or PE complicated by small for gestational age (SGA) and seven cases had mild PE not complicated by SGA. Pregnancies with multiple gestations, or fetal and placental anomalies (such as placenta accreta, placenta membranacea, placentas from fetuses with chromosomal anomalies or developmental anomalies, or macroscopic and microscopic signs of infection) were excluded. The dataset is available via ArrayExpress (www.ebi.ac.uk/microarray-as/ae/), accession no. E-TABM-682. The criteria to diagnose PE as well as severe PE in dataset #2 and #3 were similar [12], [13]. All data used for this study have been retrieved from www.ncbi.nlm.nih.gov/geo/or www.ebi.ac.uk/microarray-as/ae/, and no additional ethical approvals were obtained. A more comprehensive description of the tissue sampling and study population characteristics can be found in the original papers for the respective studies [8], [9], [11].

**Table 1:** Descriptives of the three dataset used in this study.

Dataset	Number ofsamplesPreeclampsia(PE)	Number ofsamplesNon-PE (NP)	Mean gestationalage PE, weeks(min, max)	Mean gestationalage NP, weeks(min, max)	Microarray platform	Number ofprobes/probesets
#1 [11]		2^nd^ trimester, n = 273^rd^ trimester, n = 9		19.3 (14.0, 24.0)38.6 (37.0, 40.0)	Affymetrix, HG-U133A&BGeneChip	44,928
#2 [9]	3^rd^ trimester,n = 12	3^rd^ trimester, n = 11	32.1 (24.1, 37.6)	31.0 (24.7, 36.6)	Affymetrix, HG-U133A&BGeneChip	44,928
#3 [8]	3^rd^ trimester,n = 37	3^rd^ trimester, n = 58	31.9 (25.0, 39.0)	38.7 (37.0, 42.0)	Illumina, HumanWG-6 v2Expression Beadchip	48,095

### Probe-mapping and Construction of Probe Pairs between Microarray Platforms

The transcription data that the three studies used in this work originate from has been produced on two different microarray platforms. Dataset #1 and #2 were analysed on Affymetrix HG-U133A&B GeneChips (Affymetrix, Santa Clara, CA, USA) including 44,928 probesets, and dataset #3 was analysed on Illumina HumanWG-6 v2 Expression BeadChips (Illumina Inc., San Diego, CA, USA) including 48,095 probes. Affymetrix interrogates mRNA expression using a panel of different 25mer probes per transcript (probesets), whereas Illumina uses multiple, identical 50mer probes per transcript. Both platforms provide probe annotations, but since the sequence of the human genome is constantly updated, these annotations do not always match the latest updated sequence [14]. Having accurate knowledge of which transcripts the probes and probesets are measuring is essential to ensure accurate biological interpretation of the results in downstream analyses. Therefore, probes and probesets from the two platforms were remapped to Ensembl transcript predictions using the available Ensembl annotation for expression microarrays (Ensembl release 57). This Ensembl annotation was produced by first aligning the probe sequences to the corresponding genome sequence (Ensembl release 57), using the exonerate alignment tool [15]. A default of one base pair mismatch was permitted between the probe and the genome sequence assembly. Probes that matched at 100 or more locations (e.g. suspected Alu repeats) were discarded. The remaining probes or probesets were associated with Ensembl transcript predictions. For Affymetrix probesets, it was required that >50% of the probes matched a given transcript sequence. This mapping procedure is described in more detail at the Ensembl webpage (www.ensembl.org/info/docs/microarray_probe_set_mapping.html). For the current study, Illumina-Affymetrix probe pairs were constructed if an Affymetrix probeset and an Illumina probe mapped to the same Ensembl transcript. This resulted in multiple probe pairs from some transcripts. E.g., if two different Affymetrix probesets (A1 and A2) and two Illumina probes (I1 and I2) mapped to the same transcript, this resulted in four possible probe pairs (A1–I1, A1–I2, A2–I1 and A2–I2).

### Pre-processing of Microarray Data

All pre-processing procedures were performed using the open source software R, available via www.bioconductor.org. Affymetrix gene expression values from dataset #1 and #2 were imported into R and extracted using the Robust Multichip Average (RMA) method [16] implemented in the *affy* R library. Illumina gene expression values from dataset #3 were imported and extracted using the *lum*i R library. Both methods for extracting expression values included quantile normalisation. Next, transcription values were inverse normal transformed, for each dataset separately, to obtain perfect normally distributed values where the mean is set to zero and standard deviation to one, as described previously [17]. Briefly, transcription values for all probes were first inverse normal transformed for each individual separately to adjust for variation between samples (e.g. RNA quantity). Second, the transcription values for all individuals within each substudy were inverse normal transformed for each probe, to adjust for variation between probes (e.g. probe specificity). This normalisation procedure produces comparable transcription values across individuals and transcripts (independent of RNA quality, tissue sampling method and platform etc.).

### Statistical Analyses of Microarray Data

The normalised transcription values from the three datasets were pooled and analysed using the moderated *t*-test implemented in *limma* R library [18], [19], available via the bioconductor project (www.bioconductor.org). First, the transcription values were fitted to a linear model with PE-status and gestational age as covariables, as well as a factor with values (1, 2 or 3) to separate the three studies. This factor allows the mean expression level for each transcript to differ between studies, which is expected if the samples included in three studies differed in terms of gestational age, different rate of PE incidence and sampling methods. Second, an empirical Bayesian method [18] was applied to the fitted model object. To correct for multiple testing, a Bonferroni adjusted *P* value of 0.05/33,088 (number of Illumina-Affymetrix probe pairs) = 1.51*10^−6^ was used as significance threshold in all analyses. To evaluate if the majority of the transcripts were regulated by gestational age or PE-status, the proportion of non-differentially expressed genes were estimated, as described previously [20]. In addition to the variables described above, we assessed the interaction between PE and gestational age by including an interaction term (PE*gestational age) in the linear model.

### Linear Model Fitting for Gestational Age

Gene expression levels are known to vary over gestation, but whether this relationship is linear throughout pregnancy is not known. In a previous study of gestational age-related transcriptional changes at the maternal-fetal interface [11] (dataset #1, Table 1), gestational age was categorised as either mid-gestation or term, whereas we have assumed a linear relationship between gestational age and transcription levels. To evaluate if these two methods for estimating gestational age effects provided similar results, we first reanalysed dataset #1 using the moderated *t*-test two times. The first time we used gestational age as a continuous variable, and the second time we dichotomised gestational age into mid-gestation and term. We then compared the log2 fold change (log2FC) values between analyses for each transcript.

### Ingenuity Pathway Analyses

We used Ingenuity Pathway Analysis (IPA) v7.5 (Ingenuity Systems, Redwood City, CA, USA) to study the biological function of the genes that were differentially expressed in association with PE or gestational age. Fischer’s exact test was used to investigate if any biological function were over-represented among these genes. No adjustment for multiple testing was done in the IPA analyses.

### Concordance with Previous Studies

To investigate whether our assessment of PE- and gestational age-related transcripts was similar to the individual three sub-studies included, we compared our results to those previously published [8], [9], [11]. However, as the three sub-studies originally were analysed using different methods and settings, results were not directly comparable. We therefore reanalysed each dataset (#1–#3) separately using the moderated *t*-test, and compared these results to the results when the three datasets were pooled. For each transcript, the log2FCs corresponding to PE and gestational age were compared across datasets. In addition, we compared the top 100 most significant results (ranked by *P* value) for PE- and gestational age-related transcripts between the datasets.

## Results

### Study Group Characteristics and Microarray Datasets

Genome-wide transcription data from 154 tissue samples from the maternal-fetal interface, originating from three different datasets (Table 1), were used in this work. A total of 105 non-preeclamptic (NP) controls with gestational age ranging between week 14 to 42 in addition to 49 PE cases with gestational age ranging from week 24 to 39 were included in the analyses (Table 1).

### Probe Remapping and Construction of Probe Pairs Between Microarray Platforms

A total of 33,088 Illumina-Affymetrix probe pairs (corresponding to 25,903 Affymetrix probesets and 17,933 Illumina probes) were identified by remapping probes from Affymetrix HG-U133A&B GeneChips and Illumina HumanWG-6 v2 BeadChips to Ensembl transcript predictions. These 33,088 probe pairs target 18,180 Ensembl transcripts representing 14,678 different HGNC (HUGO (Human Genome Organisation) Gene Nomenclature Committee) genes.

### Linear Model Fitting for Gestational Age

**Figure 1:**
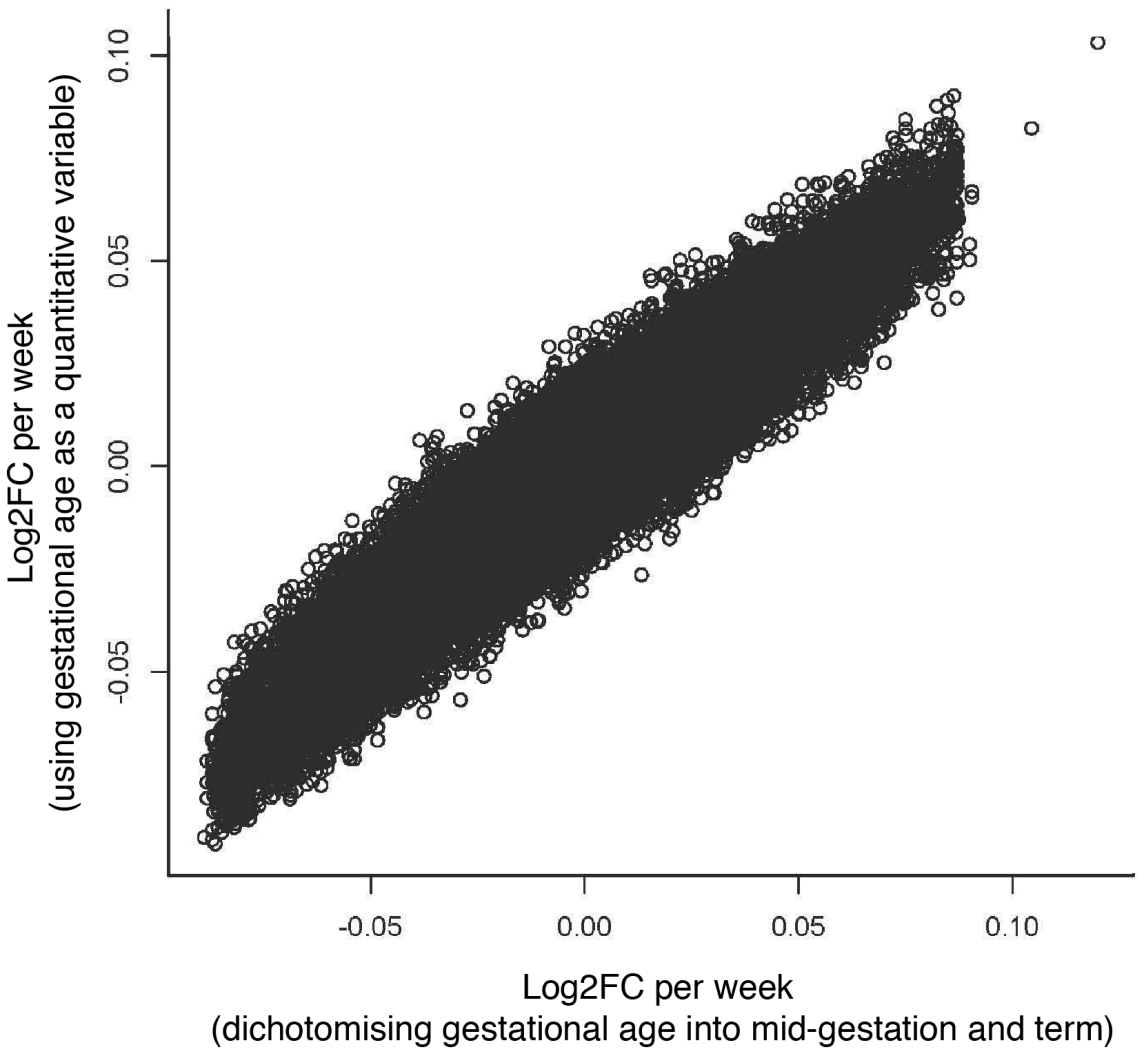
A comparison of two methods for identifying gestational age-related transcripts; using gestational age as a linear variable age or dichotomising it to midgestation and term. The correlation coefficient between the log2 fold change (log2FC) values in the two methods (Pearson’s product-moment correlation coefficient) is 0.94 (*P* value <2.2*10^−16^).

**Figure 2:**
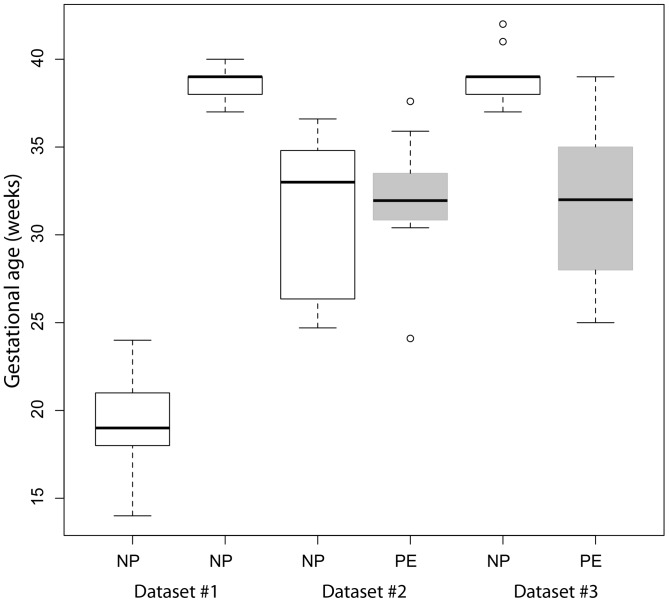
Distribution of gestational ages in the three datasets. The figure shows the median, 1^st^ and 3^rd^ quantile, range of the data and outliers. The grey boxes represent the preeclamptic cases (PE) and white boxes represent normal pregnancies (NP).

The comparison of the two different methods for identifying gestational age-related transcripts (using gestational age as a quantitative variable versus dichotomising it to mid-gestation and term) showed that these two methods gave similar results (Figure 1, Pearson’s product-moment correlation coefficient 0.94, *P*<2.2*10^−16^). Consequently, we conclude that gestational age can be used as a linear variable as well as being dichotomised into mid-gestation and term. Using a linear relationship allows for interpolation of the effect of gestational age throughout the total range of our data and compensates for the discrepancy in gestational age between datasets (Figure 2).

### PE- and Gestational Age-related Transcripts

**Figure 3:**
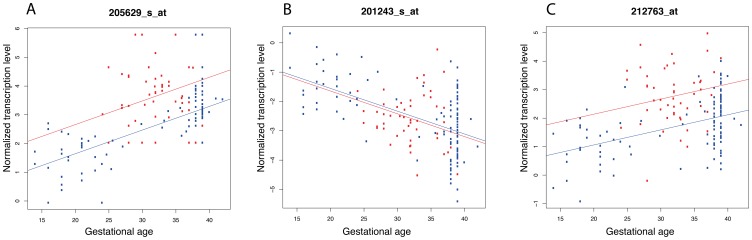
Variation in transcription values for three selected transcripts depending on gestational age. Transcription values (normalised) are plotted for different gestational ages (weeks). The red points represent preeclamptic (PE) pregnancies and blue points normal pregnancies (NP). The lines are the estimated regression lines for gestational age (red line for PE, blue line for NP), separated by the regression coefficient for PE-status. In Figure A, the transcription level is significantly associated with both PE-status and gestational age. In B, the transcription level is associated with gestational age only, and in C with PE-status only.

Of the 33,088 Illumina-Affymetrix probe pairs analysed, 29 were differentially expressed between PE and NP after correction for multiple testing (Table 2). Together, these probe pairs represents 22 different transcripts. In contrast, as many as 174 probe pairs, representing 92 different transcripts, were significantly associated with gestational age (Table 3). For the most significant observations, there were clear changes in transcription levels associated with either PE or gestational age (Figure 3, Figure S1 and S2). We did not detect any significant interactions between PE and gestational age. Two transcripts were associated with both PE-status and gestational age; fibroblast activation protein (*FAP*) and corticotropin releasing hormone (*CRH*). *FAP* was down-regulated in PE and decreased expression over gestation, whereas *CRH* was up-regulated in PE and increased expression over gestation. The estimated fraction of differentially expressed transcripts [20] was 49% for gestational age and 30% for PE. Since transcription values for the three datasets were standardised for each transcript prior to merging, all between-study-effects such as tissue source would be excluded. We did not allow for interaction between the study/tissue source and the effect of PE or gestational age on transcription values. To evaluate if the inclusion of controls with very low gestational age affected the results, we performed the same analyses excluding controls (n = 15) with gestational age <20 weeks. The results were very similar to the original results with a correlation between the –log10 (*P* values) for PE-associated transcripts of R = 0.96, and for the gestational age-associated transcripts r = 0.88. However, removing these 15 individuals decreased the number of significantly associated transcripts from 174 to 72 for gestational age, and from 29 to 18 for PE. Still, the ranking of the *P* values for the two subanalyses were the same (as indicated by the high correlation coefficients), and the decrease in number of significant findings most likely reflects the decrease in power by decreasing the sample size. Similarly, to test if the inclusion of cases with mild PE influenced the results, we performed the same analyses by excluding the seven cases with mild PE from dataset #3. These results are also very similar to the primary results with a correlation coefficient between the log2FC for PE-associated transcripts of R = 0.99.

**Table 2:** Preeclampsia-associated transcripts (Bonferroni corrected *P*<0.05, nominal *P*<1.52*10^−6^).

Gene symbol	Description	Fold change	*P* value	Illumina probe id	Affymetrix probe id
*AMD1*	adenosylmethionine decarboxylase 1	2.20	3.6E-08	ILMN_1788462	201196_s_at
*AMD1*	adenosylmethionine decarboxylase 1	2.00	1.2E-06	ILMN_1788462	201197_at
*ANGPTL2*	angiopoietin-like 2	−2.34	2.4E-09	ILMN_1772612	213001_at
*ANGPTL2*	angiopoietin-like 2	−2.03	7.3E-07	ILMN_1772612	213004_at
*CALML4*	calmodulin-like 4	−2.00	1.2E-06	ILMN_1652389	221879_at
*CAMSAP1L1*	calmodulin regulated spectrin-associatedprotein 1-like 1	2.11	1.6E-07	ILMN_1792660	212763_at
*CAMSAP1L1*	calmodulin regulated spectrin-associatedprotein 1-like 1	2.01	1.1E-06	ILMN_1792660	212765_at
*COL6A1*	collagen, type VI, alpha 1	−2.06	4.1E-07	ILMN_1732151	213428_s_at
*CRH*	corticotropin releasing hormone	2.02	8.5E-07	ILMN_1668035	205629_s_at
*EGFR*	epidermal growth factor receptor	2.06	3.9E-07	ILMN_1798975	201984_s_at
*FAP*	fibroblast activation protein, alpha	−1.99	1.5E-06	ILMN_1741468	209955_s_at
*FIBIN*	fin bud initiation factor homolog	−2.17	6.0E-08	ILMN_1716247	226769_at
*KIAA1919*	KIAA1919	2.00	1.2E-06	ILMN_1691916	232139_s_at
*KIAA1919*	KIAA1919	2.09	2.4E-07	ILMN_1691916	238828_at
*LIMCH1*	LIM and calponin homology domains 1	2.07	3.6E-07	ILMN_1664138	212325_at
*LIMCH1*	LIM and calponin homology domains 1	2.18	5.0E-08	ILMN_1664138	212327_at
*LIMCH1*	LIM and calponin homology domains 1	2.20	3.3E-08	ILMN_1664138	212328_at
*MAF*	v-maf musculoaponeurotic fibrosarcomaoncogene homolog	−2.05	4.5E-07	ILMN_1719543	206363_at
*NDFIP2*	Nedd4 family interacting protein 2	2.09	2.5E-07	ILMN_1677396	224799_at
*NDFIP2*	Nedd4 family interacting protein 2	1.99	1.4E-06	ILMN_1677396	224801_at
*NEBL*	nebulette	2.01	1.1E-06	ILMN_1808824	207279_s_at
*PLAC9*	placenta-specific 9	−1.99	1.4E-06	ILMN_1790859	227419_x_at
*PMP22*	peripheral myelin protein 22	−2.00	1.3E-06	ILMN_1785646	210139_s_at
*RARRES2*	retinoic acid receptor responder 2	−2.12	1.3E-07	ILMN_1811873	209496_at
*SCRN1*	secernin 1	−2.04	5.2E-07	ILMN_1756439	201462_at
*SERPINF1*	serpin peptidase inhibitor, clade F, member 1	−2.07	3.3E-07	ILMN_1685078	202283_at
*SLC39A8*	solute carrier family 39, member 8	2.04	6.1E-07	ILMN_1695316	219869_s_at
*SRPX*	sushi-repeat-containing protein, X-linked	−2.18	4.5E-08	ILMN_1709486	204955_at
*TP53INP2*	tumor protein p53 inducible nuclear protein 2	−2.10	2.0E-07	ILMN_1686906	224836_at

**Table 3:** Gestational age-associated transcripts (Bonferroni corrected *P*<0.05, nominal *P*<1.52^*^10^−6^).

Gene symbol	Description	Fold change(per week)	*P* value	Illumina probe id	Affymetrix probe id
*ADAMTS5*	ADAM metallopeptidase with thrombospondintype 1 motif, 5	−1.057	3.6E-07	ILMN_1671747	219935_at
*ADAMTS5*	ADAM metallopeptidase with thrombospondintype 1 motif, 5	−1.057	3.7E-07	ILMN_1671747	229357_at
*ADAMTS5*	ADAM metallopeptidase with thrombospondintype 1 motif, 5	−1.055	9.0E-07	ILMN_1671747	235368_at
*ANGPT2*	angiopoietin 2	−1.059	2.0E-07	ILMN_1774207	205572_at
*ANGPT2*	angiopoietin 2	−1.056	7.6E-07	ILMN_1774207	211148_s_at
*ANGPT2*	angiopoietin 2	−1.058	2.6E-07	ILMN_1774207	236034_at
*ANGPT2*	angiopoietin 2	−1.058	2.3E-07	ILMN_1774207	237261_at
*ANKRD50*	ankyrin repeat domain 50	−1.055	9.6E-07	ILMN_1729342	225731_at
*ANKRD50*	ankyrin repeat domain 50	−1.057	5.1E-07	ILMN_1729342	225735_at
*APH1B*	anterior pharynx defective 1 homolog B (C. elegans)	−1.055	9.3E-07	ILMN_1862217	226358_at
*ASPH*	aspartate beta-hydroxylase	-−1.055	1.0E-06	ILMN_1693771	224996_at
*ASPH*	aspartate beta-hydroxylase	−1.055	1.0E-06	ILMN_1739719	224996_at
*ASS1*	argininosuccinate synthase 1	−1.058	2.9E-07	ILMN_1688234	207076_s_at
*ATP1B1*	ATPase, Na+/K+ transporting, beta 1 polypeptide	−1.055	9.2E-07	ILMN_1736862	201243_s_at
*ATP2B4*	ATPase, Ca++ transporting, plasma membrane 4	−1.055	1.0E-06	ILMN_1664772	212136_at
*ATP6V1E1*	ATPase, H+ transporting, lysosomal 31kDa, V1subunit E1	1.055	1.1E-06	ILMN_1798485	208678_at
*BCAT1*	branched chain amino-acid transaminase 1, cytosolic	−1.055	1.2E-06	ILMN_1766169	225285_at
*BCAT1*	branched chain amino-acid transaminase 1, cytosolic	−1.057	5.1E-07	ILMN_1766169	226517_at
*BMP6*	bone morphogenetic protein 6	1.064	1.7E-08	ILMN_1747650	206176_at
*C1orf115*	chromosome 1 open reading frame 115	1.055	1.1E-06	ILMN_1674817	218546_at
*C20orf194*	chromosome 20 open reading frame 194	−1.056	7.1E-07	ILMN_1673005	225825_at
*C3orf58*	chromosome 3 open reading frame 58	−1.056	8.0E-07	ILMN_1797372	226464_at
*CAB39L*	calcium binding protein 39-like	−1.058	2.8E-07	ILMN_1730529	225914_s_at
*CAB39L*	calcium binding protein 39-like	−1.057	5.3E-07	ILMN_1660815	225914_s_at
*CAB39L*	calcium binding protein 39-like	1.060	1.3E-07	ILMN_1665449	226028_at
*CAPN6*	calpain 6	1.057	3.5E-07	ILMN_1782654	202965_s_at
*CCNYL1*	cyclin Y-like 1	−1.055	1.1E-06	ILMN_1810069	227280_s_at
*CD200*	CD200 molecule	1.065	1.2E-08	ILMN_1706722	209582_s_at
*CD200*	CD200 molecule	1.063	3.0E-08	ILMN_1706722	209583_s_at
*CDH11*	cadherin 11, type 2, OB-cadherin (osteoblast)	−1.059	1.5E-07	ILMN_1672611	207172_s_at
*CDH11*	cadherin 11, type 2, OB-cadherin (osteoblast)	−1.066	6.5E-09	ILMN_1672611	207173_x_at
*CEACAM1*	carcinoembryonic antigen-related cell adhesionmolecule 1 (biliary glycoprotein)	1.059	1.5E-07	ILMN_1716815	209498_at
*CEACAM1*	carcinoembryonic antigen-related cell adhesionmolecule 1 (biliary glycoprotein)	1.057	4.2E-07	ILMN_1664330	209498_at
*CEBPB*	CCAAT/enhancer binding protein (C/EBP), beta	1.056	8.0E-07	ILMN_1693014	212501_at
*CETP*	cholesteryl ester transfer protein, plasma	1.059	1.8E-07	ILMN_1681882	206210_s_at
*CITED2*	Cbp/p300-interacting transactivator, withGlu/Asp-rich carboxy-terminal domain, 2	−1.060	1.2E-07	ILMN_1663092	209357_at
*CMPK1*	cytidine monophosphate (UMP-CMP) kinase 1,cytosolic	−1.057	3.9E-07	ILMN_1738642	217870_s_at
*COL14A1*	collagen, type XIV, alpha 1	−1.055	9.5E-07	ILMN_1786598	212865_s_at
*COL1A2*	collagen, type I, alpha 2	−1.057	3.6E-07	ILMN_1785272	202403_s_at
*COL1A2*	collagen, type XXI, alpha 1	−1.060	1.1E-07	ILMN_1785272	202404_s_at
*COL21A1*	collagen, type III, alpha 1	−1.063	3.3E-08	ILMN_1732850	208096_s_at
*COL3A1*	collagen, type III, alpha 1	−1.060	1.3E-07	ILMN_1773079	201852_x_at
*COL3A1*	collagen, type III, alpha 1	−1.058	2.3E-07	ILMN_1773079	211161_s_at
*COL3A1*	collagen, type III, alpha 1	−1.058	3.4E-07	ILMN_1773079	215076_s_at
*COL5A1*	collagen, type V, alpha 1	−1.056	6.7E-07	ILMN_1706505	212489_at
*COL5A2*	collagen, type V, alpha 2	−1.059	1.9E-07	ILMN_1729117	221729_at
*COL5A2*	collagen, type V, alpha 2	−1.055	9.7E-07	ILMN_1729117	221730_at
*COL6A3*	collagen, type VI, alpha 3	−1.065	1.2E-08	ILMN_1706643	201438_at
*CRH*	corticotropin releasing hormone	1.059	2.0E-07	ILMN_1668035	205629_s_at
*CTSC*	cathepsin C	−1.059	2.2E-07	ILMN_1792885	225646_at
*CTSC*	cathepsin C	−1.056	6.3E-07	ILMN_1689086	225646_at
*CUX1*	cut-like homeobox 1	−1.055	1.2E-06	ILMN_1727603	225221_at
*DCN*	decorin	−1.059	1.7E-07	ILMN_1701748	209335_at
*DCN*	decorin	−1.058	2.9E-07	ILMN_1768227	209335_at
*DCN*	decorin	−1.057	5.1E-07	ILMN_1666672	209335_at
*DCN*	decorin	−1.055	9.4E-07	ILMN_1701748	211896_s_at
*DEPDC7*	DEP domain containing 7	−1.057	4.5E-07	ILMN_1718152	228293_at
*DUSP1*	dual specificity phosphatase 1	1.060	9.7E-08	ILMN_1781285	201041_s_at
*EFHD1*	EF-hand domain family, member D1	1.055	1.2E-06	ILMN_1779448	209343_at
*EHBP1*	EH domain binding protein 1	−1.056	6.3E-07	ILMN_1803348	212653_s_at
*ELOVL6*	ELOVL family member 6, elongation of long chainfatty acids (FEN1/Elo2, SUR4/Elo3-like, yeast)	−1.058	2.5E-07	ILMN_1700546	204256_at
*ERG*	v-ets erythroblastosis virus E26 oncogenehomolog (avian)	1.054	1.3E-06	ILMN_1768301	213541_s_at
*FABP4*	fatty acid binding protein 4, adipocyte	1.074	7.1E-11	ILMN_1773006	203980_at
*FADS1*	fatty acid desaturase 1	−1.058	2.5E-07	ILMN_1670134	208962_s_at
*FAP*	fibroblast activation protein, alpha	−1.055	1.1E-06	ILMN_1741468	209955_s_at
*FBXO42*	F-box protein 42	1.057	4.3E-07	ILMN_1756874	221813_at
*FKBP14*	FK506 binding protein 14, 22 kDa	−1.056	8.6E-07	ILMN_1665243	230728_at
*FKBP7*	FK506 binding protein 7	−1.057	4.2E-07	ILMN_1717737	224002_s_at
*FNBP1L*	formin binding protein 1-like	−1.058	3.3E-07	ILMN_1754600	215017_s_at
*GALNT3*	UDP-N-acetyl-alpha-D-galactosamine:polypeptideN-acetylgalactosaminyltransferase 3 (GalNAc-T3)	−1.056	8.3E-07	ILMN_1671039	203397_s_at
*GAS1*	growth arrest-specific 1	−1.056	6.6E-07	ILMN_1772910	204457_s_at
*GFOD2*	glucose-fructose oxidoreductase domaincontaining 2	1.055	1.1E-06	ILMN_1744006	221028_s_at
*GJA1*	gap junction protein, alpha 1, 43kDa	−1.062	5.3E-08	ILMN_1727087	201667_at
*GLB1*	galactosidase, beta 1	−1.058	2.5E-07	ILMN_1790862	201576_s_at
*GLUL*	glutamate-ammonia ligase	−1.061	8.8E-08	ILMN_1678881	215001_s_at
*GLUL*	glutamate-ammonia ligase	−1.056	6.3E-07	ILMN_1653496	215001_s_at
*GPSM1*	G-protein signaling modulator 1	−1.060	9.5E-08	ILMN_1751666	226043_at
*GPSM1*	G-protein signaling modulator 1	−1.056	6.3E-07	ILMN_1709307	226043_at
*GPSM1*	G-protein signaling modulator 1	−1.056	7.1E-07	ILMN_1796392	226043_at
*GPX8*	glutathione peroxidase 8 (putative)	−1.058	2.3E-07	ILMN_1767665	228141_at
*HAPLN1*	hyaluronan and proteoglycan link protein 1	−1.056	6.7E-07	ILMN_1678812	205524_s_at
*HBD*	hemoglobin, delta	1.061	5.5E-08	ILMN_1815527	206834_at
*HOMER1*	homer homolog 1 (Drosophila)	−1.056	7.2E-07	ILMN_1804568	213793_s_at
*HOMER1*	homer homolog 1 (Drosophila)	−1.057	4.5E-07	ILMN_1804568	226651_at
*IDH1*	isocitrate dehydrogenase 1 (NADP+), soluble	−1.061	6.0E-08	ILMN_1696432	201193_at
*IL6ST*	interleukin 6 signal transducer	−1.065	9.6E-09	ILMN_1849013	212195_at
*IL6ST*	interleukin 6 signal transducer	−1.061	5.8E-08	ILMN_1797861	212195_at
*IL6ST*	interleukin 6 signal transducer	−1.059	1.6E-07	ILMN_1746604	212195_at
*ITGA1*	integrin, alpha 1	−1.055	9.8E-07	ILMN_1802411	214660_at
*KCTD12*	potassium channel tetramerisationdomain containing 12	−1.057	4.7E-07	ILMN_1742332	212192_at
*KDELR3*	KDEL (Lys-Asp-Glu-Leu) endoplasmicreticulum protein retention receptor 3	−1.056	6.0E-07	ILMN_1713901	204017_at
*KIAA1598*	KIAA1598	−1.055	1.3E-06	ILMN_1805992	221802_s_at
*KLF2*	Kruppel-like factor 2 (lung)	−1.060	1.2E-07	ILMN_1735930	219371_s_at
*KREMEN1*	kringle containing transmembrane protein 1	1.059	2.1E-07	ILMN_1772697	227250_at
*LDLR*	low density lipoprotein receptor	−1.056	8.1E-07	ILMN_1651611	202068_s_at
*LGALS3*	lectin, galactoside-binding, soluble, 3	−1.055	1.2E-06	ILMN_1665479	208949_s_at
*LGALS3*	lectin, galactoside-binding, soluble, 3	−1.061	6.1E-08	ILMN_1747118	208949_s_at
*LGALS8*	lectin, galactoside-binding, soluble, 8	−1.059	1.8E-07	ILMN_1669930	208934_s_at
*LGALS8*	lectin, galactoside-binding, soluble, 8	1.059	2.1E-07	ILMN_1669930	208936_x_at
*LIMA1*	LIM domain and actin binding 1	1.056	6.7E-07	ILMN_1704369	217892_s_at
*LMO4*	LIM domain only 4	−1.055	9.4E-07	ILMN_1703487	227155_at
*LUM*	lumican	−1.056	7.7E-07	ILMN_1790529	201744_s_at
*MAN1A1*	mannosidase, alpha, class 1A, member 1	−1.056	6.8E-07	ILMN_1742187	221760_at
*MCC*	mutated in colorectal cancers	−1.063	3.0E-08	ILMN_1795503	226225_at
*MFSD2A*	major facilitator superfamily domaincontaining 2A	−1.054	1.5E-06	ILMN_1798284	225316_at
*MMRN2*	multimerin 2	1.055	1.0E-06	ILMN_1715788	219091_s_at
*NAP1L1*	nucleosome assembly protein 1-like 1	1.057	4.5E-07	ILMN_1705876	208752_x_at
*NAP1L1*	nucleosome assembly protein 1-like 1	−1.056	6.5E-07	ILMN_1705876	208753_s_at
*NAP1L1*	nucleosome assembly protein 1-like 1	−1.059	2.2E-07	ILMN_1699208	208753_s_at
*NAP1L1*	nucleosome assembly protein 1-like 1	−1.056	7.7E-07	ILMN_1705876	213864_s_at
*NAP1L1*	nucleosome assembly protein 1-like 1	−1.064	1.9E-08	ILMN_1699208	213864_s_at
*NDRG2*	NDRG family member 2	−1.061	7.4E-08	ILMN_1670535	206453_s_at
*NID1*	nidogen 1	1.055	9.5E-07	ILMN_1674719	202007_at
*NID2*	nidogen 2 (osteonidogen)	−1.060	1.4E-07	ILMN_1698706	204114_at
*NPL*	N-acetylneuraminate pyruvate lyase(dihydrodipicolinate synthase)	−1.062	5.3E-08	ILMN_1782070	221210_s_at
*OAT*	ornithine aminotransferase	−1.056	7.8E-07	ILMN_1654441	201599_at
*OLA1*	Obg-like ATPase 1	−1.058	2.4E-07	ILMN_1659820	219293_s_at
*OLFML3*	olfactomedin-like 3	−1.057	4.9E-07	ILMN_1727532	218162_at
*PAPLN*	papilin, proteoglycan-like sulfatedglycoprotein	−1.060	9.7E-08	ILMN_1710495	226435_at
*PCYOX1*	prenylcysteine oxidase 1	−1.061	8.3E-08	ILMN_1679725	225274_at
*PDGFRA*	platelet-derived growth factor receptor,alpha polypeptide	−1.055	1.2E-06	ILMN_1681949	203131_at
*PDZRN3*	PDZ domain containing ring finger 3	−1.056	8.5E-07	ILMN_1699552	212915_at
*PDZRN3*	PDZ domain containing ring finger 3	−1.055	1.2E-06	ILMN_1703511	212915_at
*PLA2G7*	phospholipase A2, group VII (platelet-activating factor acetylhydrolase, plasma)	−1.061	7.4E-08	ILMN_1701195	206214_at
*PPL*	periplakin	1.058	2.4E-07	ILMN_1806030	203407_at
*PPP1R14C*	protein phosphatase 1, regulatory(inhibitor) subunit 14C	1.056	8.4E-07	ILMN_1664855	226907_at
*PTPRB*	RAB6B, member RAS oncogene family	1.055	1.0E-06	ILMN_1821052	205846_at
*RAB6B*	roundabout, axon guidance receptor,homolog 1 (Drosophila)	1.059	1.4E-07	ILMN_1752299	225259_at
*ROBO1*	ribosomal protein, large, P0	−1.060	1.1E-07	ILMN_1806790	213194_at
*RPLP0*	ribosomal protein, large, P0	−1.059	1.5E-07	ILMN_1709880	201033_x_at
*RPLP0*	ribosomal protein, large, P0	−1.057	5.3E-07	ILMN_1748471	201033_x_at
*RPLP0*	ribosomal protein, large, P0	−1.055	9.9E-07	ILMN_1709880	208856_x_at
*RPLP0*	ribosomal protein, large, P0	−1.057	4.2E-07	ILMN_1709880	211720_x_at
*RPLP0*	ribosomal protein, large, P0	−1.055	1.2E-06	ILMN_1709880	211972_x_at
*RPS2*	ribosomal protein S2, pseudogene 6	−1.061	6.4E-08	ILMN_1662982	203107_x_at
*RPS2*	ribosomal protein S2, pseudogene 6	−1.063	2.9E-08	ILMN_1662982	212433_x_at
*RPS2*	ribosomal protein S2, pseudogene 6	−1.061	8.7E-08	ILMN_1709604	212433_x_at
*SC4MOL*	sterol-C4-methyl oxidase-like	−1.058	3.0E-07	ILMN_1720889	209146_at
*SC4MOL*	sterol-C4-methyl oxidase-like	−1.057	4.8E-07	ILMN_1689842	209146_at
*SESTD1*	SEC14 and spectrin domains 1	−1.059	2.0E-07	ILMN_1782341	226763_at
*SLC2A10*	solute carrier family 2 (facilitatedglucose transporter), member 10	−1.062	4.2E-08	ILMN_1663351	221024_s_at
*SLC39A10*	solute carrier family 39 (zinctransporter), member 10	−1.055	1.1E-06	ILMN_1656129	225295_at
*SMYD3*	SET and MYND domain containing 3	−1.056	7.8E-07	ILMN_1741954	218788_s_at
*SNORA10*	small nucleolar RNA, H/ACA box 10	−1.059	1.9E-07	ILMN_1709604	203107_x_at
*SOAT1*	sterol O-acyltransferase 1	−1.062	4.8E-08	ILMN_1699100	221561_at
*SOAT1*	sterol O-acyltransferase 1	−1.062	4.1E-08	ILMN_1699100	228479_at
*SPP1*	secreted phosphoprotein 1	−1.059	2.0E-07	ILMN_1651354	209875_s_at
*STAT5B*	signal transducer and activator oftranscription 5B	1.055	1.2E-06	ILMN_1777783	205026_at
*STX3*	syntaxin 3	1.056	5.9E-07	ILMN_1659544	209238_at
*SVIL*	supervillin	−1.054	1.3E-06	ILMN_1671404	202565_s_at
*SYNPO*	synaptopodin	1.057	3.6E-07	ILMN_1711491	202796_at
*TCF7L2*	transcription factor 7-like 2(T-cell specific, HMG-box)	−1.055	1.1E-06	ILMN_1672486	212759_s_at
*TCF7L2*	transcription factor 7-like 2(T-cell specific, HMG-box)	−1.059	1.7E-07	ILMN_1672486	212761_at
*TCF7L2*	transcription factor 7-like 2(T-cell specific, HMG-box)	−1.058	2.6E-07	ILMN_1672486	212762_s_at
*TCF7L2*	transcription factor 7-like 2(T-cell specific, HMG-box)	−1.063	2.3E-08	ILMN_1672486	216035_x_at
*TCF7L2*	transcription factor 7-like 2(T-cell specific, HMG-box)	−1.060	1.4E-07	ILMN_1672486	216037_x_at
*TMEM150C*	transmembrane protein 150C	−1.056	8.3E-07	ILMN_1797047	229623_at
*TPPP3*	tubulin polymerization-promoting proteinfamily member 3	1.057	3.9E-07	ILMN_1797744	218876_at
*TTC3L*	tetratricopeptide repeat domain 3	−1.055	9.7E-07	ILMN_1761476	210645_s_at
*TUBB*	tubulin, beta	−1.056	6.1E-07	ILMN_1665583	212320_at
*TUBB*	tubulin, beta	−1.054	1.4E-06	ILMN_1703692	212320_at
*unspecific*		−1.060	1.1E-07	ILMN_1748471	211720_x_at
*unspecific*		−1.058	3.5E-07	ILMN_1748471	211972_x_at
*UQCRH*	ubiquinol-cytochrome c reductase hingeprotein-like	−1.055	1.1E-06	ILMN_1718136	202233_s_at
*VCAN*	versican	−1.057	3.6E-07	ILMN_1687301	204619_s_at
*VCAN*	versican	−1.058	2.4E-07	ILMN_1687301	204620_s_at
*VCAN*	versican	−1.058	2.5E-07	ILMN_1687301	211571_s_at
*VCAN*	versican	−1.058	2.3E-07	ILMN_1687301	215646_s_at
*WARS*	tryptophanyl-tRNA synthetase	1.055	1.1E-06	ILMN_1727271	200629_at
*WNT5A*	wingless-type MMTV integration site family,member 5A	−1.054	1.4E-06	ILMN_1800317	213425_at
*XPOT*	exportin, tRNA	−1.055	1.3E-06	ILMN_1743711	212160_at
*ZFHX3*	zinc finger homeobox 3	−1.060	1.2E-07	ILMN_1808587	226137_at
*ZFP42*	zinc finger protein 42 homolog (mouse)	−1.054	1.4E-06	ILMN_1751127	243161_x_at
*ZKSCAN1*	zinc finger with KRAB and SCAN domains 1	−1.055	1.0E-06	ILMN_1687567	225935_at

### Ingenuity Pathway Analyses

IPA analysis of the 22 PE-associated transcripts (Table 2) identified in our analyses demonstrated an over-representation of biological functions such as cellular growth and proliferation, cell death, endocrine system disorders and metabolic disease (Table 4). IPA analysis of the 92 gestational age-associated transcripts (Table 3) demonstrated over-representation of the biological functions cellular assembly and organisation, tissue development, cellular movement, cardiovascular system development and function, cellular growth and proliferation, connective tissue development, cell-to-cell signalling and interaction, and cell cycle (Table 5).

**Table 4:** Ingenuity Pathway Analysis of preeclampsia-associated transcripts.

Biological functions	Genes included	*P* value
Cellular growth and proliferation	*COL6A1, CRH, EGFR, PMP22, SERPINF1*	2.92*10^−4^−4.40*10^−2^
Cell death	*CRH, EGFR, FAP, PMP22, SERPINF1*	1.28*10^−3^−1.53*10^−2^
Endocrine system disorders	*CAMSAP1L1, CRH, EGFR, KIAA1919, LIMCH1, NEBL, PMP22, SCRN1, SERPINF1*	2.57*10^−3^−2.86*10^−2^
Metabolic disease	*CAMSAP1L1, CRH, KIAA1919, LIMCH1, NEBL, PMP22, SCRN1, SERPINF1*	2.30*10^−3^−2.57*10^−2^

**Table 5:** Ingenuity Pathway Analysis of gestational age-associated transcripts.

Biological functions	Genes included	*P* value
Cellular assembly and organization	*COL1A2, COL3A1, COL5A1, COL5A2, LIMA1, VCAN, IL6ST, NID1, LGALS3*	1.88*10^−7^−1.12*10^−2^
Tissue development	*BMP6, CDH11, CITED2, COL1A2, COL3A1, COL5A1, COL5A2, COL6A3, DCN, ERG,* *IL6ST, ITGA1, KLF2, LGALS3, LGALS8, NID1, SVIL, TCF7L2, VCAN, WNT5A, SPP1*	3.31*10^−5^−1.05*10^−2^
Cellular movement	*BMP6, CDH11, CEACAM1, CITED2, DCN, FAP, GJA1, ITGA1, LGALS3, PDGFRA,* *ROBO1, SPP1, TCF7L2, VCAN, WARS, WNT5A*	5.35*10^−5^−1.02*10^−2^
Cardiovascular system developmentand function	*BMP6, CEACAM1, CITED2, COL1A2, COL3A1, DCN, ITGA1, TCF7L2, WARS, WNT5A*	2.82*10^−4^−2.19*10^−2^
Cellular growth and proliferation	*BCAT1, BMP6, CDH11, CEACAM1, CEBPB, CITED2, COL6A3, COL14A1, CRH, CTSC,* *CUX1, DCN, DUSP1, ERG, FADS1, GAS1, GJA1, GLUL, IL6ST, ITGA1, KLF2, LGALS3,* *MCC, NAP1L1, PDGFRA, SOAT1, SPP1, TCF7L2, VCAN, WARS, WNT5A, ZFHX3*	4.04*10^−4^−1.46*10^−2^
Connective tissue development	*CDH11, COL14A1, IL6ST, PDGFRA, SPP1, TCF7L2, VCAN, WNT5A*	1.04*10^−3^−1.46*10^−2^
Cell-to-cell signaling and interaction	*BMP6, CD200,CEACAM1,CEBPB, CRH, DCN, ERG, IL6ST, ITGA1, LDLR, LGALS3,* *LGALS8, NID1, PDGFRA, SPP1, VCAN, WNT5A*	3.96*10^−3^−1.46*10^−2^
Cell cycle	*BCAT1, BMP6, CEBPB, CRH, DCN, DUSP1, GJA1, LGALS3*	7.35*10^−3^−1.22*10^−2^

### Concordance with Previous Studies

Since the number of significant transcripts in the previous publications of the included datasets was very limited when applying a stringent threshold for multiple testing (Bonferroni), no rigorously comparison between datasets could be performed. However, lists of transcripts/probes that were claimed to be differentially expressed in the three original studies are included (Tables S1–S3). A comparison of our reanalysis of dataset #1 with 36 NP samples and our analyses of all three datasets combined with 154 samples showed that the log2FC values for gestational age-associated transcripts were highly correlated (Pearson’s product-moment correlation coefficient 0.94, *P*<2.2*10^−16^). In addition, the top 100 transcripts from the reanalysis of dataset #1 were all nominally significant (*P*<0.05) in our analysis of the pooled datasets and 20 transcripts were shared between the top 100 transcripts from dataset #1 and the pooled analyses.

Comparing our separate reanalyses of dataset #2 and #3 (with 12 or 37 PE samples and 11 or 58 NP samples, respectively) with our analysis of the pooled dataset (49 PE and 105 NP), we found that the correlation between the log2FC values for PE in dataset #2 and the pooled dataset (Pearson’s product-moment correlation coefficient 0.57, *P*<2.2*10^−16^) were similar to the correlation between the PE log2FC values for dataset #3 and the pooled dataset (Pearson’s product-moment correlation coefficient 0.71, *P*<2.2*10^−16^). For the sub-analysis of PE-associated transcripts in dataset #2, 78 of the top 100 transcripts were nominally significant (*P*<0.05) in our pooled analyses of PE-associated transcripts, but only two of these were significant in the pooled analyses after correction for multiple testing and four transcripts were shared between the top 100 transcripts from dataset #2 and the pooled analyses. For the reanalysis of dataset #3, 93 of the top 100 transcripts were nominally significant (*P*<0.05) in our total analysis, of which nine were significant in the pooled dataset after correction for multiple testing. Seven transcripts were shared between the top 100 transcripts from dataset #3 and the pooled analyses. The correlation between dataset #2 and #3 log2FC values from the PE-associated transcripts was low (Pearson’s product-moment correlation coefficient 0.04, *P*<1.04*10^−13^). This is not surprising, since none of the PE-associated transcripts in dataset #2 and only two in dataset #2 were significant after correcting for multiple testing (Bonferroni).

## Discussion

In this work, we have pooled data from three different genome-wide transcription analyses of samples from the maternal-fetal interface to generate a dataset consisting of 154 samples. To the best of our knowledge, this is the largest dataset used to assess changes in transcription levels associated with either PE or gestational age. A total of 92 gestational age-related and 22 PE-associated transcripts were identified. These numbers by themselves indicates that a large fraction of variance in transcription levels at the maternal-fetal interface can be attributed to gestational age rather than PE-status. The large sample size achieved by pooling datasets from three different studies enabled us to apply a stringent significance threshold (Bonferroni adjusted *P* values) in order to minimise the probability of false positives. Using Bonferroni cut-off of 0.05 corresponds to false discovery rate (FDR) cut-offs of 2.8*10^−4^ for gestational age and 1.7*10^−3^ for PE [21]. Previous genome-wide transcription studies on PE have not used such stringent threshold for significance, and those findings should be interpreted with care. In accordance with this, only few PE- or gestational age- associated transcripts from previous publications have been replicated in others or our study.

The most significant finding among the PE-associated transcripts was the down-regulation (*P* = 2.43*10^−9^, >2 fold down-regulated) of angiopoietin-like 2 (*ANGPTL2*). The *ANGPTL2* protein is a secreted glycoprotein with homology to the angiopoietins, which are important angiogenic factors. Although the angiopoietin-like proteins do not bind to the angiopoietin receptor, they are believed to play a role in angiogenesis via induction of endothelial cell sprouting in blood vessels [22]. We also observed a down-regulation of *RARRES2* (retinoic acid receptor responder 2, also called chemerin) among the PE-associated transcripts. It was recently demonstrated that chemerin could induce angiogenesis *in vitro* [23], [24]. Down-regulation of these angiogenic factors may be linked to the pathogenesis of PE through abnormal vascular morphology, as placentas from women with severe PE, especially in combination with fetal growth restriction, are characterised by decreased capillary volume and surface area [25].

The expression of *CRH* and *FAP* were significantly associated with both PE-status and gestational age. While the expression of *CRH* increased with gestational age and was up-regulated in PE, the expression of *FAP* decreased with gestational age and was down-regulated in PE. The transcriptional changes of *FAP* and *CRH* are probably linked to both gestational age and PE. During pregnancy, *CRH* is produced by decidual and placental tissue [26], and released into the fetal and maternal circulation. Maternal plasma *CRH* levels increase over gestation [27], concurrent with our observation of *CRH* among the gestational age-related transcripts. A further elevation of maternal plasma *CRH* levels has been shown in PE compared to normal pregnancies at the same gestational age [28]. Increased levels of *CRH* may contribute to the pathogenesis of PE trough its role in regulation of vascular resistance and blood flow in utero-placental tissue [29].

Among the transcripts that were up- or down- regulated due to gestational age, we noted decreased expression of galectin 3 (*LGALS3*) and increased expression of galectin (*LGALS8*). Galectins are highly expressed at the maternal-fetal interface, and regarded as multifunctional regulators of fundamental cellular processes due to their capacity to modulate functions such as cell-extracellular matrix interactions, proliferation, adhesion, and invasion [30]. Galectin 3 is expressed in placental cell columns, but not in invasive extravillous trophoblasts [31]. The negative correlation between expression and trophoblast invasiveness is in accordance with our finding of decreased galectin 3 expression over gestation. Galectin 8 is expressed by decidual cells, villous and extravillous trophoblasts [32], but its role is less clearly understood.

The IPA analyses of the 22 PE-associated transcripts demonstrated an over-representation of genes associated with metabolic disease (Table 4). PE share several metabolic abnormalities with cardiovascular diseases and diabetes, and having a PE complicated pregnancy is associated with increased risk of type 2 diabetes later in life [33]. This agrees with pregnancy acting as a stress factor which could reveal a pre-existing disposition to later life metabolic disease [34]. IPA analysis of the 92 transcripts that were associated with gestational age revealed an over-representation of genes involved in cell assembly and organisation, tissue development, cellular movement, tissue morphology, and connective tissue development and function (Table 5). These findings are in agreement with known biological processes taking place at the maternal-fetal interface during pregnancy, such as trophoblast proliferation, differentiation, invasion and extracellular matrix remodelling.

It is important to consider that the data used in our study was produced on two different microarray platforms, and that tissue sampling procedures differed between the three sub-studies included in our dataset. In dataset #1 and #2, basal plate biopsies were used for transcriptional analyses, whereas in dataset #3, decidual tissue was collected by vacuum suction of the entire placental bed. These differences may pose a potential bias, as gene expression has been shown to differ depending on tissue sampling site [35]. Inter- and intra-platform reproducibility has been shown to be good in terms of detecting differentially expressed genes [36]–[38]. However, the reproducibility of absolute transcription levels is poor, and pre-processing is required before comparisons of transcription values across platforms can be made. To deal with these limitations, and be able to make both inter- and intra-platform comparisons of transcription values, we performed inverse normal transformation. After this transformation, the distribution of transcription values is assumed equal for all probes, independent of study, sampling method and platform. However, PE-status, gestational age and tissue sampling method differed between studies, and consequently we had to include this as a factor giving a separate level for each study in the linear regression model. In our analyses, we exclusively searched for observations that agreed between datasets, and the limitations mentioned above will rather reduce the power of identifying differentially expressed genes than introduce false positive results. Combined with the fact that our pooled dataset only targets 14,678 genes, and that we are using a very stringent threshold for significance, our results likely represent only a small fraction of the total number of transcripts that are influenced by either PE or gestational age. Had all samples been collected by the same method and analysed on the same arrays, it is likely that a much larger number of differentially expressed transcripts would be identified. In our analyses, we did not allow for interaction between the study/tissue source and the effect of PE or gestational age on transcription values. The main reason for this is that the cause of such interaction would be impossible to determine (e.g. sampling method, microarray type, PE heterogeneity etc.). Instead, we focused on identifying shared effects across studies.

Another limitation in our dataset is the heterogeneity of the PE group. In dataset #2, all cases had severe PE of which 5 were complicated by FGR. In dataset #3 30 of 37 cases had severe PE or PE complicated by SGA, seven had mild PE. We recognise that this may indicate that some of our PE-associated transcripts may be linked to SGA pathogenesis or restricted to severe PE, and that it would have been preferable to use a more homogenous case group. The sample size of PE cases was too small to allow for any stratification in the analyses. It is therefore important to consider that our results might not be generalisable to all kinds of PE. However, PE is a complex disorder. Classifying individuals into e.g. severe and mild PE does not necessarily mean that these are different disorders. Rather, the diagnosis of both mild and severe PE are combinations of different quantitative characteristics, and at some pre-defined cut-off, the disorder is regarded as severe. This means that the underlying causes of PE might be as complex within the patients with severe PE as between cases with severe and mild PE. The NP group is also heterogeneous, including samples from preterm labour without infection (1/3 of these due to cervical insufficiency), elective terminations (of which the future pregnancy outcome is unknown), as well as normal pregnant women. Including cases with preterm labour may have influenced our results, as this condition with or without infection is likely to result from some sort of pathology that could result in gene expression changes. A possible shared pathology between preterm birth or PE might also result in similar changes in gene expression, which would reduce our power to identify PE-specific differentially expressed genes. However, it is almost impossible to match severe PE cases with regards to gestational age by using completely healthy individuals, as normal pregnancies in this gestational age range (week 24–36) are rare. The inclusion of samples with gestational age <20 weeks may also have confounded our results, as the future pregnancy outcome is unknown, and they may have developed PE later in pregnancy. However, excluding these samples from the analyses did not change the results, but rather decreased the power due to the smaller sample size.

Unfortunately, we were not able validate the microarray results, e.g. through quantitative real-time (qRT-) PCR, for all our significant findings, mainly due to lack of RNA. However, validations have previously been performed for a subset of our genes: *ANGPTL2, CRH, TCF7L2, PLA2G7 and TCF7L2* [8], [11] with good results. Even though more comprehensive corroborative studies might have be useful for verifying the results, there are many examples where this is not feasible, e.g. due to lack of RNA [39]. It is also worth considering that microarray data are generally much more accurate compared to qRT-PCR. For qRT-PCR, housekeeping genes are assumed to be equally expressed in all samples, and commonly used for normalisation procedures. However, it has been shown that the expression of housekeeping genes vary dramatically between individuals and that the heritability is as high as 0.56 for some housekeeping genes [17]. Consequently, housekeeping genes are not ideal for internal standardisation. The normalisation procedure performed for microarray experiments, based on the average expression level per individual, is likely to generate more precise estimates.

To our knowledge, this is the first study to simultaneously assess the effects of gestational age and PE-status on gene expression at the maternal-fetal interface. We found that a much large number of transcripts were influenced by gestational age compared to PE status. Based on this, we strongly recommend that adjustments for gestational age should be performed in similar studies. The large sample size achieved by pooling different datasets allowed us to apply a stringent threshold for significance, which has not been feasible in most previous studies. The transcripts identified in this study are likely to be influenced by gestational age or disease status, or even play a direct role in the development of PE.

## Supporting Information

Figure S1 **Variation in transcription values depending on gestational age for all transcripts that were significantly associated with preeclampsia (PE).** Normalised transcription values are plotted for different gestational ages (weeks). The red points represent PE pregnancies and blue points normal pregnancies (NP). The lines are the estimated regression lines for gestational age (red line for PE and blue line for NP), separated by the regression coefficient for PE-status.
(PDF)

Figure S2 **Variation in transcription values depending on gestational age for all transcripts that were significantly associated with gestational age.** Normalised transcription values are plotted for different gestational ages (weeks). The red points represent preeclamptic (PE) pregnancies, and blue points normal pregnancies (NP). The lines are the estimated regression lines for gestational age (red line for PE and blue line for NP), separated by the regression coefficient for PE-status.
(PDF)

Table S1 Genes reported as differentially expressed in PE in the study by Winn et al. [9].
(XLSX)

Table S2 Genes reported as differentially expressed between between midgestation and term in the study by Winn et al. [11].
(XLSX)

Table S3 Genes reported as differentially expressed in PE in the study by Loset et al. [8].
(XLSX)

## References

[1] Lain KY, Roberts JM (2002) Contemporary concepts of the pathogenesis and management of preeclampsia. Jama 287: 3183–3186. 12076198 10.1001/jama.287.24.3183

[2] Roberts JM, Pearson G, Cutler J, Lindheimer M (2003) Summary of the NHLBI Working Group on Research on Hypertension During Pregnancy. Hypertension 41: 437–445. 12623940 10.1161/01.HYP.0000054981.03589.E9

[3] Witlin AG, Sibai BM (1997) Hypertension in pregnancy: current concepts of preeclampsia. Annu Rev Med 48: 115–127. 9046950 10.1146/annurev.med.48.1.115

[4] Roberts JM, Gammill HS (2005) Preeclampsia: recent insights. Hypertension 46: 1243–1249. 16230510 10.1161/01.HYP.0000188408.49896.c5

[5] Goldenberg RL, Rouse DJ (1998) Prevention of premature birth. N Engl J Med 339: 313–320. 9682045 10.1056/NEJM199807303390506

[6] Redman CW, Sargent IL (2010) Immunology of pre-eclampsia. Am J Reprod Immunol 63: 534–543. 20331588 10.1111/j.1600-0897.2010.00831.x

[7] Lian IA, Toft JH, Olsen GD, Langaas M, Bjorge L, et al (2010) Matrix metalloproteinase 1 in pre-eclampsia and fetal growth restriction: reduced gene expression in decidual tissue and protein expression in extravillous trophoblasts. Placenta 31: 615–620. 20452670 10.1016/j.placenta.2010.04.003

[8] Loset M, Mundal SB, Johnson MP, Fenstad MH, Freed KA, et al.. (2011) A transcriptional profile of the decidua in preeclampsia. Am J Obstet Gynecol 204: 84 e81–27. 10.1016/j.ajog.2010.08.043 PMC3011026 20934677

[9] Winn VD, Gormley M, Paquet AC, Kjaer-Sorensen K, Kramer A, et al (2009) Severe preeclampsia-related changes in gene expression at the maternal-fetal interface include sialic acid-binding immunoglobulin-like lectin-6 and pappalysin-2. Endocrinology 150: 452–462. 18818296 10.1210/en.2008-0990 PMC2630905

[10] Mikheev AM, Nabekura T, Kaddoumi A, Bammler TK, Govindarajan R, et al (2008) Profiling gene expression in human placentae of different gestational ages: an OPRU Network and UW SCOR Study. Reprod Sci 15: 866–877. 19050320 10.1177/1933719108322425 PMC2702165

[11] Winn VD, Haimov-Kochman R, Paquet AC, Yang YJ, Madhusudhan MS, et al (2007) Gene expression profiling of the human maternal-fetal interface reveals dramatic changes between midgestation and term. Endocrinology 148: 1059–1079. 17170095 10.1210/en.2006-0683

[12] Pregnancy NHBPEPWGoHBPi (2000) Report of the National High Blood Pressure Education Program Working Group on High Blood Pressure in Pregnancy. Am J Obstet Gynecol 183: S1–S22. 10920346

[13] Schroeder BM (2002) ACOG practice bulletin on diagnosing and managing preeclampsia and eclampsia. American College of Obstetricians and Gynecologists. Am Fam Physician 66: 330–331. 12152970

[14] Ballester B, Johnson N, Proctor G, Flicek P (2010) Consistent annotation of gene expression arrays. BMC Genomics 11: 294. 20459806 10.1186/1471-2164-11-294 PMC2894801

[15] Slater GS, Birney E (2005) Automated generation of heuristics for biological sequence comparison. BMC Bioinformatics 6: 31. 15713233 10.1186/1471-2105-6-31 PMC553969

[16] Irizarry RA, Hobbs B, Collin F, Beazer-Barclay YD, Antonellis KJ, et al (2003) Exploration, normalization, and summaries of high density oligonucleotide array probe level data. Biostatistics 4: 249–264. 12925520 10.1093/biostatistics/4.2.249

[17] Goring HH, Curran JE, Johnson MP, Dyer TD, Charlesworth J, et al (2007) Discovery of expression QTLs using large-scale transcriptional profiling in human lymphocytes. Nat Genet 39: 1208–1216. 17873875 10.1038/ng2119

[18] Smyth GK (2004) Linear models and empirical bayes methods for assessing differential expression in microarray experiments. Stat Appl Genet Mol Biol 3: Article3. 16646809 10.2202/1544-6115.1027

[19] Smyth GK (2005) Limma: linear models for microarray data.; Gentleman R, Carey V, Dudoit S, Irizarry R, Huber W, editors. New York: Springer.

[20] Langaas M, Lindqvist BH, Ferkingstad E (2005) Estimating the proportion of true null hypotheses, with application to DNA microarray data. Journal of the Royal Statistical Society Series B-Statistical Methodology 67: 555–572.

[21] Benjamini Y, Yekutieli D (2001) The control of the false discovery rate in multiple testing under dependency. Annals of Statistics 29: 1165–1188.

[22] Kim I, Moon SO, Koh KN, Kim H, Uhm CS, et al (1999) Molecular cloning, expression, and characterization of angiopoietin-related protein. angiopoietin-related protein induces endothelial cell sprouting. J Biol Chem 274: 26523–26528. 10473614 10.1074/jbc.274.37.26523

[23] Bozaoglu K, Curran JE, Stocker CJ, Zaibi MS, Segal D, et al (2010) Chemerin, a novel adipokine in the regulation of angiogenesis. J Clin Endocrinol Metab 95: 2476–2485. 20237162 10.1210/jc.2010-0042 PMC2869547

[24] Kaur J, Adya R, Tan BK, Chen J, Randeva HS (2010) Identification of chemerin receptor (ChemR23) in human endothelial cells: chemerin-induced endothelial angiogenesis. Biochem Biophys Res Commun 391: 1762–1768. 20044979 10.1016/j.bbrc.2009.12.150

[25] Egbor M, Ansari T, Morris N, Green CJ, Sibbons PD (2006) Pre-eclampsia and fetal growth restriction: how morphometrically different is the placenta? Placenta 27: 727–734. 16125226 10.1016/j.placenta.2005.06.002

[26] Wetzka B, Sehringer B, Schafer WR, Biller S, Hor C, et al (2003) Expression patterns of CRH, CRH receptors, and CRH binding protein in human gestational tissue at term. Exp Clin Endocrinol Diabetes 111: 154–161. 12784189 10.1055/s-2003-39778

[27] McLean M, Bisits A, Davies J, Woods R, Lowry P, et al (1995) A placental clock controlling the length of human pregnancy. Nat Med 1: 460–463. 7585095 10.1038/nm0595-460

[28] Perkins AV, Linton EA, Eben F, Simpson J, Wolfe CD, et al (1995) Corticotrophin-releasing hormone and corticotrophin-releasing hormone binding protein in normal and pre-eclamptic human pregnancies. Br J Obstet Gynaecol 102: 118–122. 7756202 10.1111/j.1471-0528.1995.tb09063.x

[29] Clifton VL, Read MA, Leitch IM, Giles WB, Boura AL, et al (1995) Corticotropin-releasing hormone-induced vasodilatation in the human fetal-placental circulation: involvement of the nitric oxide-cyclic guanosine 3′,5′-monophosphate-mediated pathway. J Clin Endocrinol Metab 80: 2888–2893. 7559870 10.1210/jcem.80.10.7559870

[30] Than NG, Romero R, Kim CJ, McGowen MR, Papp Z, et al.. (2011) Galectins: guardians of eutherian pregnancy at the maternal-fetal interface. Trends Endocrinol Metab. 10.1016/j.tem.2011.09.003 PMC3640805 22036528

[31] van den Brule FA, Price J, Sobel ME, Lambotte R, Castronovo V (1994) Inverse expression of two laminin binding proteins, 67LR and galectin-3, correlates with the invasive phenotype of trophoblastic tissue. Biochem Biophys Res Commun 201: 388–393. 8198600 10.1006/bbrc.1994.1713

[32] Kolundzic N, Bojic-Trbojevic Z, Radojcic L, Petronijevic M, Vicovac L (2011) Galectin-8 is expressed by villous and extravillous trophoblast of the human placenta. Placenta 32: 909–911. 21862124 10.1016/j.placenta.2011.07.087

[33] Carr DB, Newton KM, Utzschneider KM, Tong J, Gerchman F, et al (2009) Preeclampsia and risk of developing subsequent diabetes. Hypertens Pregnancy 28: 435–447. 19843005 10.3109/10641950802629675

[34] Williams D (2003) Pregnancy: a stress test for life. Curr Opin Obstet Gynecol 15: 465–471. 14624211 10.1097/00001703-200312000-00002

[35] Sood R, Zehnder JL, Druzin ML, Brown PO (2006) Gene expression patterns in human placenta. Proc Natl Acad Sci U S A 103: 5478–5483. 16567644 10.1073/pnas.0508035103 PMC1414632

[36] Barnes M, Freudenberg J, Thompson S, Aronow B, Pavlidis P (2005) Experimental comparison and cross-validation of the Affymetrix and Illumina gene expression analysis platforms. Nucleic Acids Res 33: 5914–5923. 16237126 10.1093/nar/gki890 PMC1258170

[37] Shi L, Reid LH, Jones WD, Shippy R, Warrington JA, et al (2006) The MicroArray Quality Control (MAQC) project shows inter- and intraplatform reproducibility of gene expression measurements. Nat Biotechnol 24: 1151–1161. 16964229 10.1038/nbt1239 PMC3272078

[38] Zhang Z, Gasser DL, Rappaport EF, Falk MJ (2010) Cross-platform expression microarray performance in a mouse model of mitochondrial disease therapy. Mol Genet Metab 99: 309–318. 19944634 10.1016/j.ymgme.2009.10.179 PMC2824080

[39] Rockett JC, Hellmann GM (2004) Confirming microarray data–is it really necessary? Genomics 83: 541–549. 15028276 10.1016/j.ygeno.2003.09.017 PMC7127508

