# Supplementary figures and images for: Differential Gene Expression at the Maternal-Fetal Interface in Preeclampsia Is Influenced by Gestational Age
Source: PLoS One. 2013 Jul 31;8(7):e69848. doi: 10.1371/journal.pone.0069848 (PMC3729459; doi:10.1371/journal.pone.0069848)

212765\_at

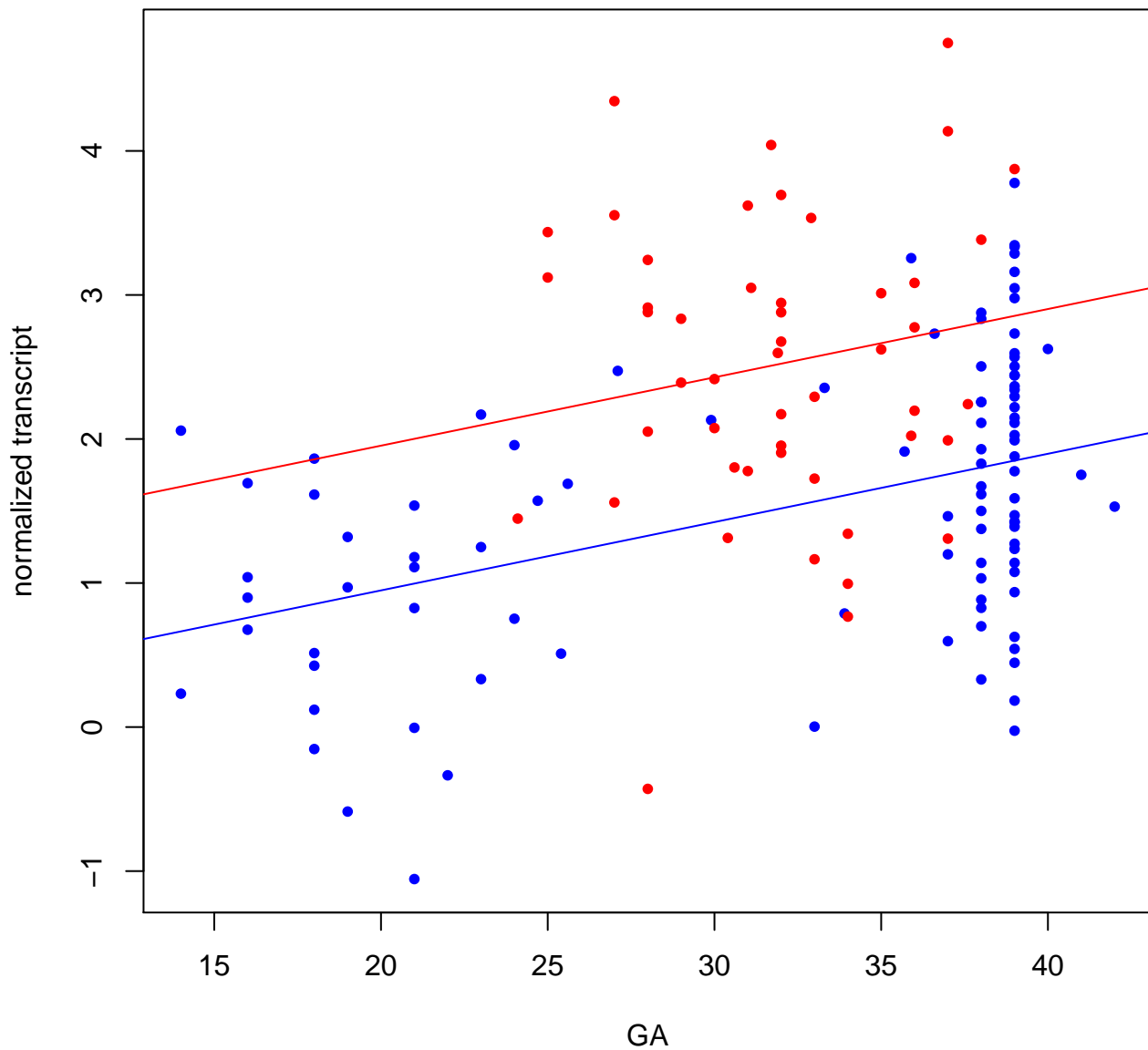

212763\_at

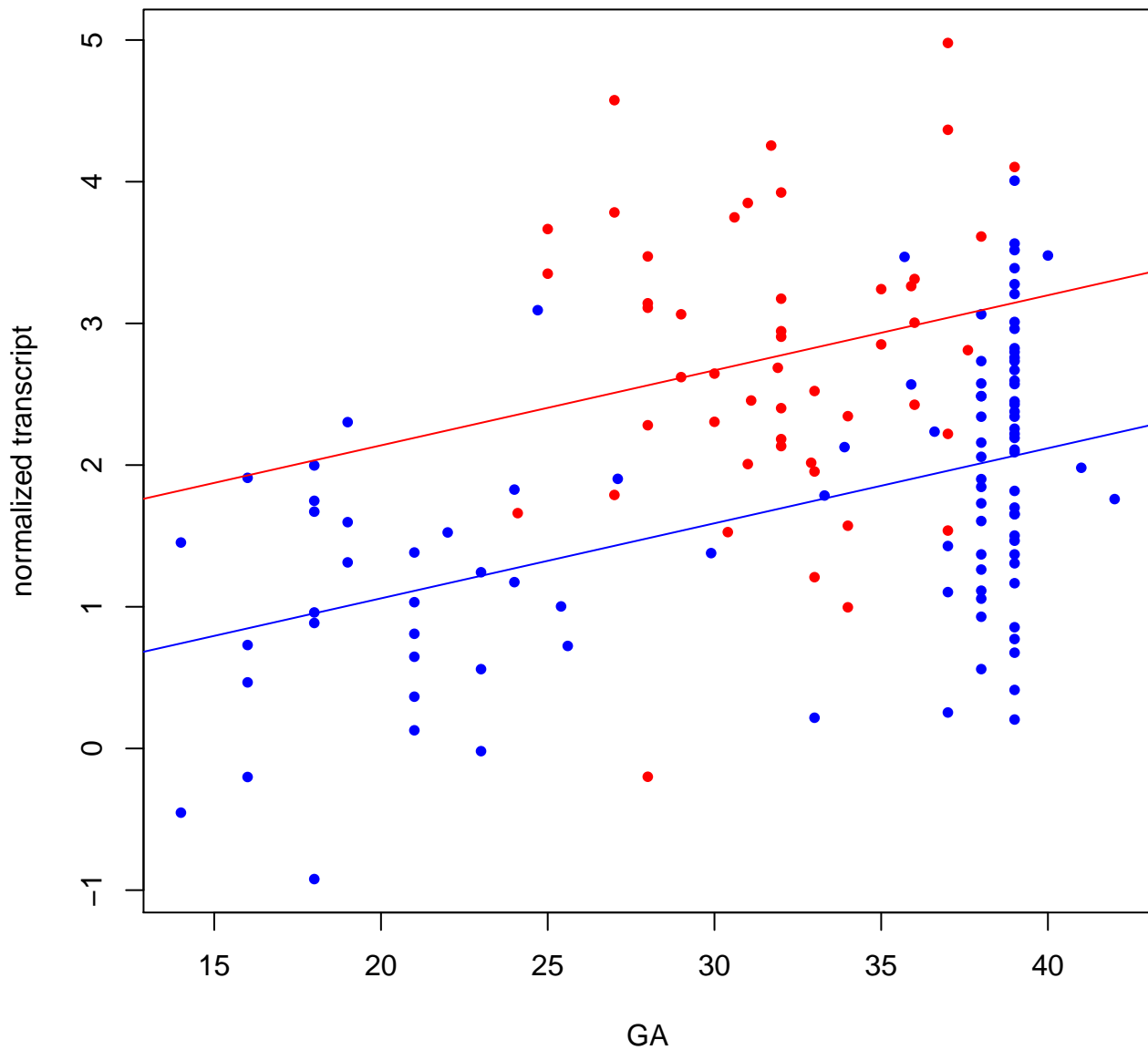

209955\_s\_at

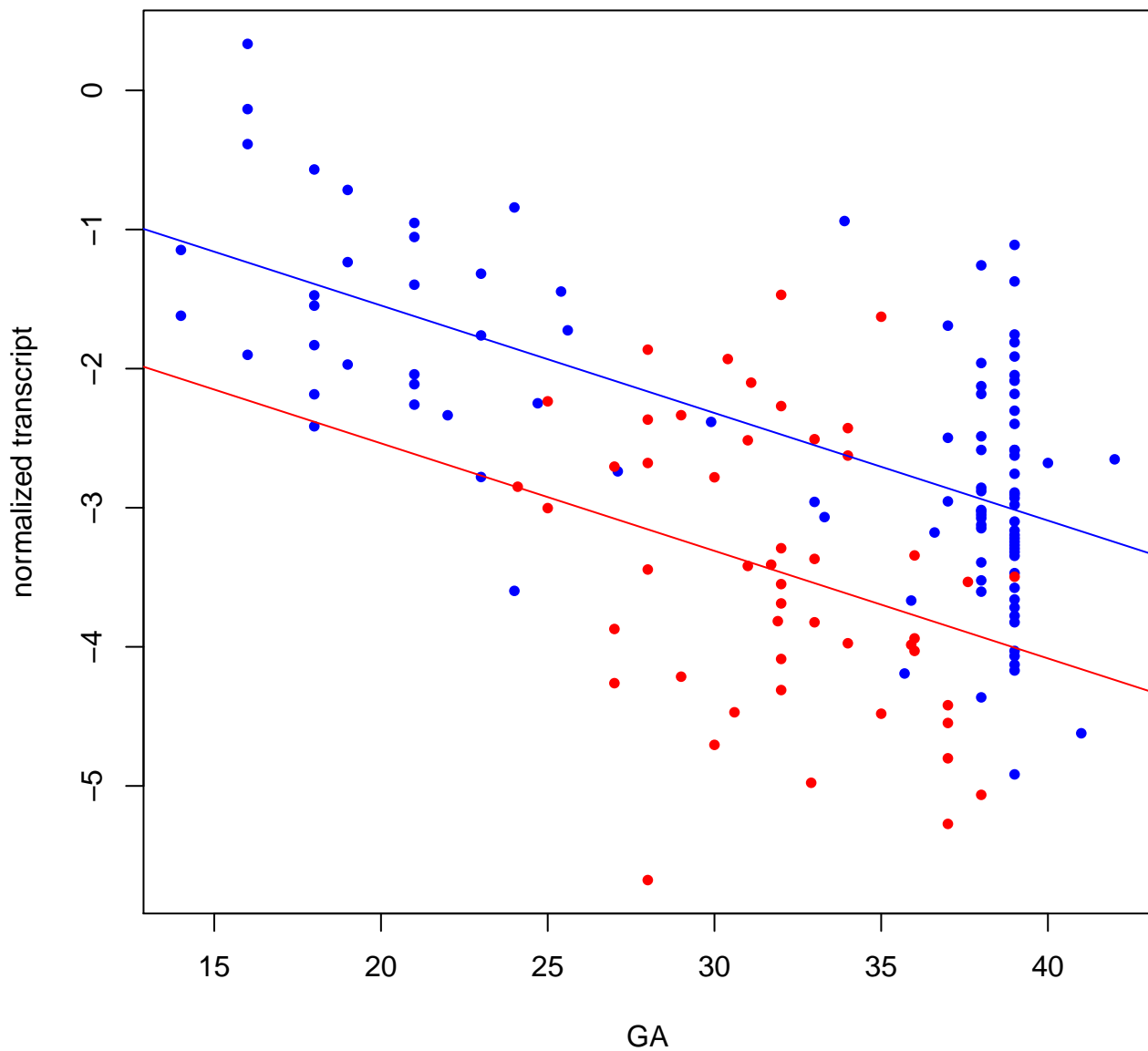

209496\_at

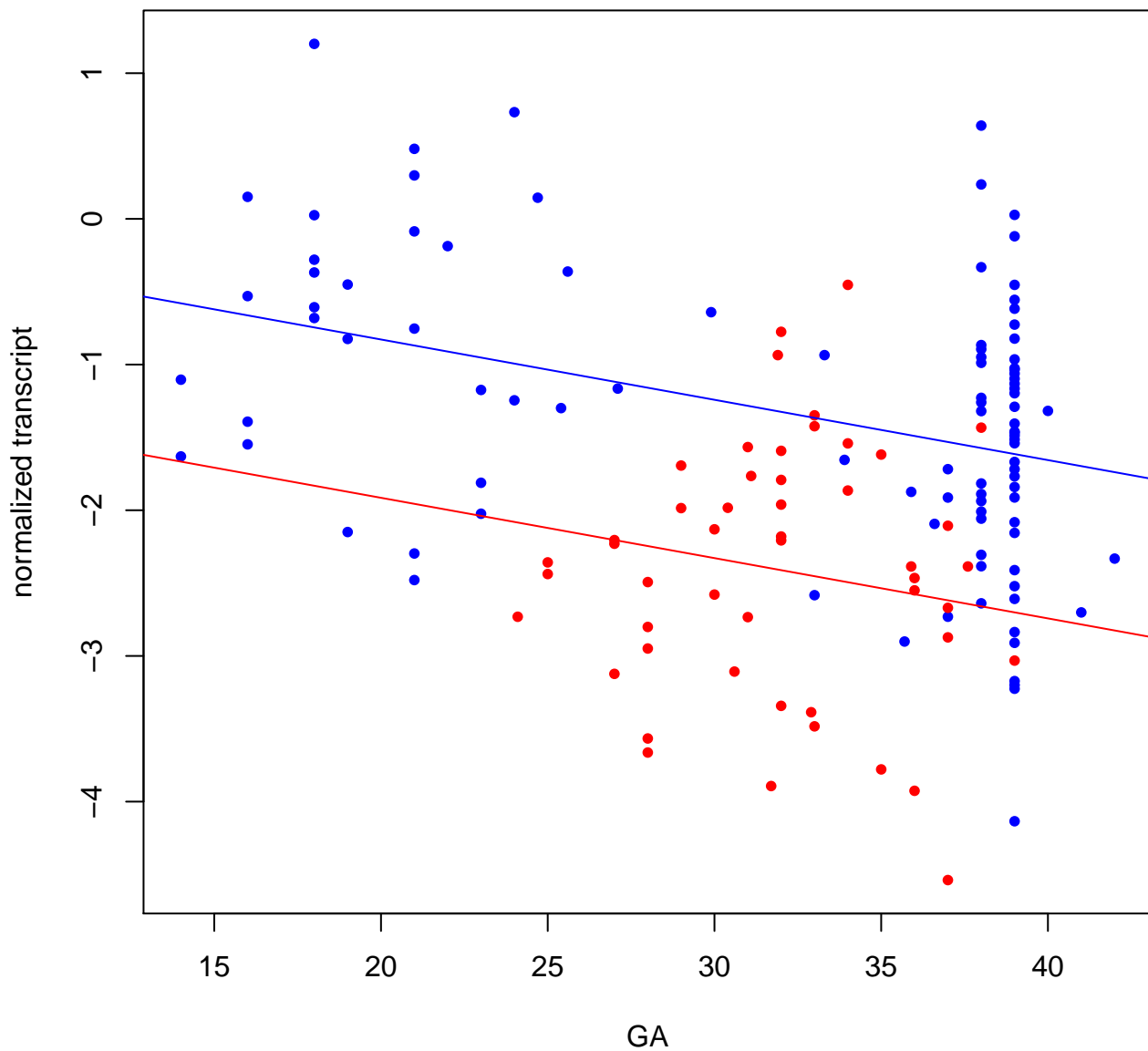

201197\_at.1

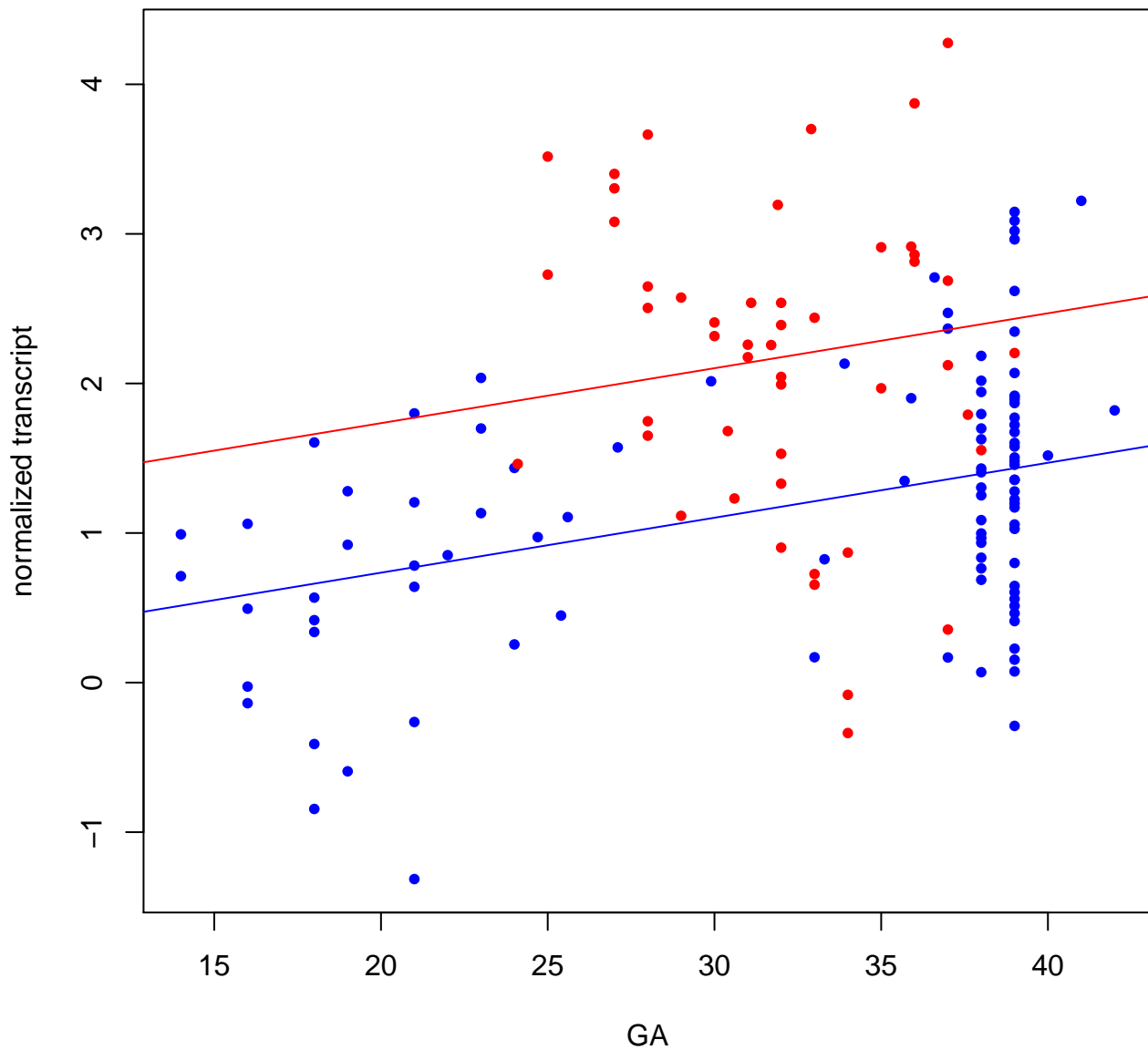

201196\_s\_at.1

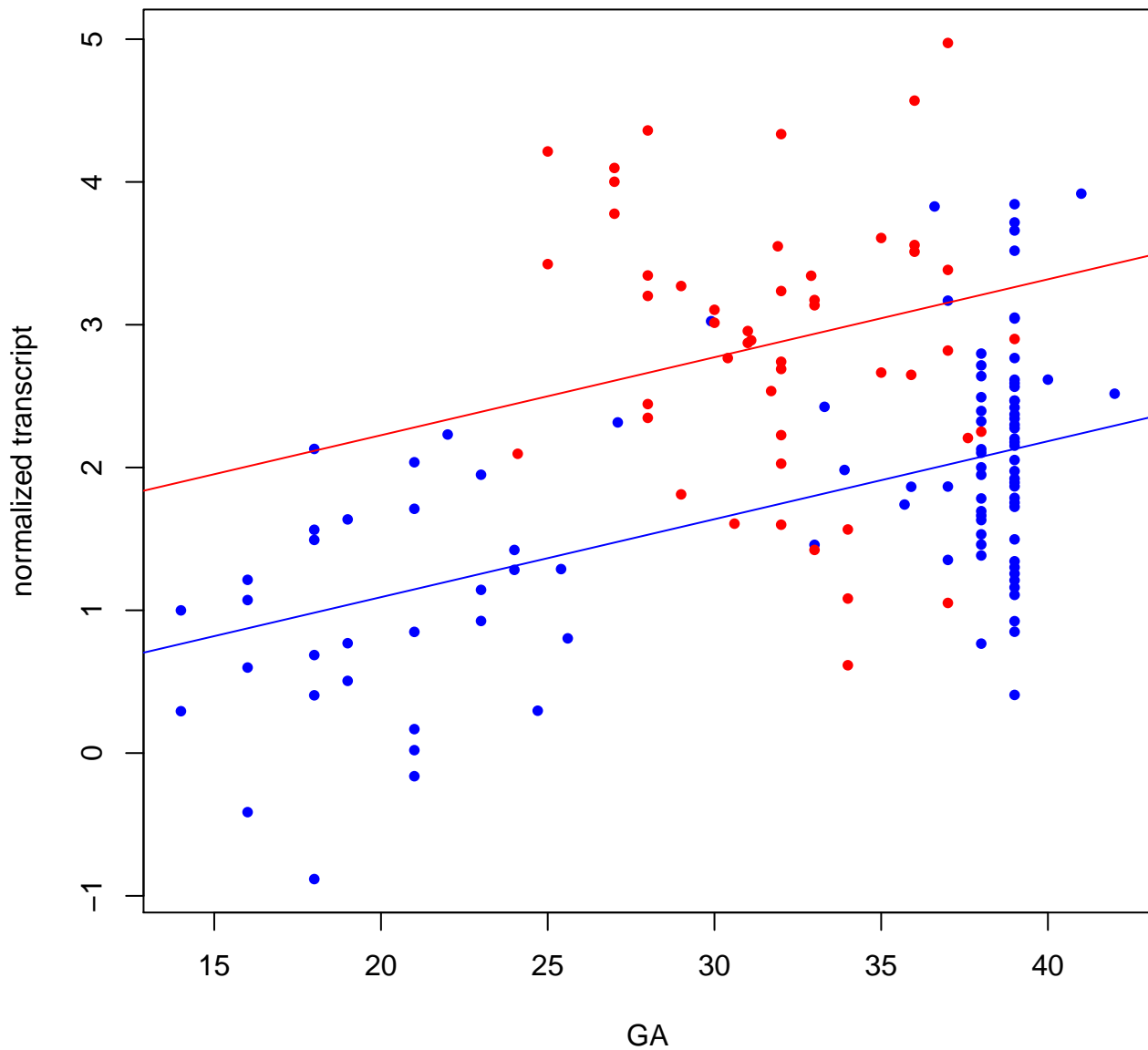

213001\_at

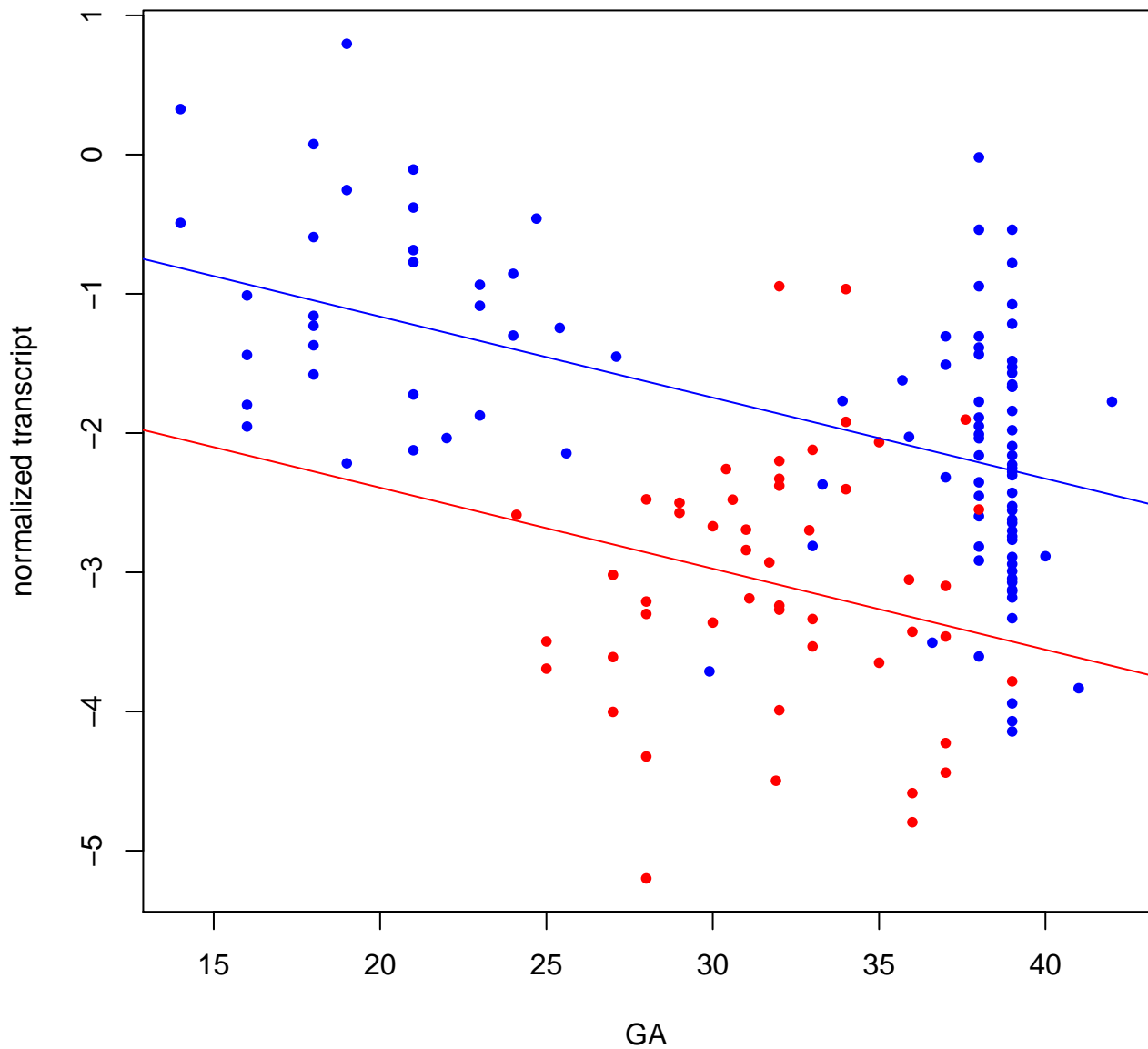

213004\_at

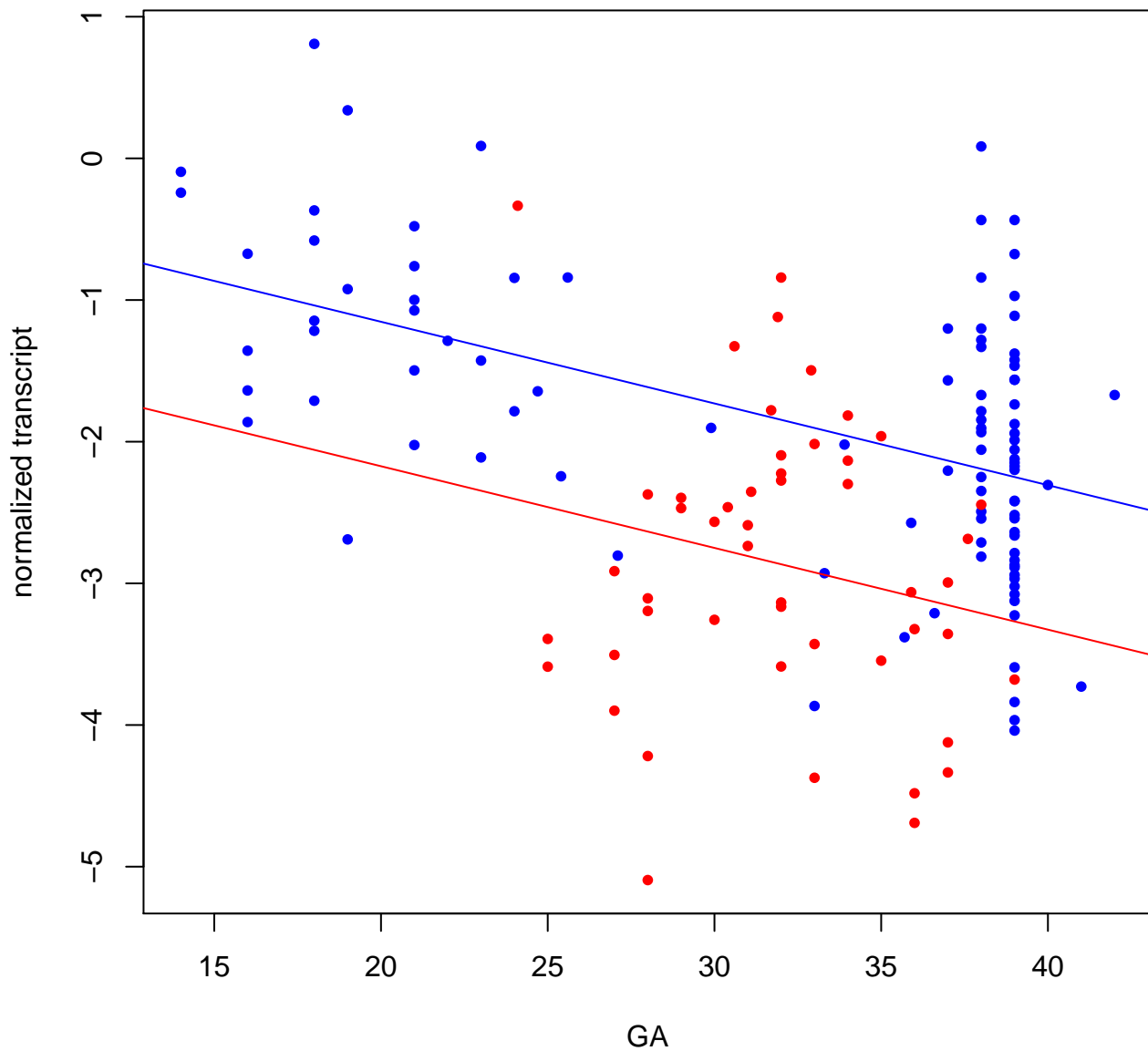

**206363\_at**

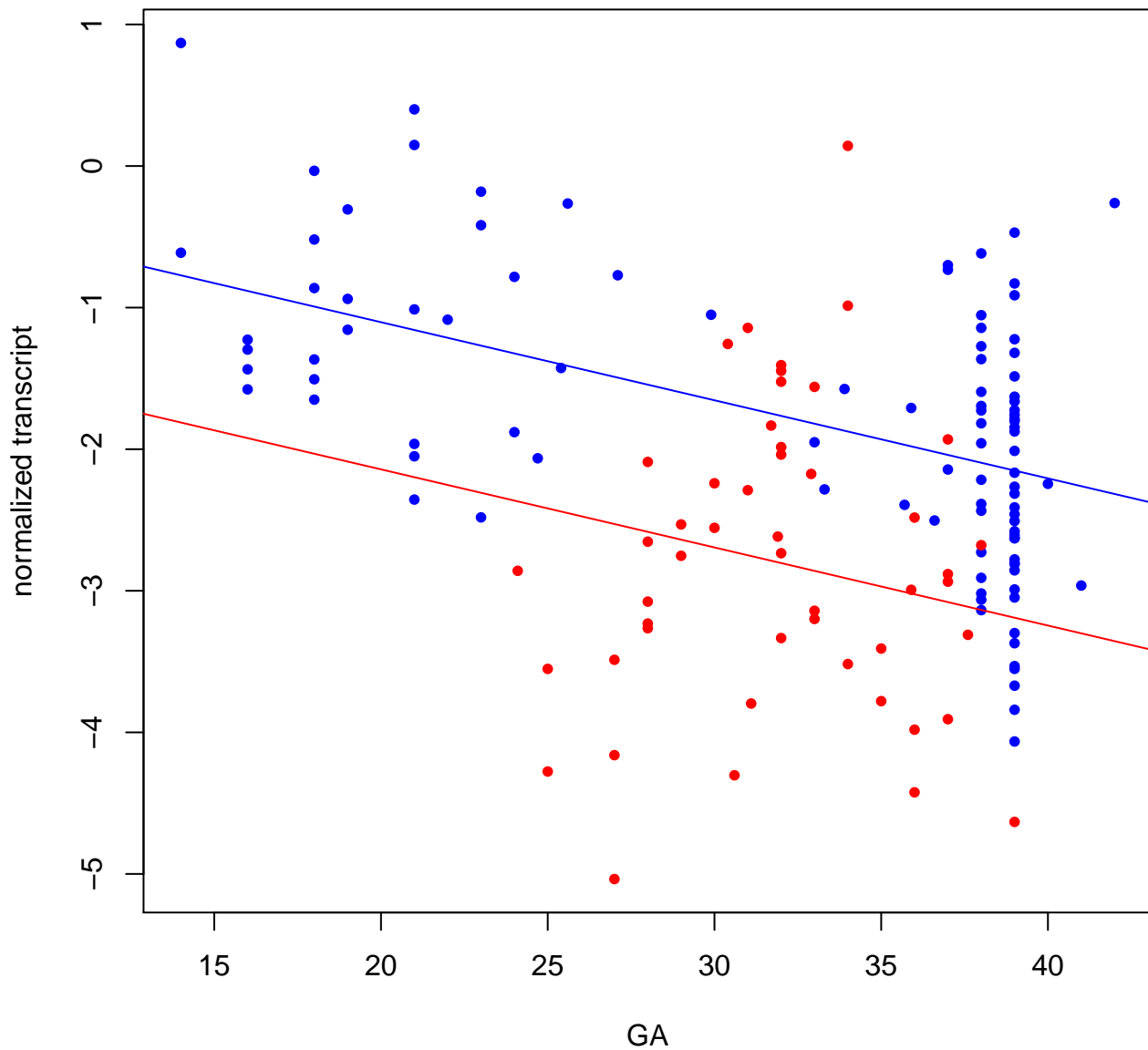

205629\_s\_at

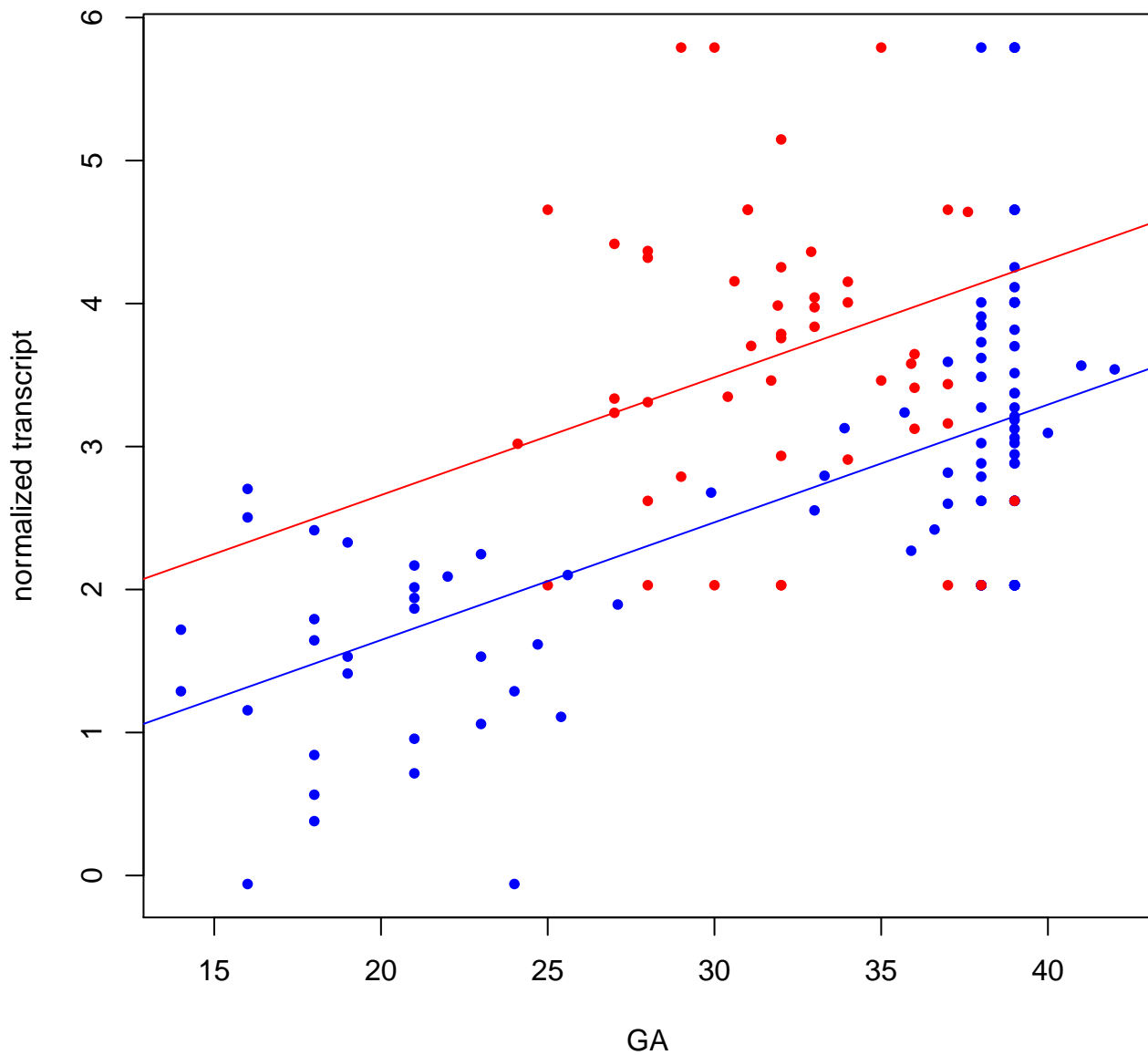

204955\_at

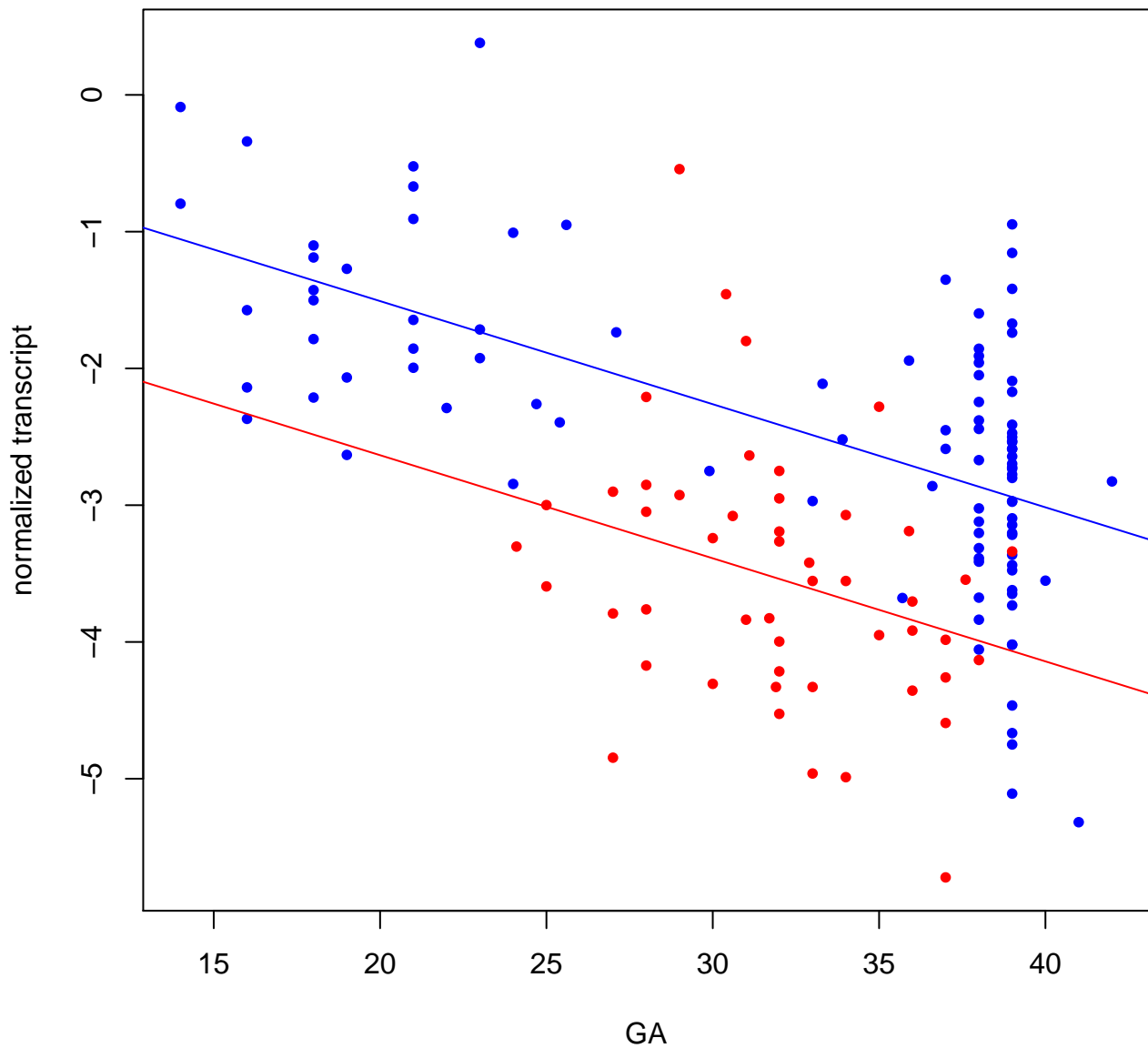

# 210139\_s\_at

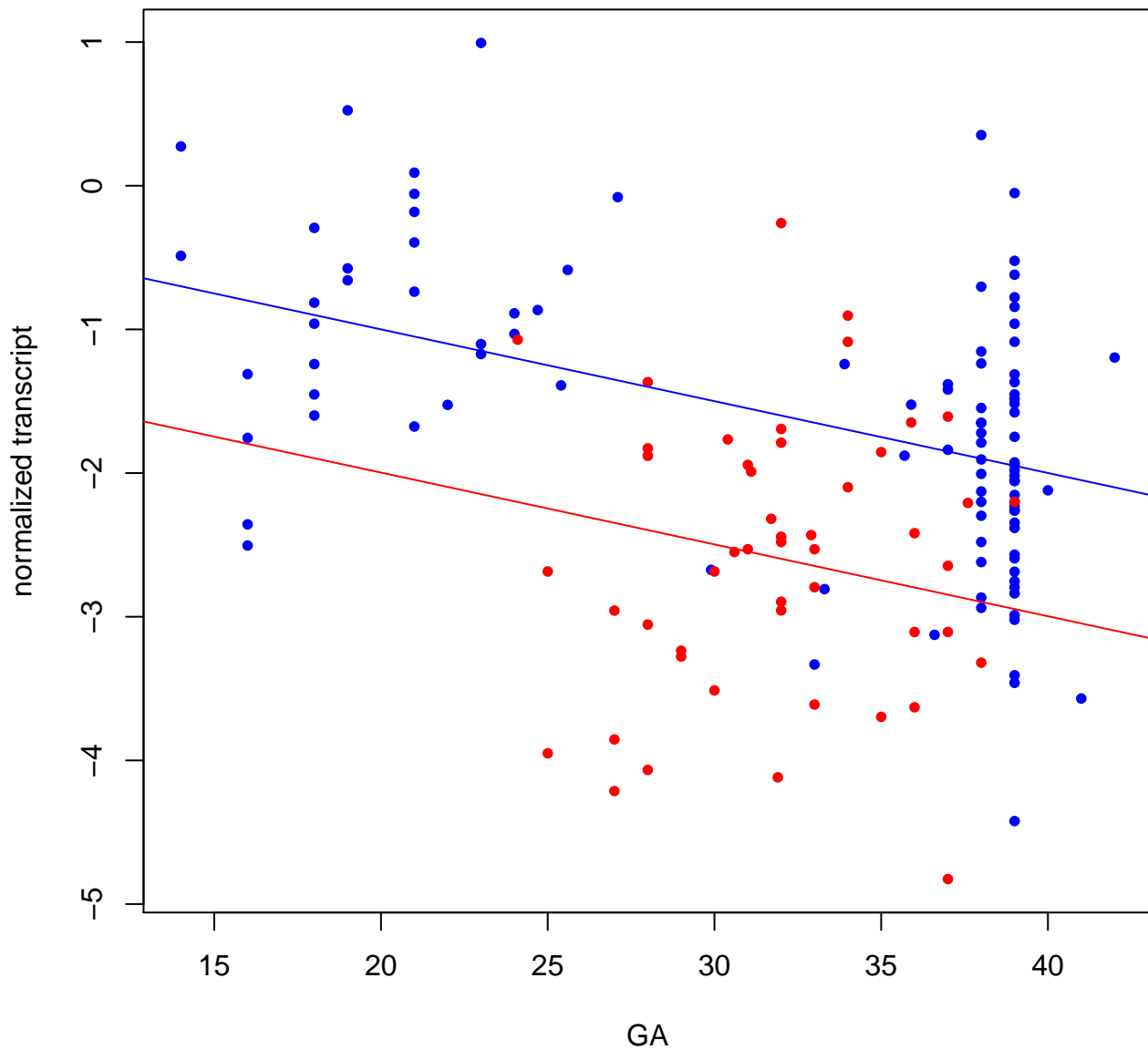

# 207279\_s\_at

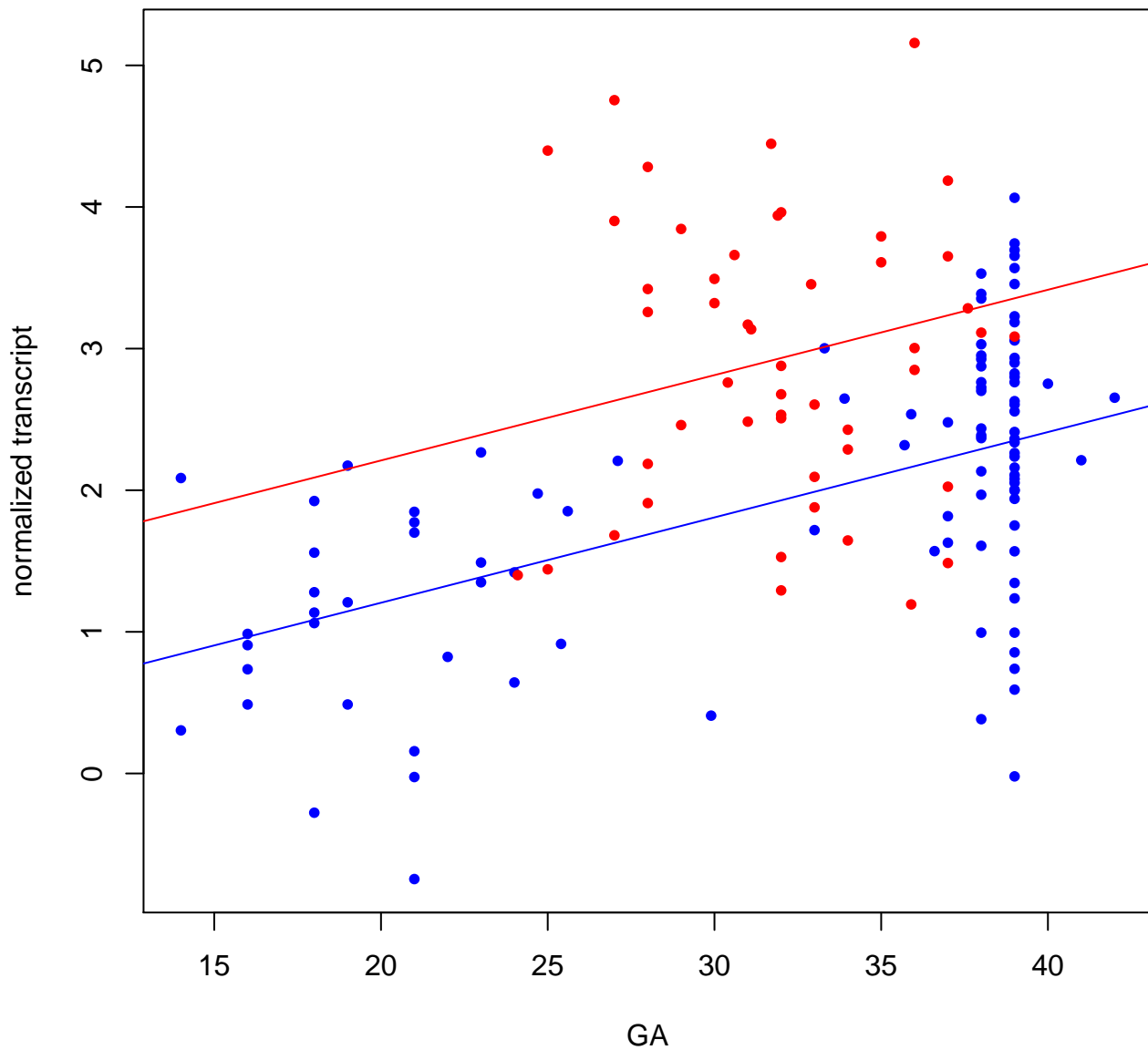

# 219869\_s\_at

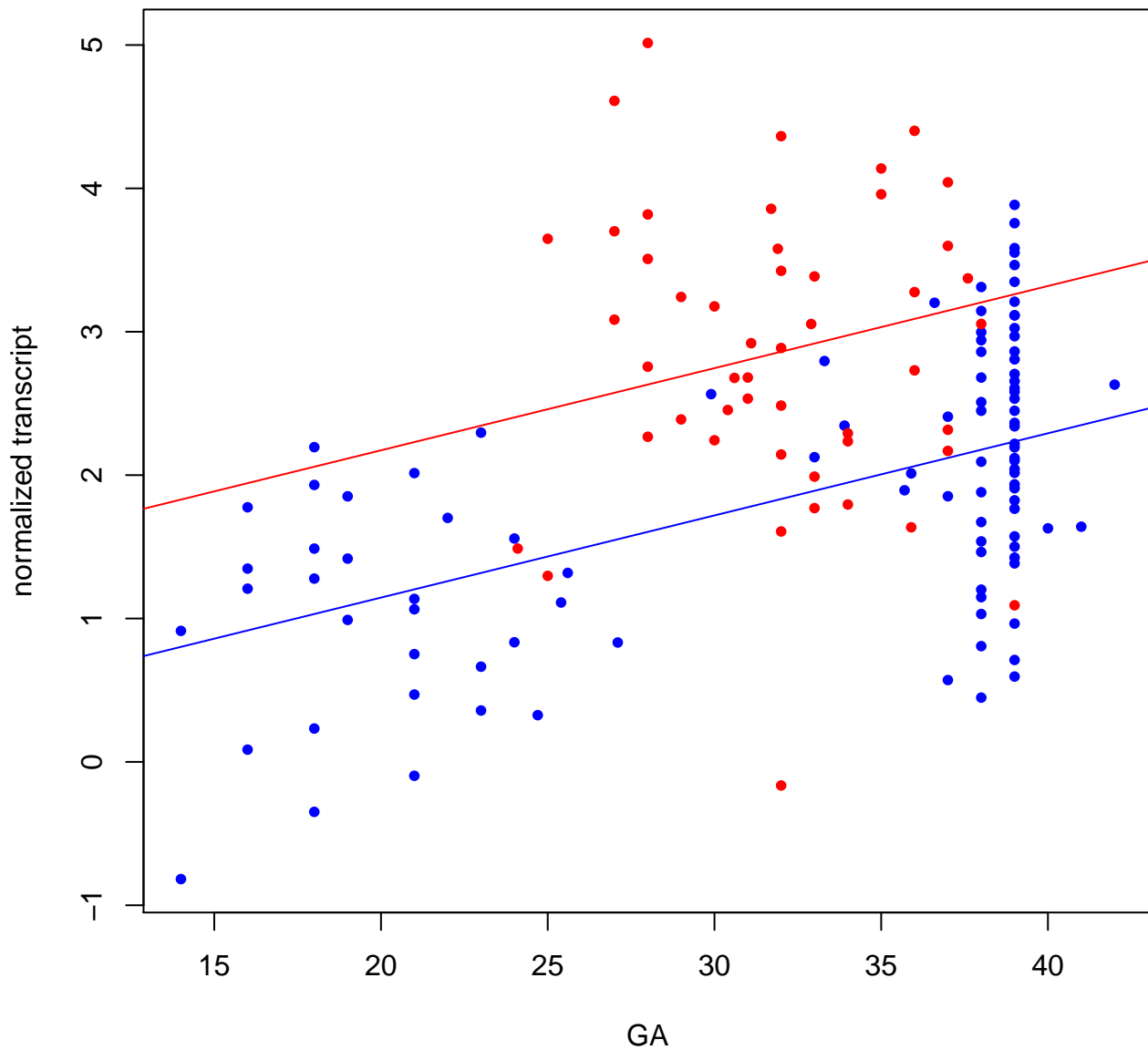

201462\_at

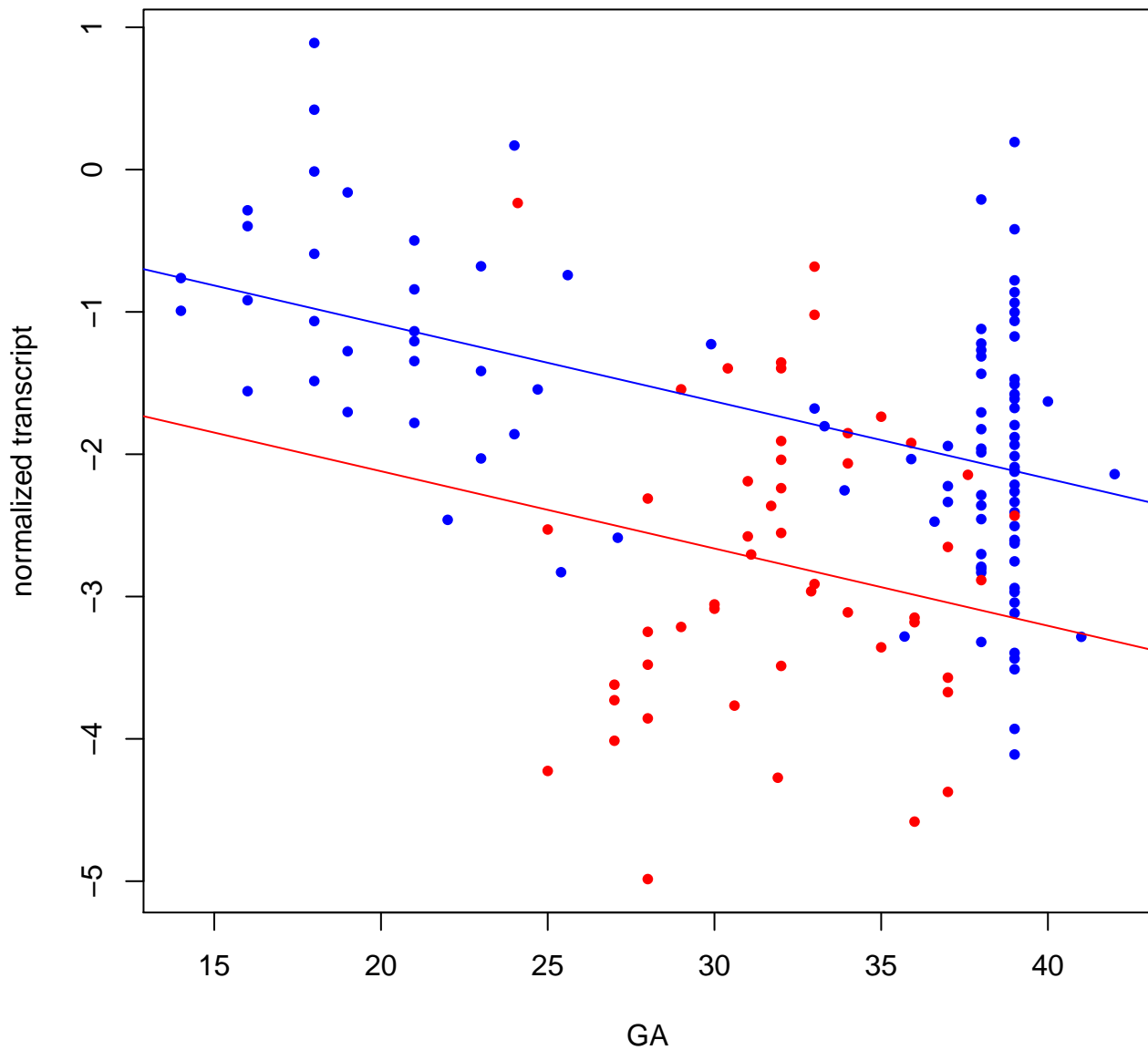

# 221879\_at.1

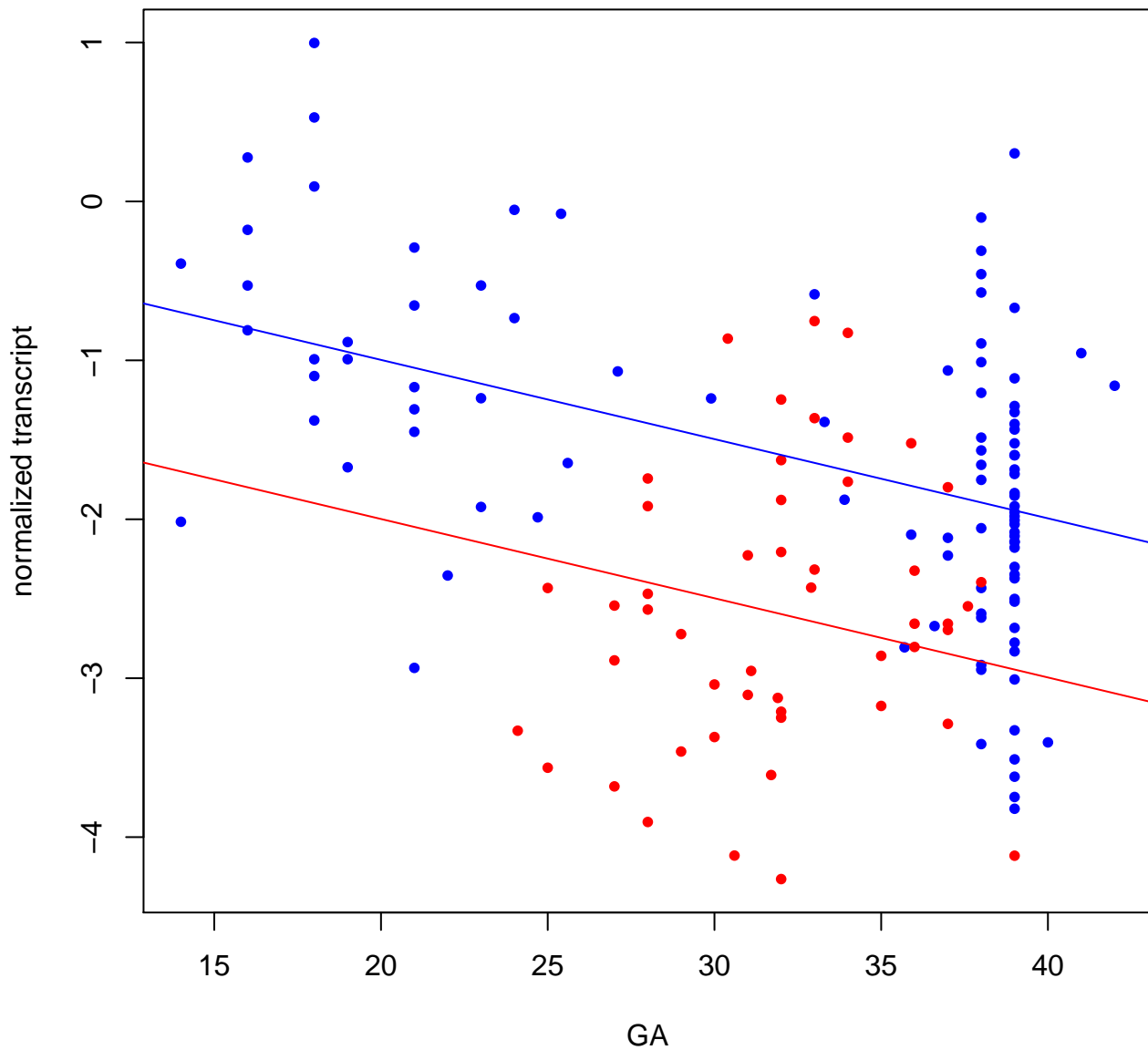

201984\_s\_at

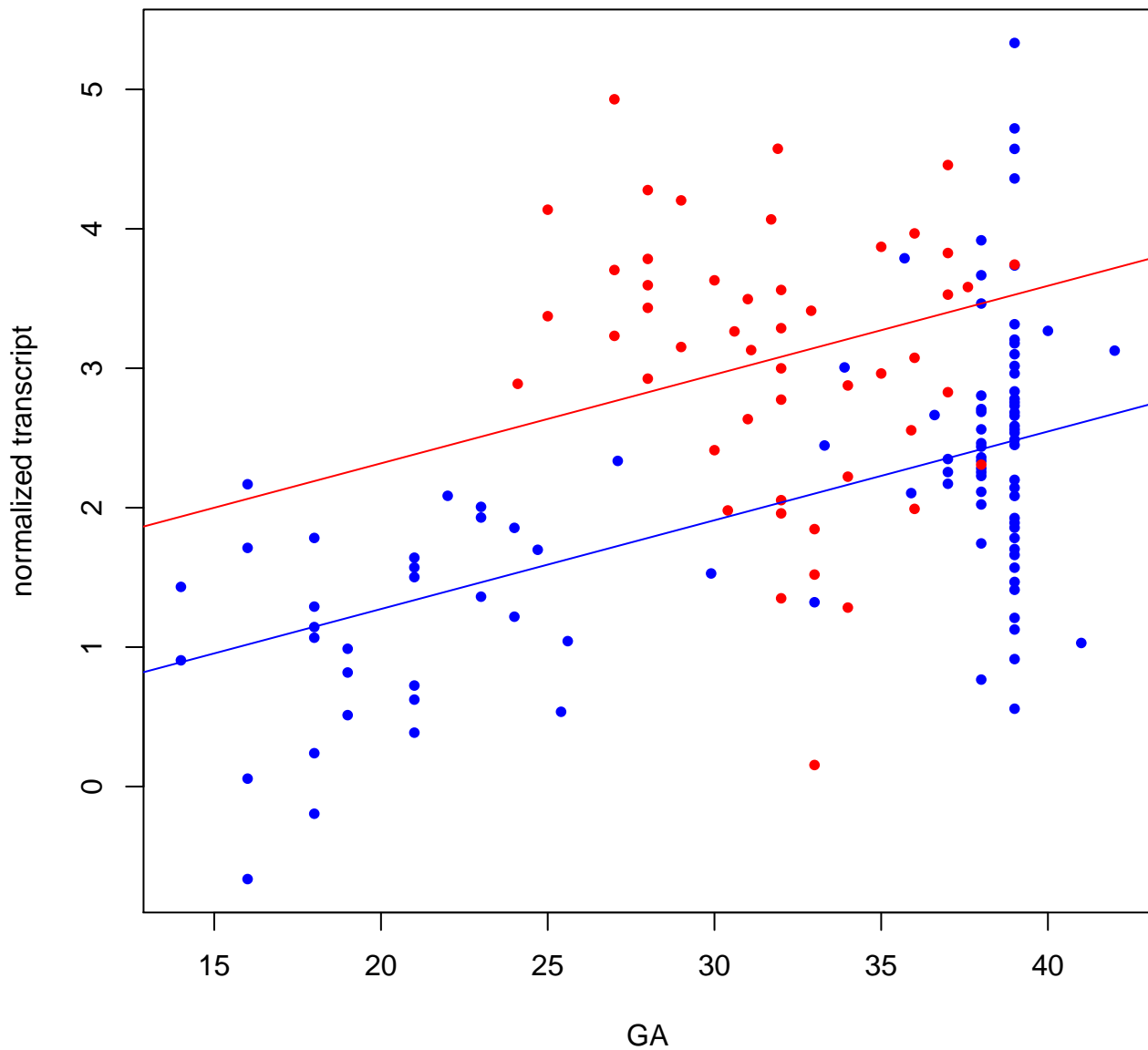

213428\_s\_at

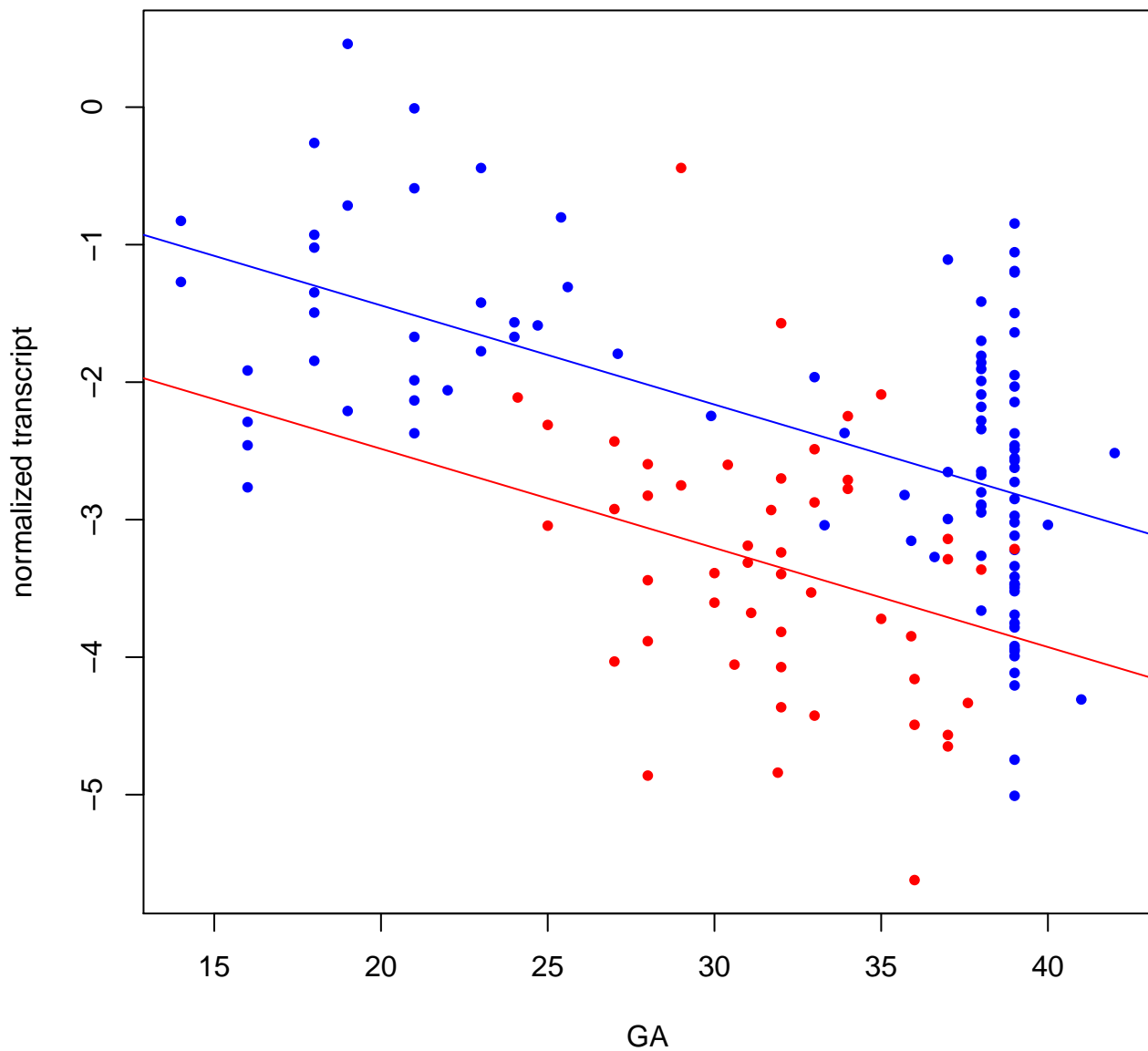

212325\_at

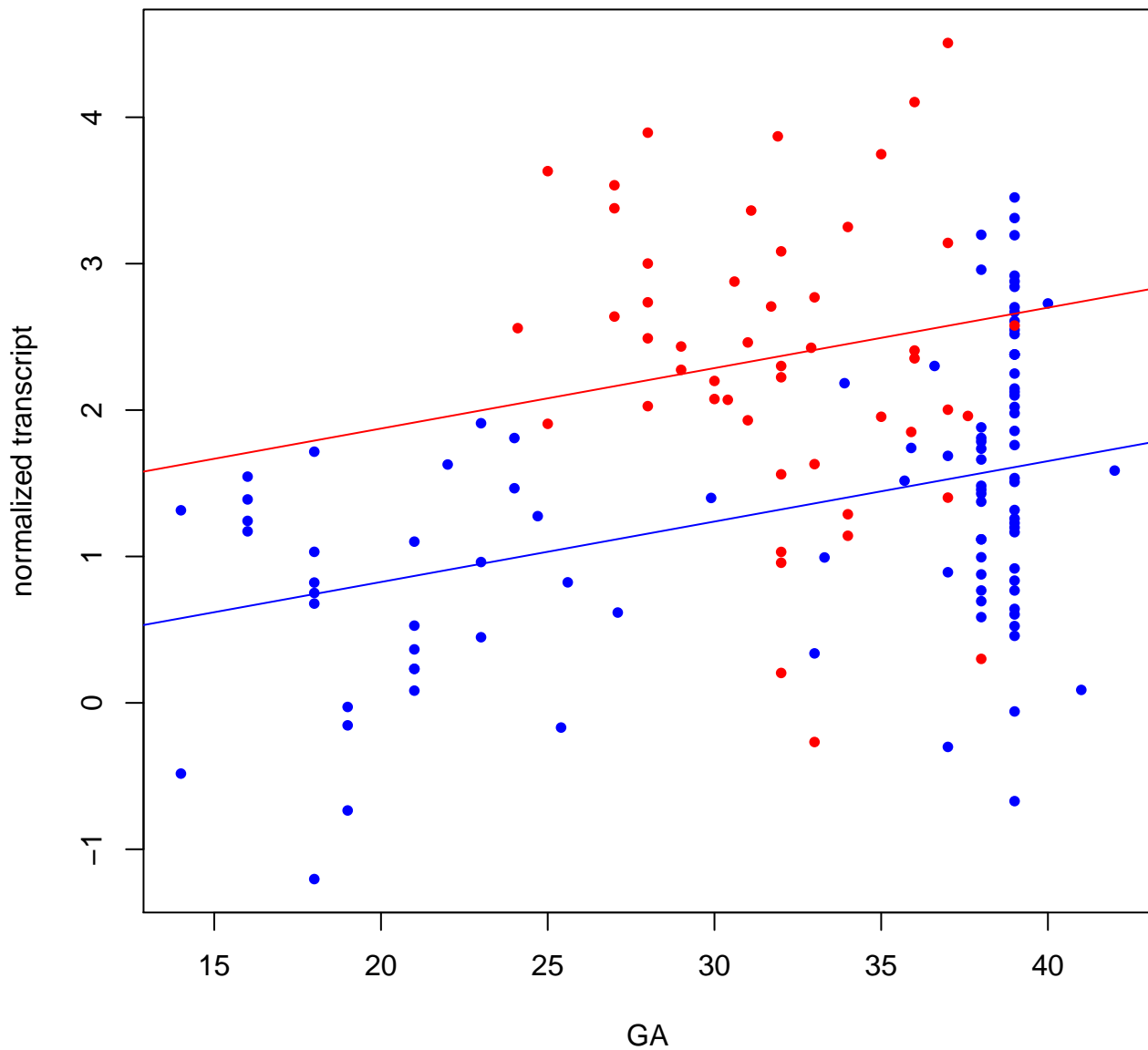

212328\_at

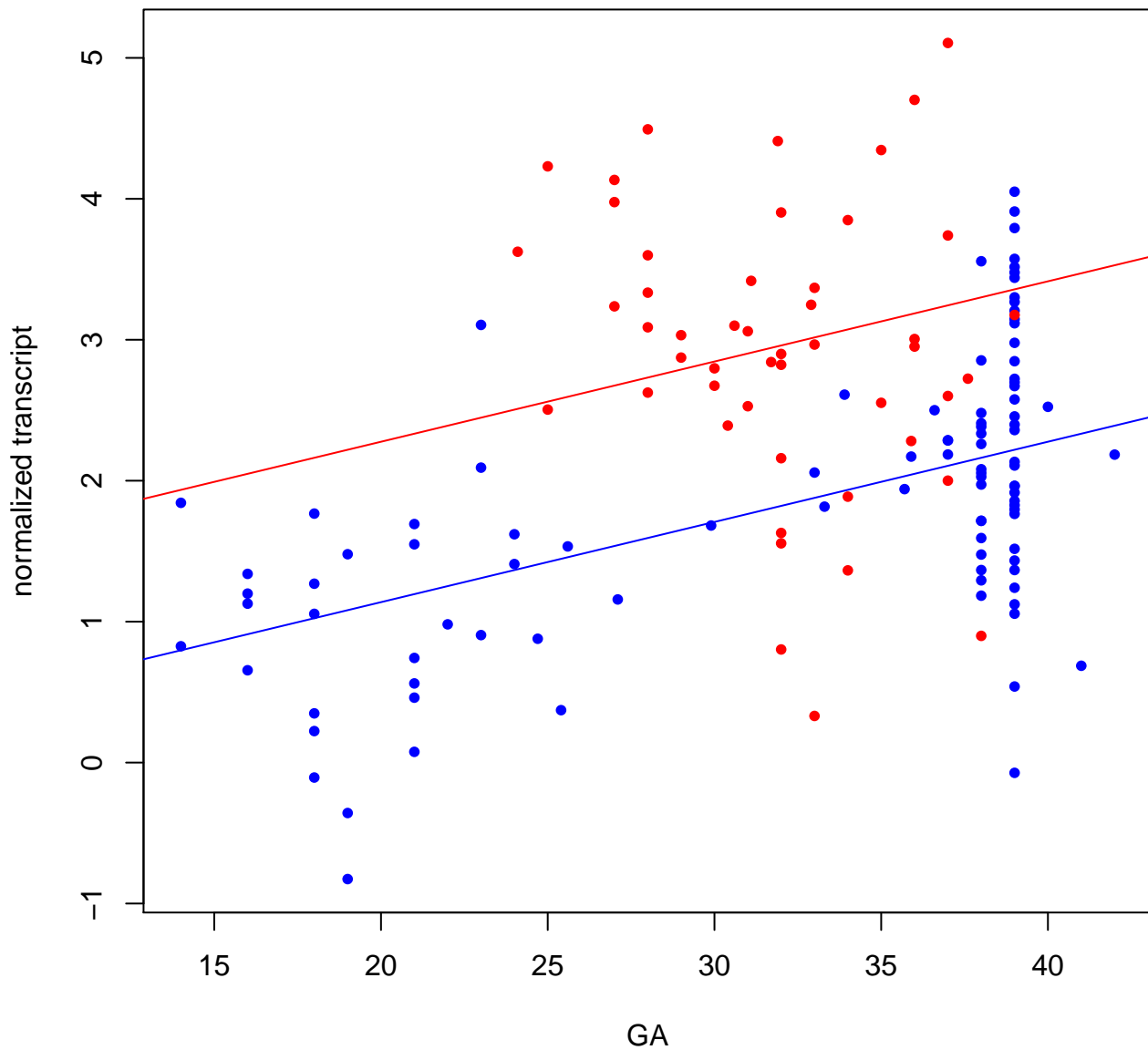

212327\_at

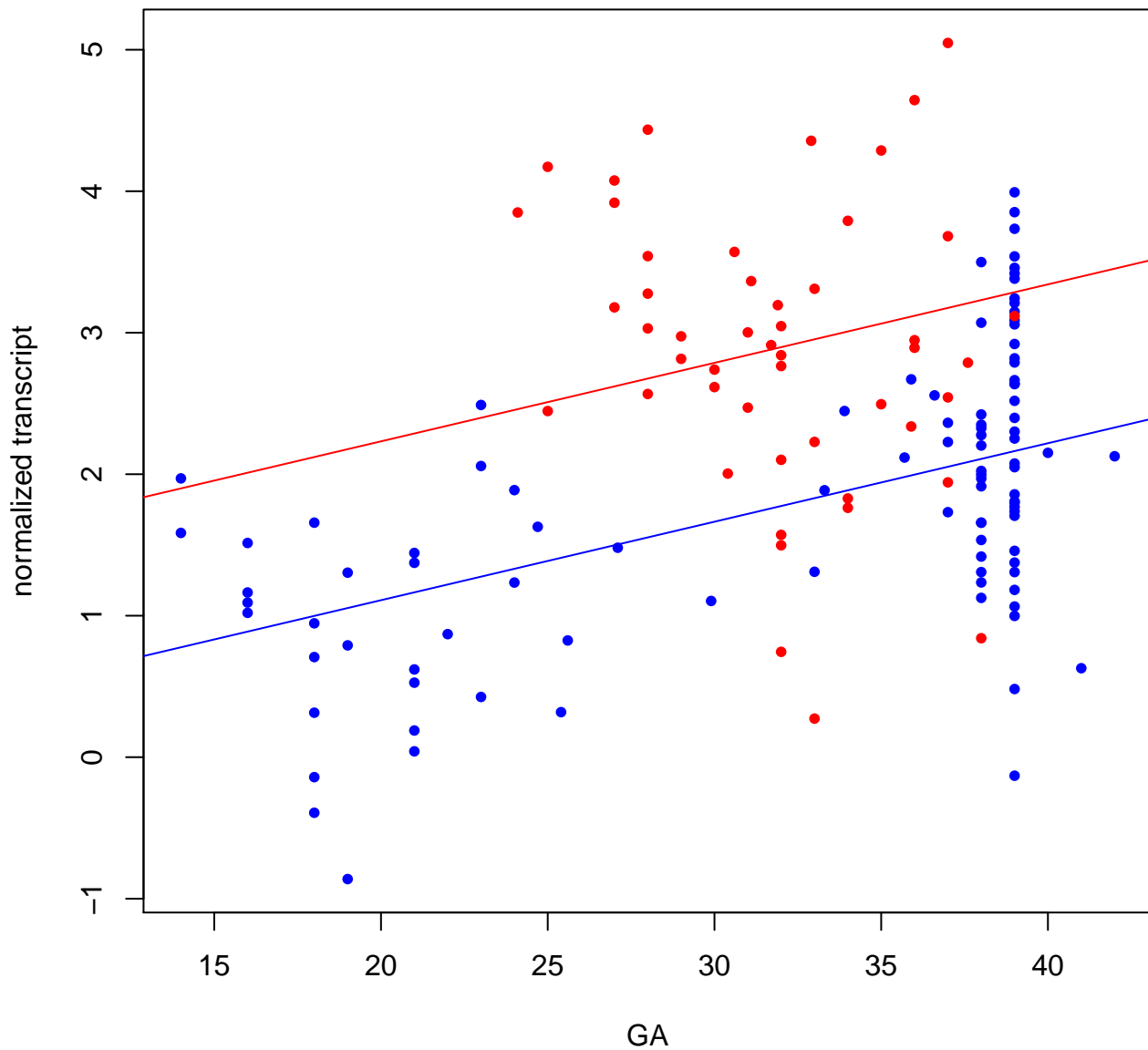

202283\_at

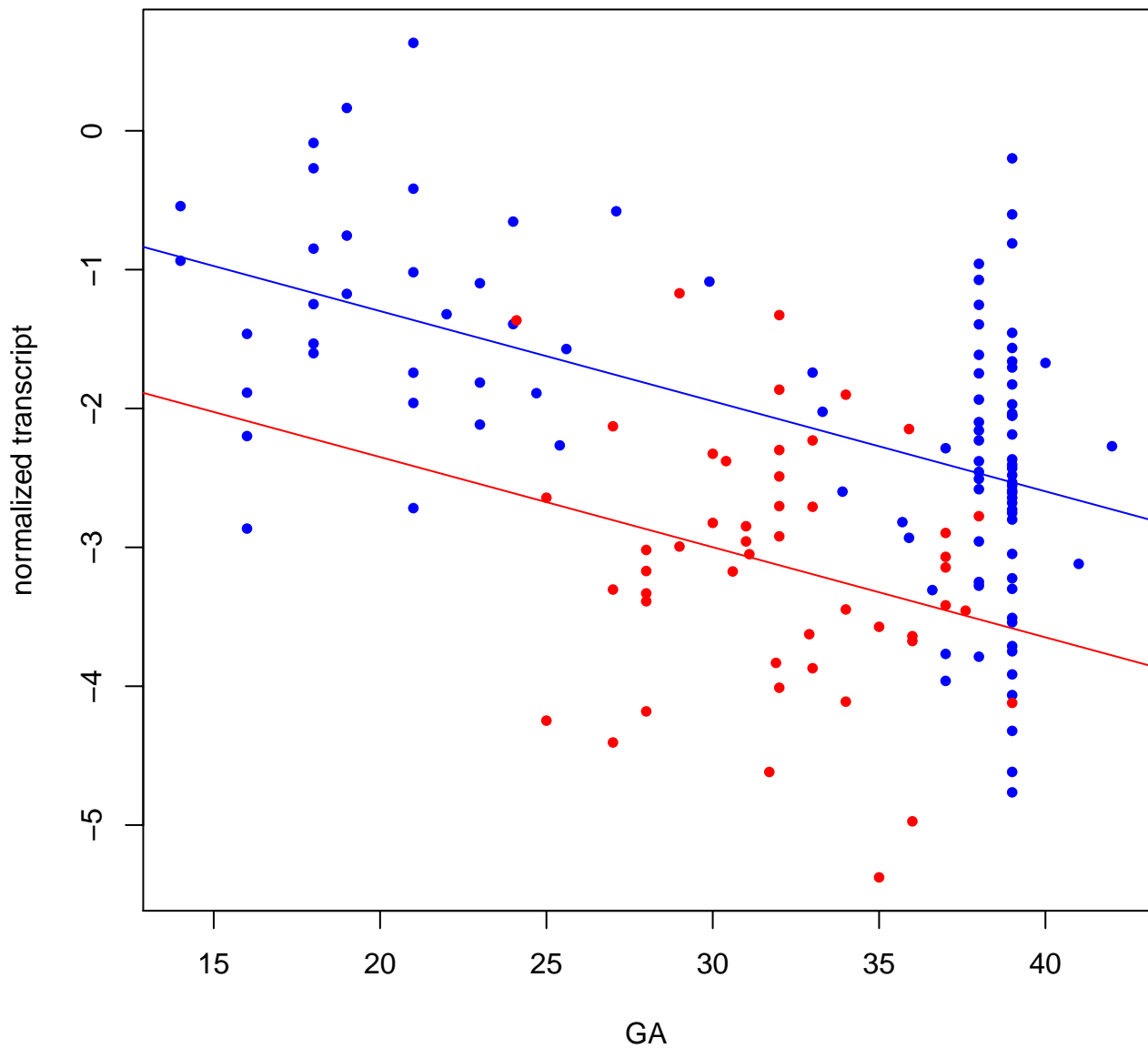

232139\_s\_at

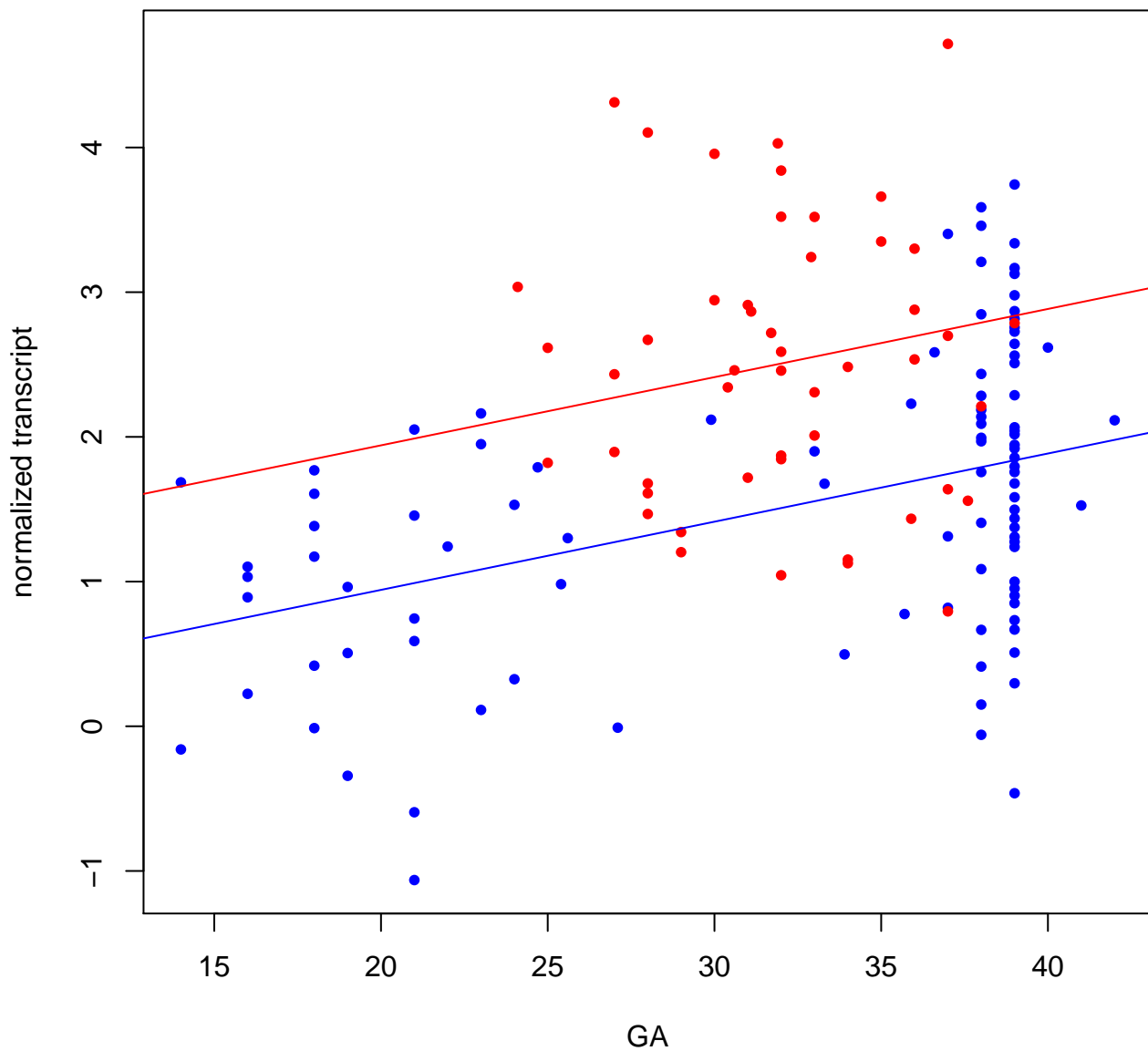

238828\_at

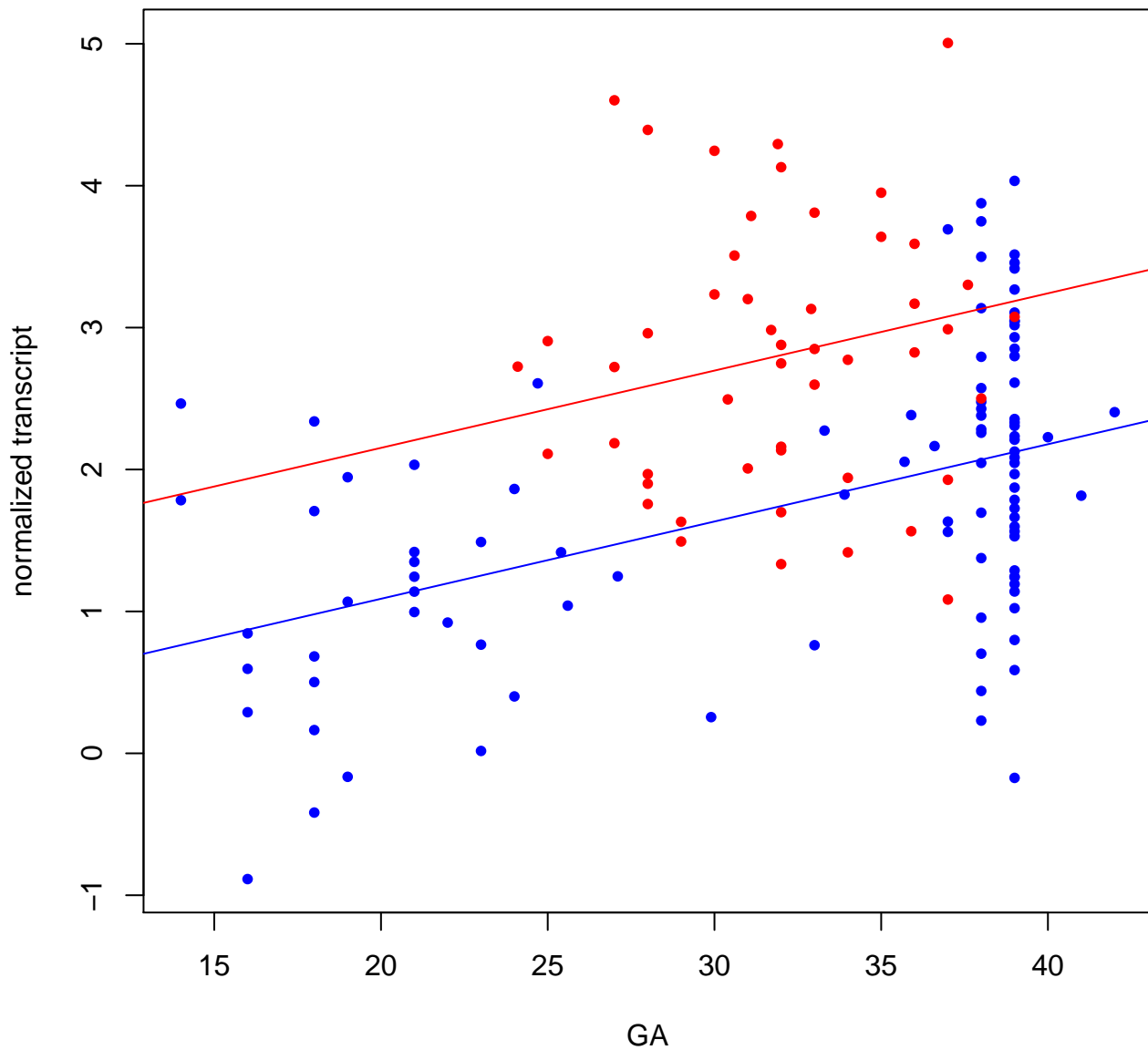

227419\_x\_at

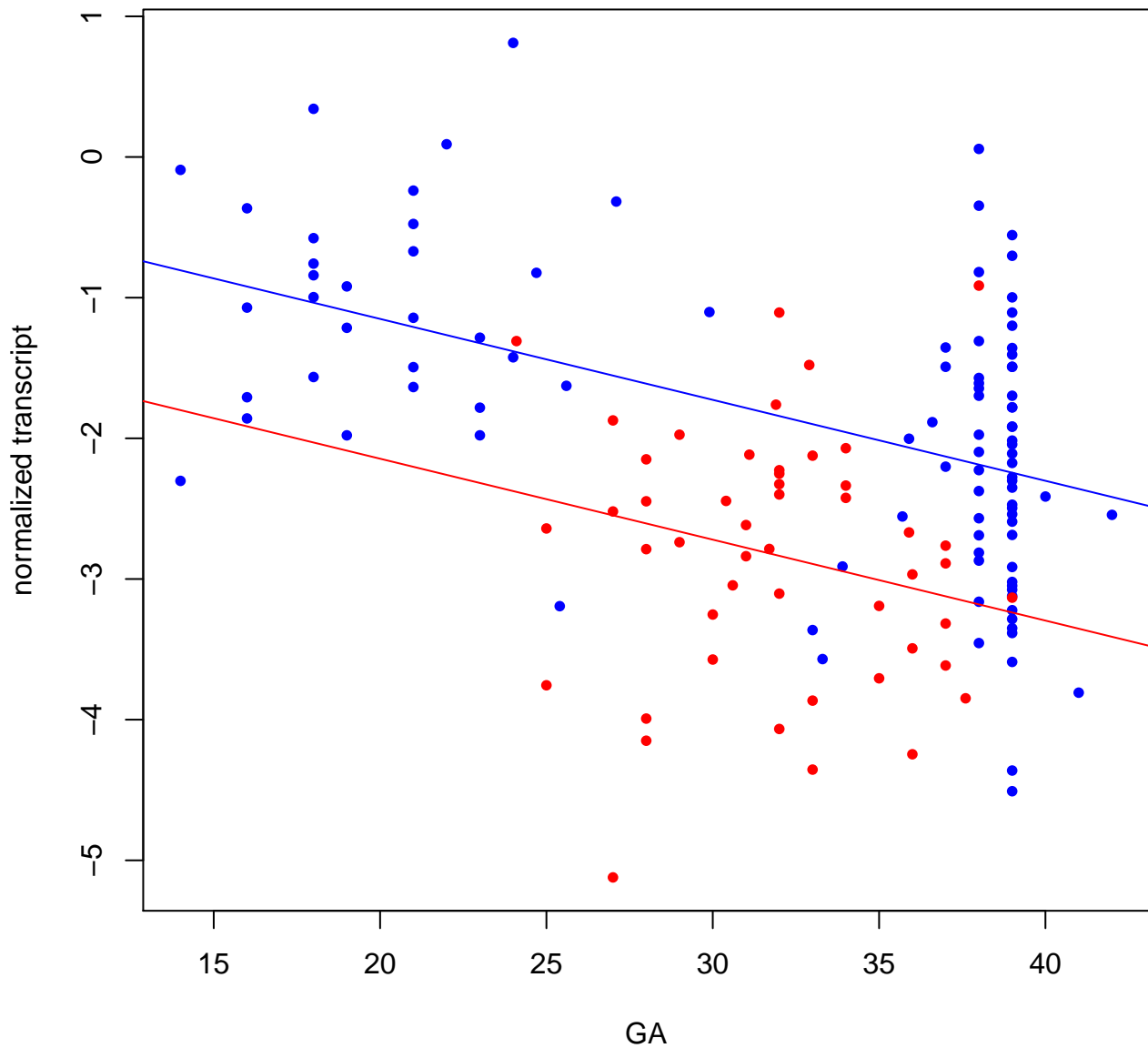

224799\_at

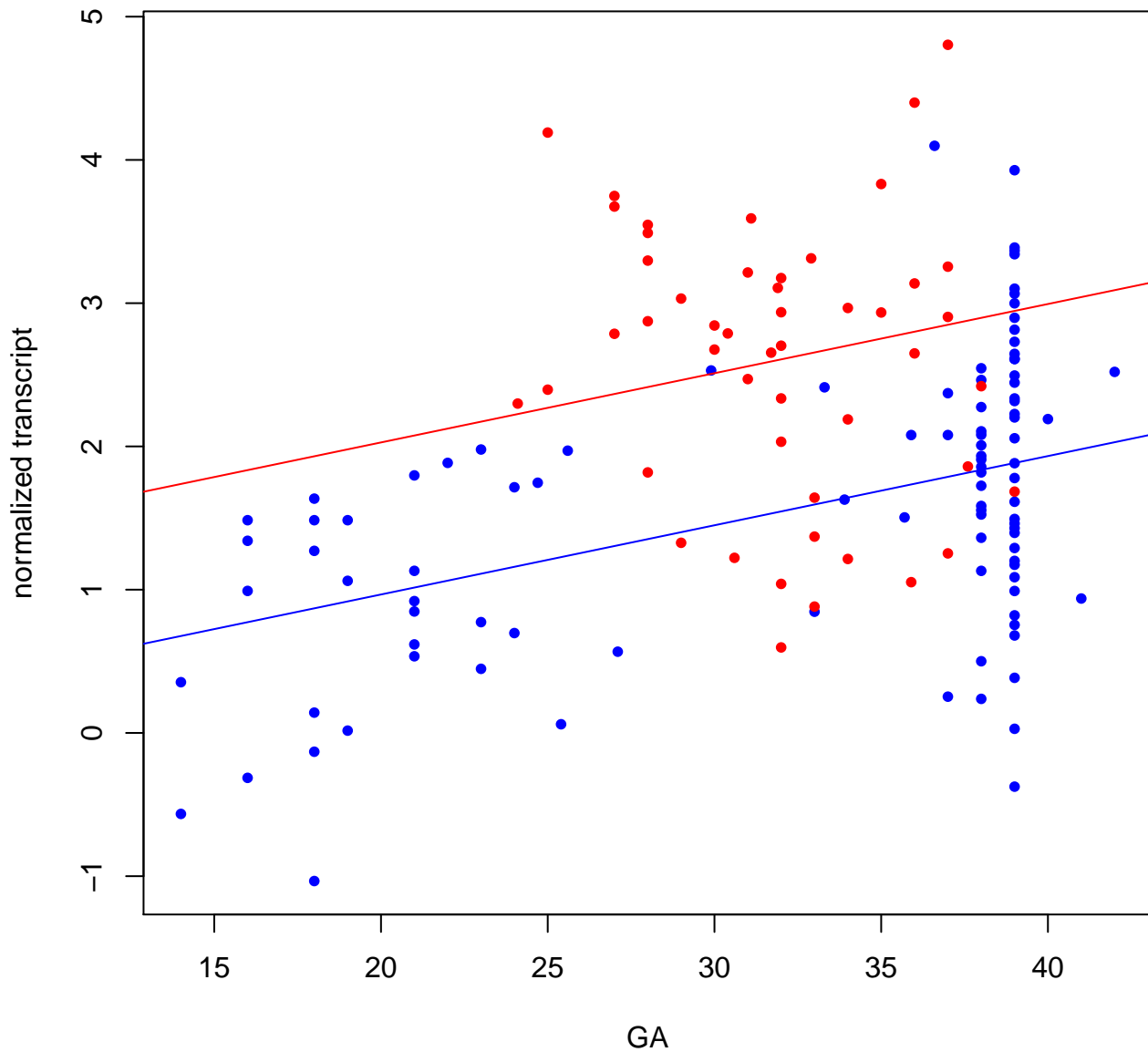

224801\_at

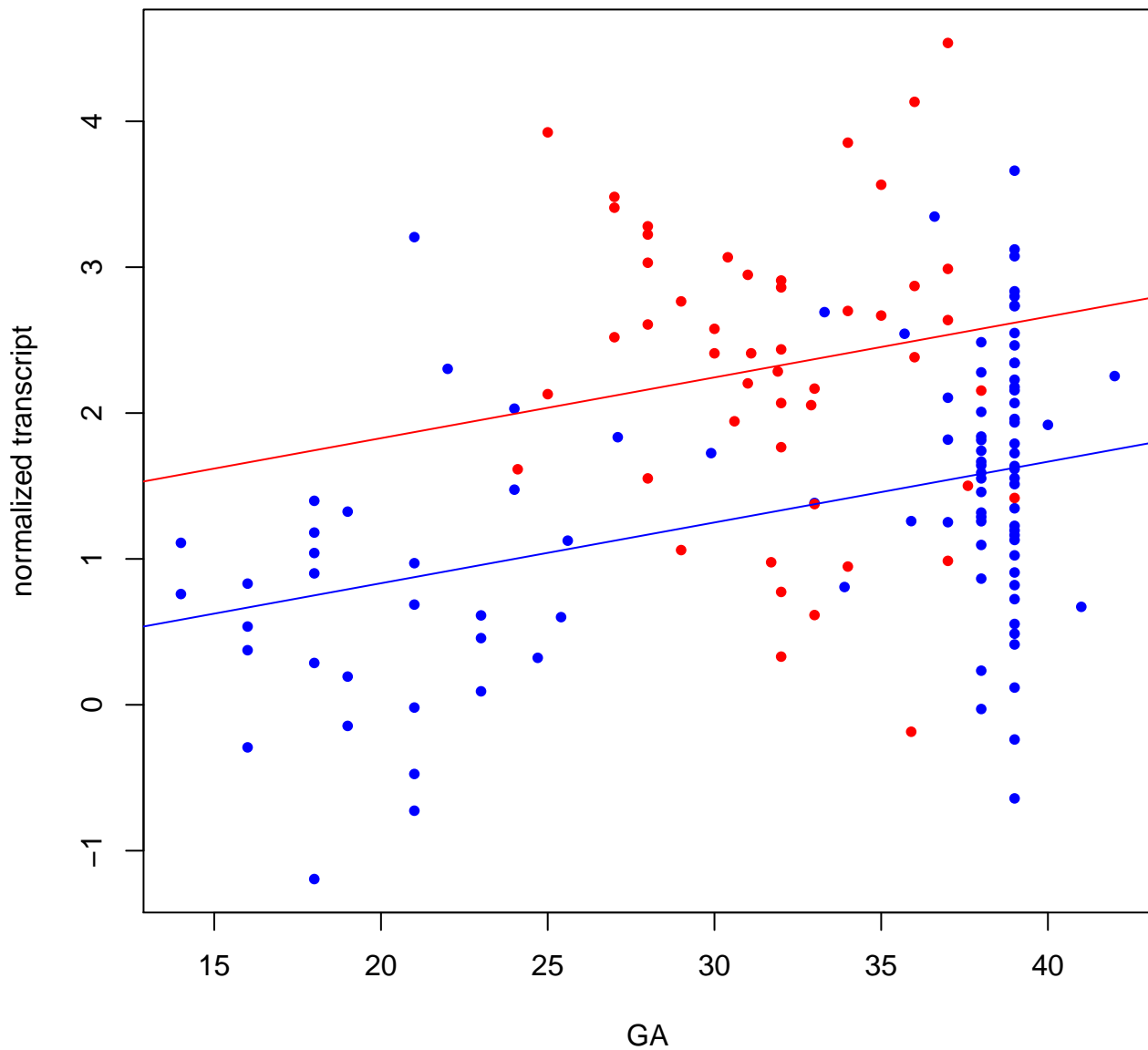

226769\_at

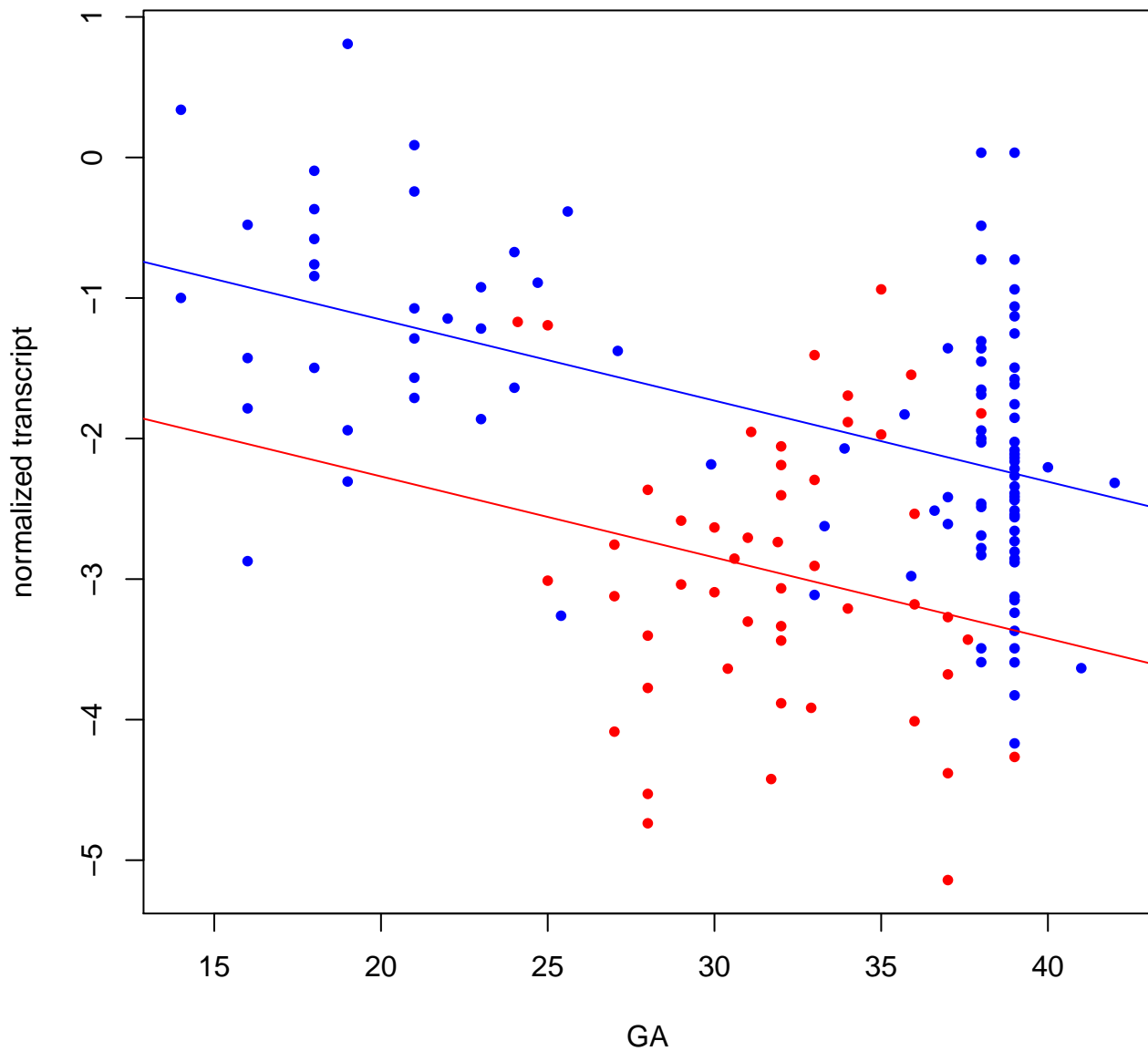

224836\_at

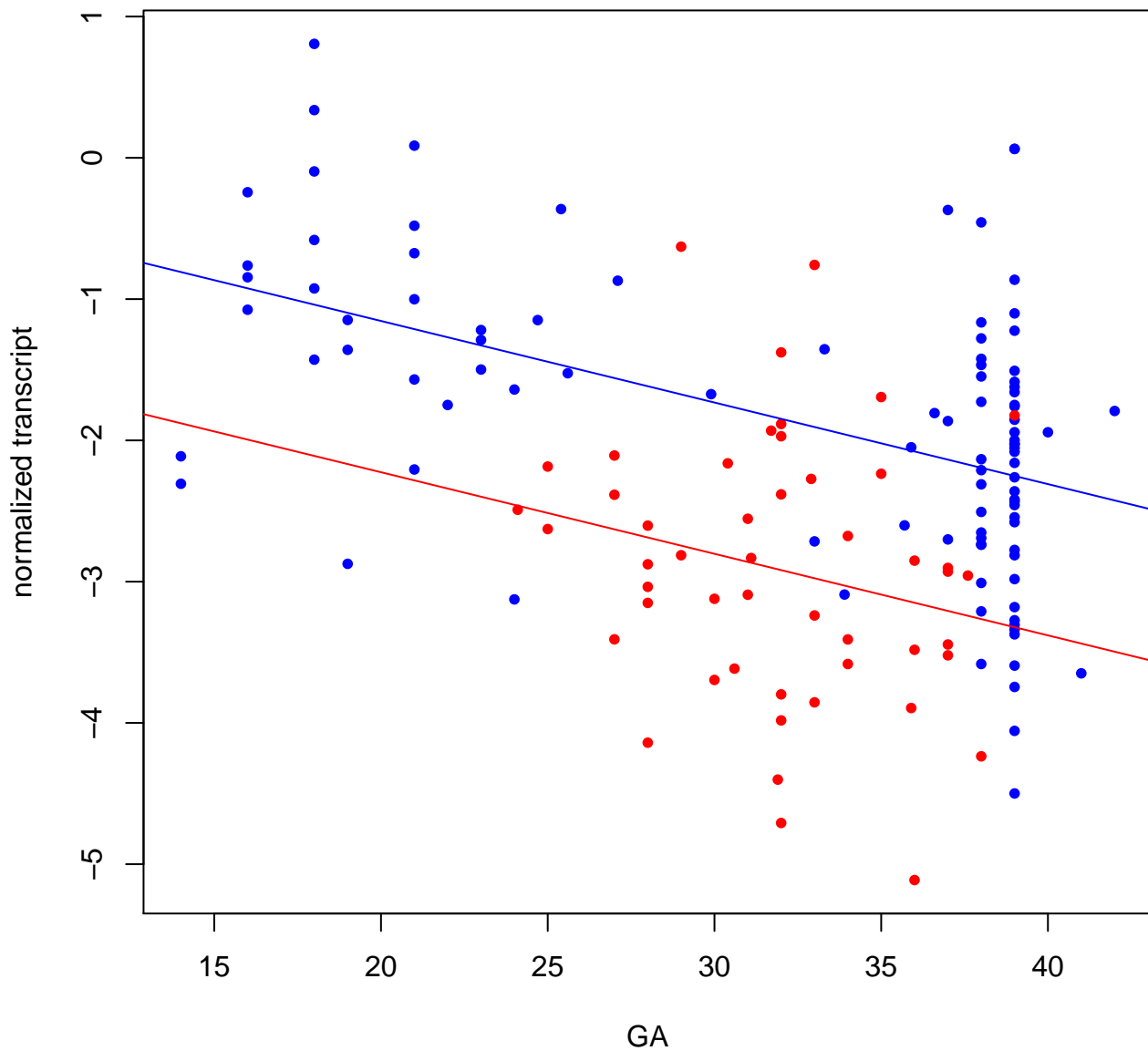

Supplement: Figure S1 — Variation in transcription values depending on gestational age for all transcripts that were significantly associated with preeclampsia (PE). Normalised transcription values are plotted for different gestational ages (weeks). The red points represent PE pregnancies and blue points normal pregnancies (NP). The lines are the estimated regression lines for gestational age (red line for PE and blue line for NP), separated by the regression coefficient for PE-status. (PDF) [file pone.0069848.s001.pdf]
