# Supplementary material for: Differential Gene Expression at the Maternal-Fetal Interface in Preeclampsia Is Influenced by Gestational Age
Source: PLoS One. 2013 Jul 31;8(7):e69848. doi: 10.1371/journal.pone.0069848 (PMC3729459; doi:10.1371/journal.pone.0069848)
Supplement: Figure S2 — Variation in transcription values depending on gestational age for all transcripts that were significantly associated with gestational age. Normalised transcription values are plotted for different gestational ages (weeks). The red points represent preeclamptic (PE) pregnancies, and blue points normal pregnancies (NP). The lines are the estimated regression lines for gestational age (red line for PE and blue line for NP), separated by the regression coefficient for PE-status. (PDF) [file pone.0069848.s002.pdf]

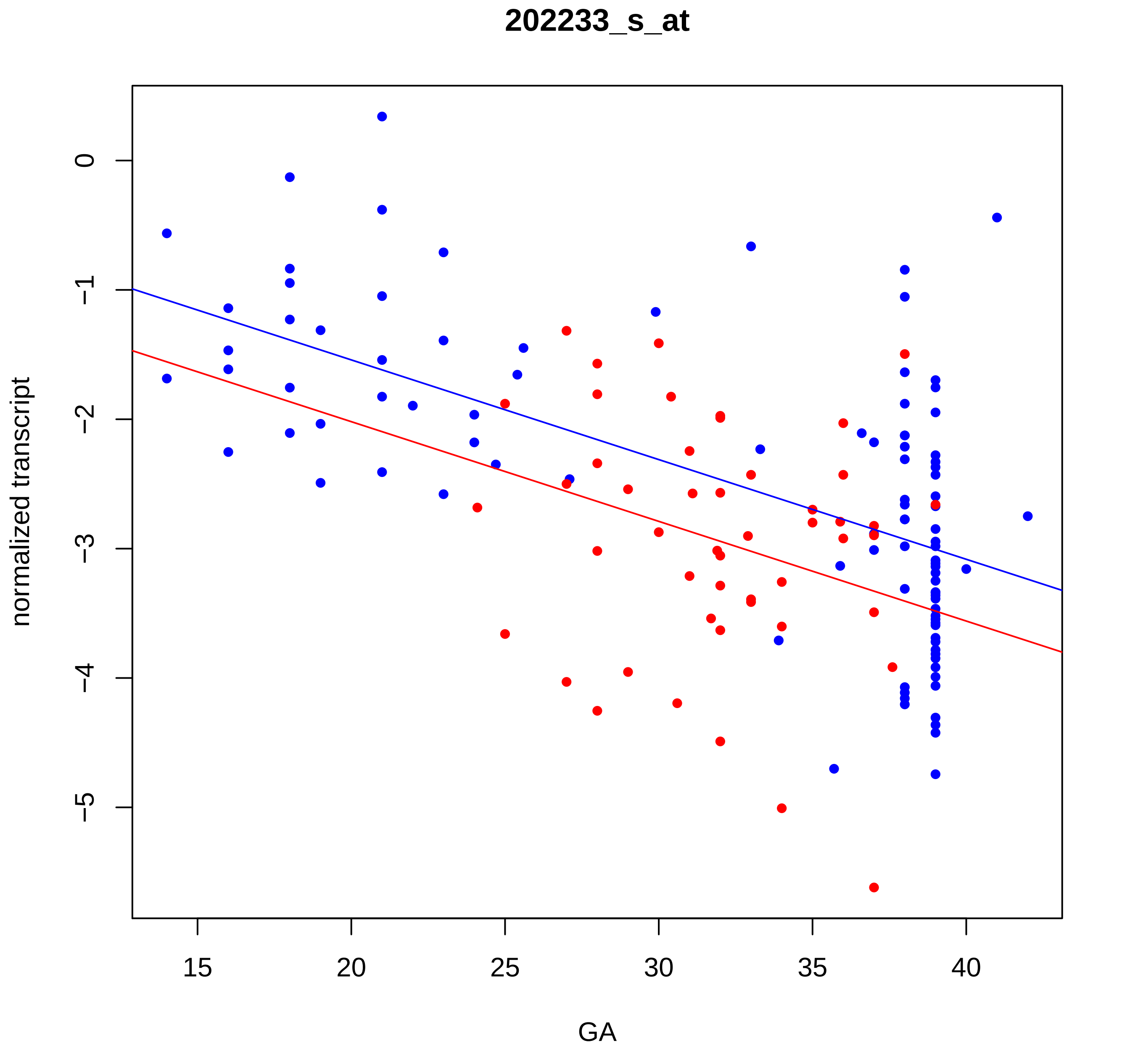
202233_s_at
normalized transcript
0
−1
−2
−3
−4
−5
15
20
25
30
35
40
GA

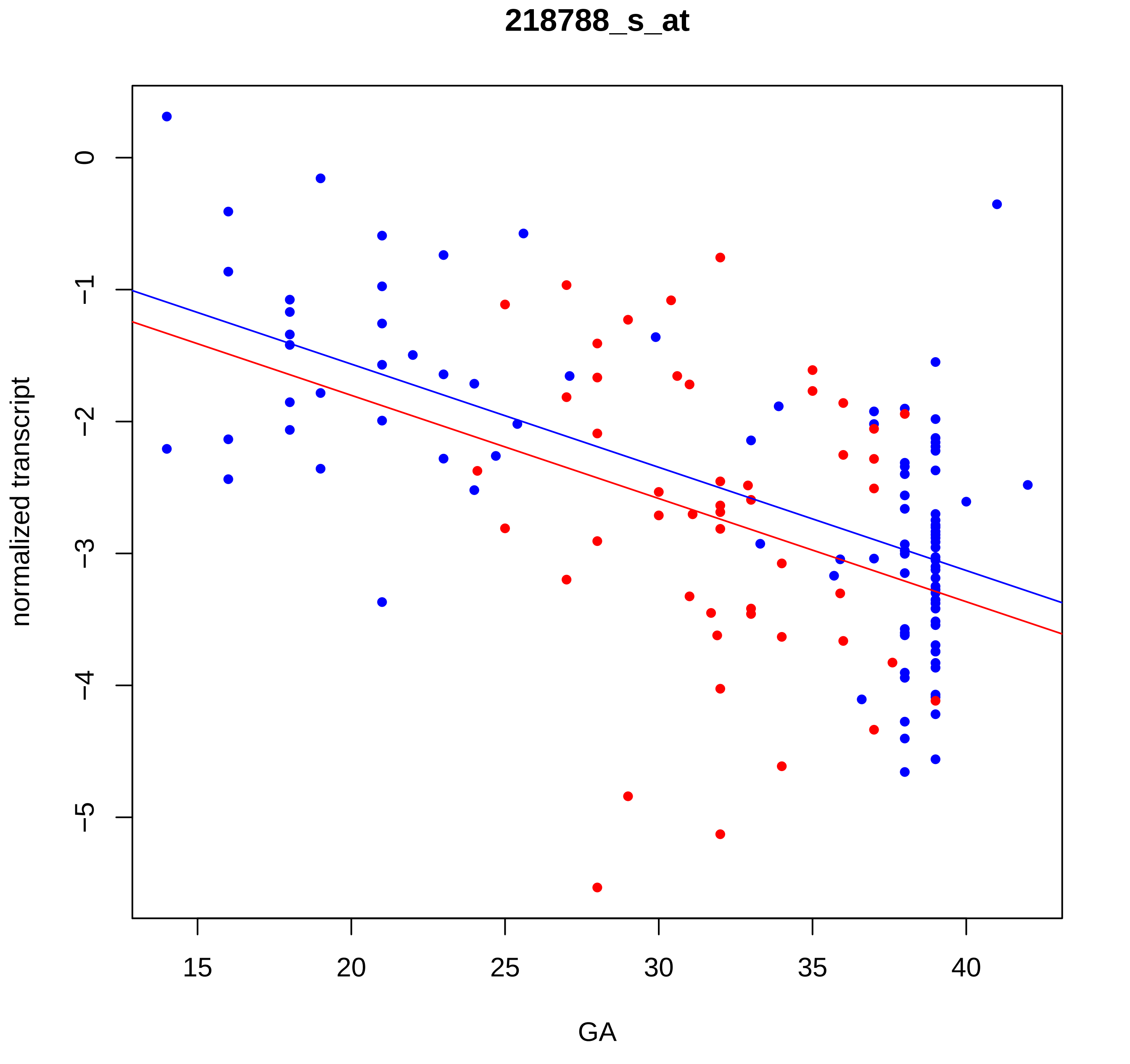
218788_s_at
normalized transcript
GA
0
−1
−2
−3
−4
−5
15
20
25
30
35
40

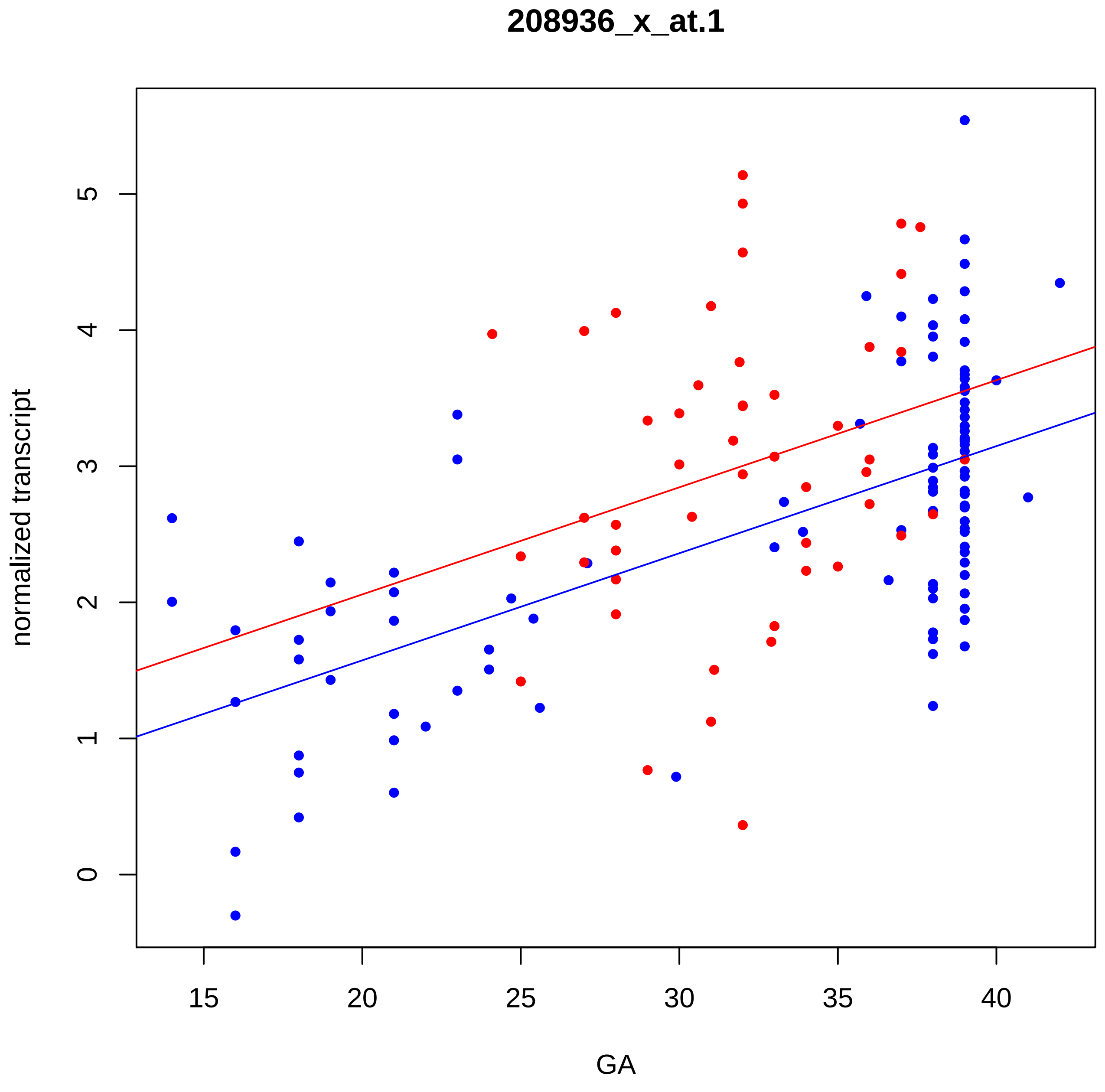

208936_x_at.1
normalized transcript
0
1
2
3
4
5
15
20
25
30
35
40
GA

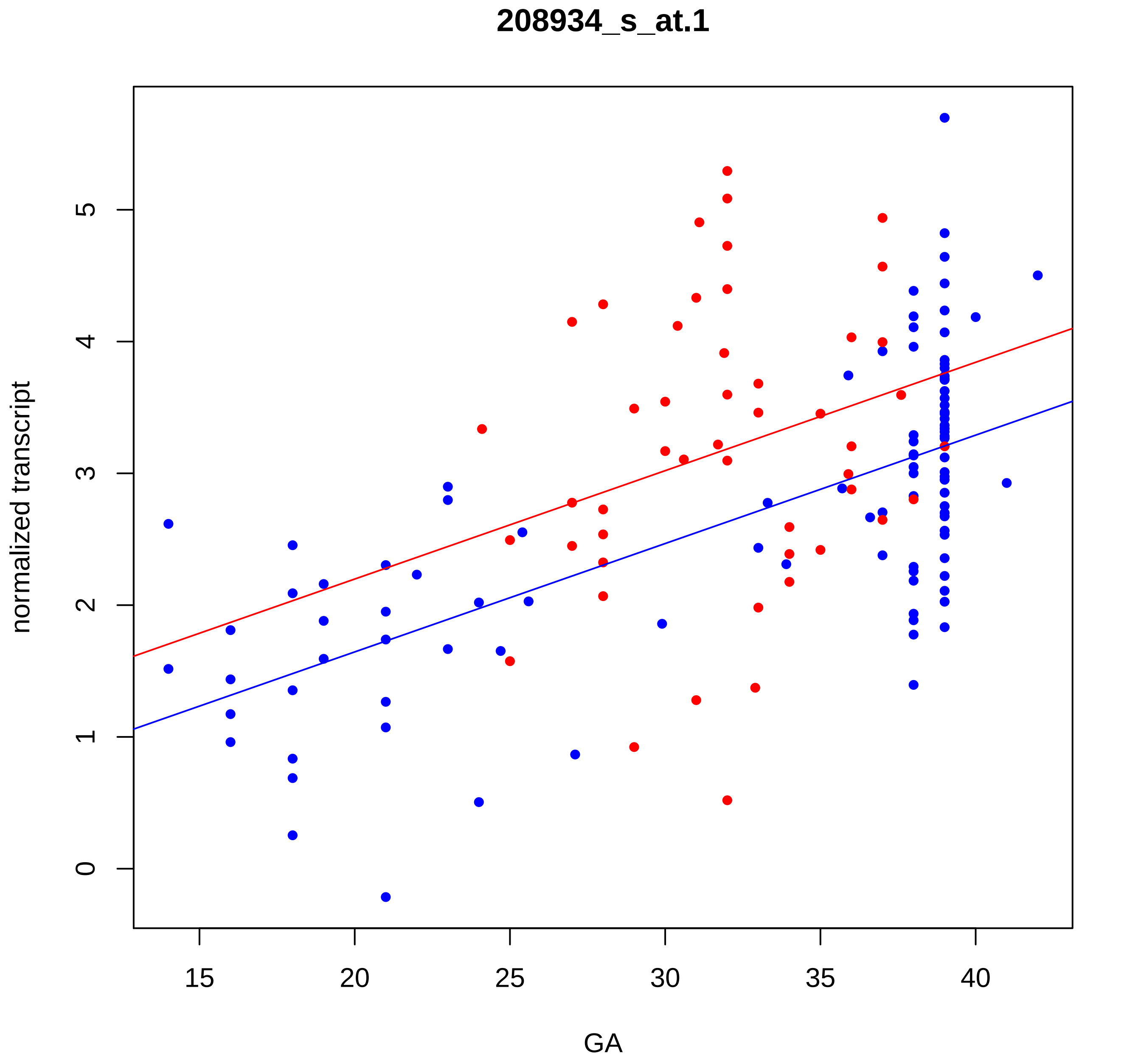
208934_s_at.1
normalized transcript
GA
0
1
2
3
4
5
15
20
25
30
35
40

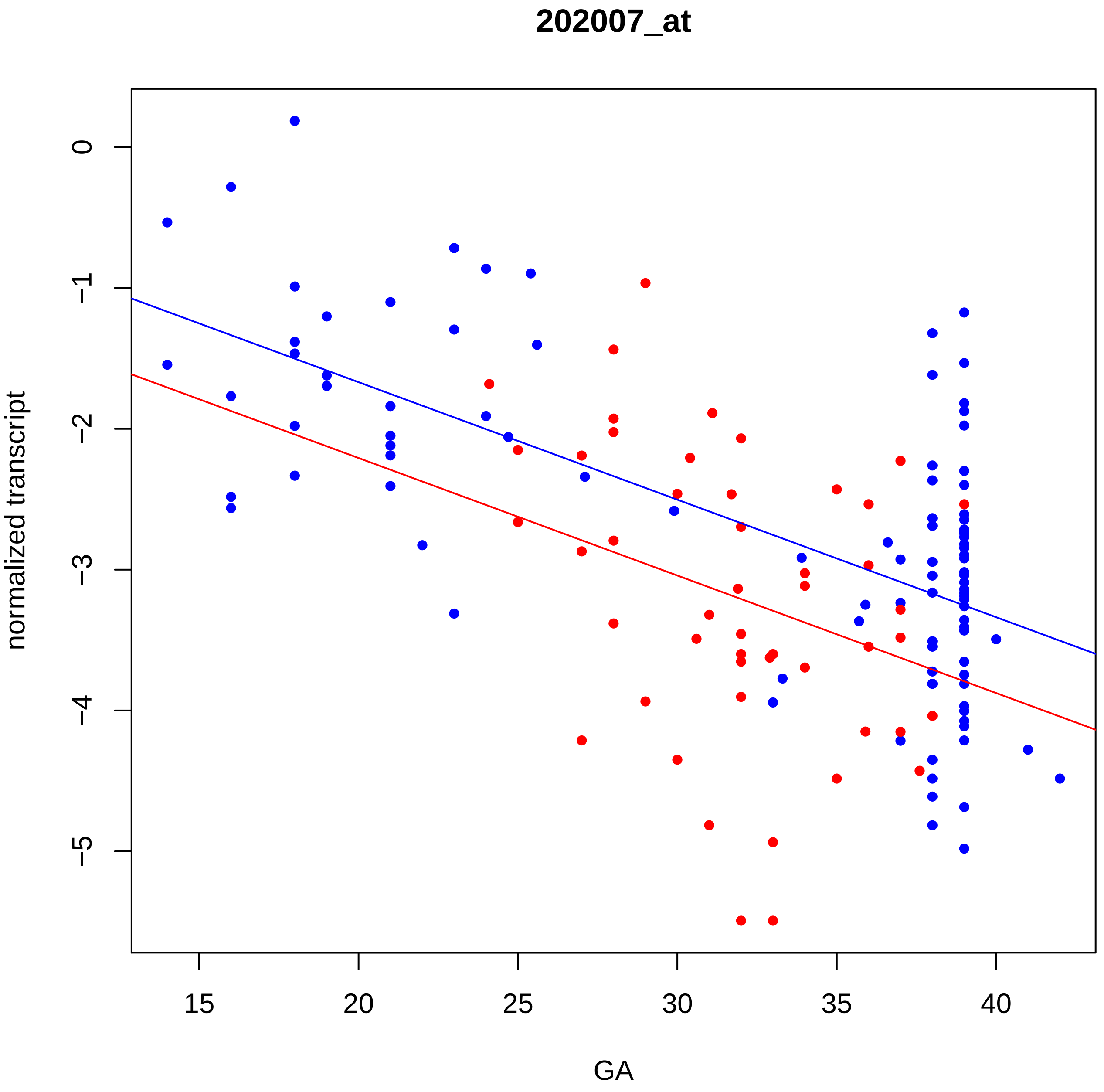
202007_at
normalized transcript
GA
0
−1
−2
−3
−4
−5
15
20
25
30
35
40

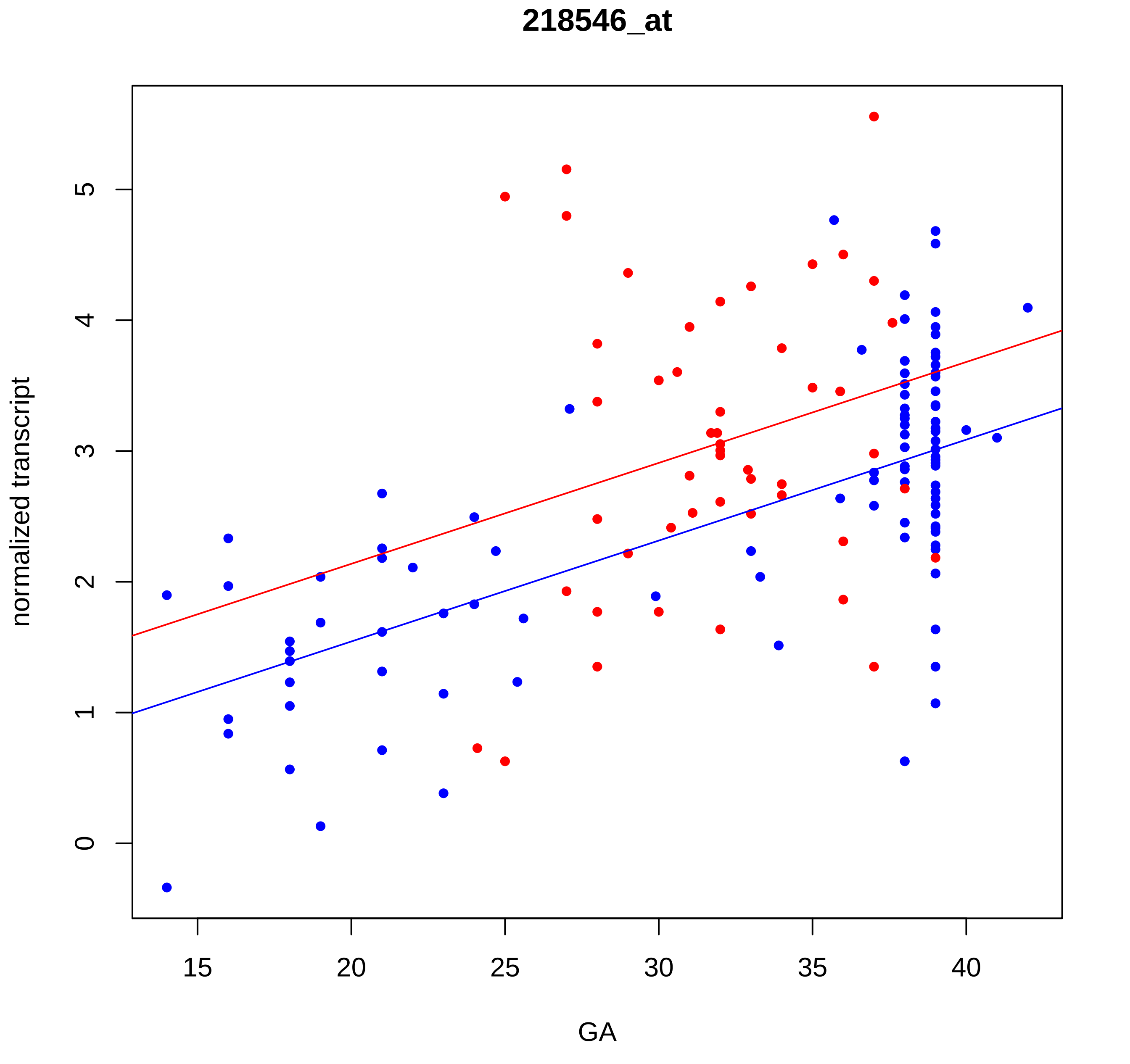

218546_at
normalized transcript
GA
0
1
2
3
4
5
15
20
25
30
35
40

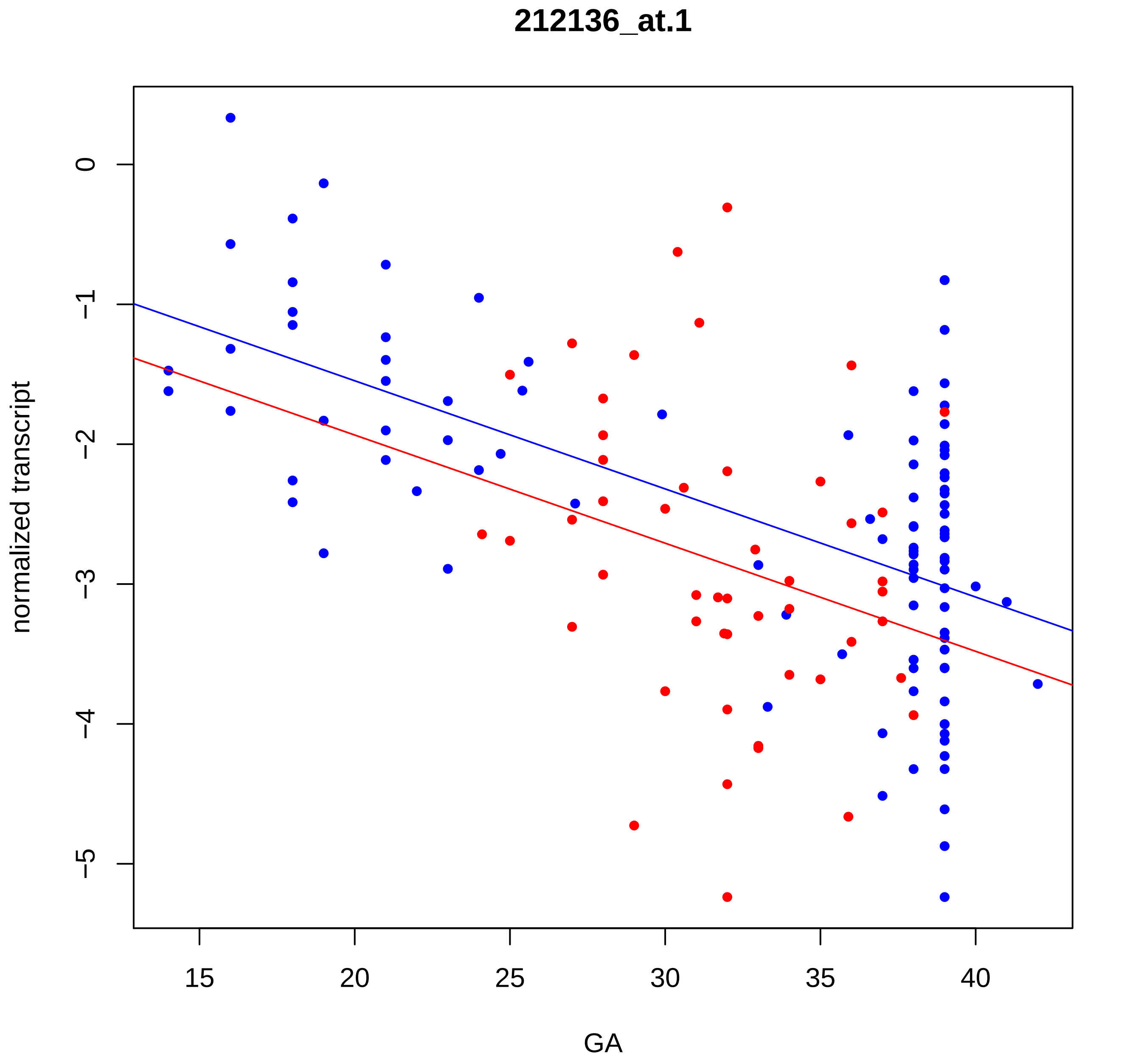

212136_at.1
normalized transcript
GA
0
−1
−2
−3
−4
−5
15
20
25
30
35
40

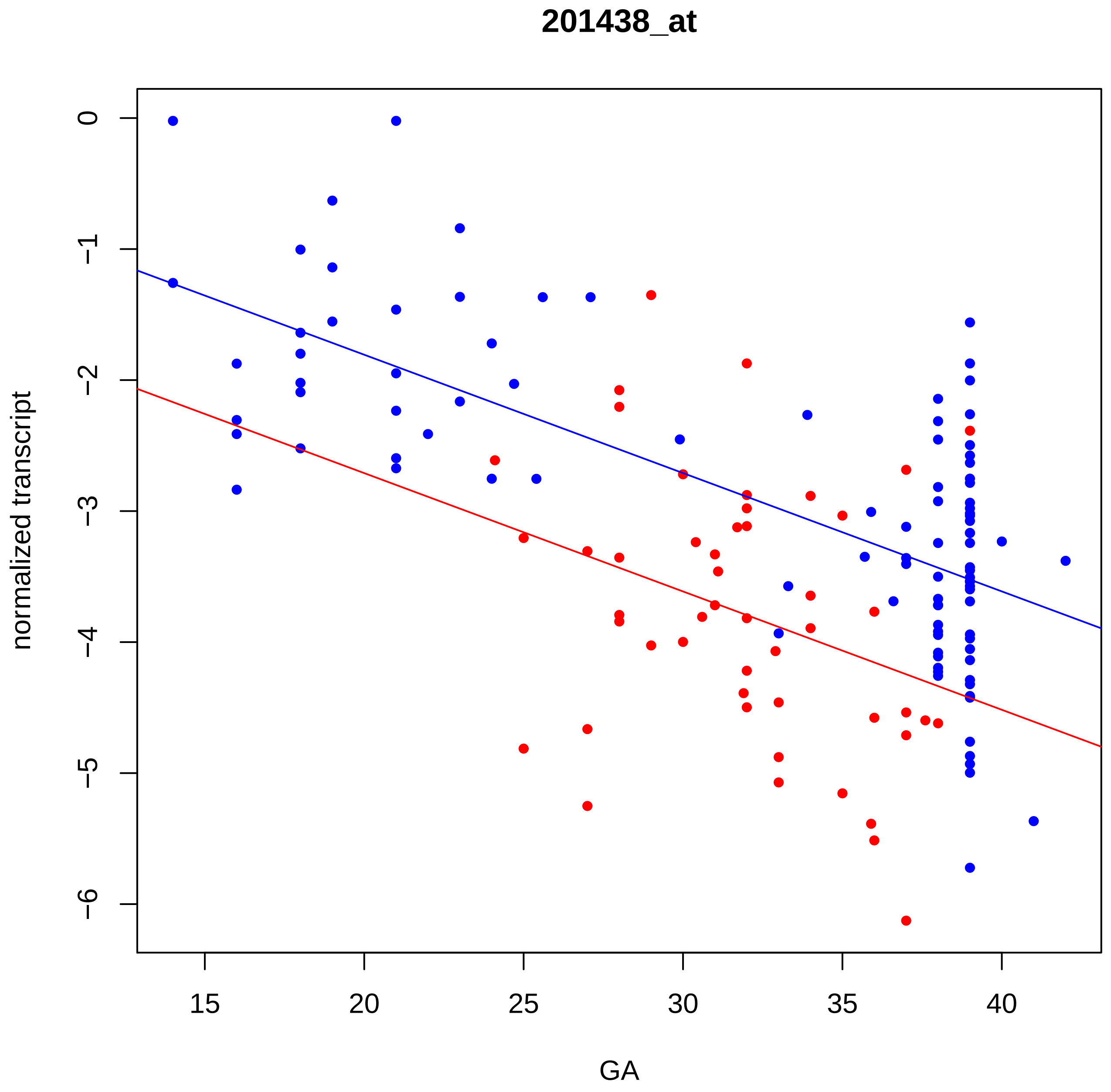
201438_at
normalized transcript
GA
0
−1
−2
−3
−4
−5
−6
15
20
25
30
35
40

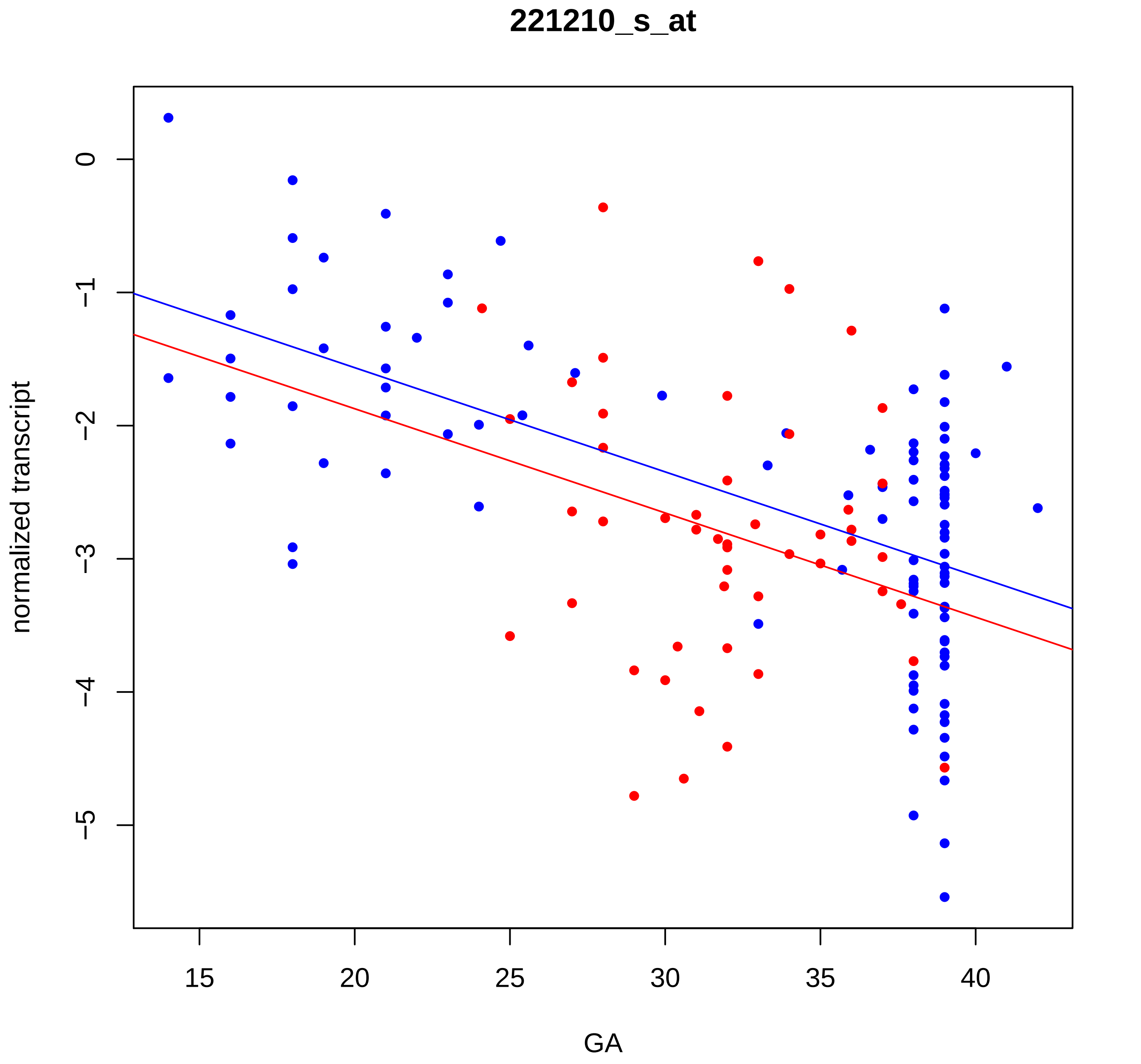
221210_s_at
normalized transcript
GA
0
−1
−2
−3
−4
−5
15
20
25
30
35
40

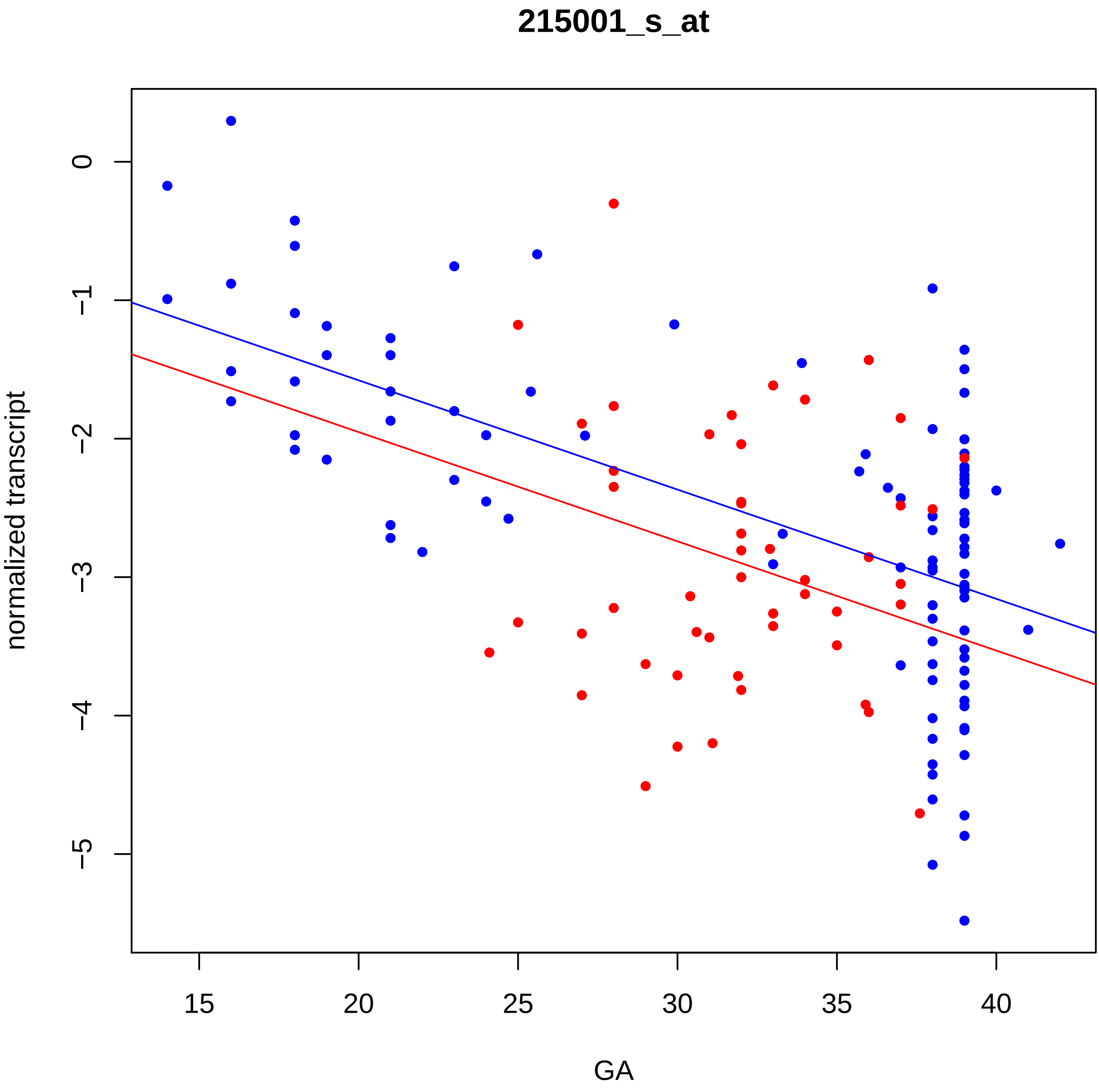

215001_s_at
normalized transcript
0
−1
−2
−3
−4
−5
15
20
25
30
35
40
GA

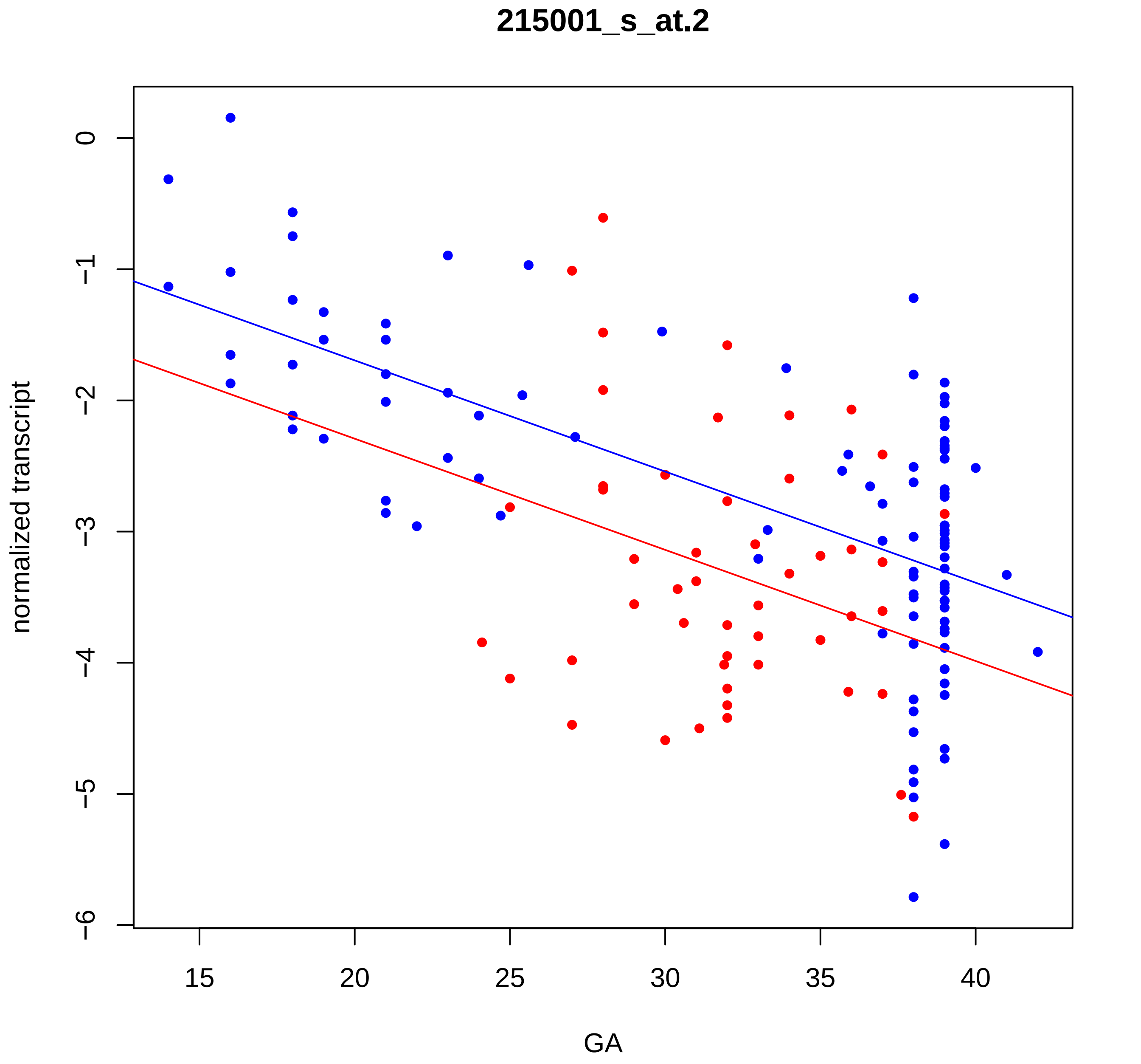
215001_s_at.2
normalized transcript
GA
0
−1
−2
−3
−4
−5
−6
15
20
25
30
35
40

209343_at

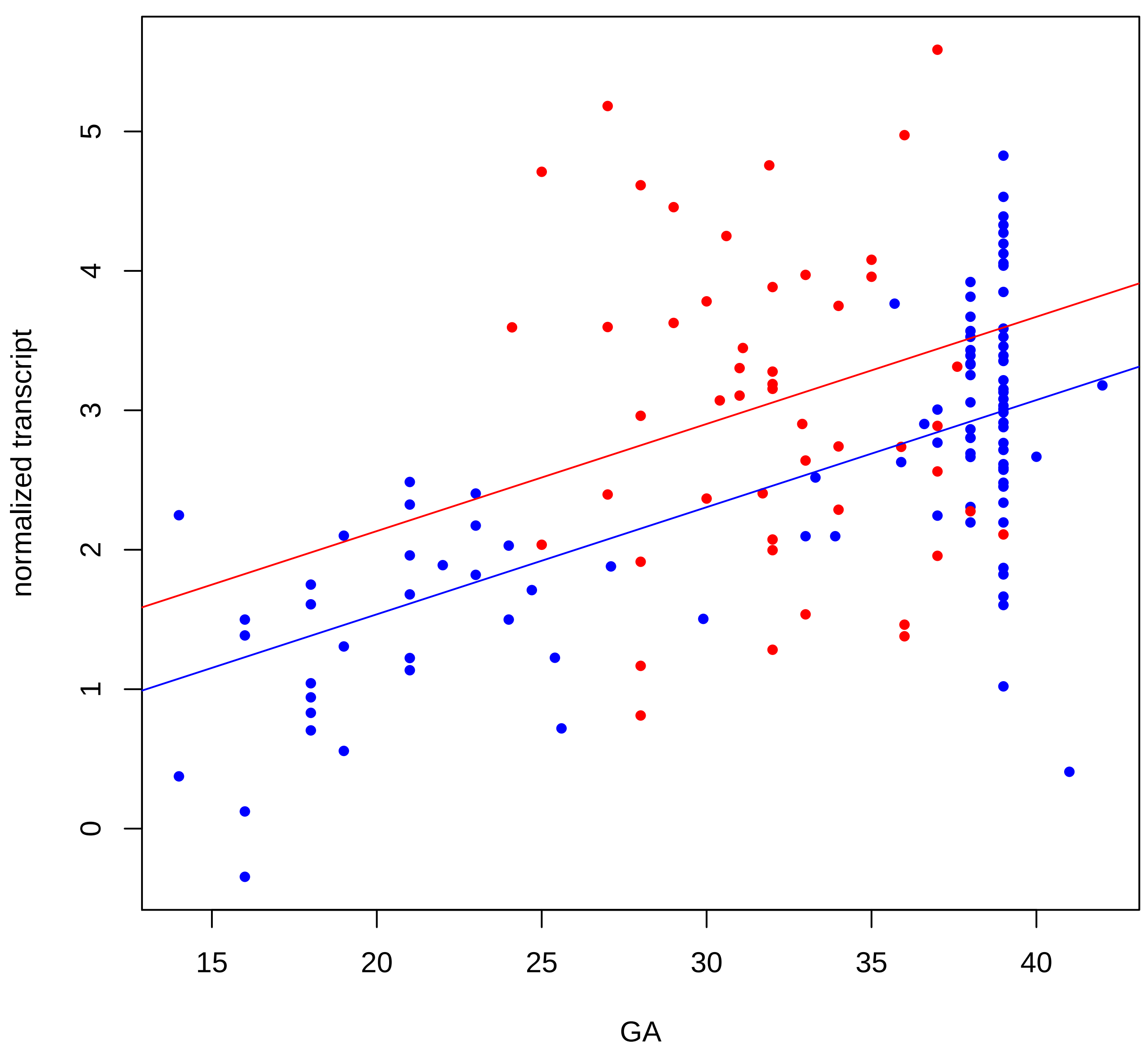

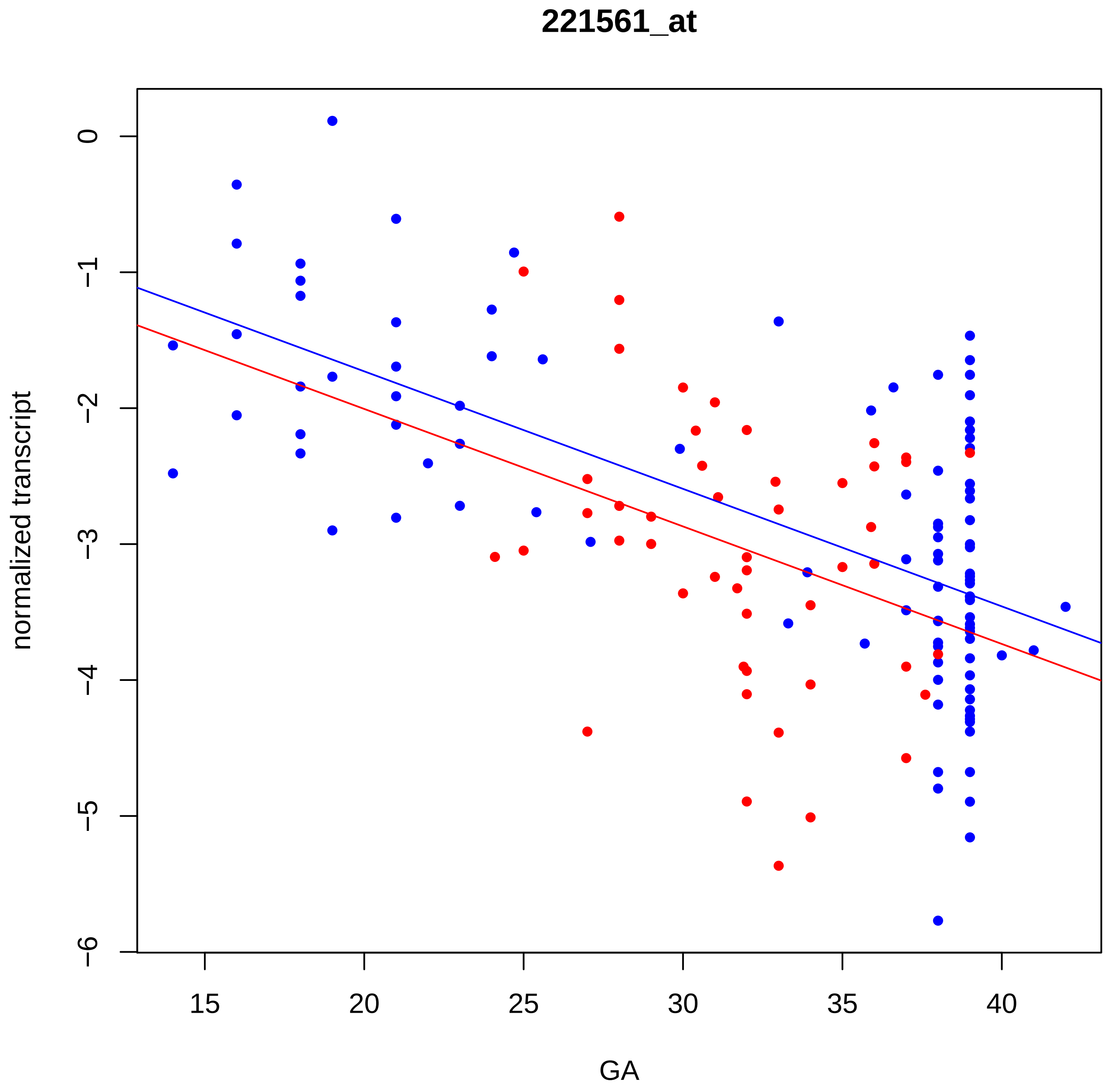

221561_at
normalized transcript
0
−1
−2
−3
−4
−5
−6
15
20
25
30
35
40
GA

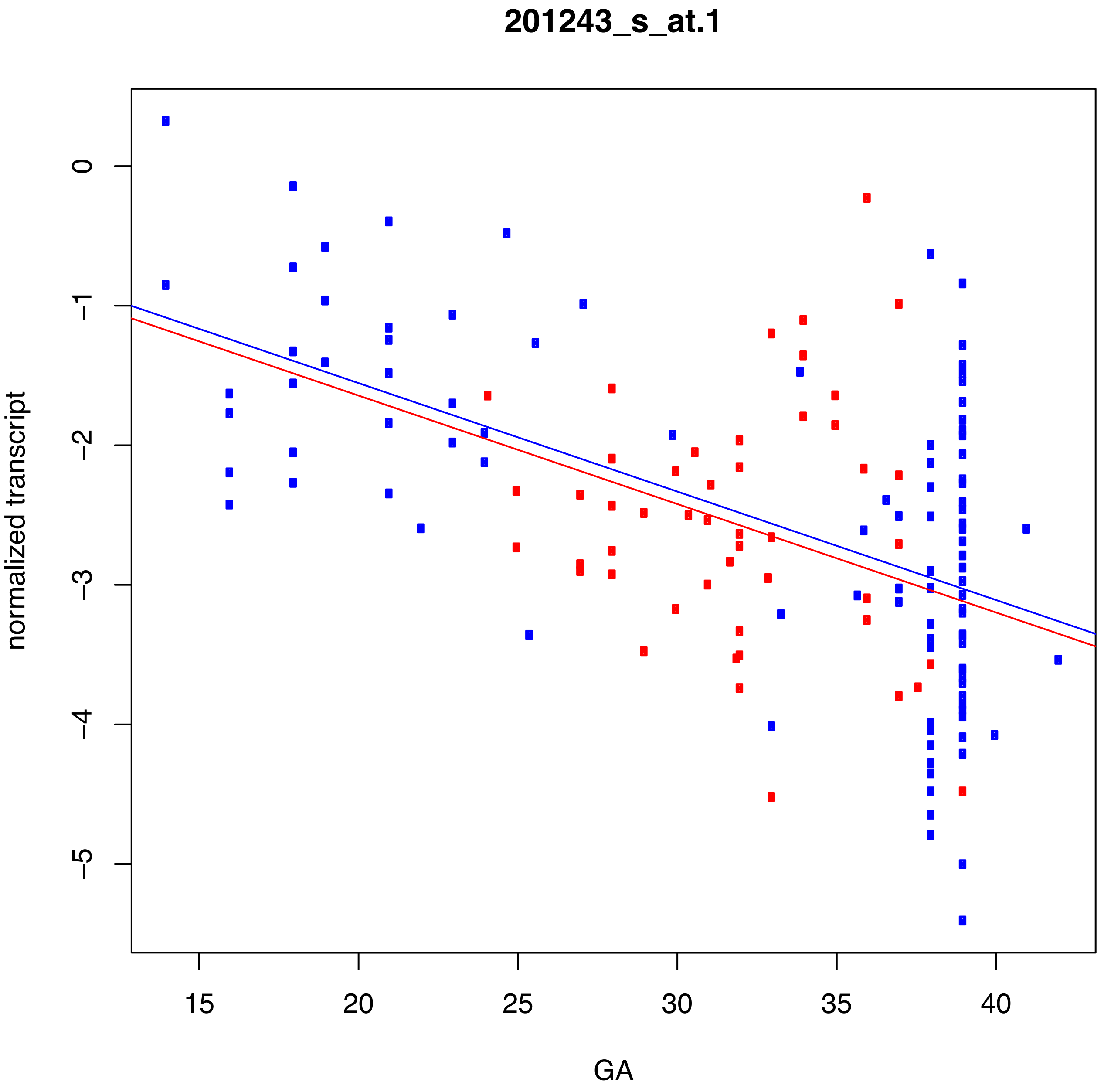
201243_s_at.1
normalized transcript
GA
0
−1
−2
−3
−4
−5
15
20
25
30
35
40

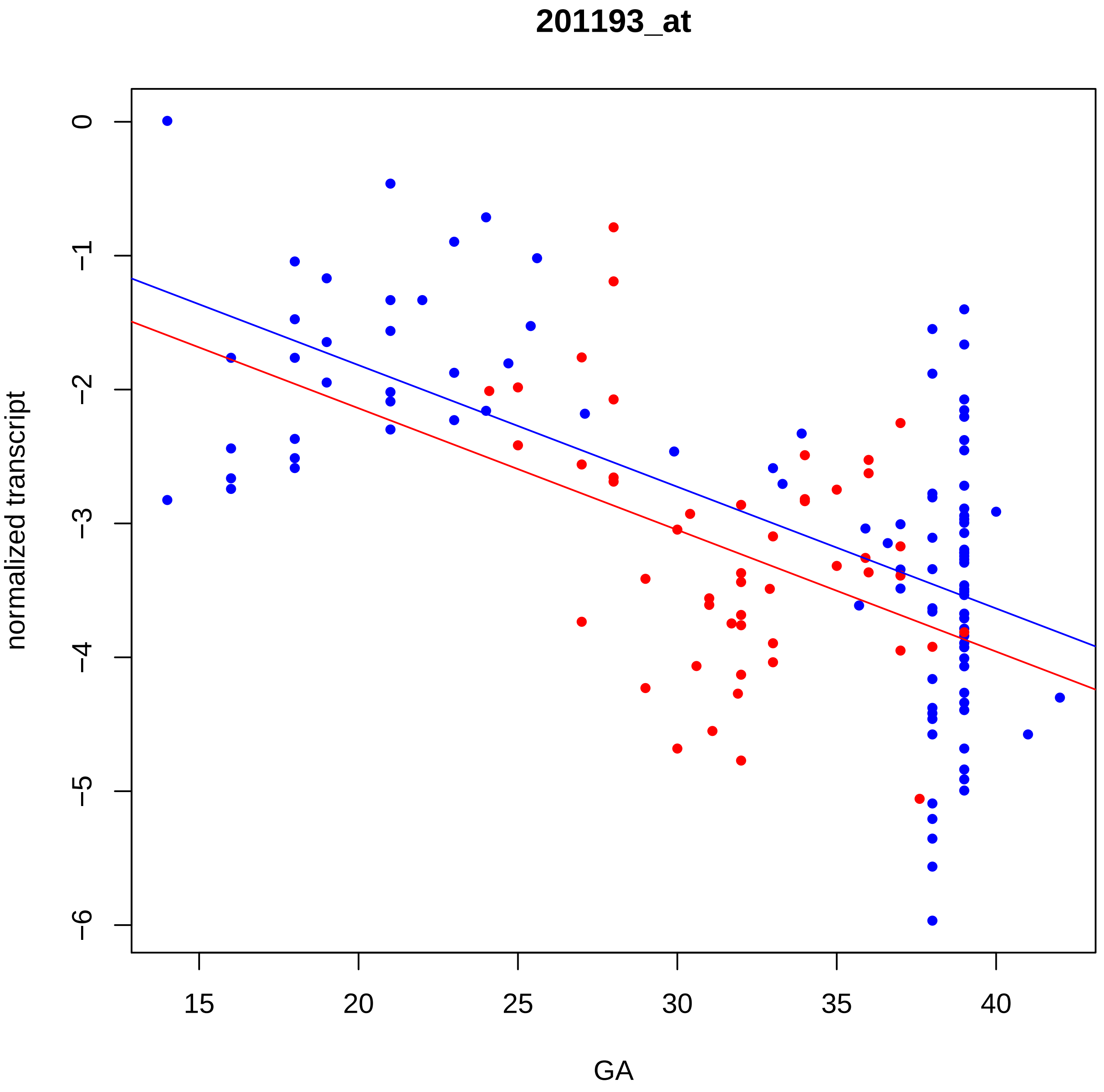

201193_at
0
−1
−2
−3
−4
−5
−6
normalized transcript
15
20
25
30
35
40
GA

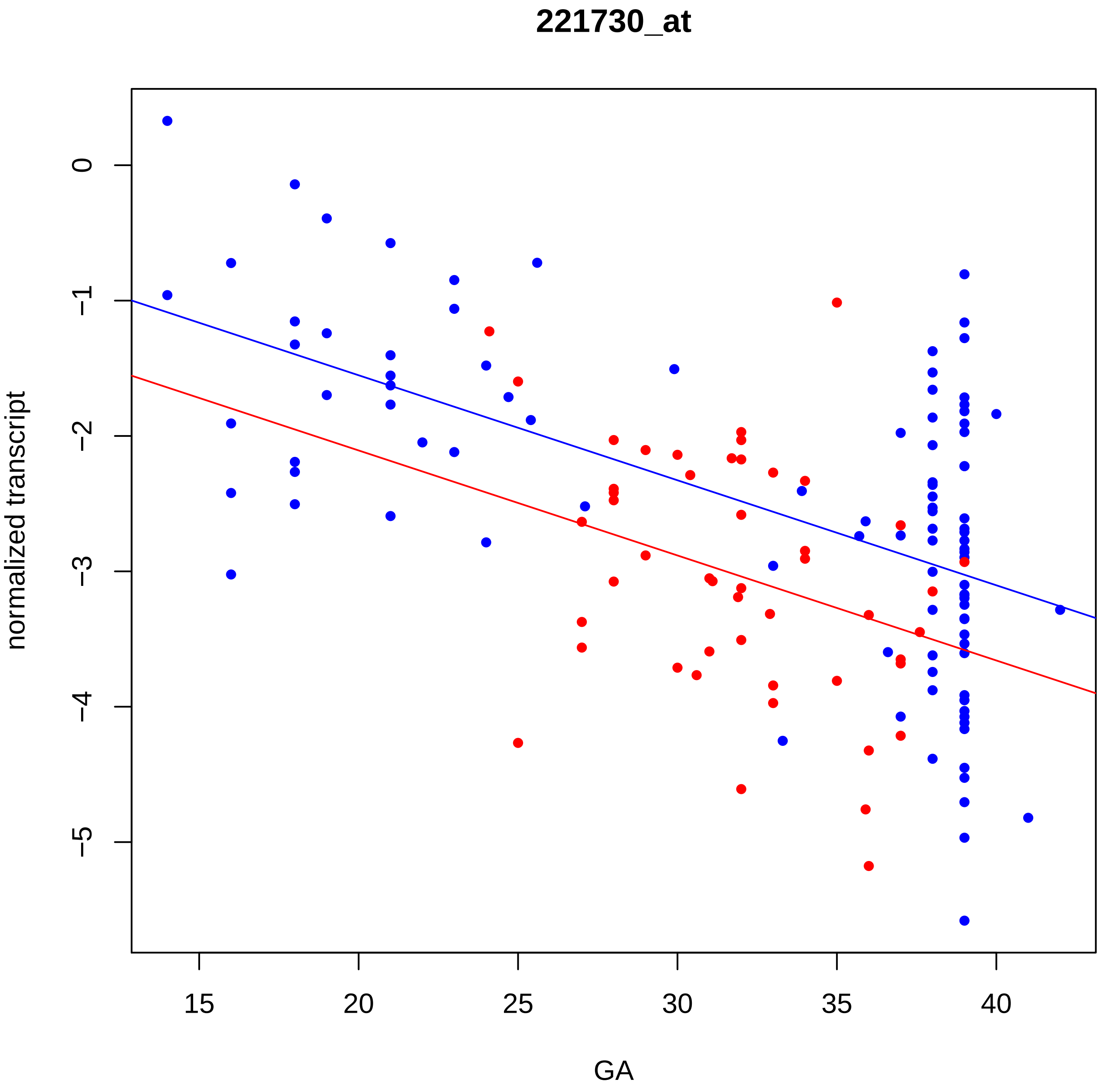
221730_at
normalized transcript
GA
0
−1
−2
−3
−4
−5
15
20
25
30
35
40

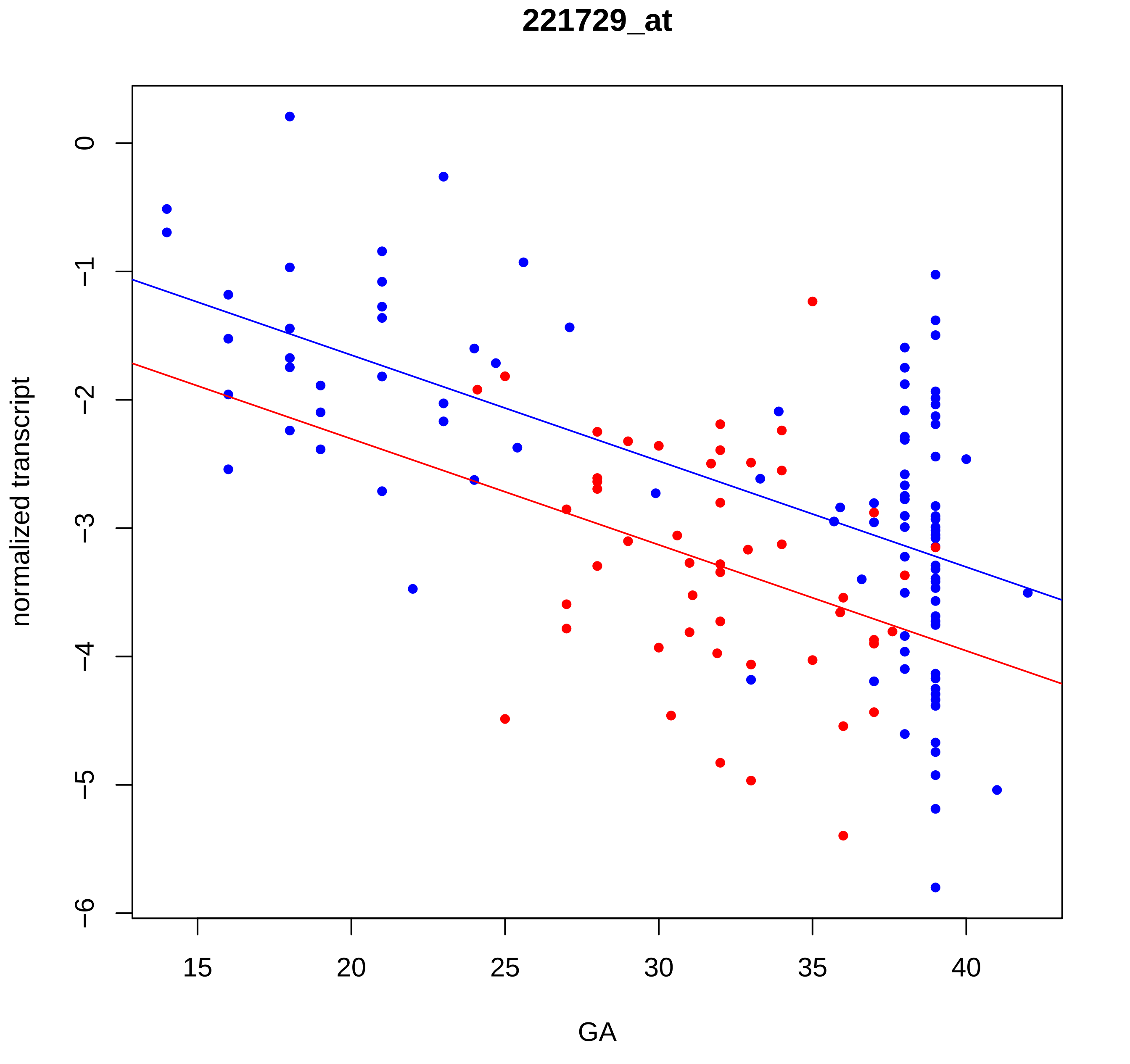
221729_at
normalized transcript
GA
0
−1
−2
−3
−4
−5
−6
15
20
25
30
35
40

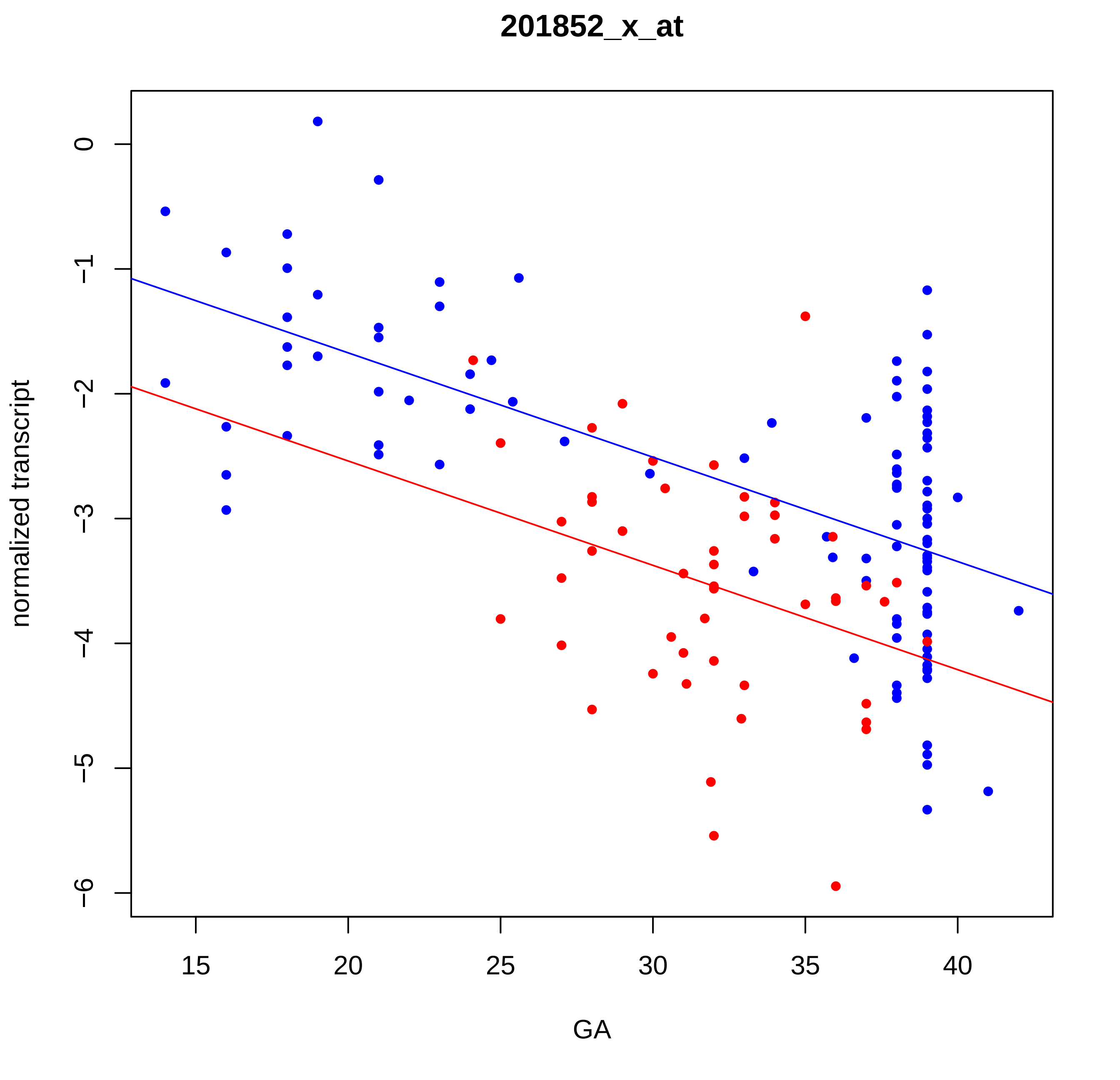
201852_x_at
normalized transcript
GA
0
−1
−2
−3
−4
−5
−6
15
20
25
30
35
40

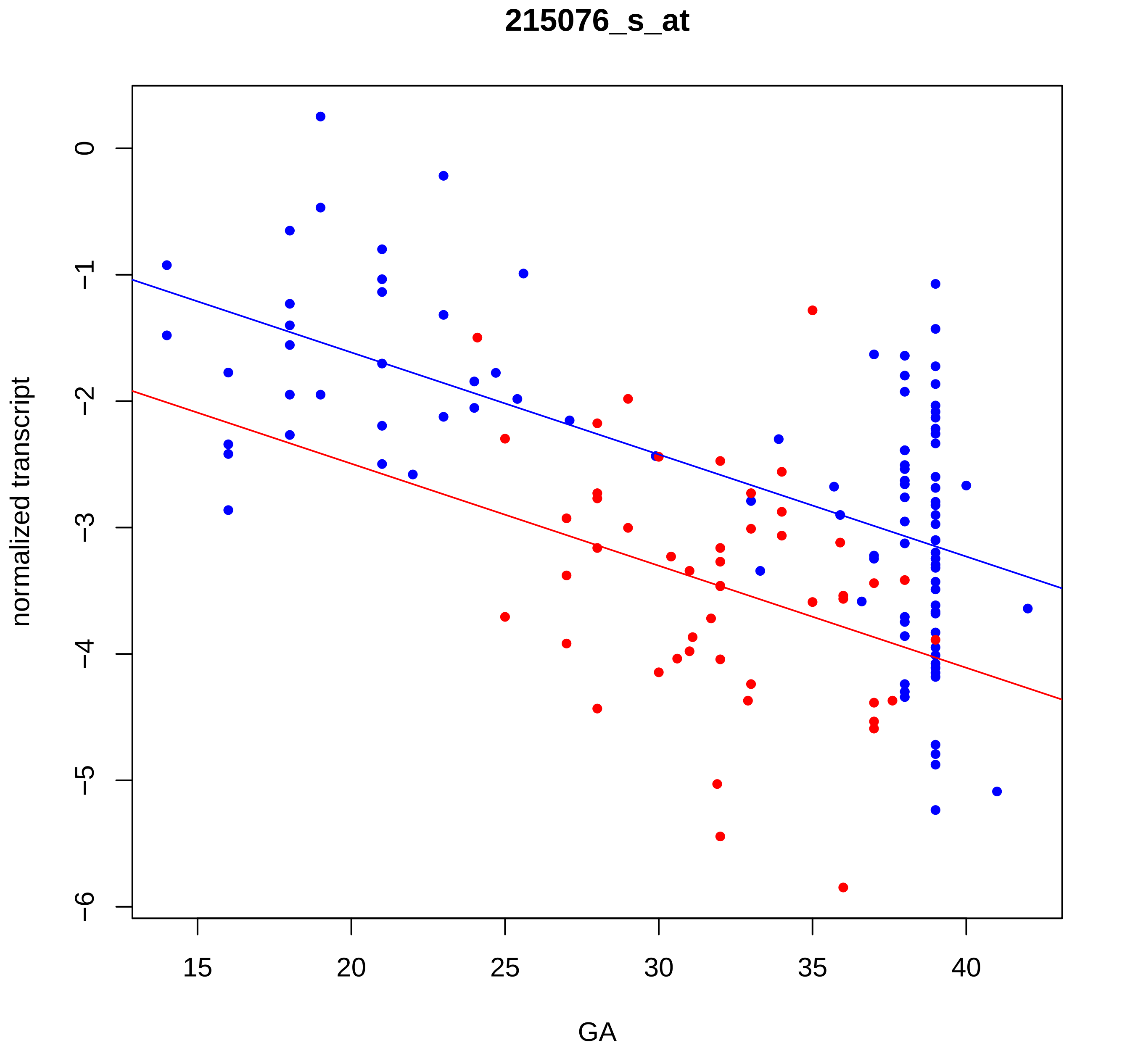

215076_s_at
normalized transcript
0
−1
−2
−3
−4
−5
−6
15
20
25
30
35
40
GA

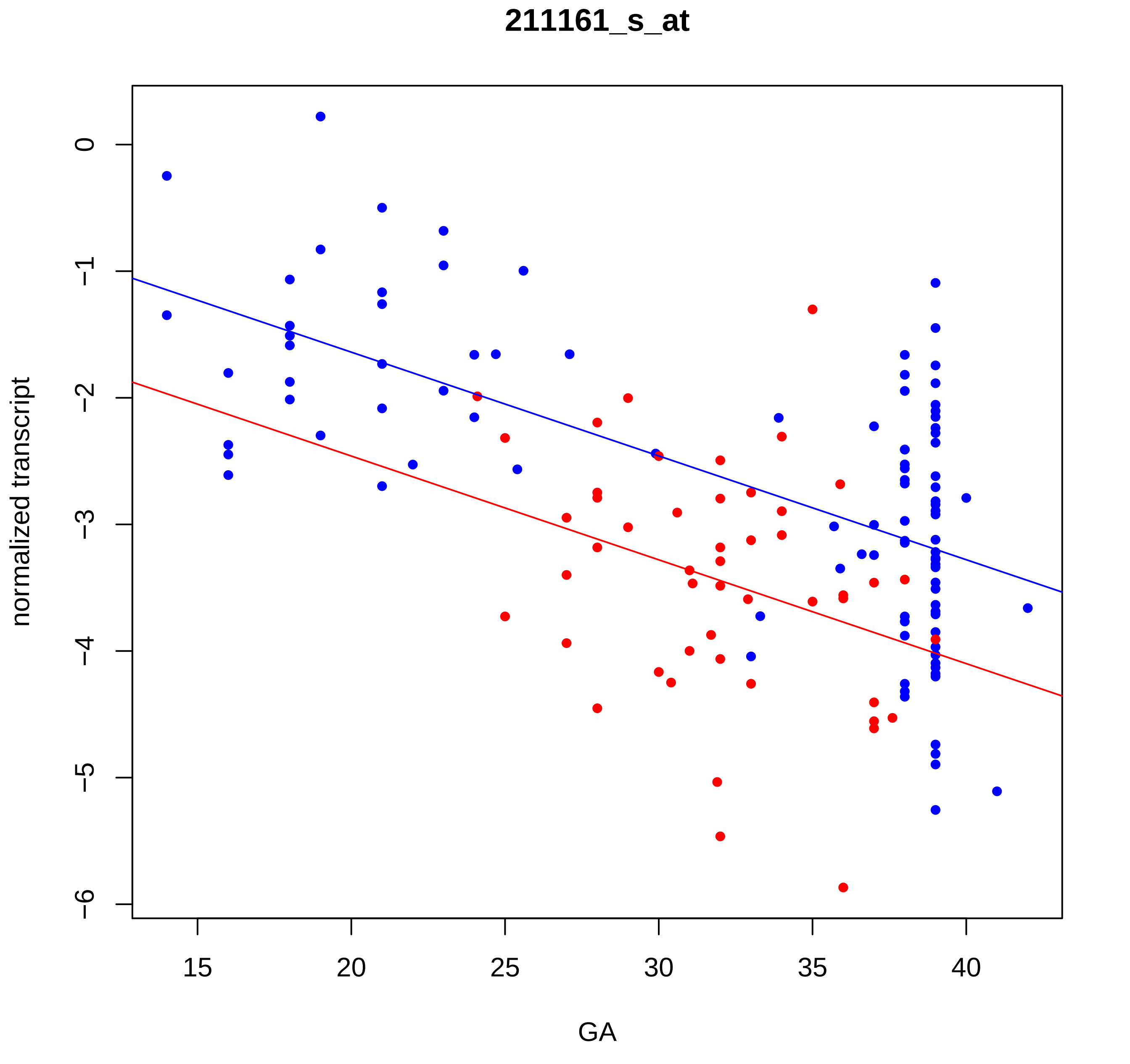

211161_s_at
normalized transcript
0
−1
−2
−3
−4
−5
−6
15
20
25
30
35
40
GA

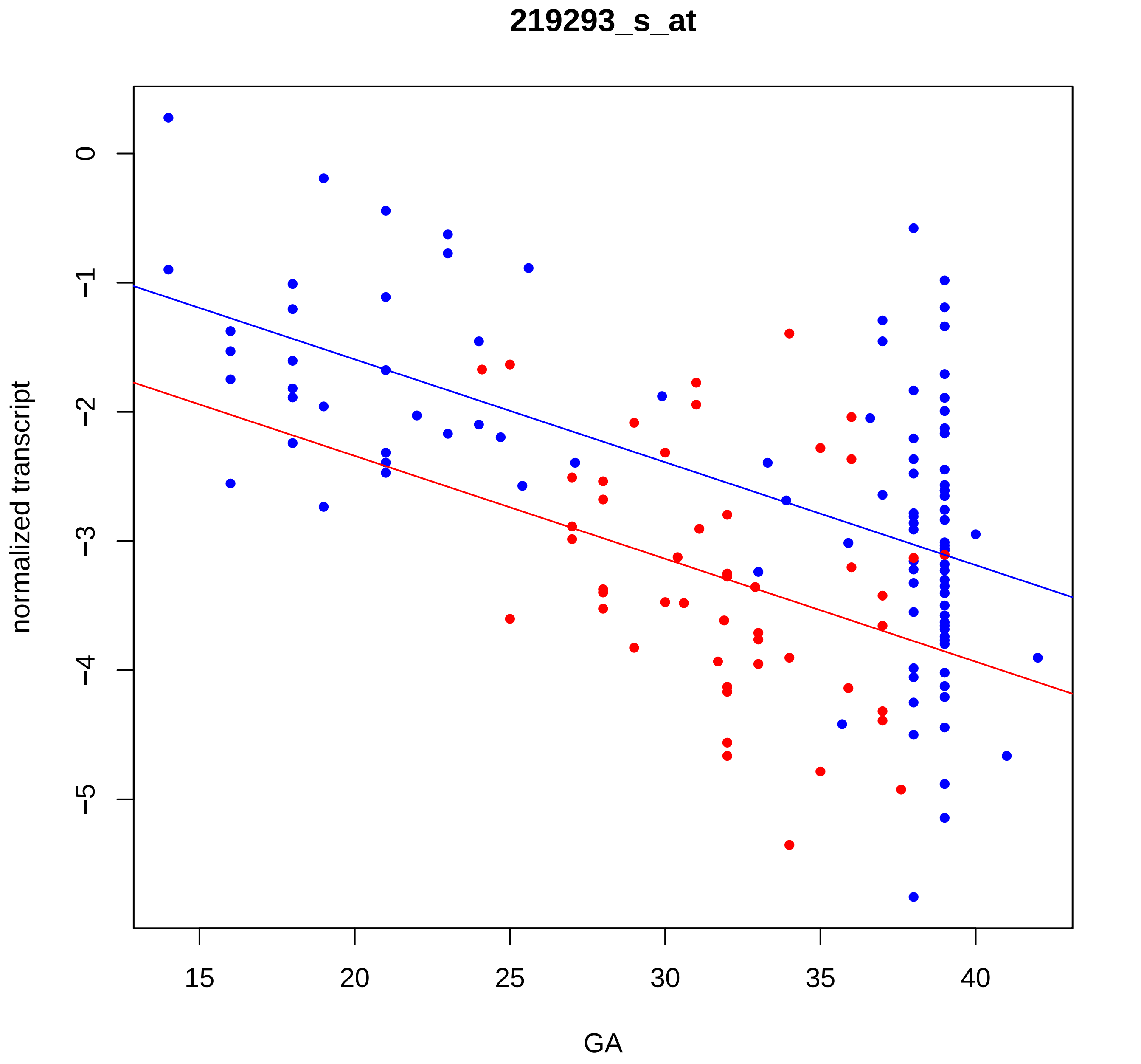

219293_s_at
normalized transcript
GA
0
−1
−2
−3
−4
−5
15
20
25
30
35
40

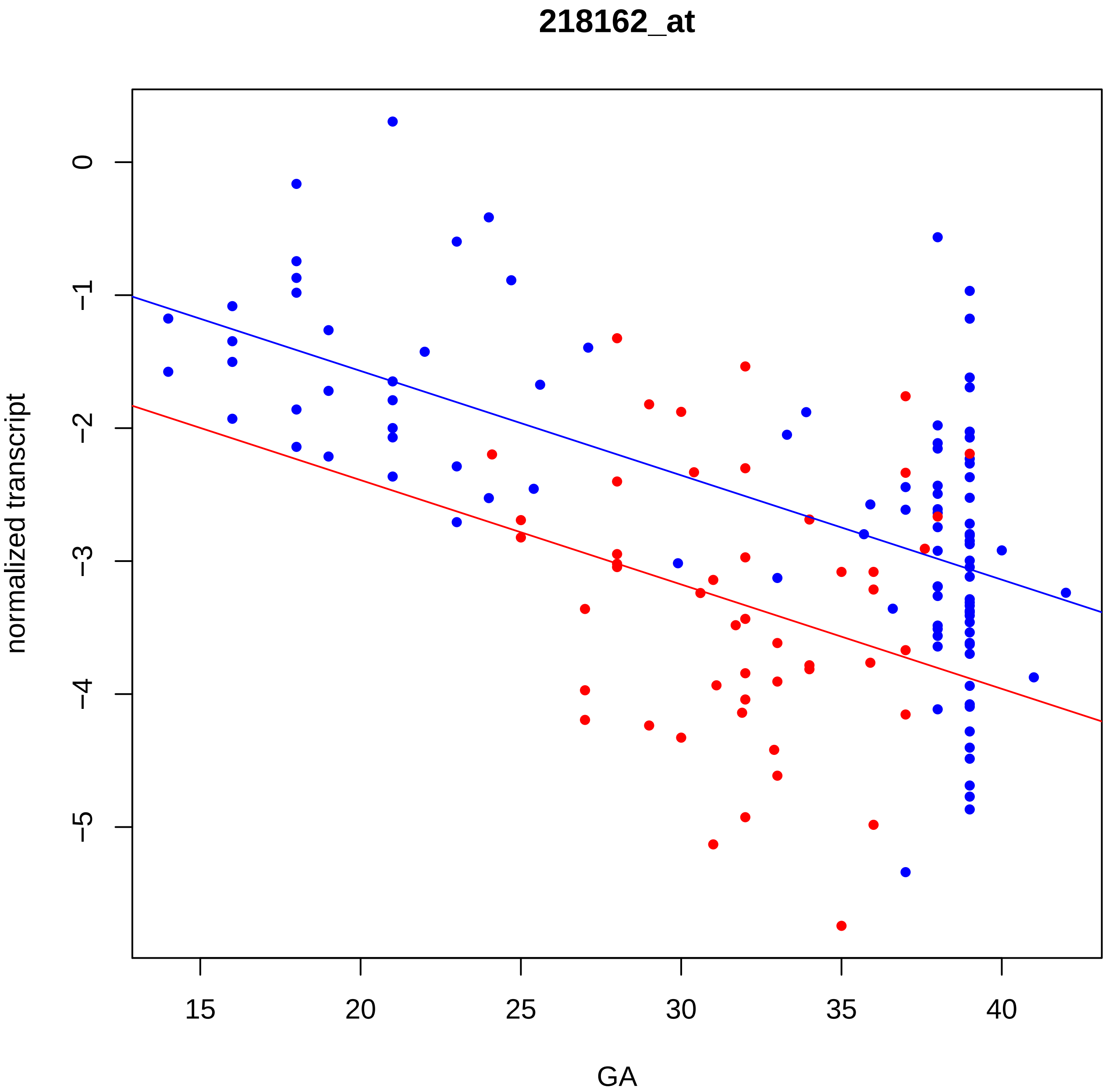

218162_at
0
−1
−2
−3
−4
−5
normalized transcript
15
20
25
30
35
40
GA

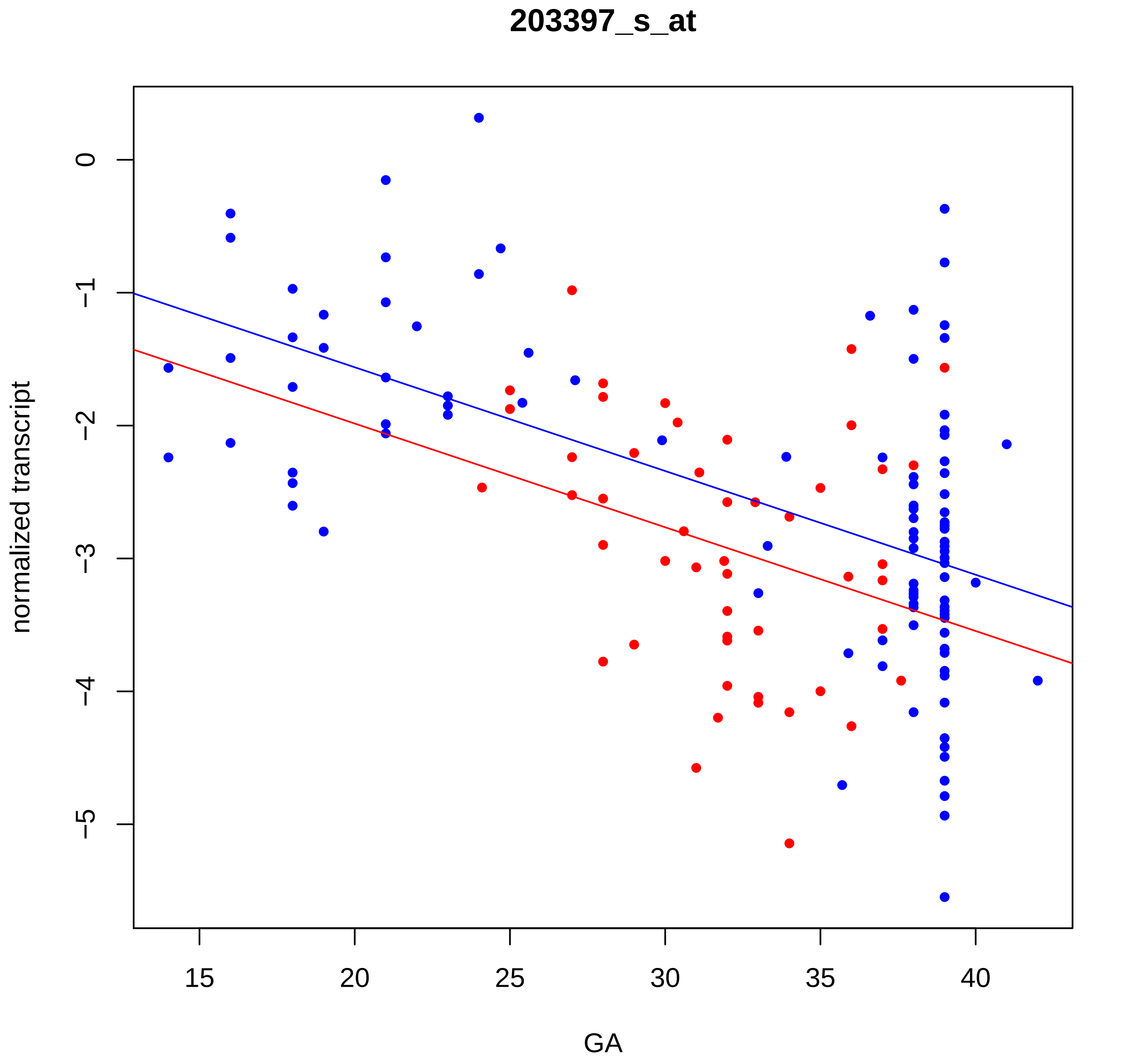
203397_s_at
normalized transcript
GA
0
−1
−2
−3
−4
−5
15
20
25
30
35
40

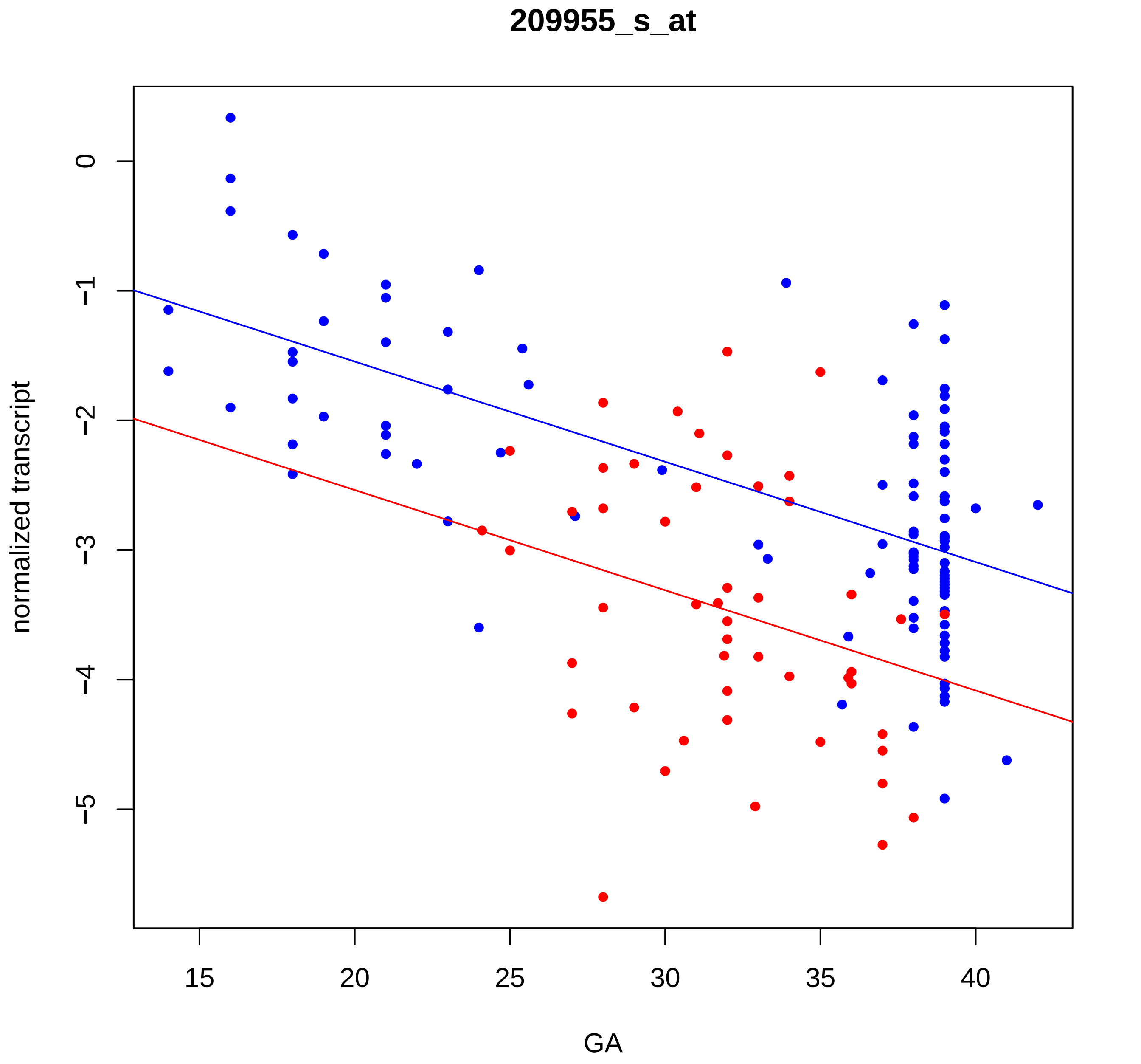
209955_s_at
normalized transcript
GA
0
−1
−2
−3
−4
−5
15
20
25
30
35
40

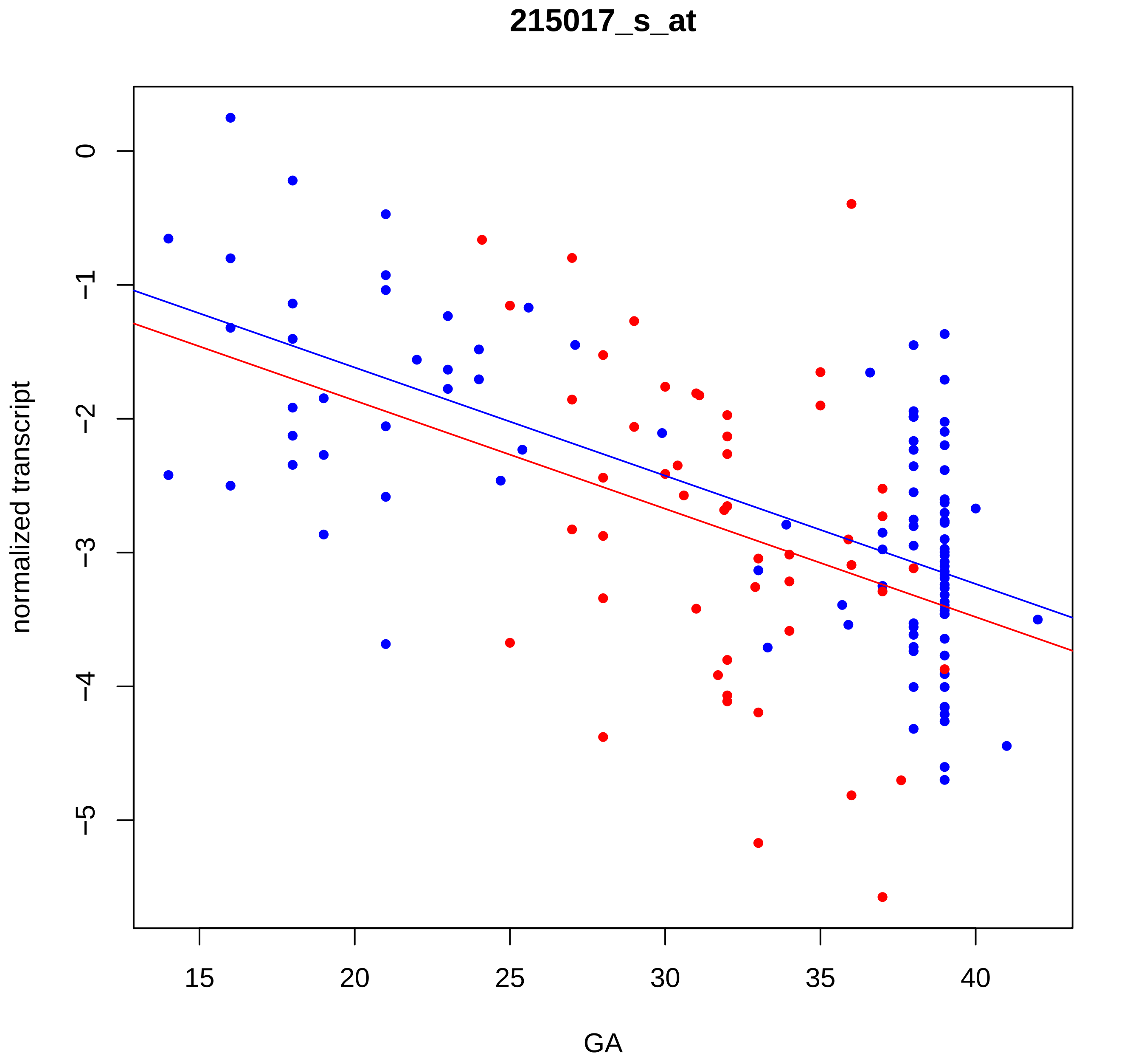
215017_s_at
normalized transcript
GA
0
−1
−2
−3
−4
−5
15
20
25
30
35
40

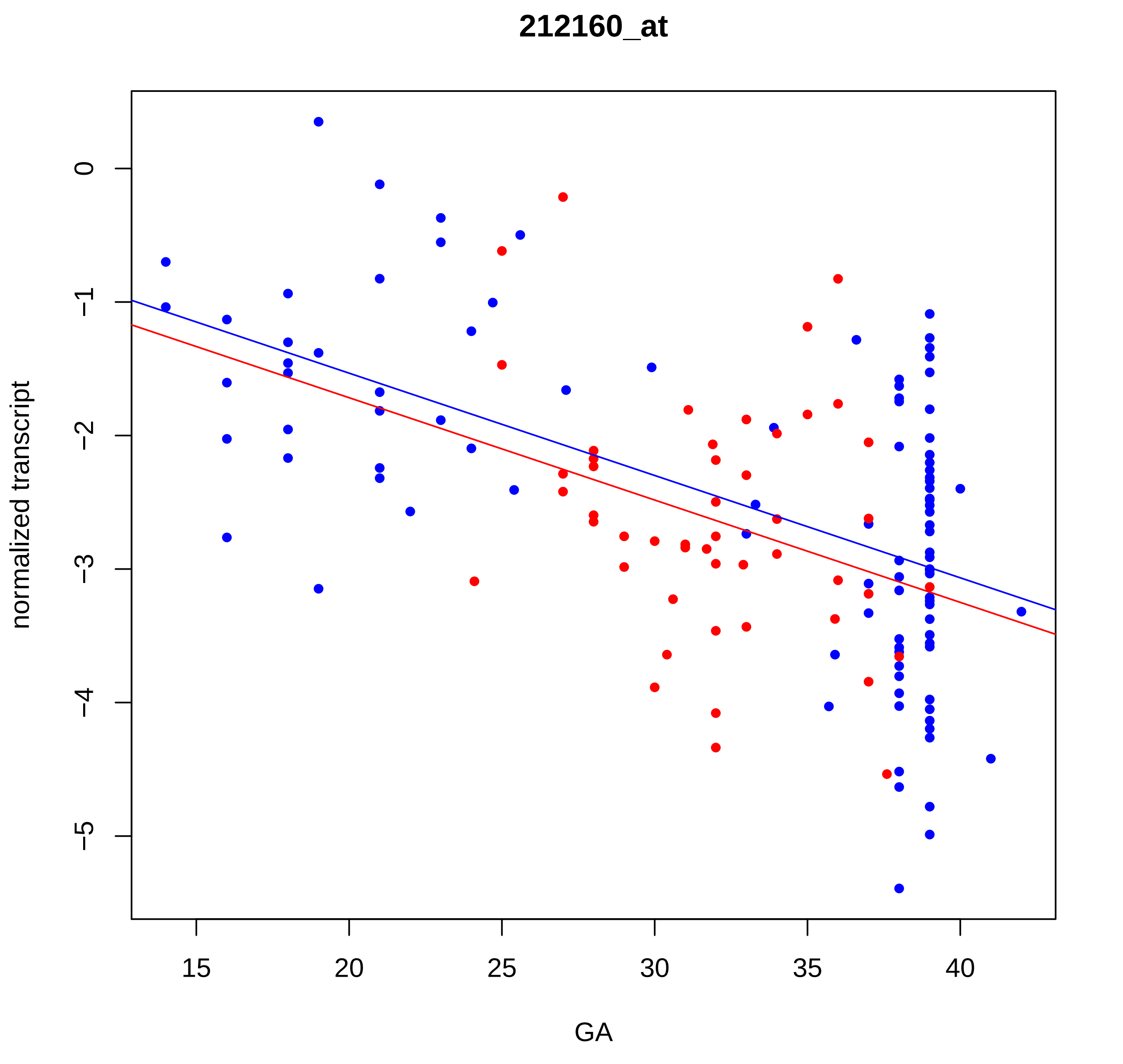

212160_at
normalized transcript
0
−1
−2
−3
−4
−5
15
20
25
30
35
40
GA

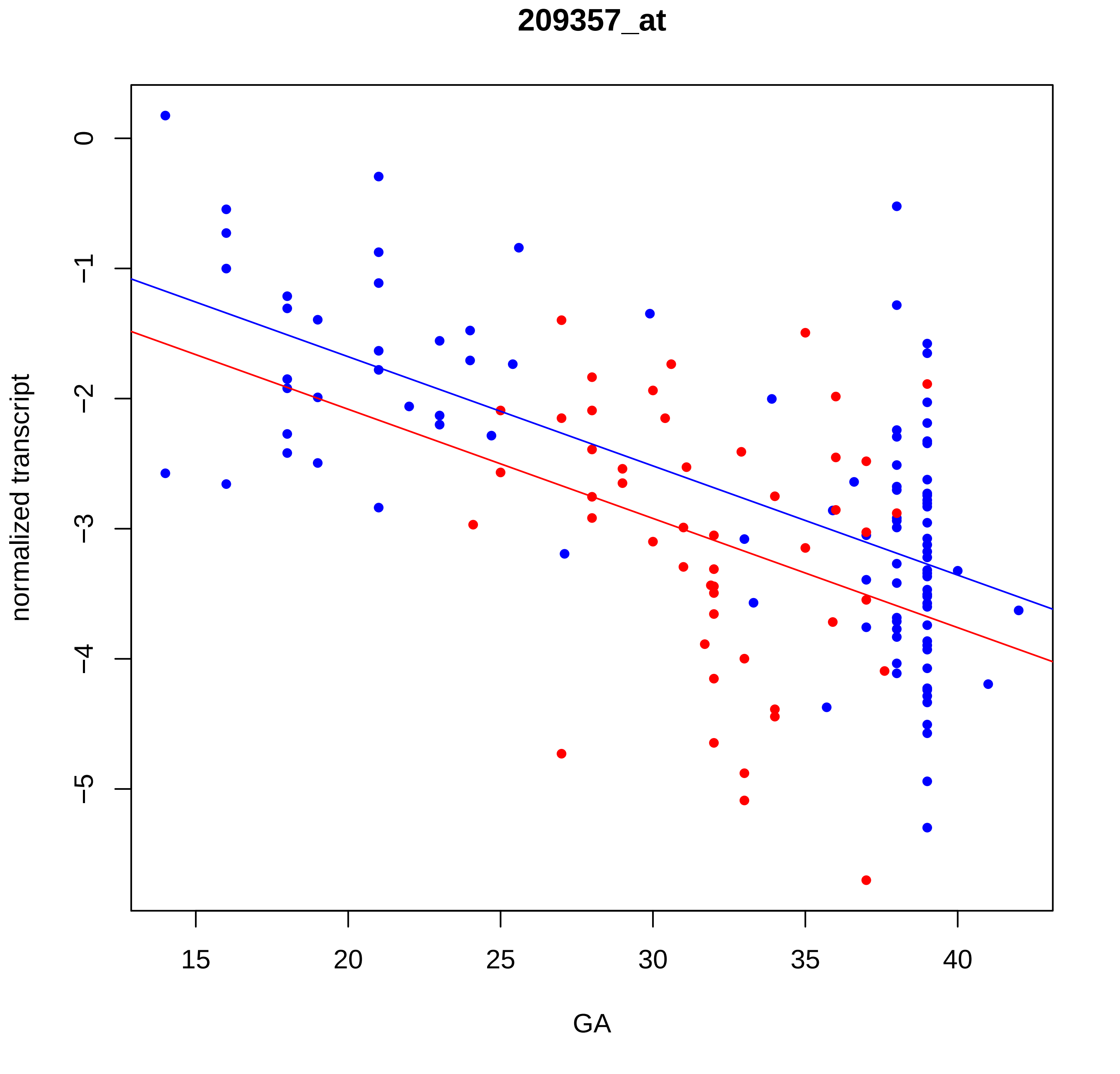

209357_at
normalized transcript
0
−1
−2
−3
−4
−5
15
20
25
30
35
40
GA

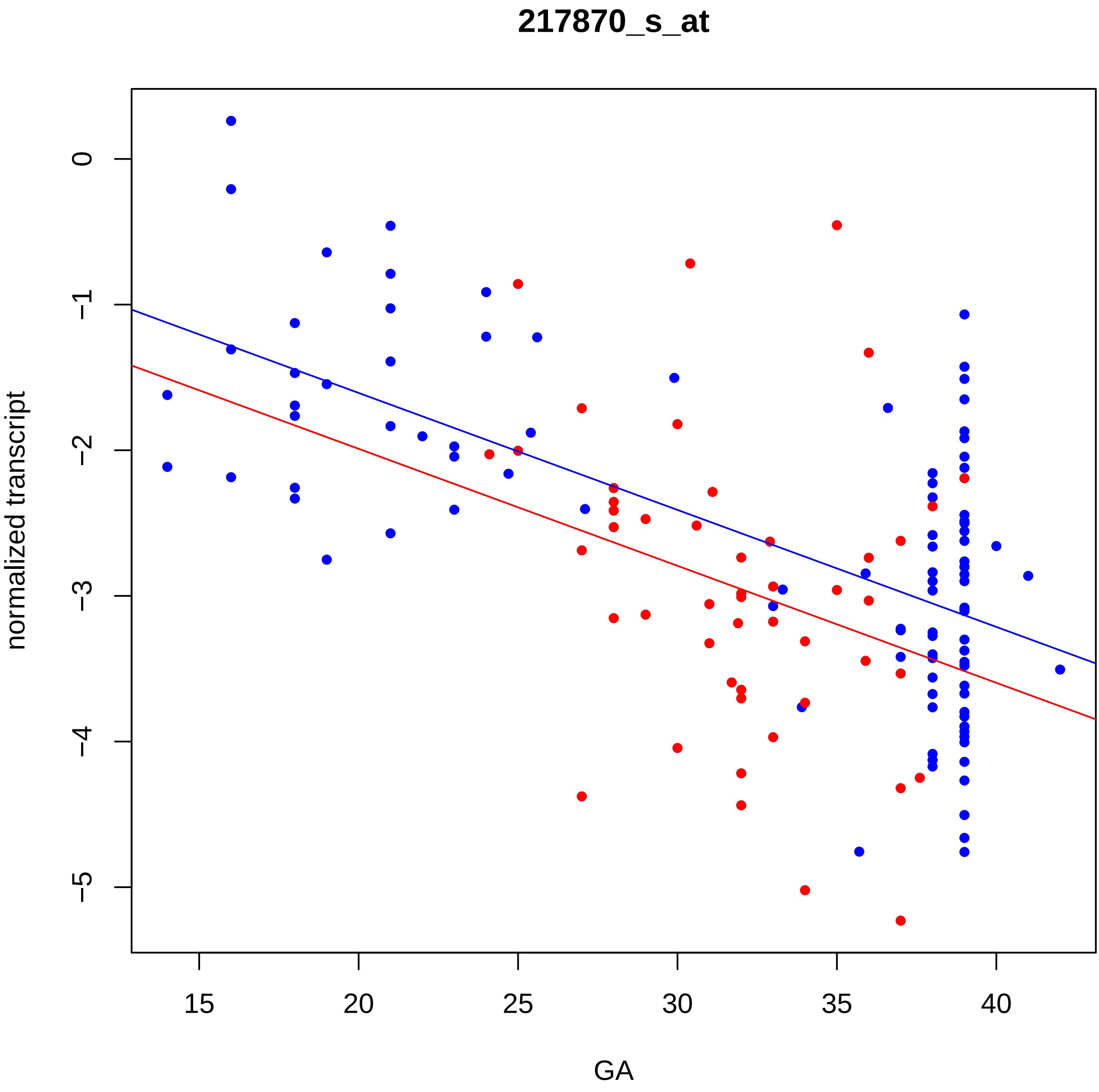

217870_s_at
normalized transcript
GA
0
−1
−2
−3
−4
−5
15
20
25
30
35
40

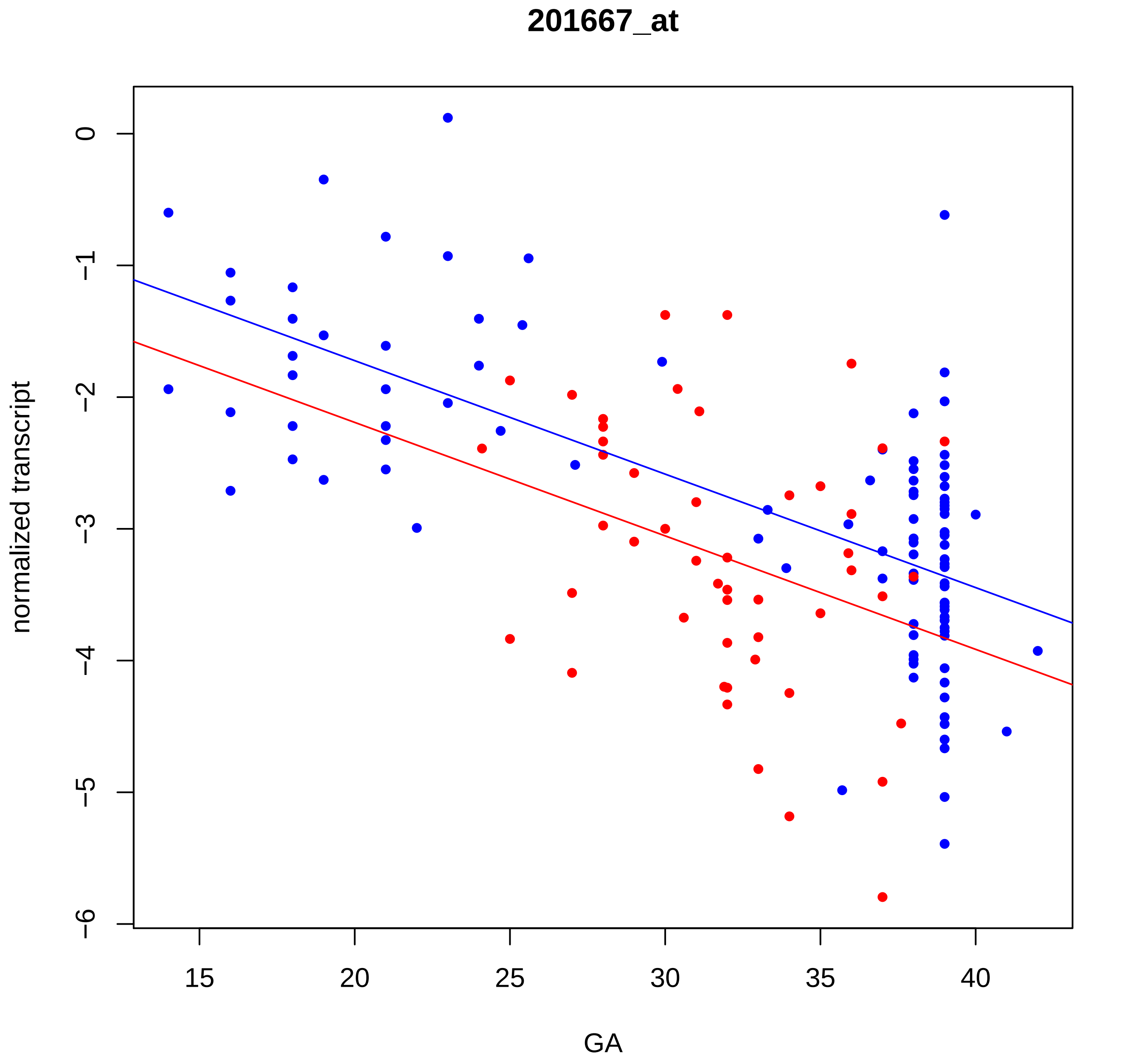

201667_at
normalized transcript
0
−1
−2
−3
−4
−5
−6
15
20
25
30
35
40
GA

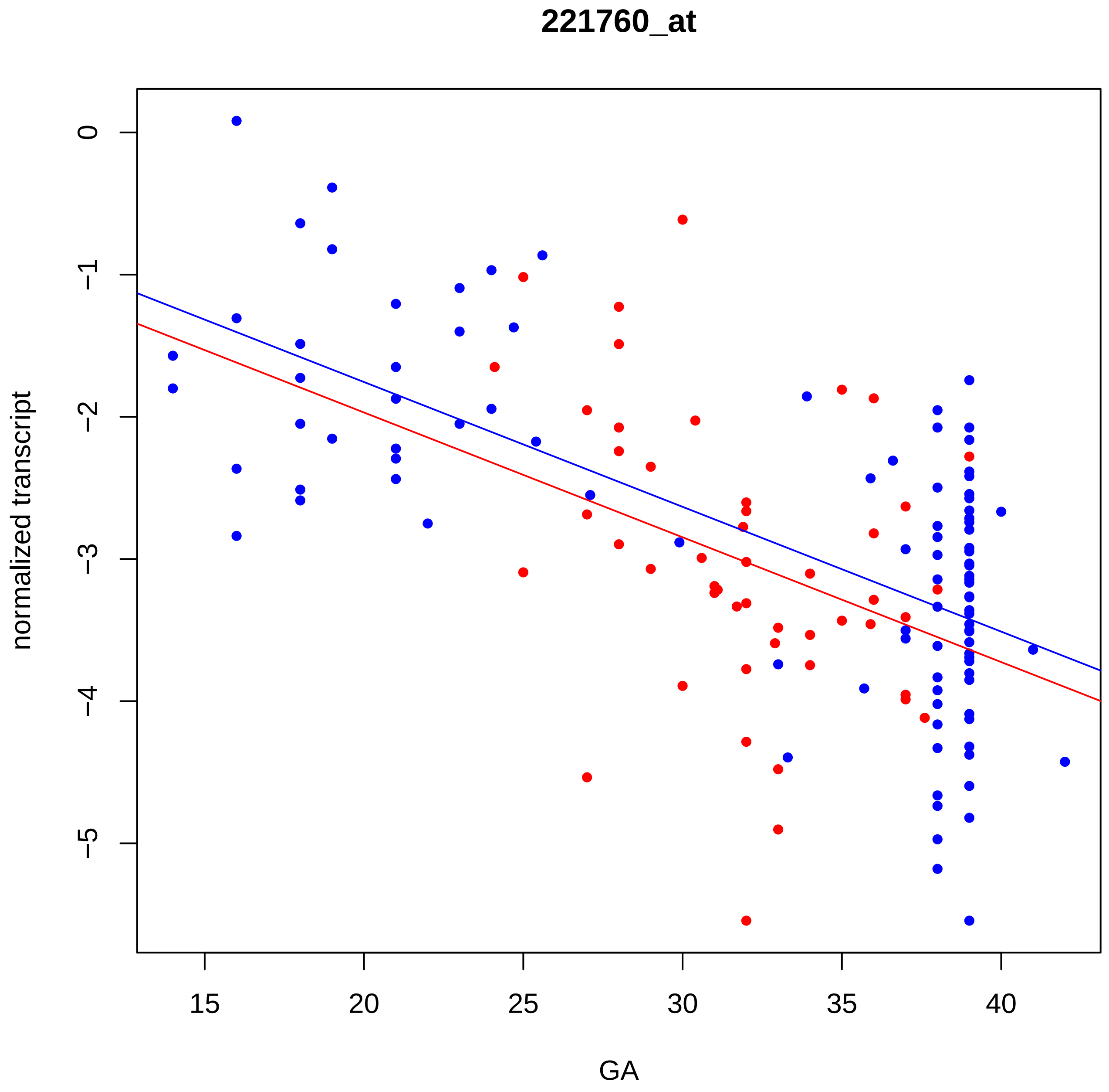
221760_at
normalized transcript
0
−1
−2
−3
−4
−5
15
20
25
30
35
40
GA

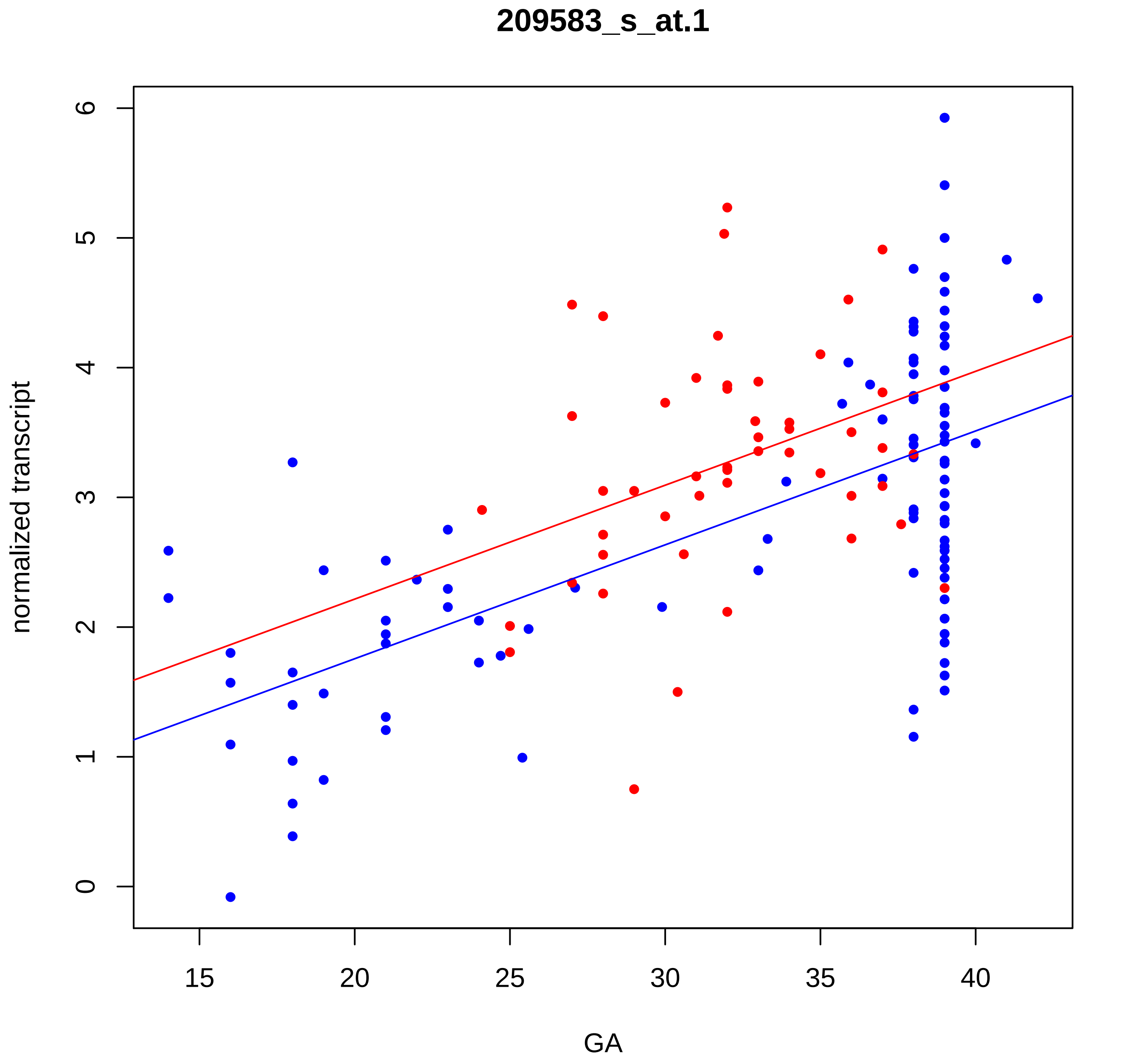

209583_s_at.1
normalized transcript
0
1
2
3
4
5
6
15
20
25
30
35
40
GA

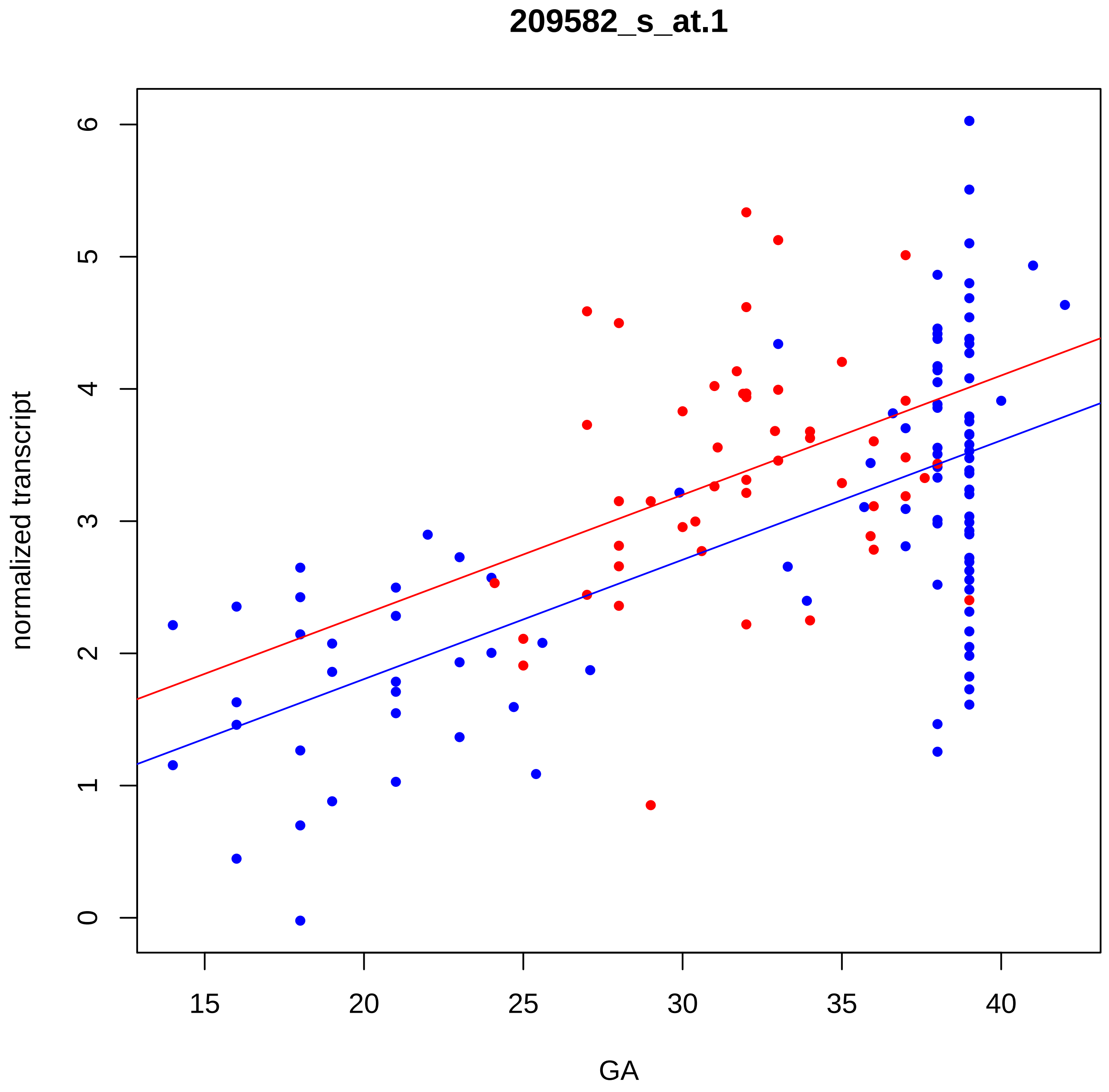

209582_s_at.1
normalized transcript
0
1
2
3
4
5
6
15
20
25
30
35
40
GA

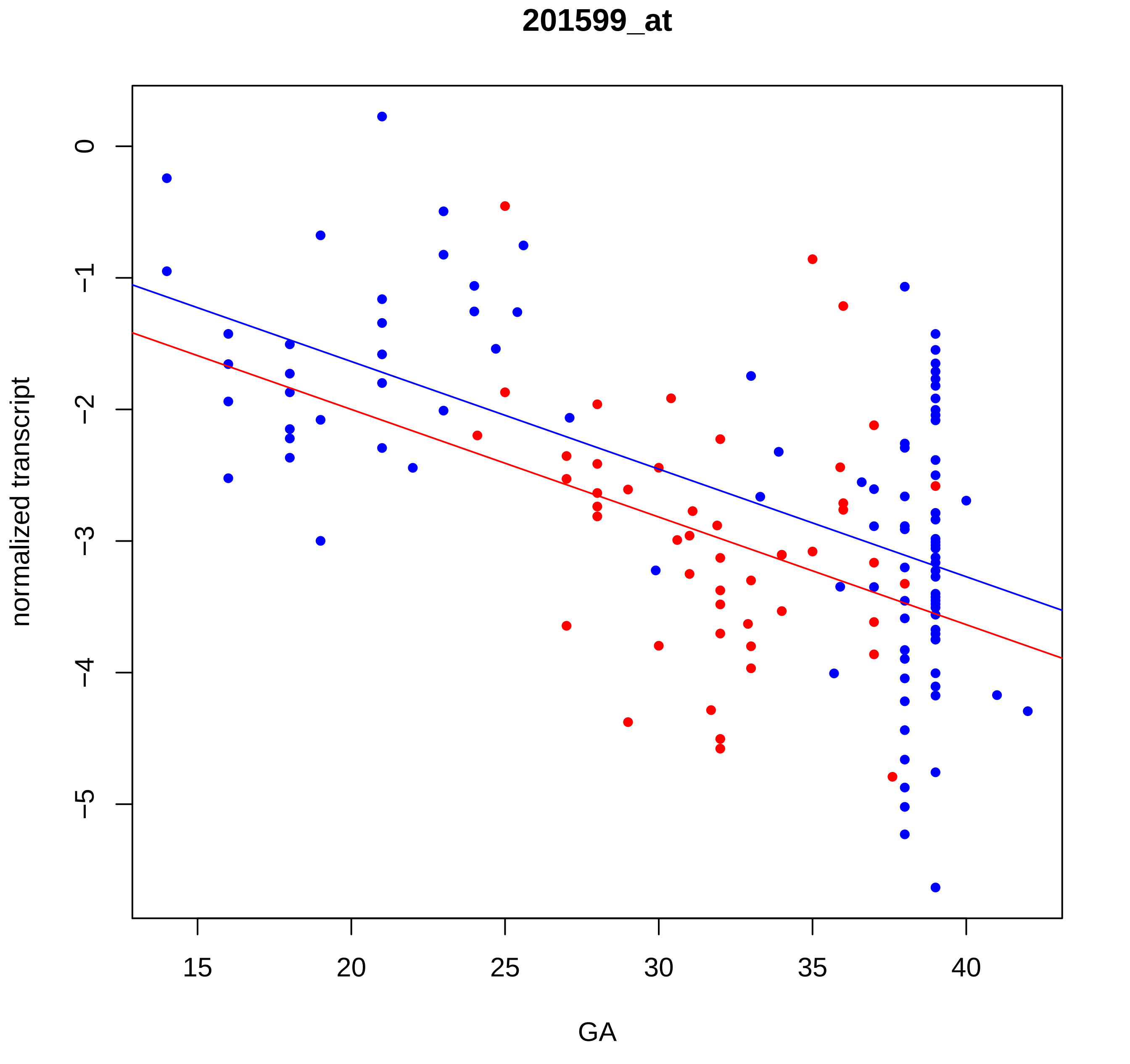

201599_at
0
−1
−2
−3
−4
−5
normalized transcript
15
20
25
30
35
40
GA

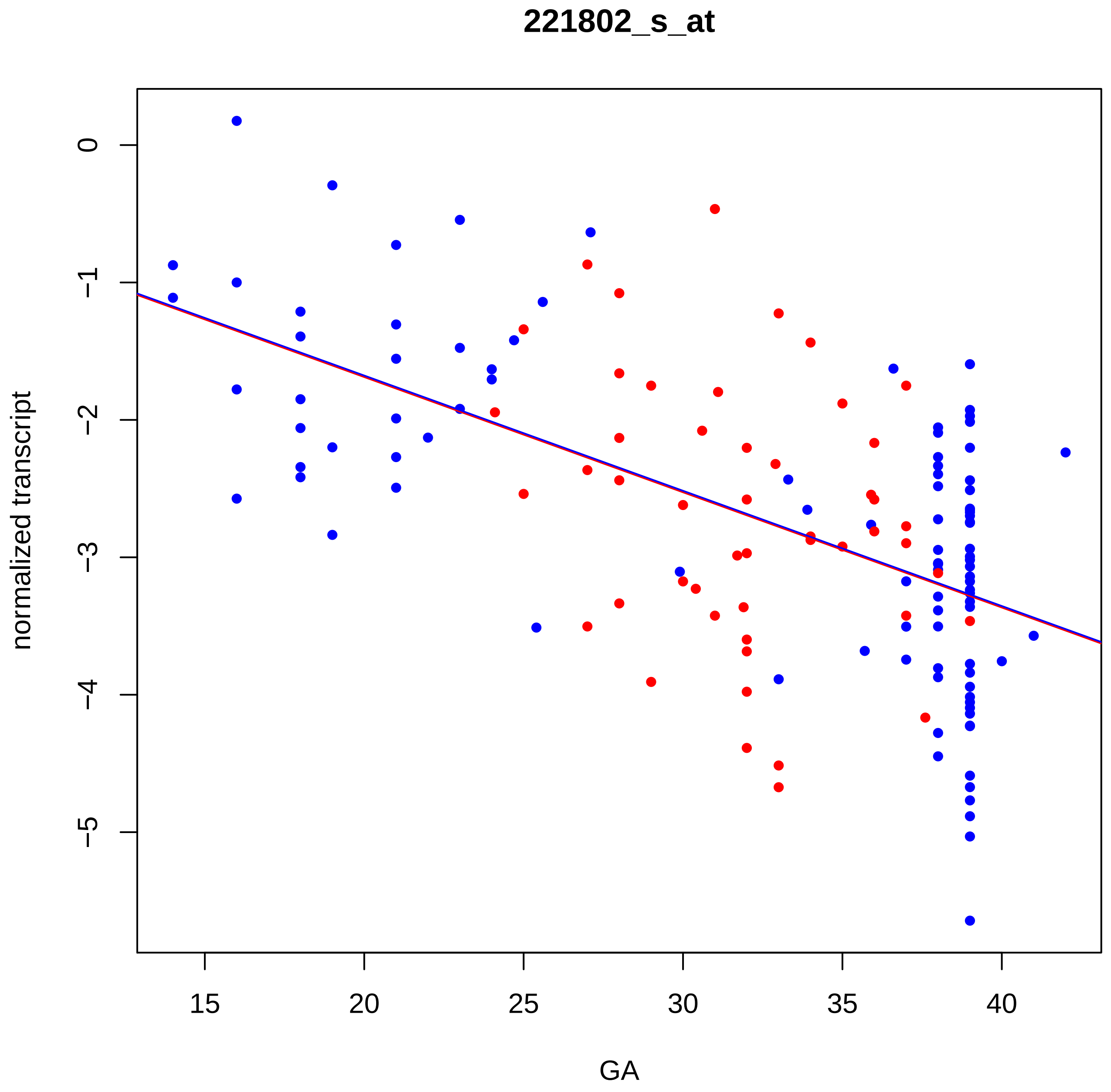

221802_s_at
normalized transcript
GA
0
−1
−2
−3
−4
−5
15
20
25
30
35
40

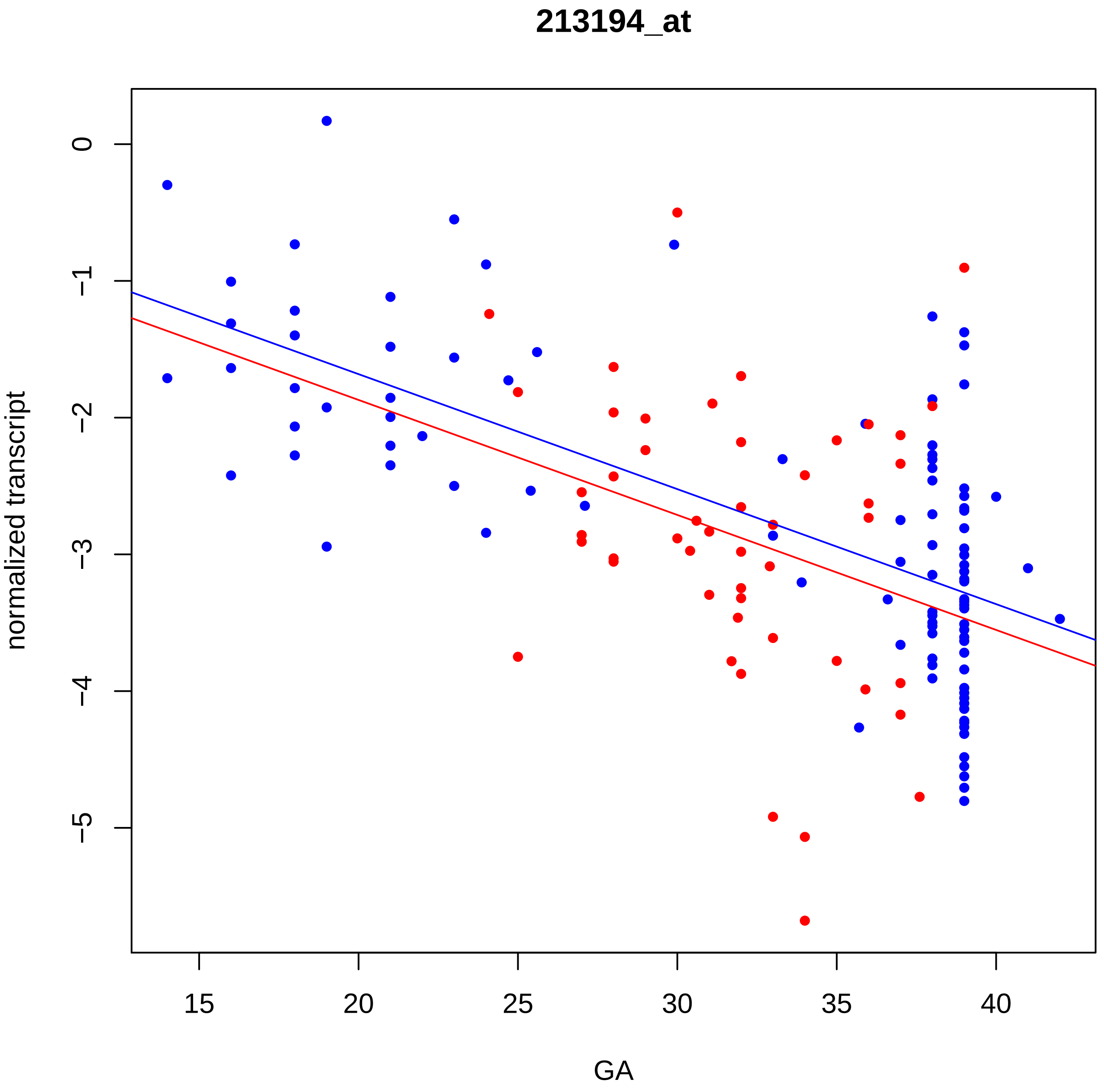
213194_at
normalized transcript
GA
0
−1
−2
−3
−4
−5
15
20
25
30
35
40

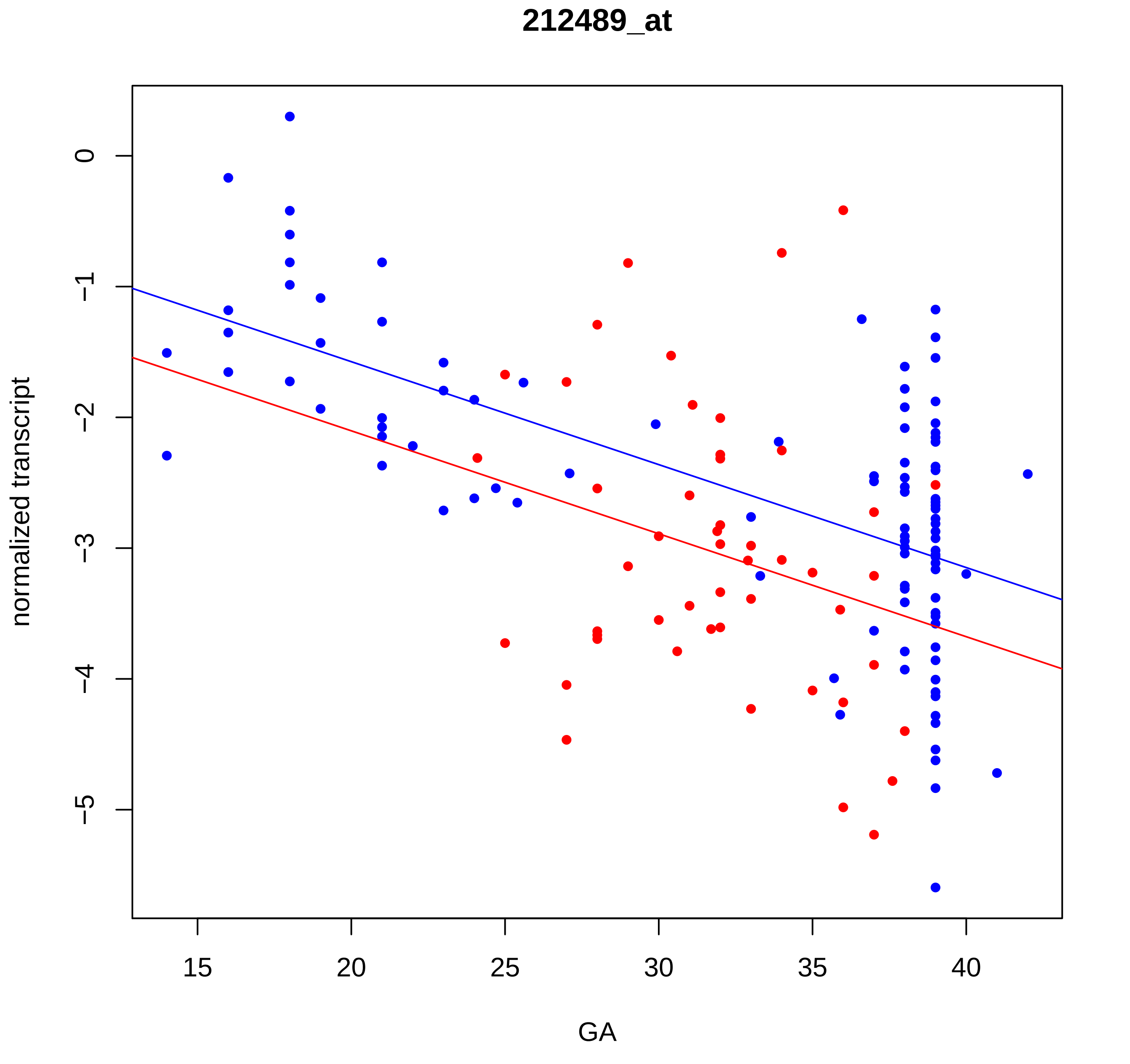

212489_at
0
−1
−2
−3
−4
−5
normalized transcript
15
20
25
30
35
40
GA

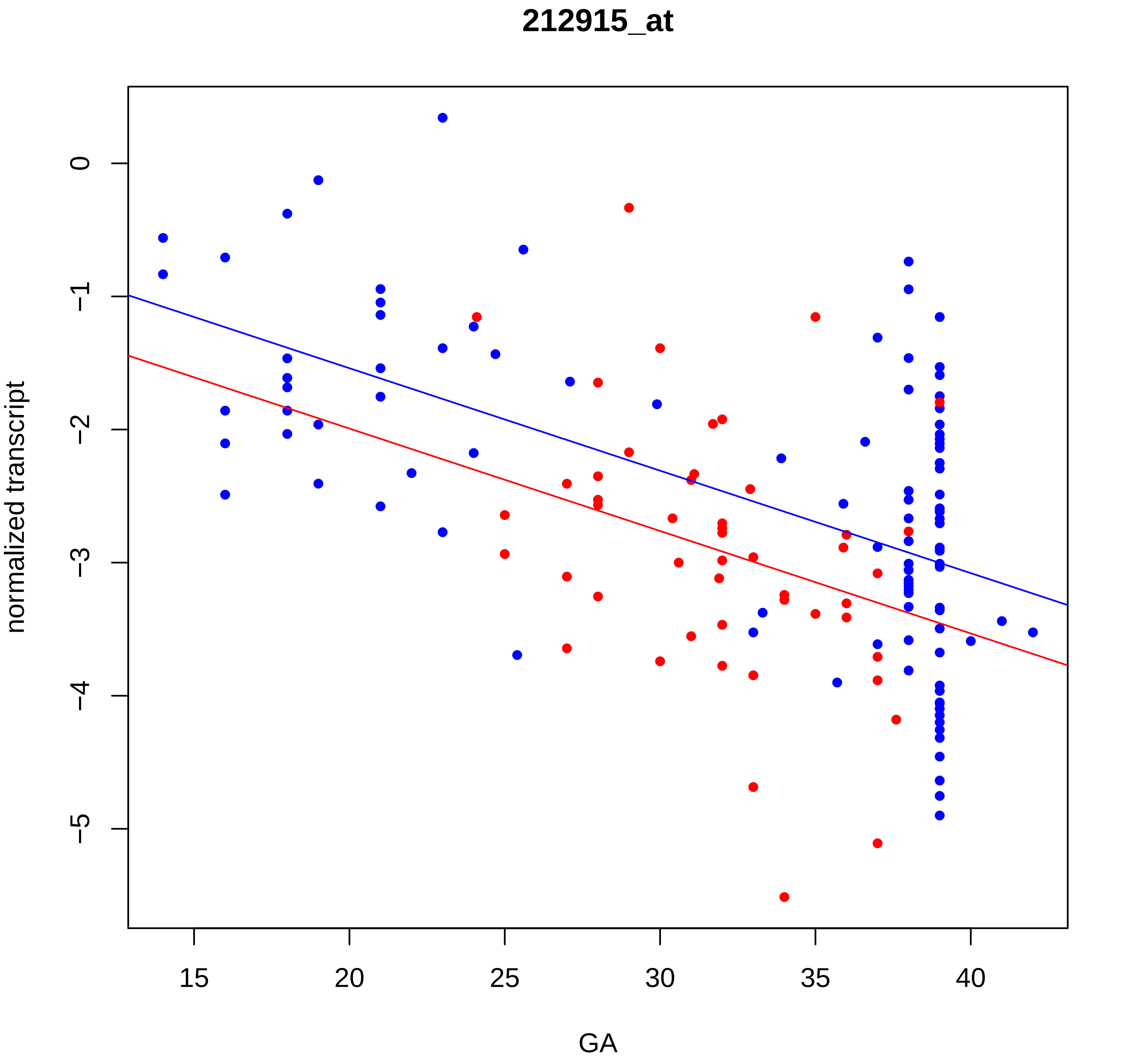
212915_at
normalized transcript
GA
0
−1
−2
−3
−4
−5
15
20
25
30
35
40

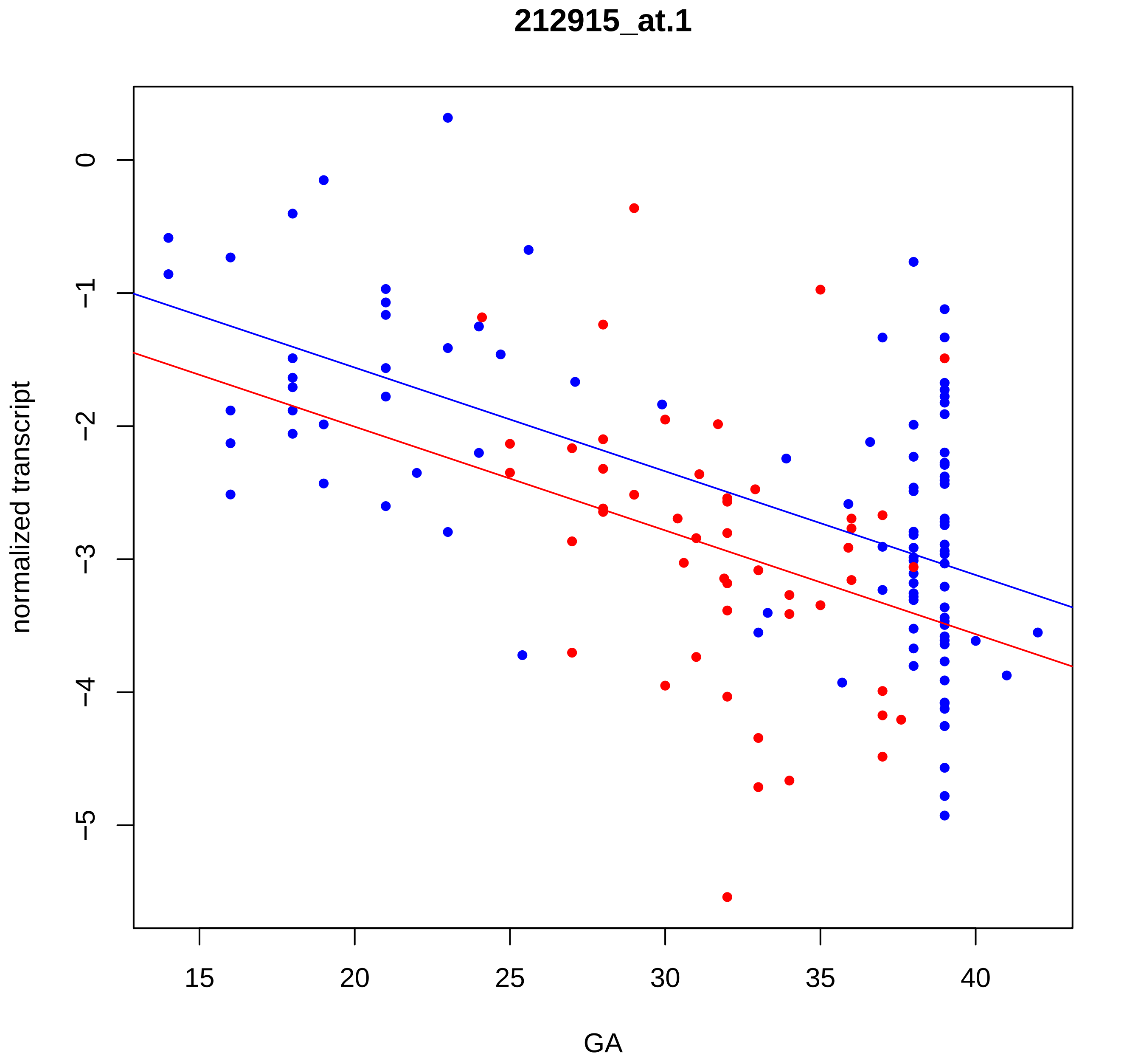

212915_at.1
0
−1
−2
−3
−4
−5
normalized transcript
15
20
25
30
35
40
GA

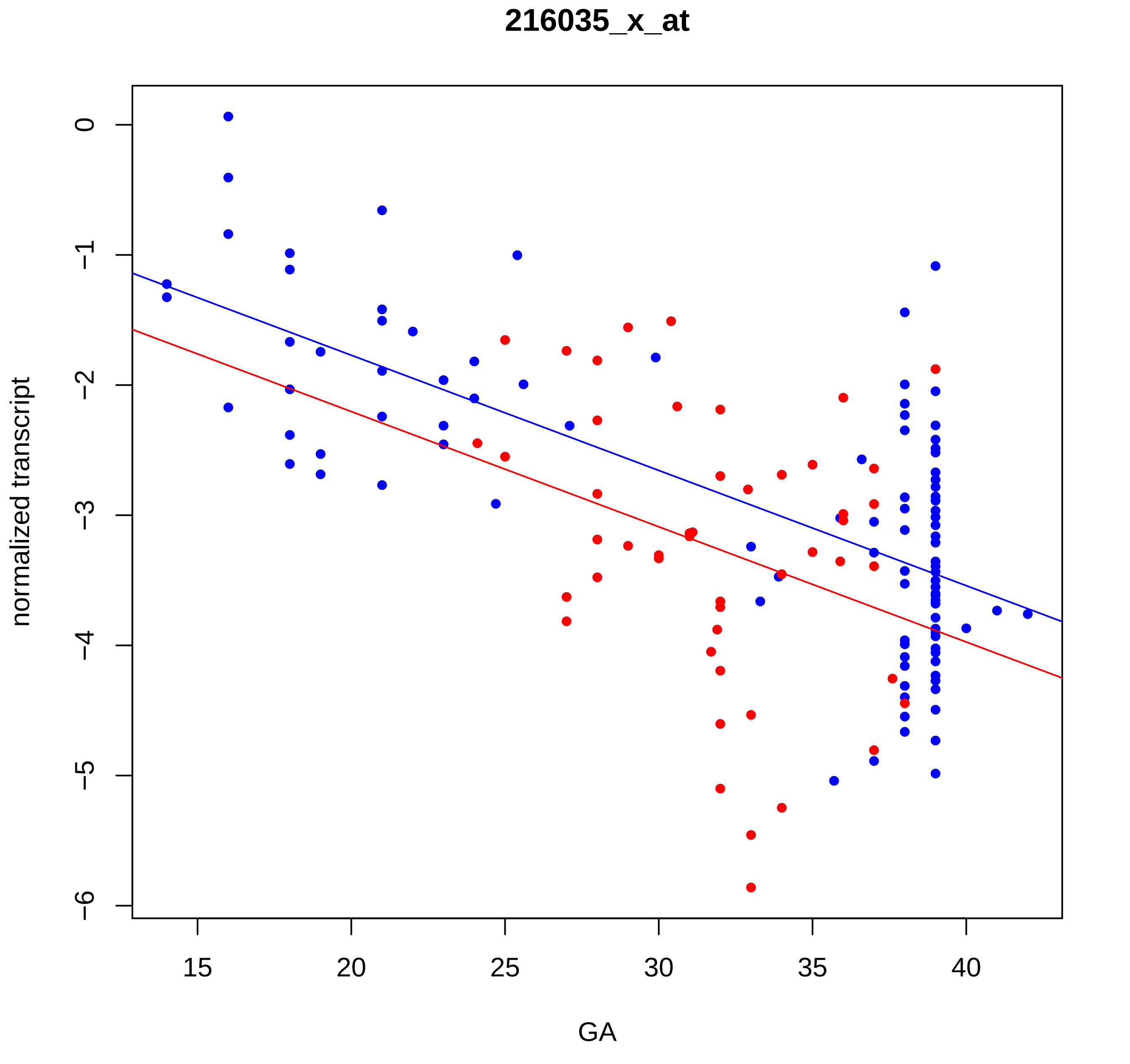
216035_x_at
0
−1
−2
−3
−4
−5
−6
normalized transcript
15
20
25
30
35
40
GA

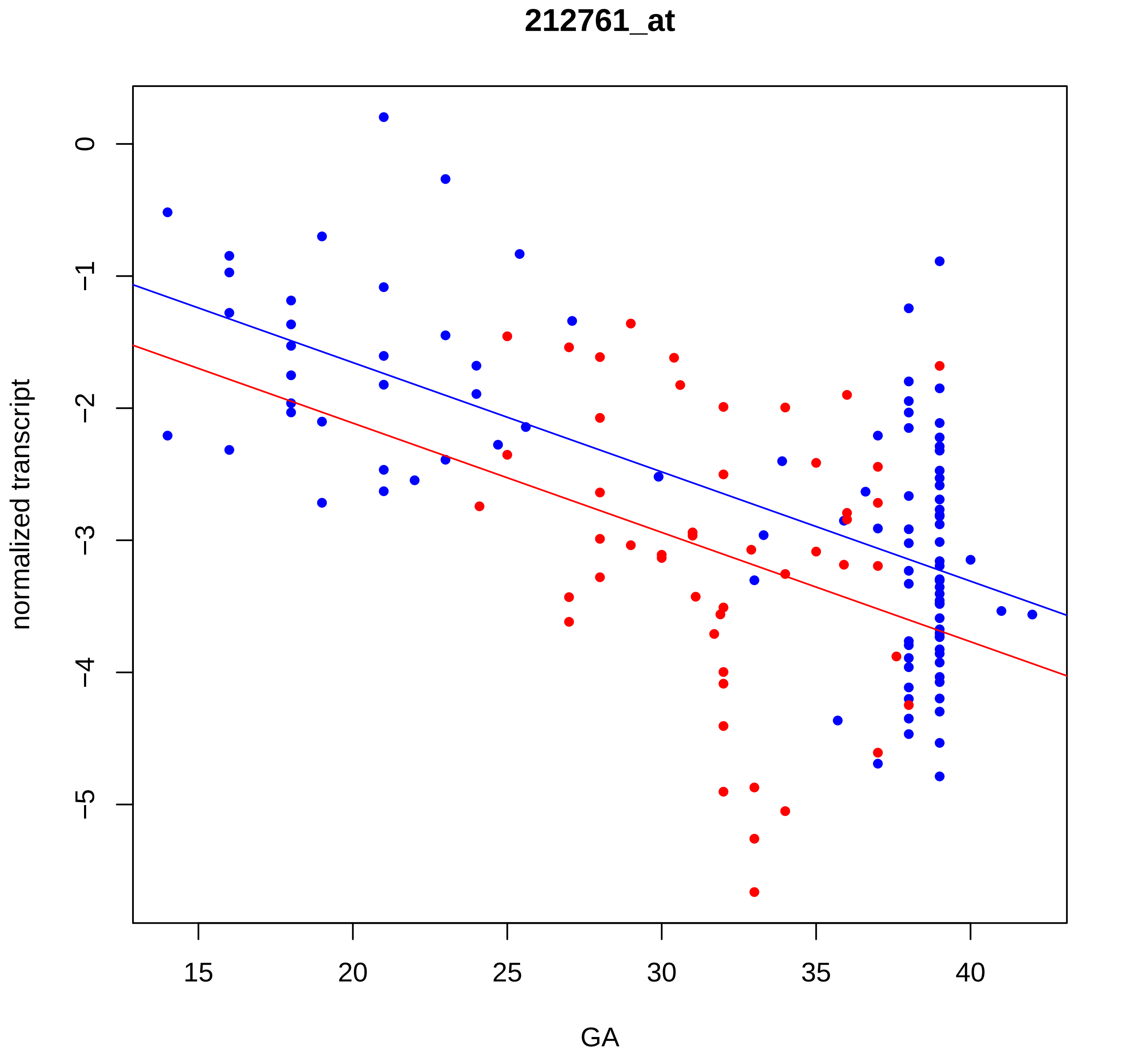

212761_at
0
−1
−2
−3
−4
−5
normalized transcript
15
20
25
30
35
40
GA

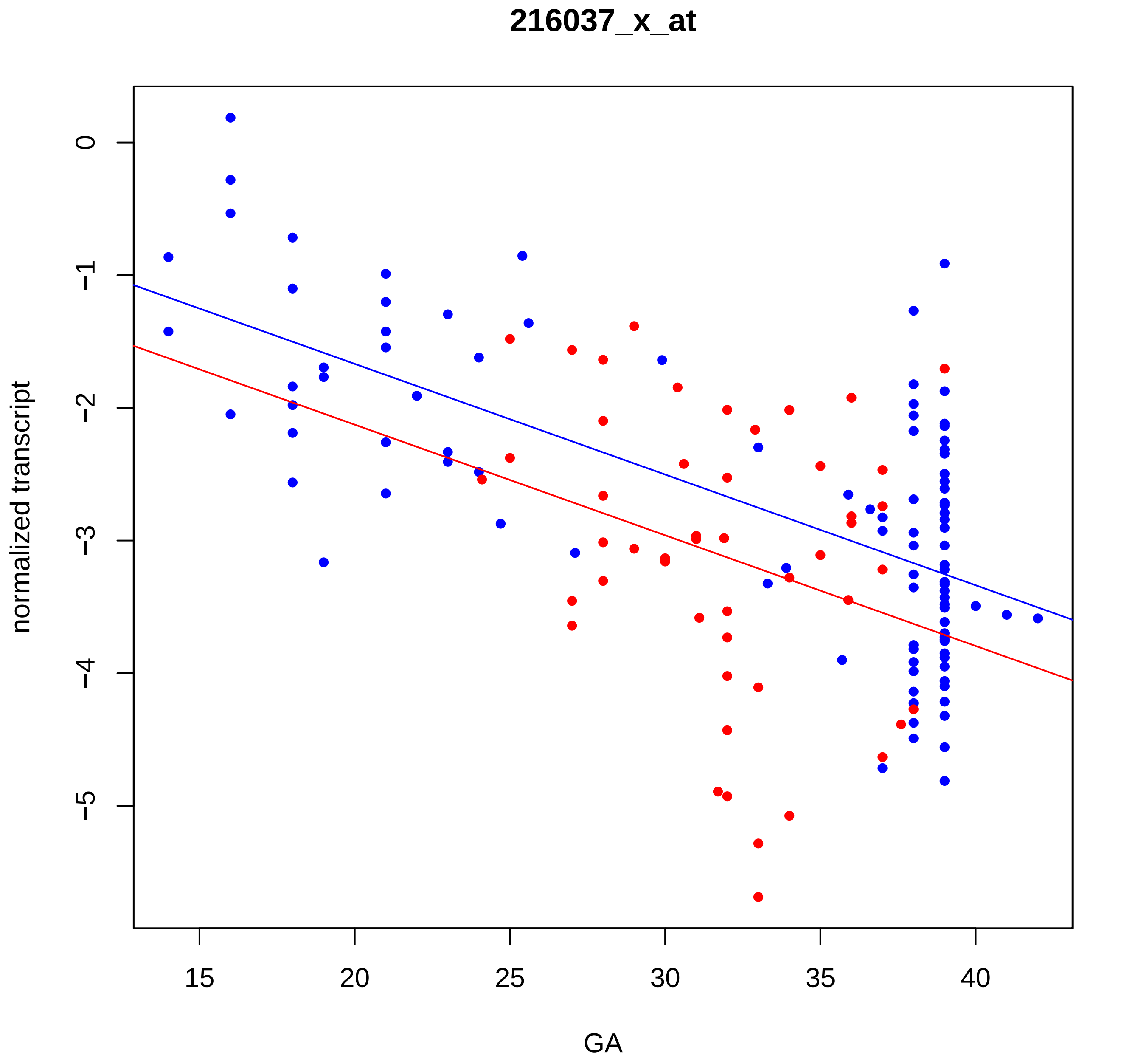

216037_x_at
normalized transcript
GA
0
−1
−2
−3
−4
−5
15
20
25
30
35
40

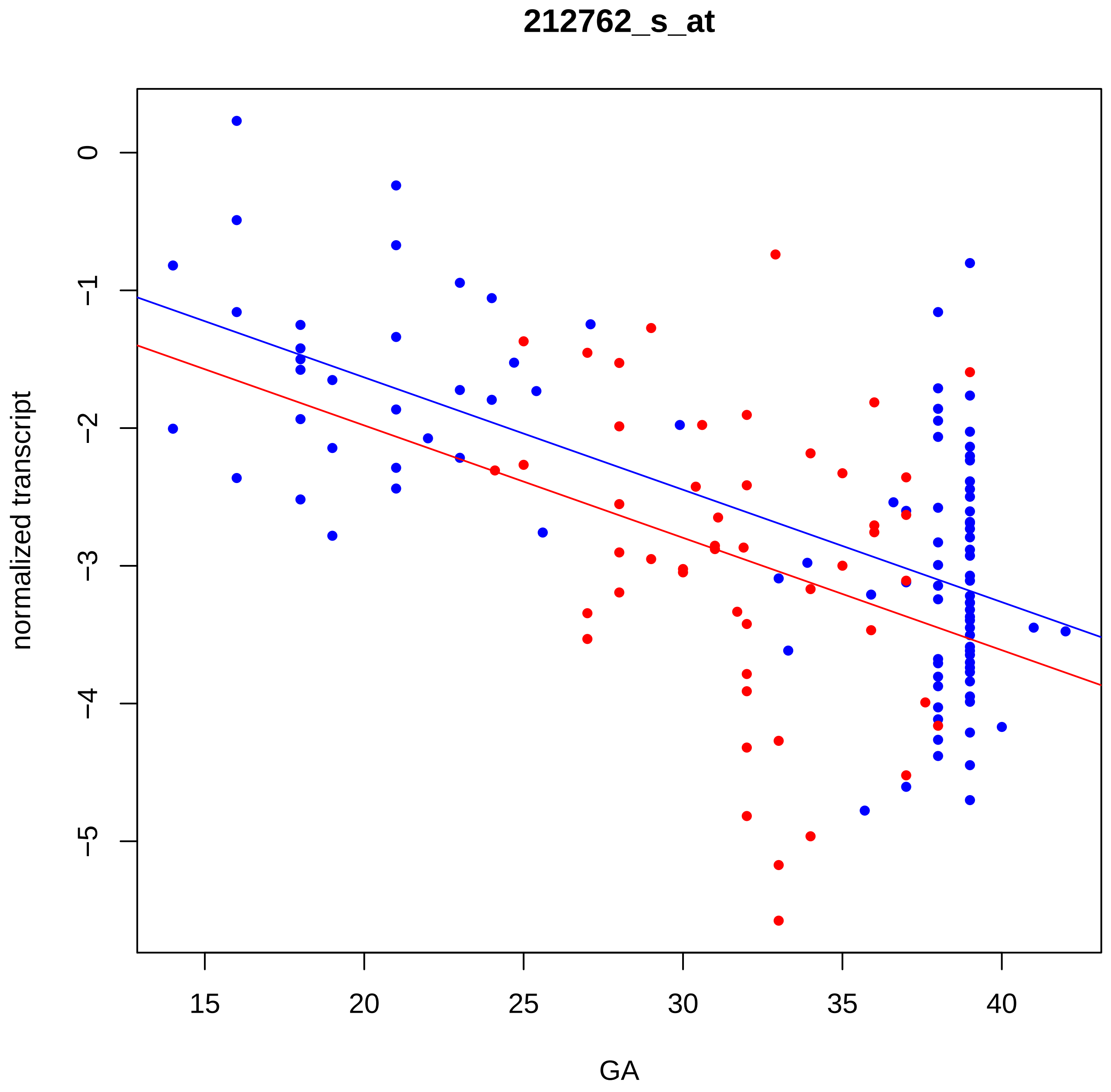

212762_s_at
normalized transcript
GA
0
−1
−2
−3
−4
−5
15
20
25
30
35
40

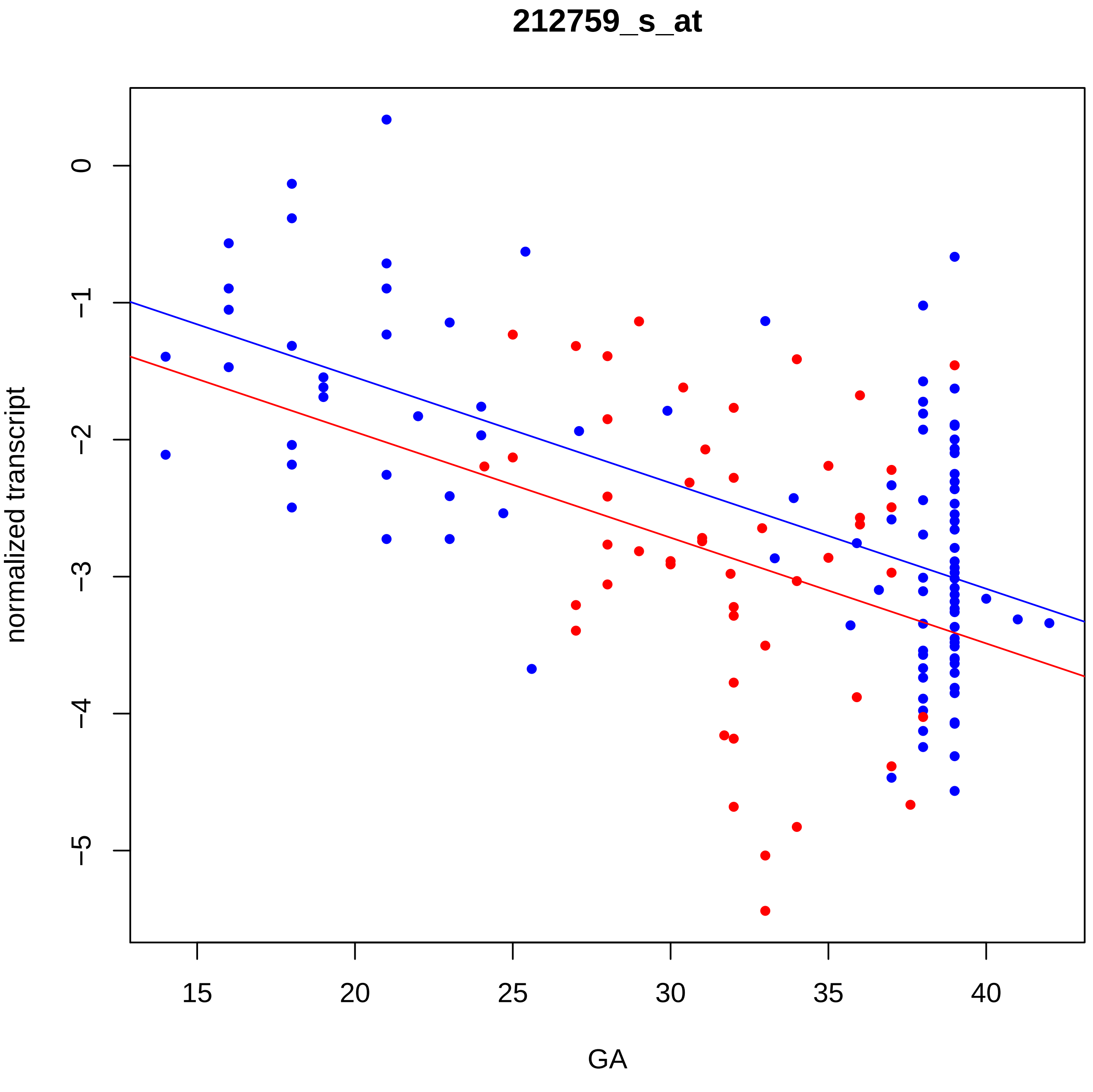

212759_s_at
normalized transcript
GA
0
−1
−2
−3
−4
−5
15
20
25
30
35
40

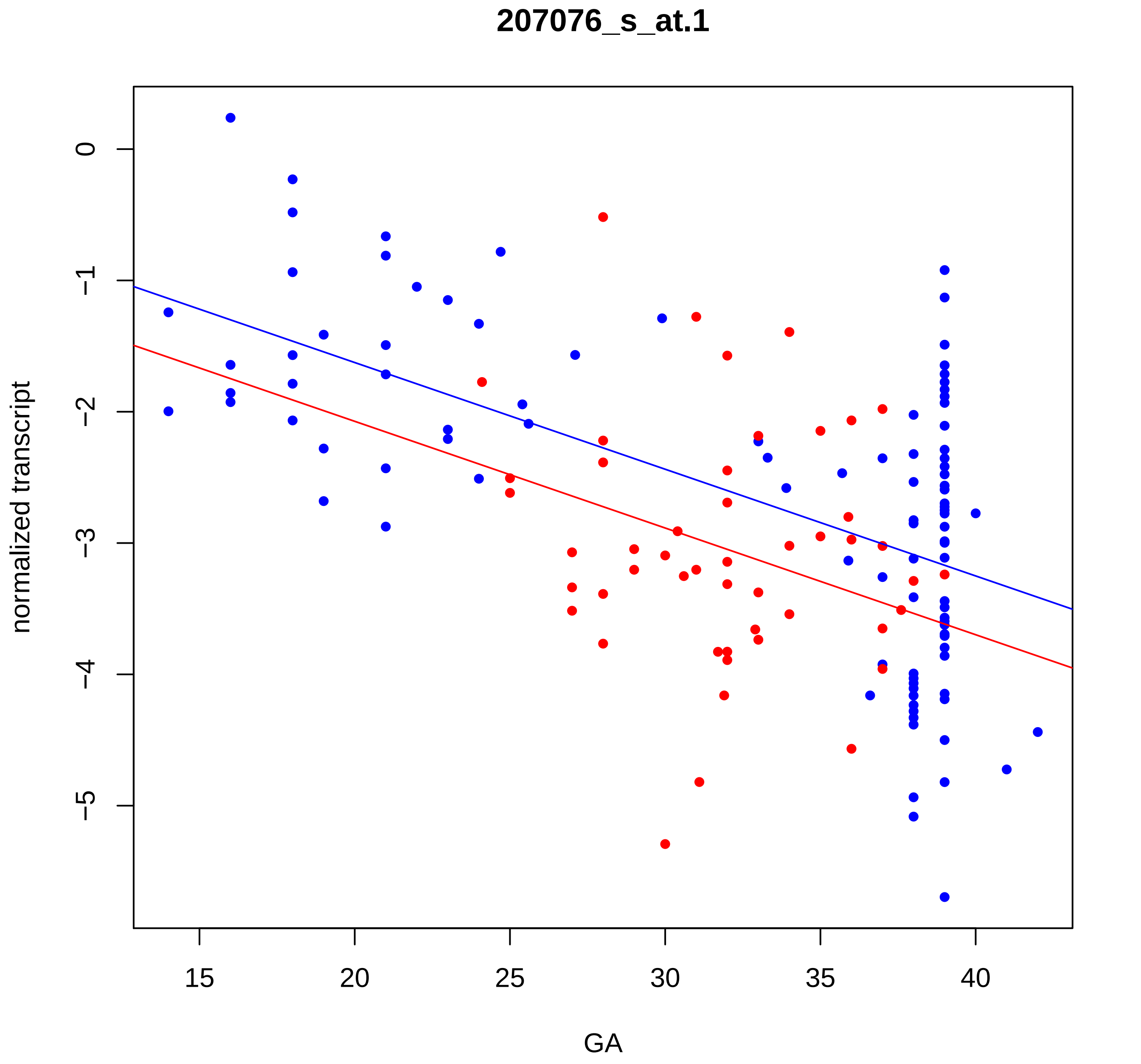

207076_s_at.1
normalized transcript
0
−1
−2
−3
−4
−5
15
20
25
30
35
40
GA

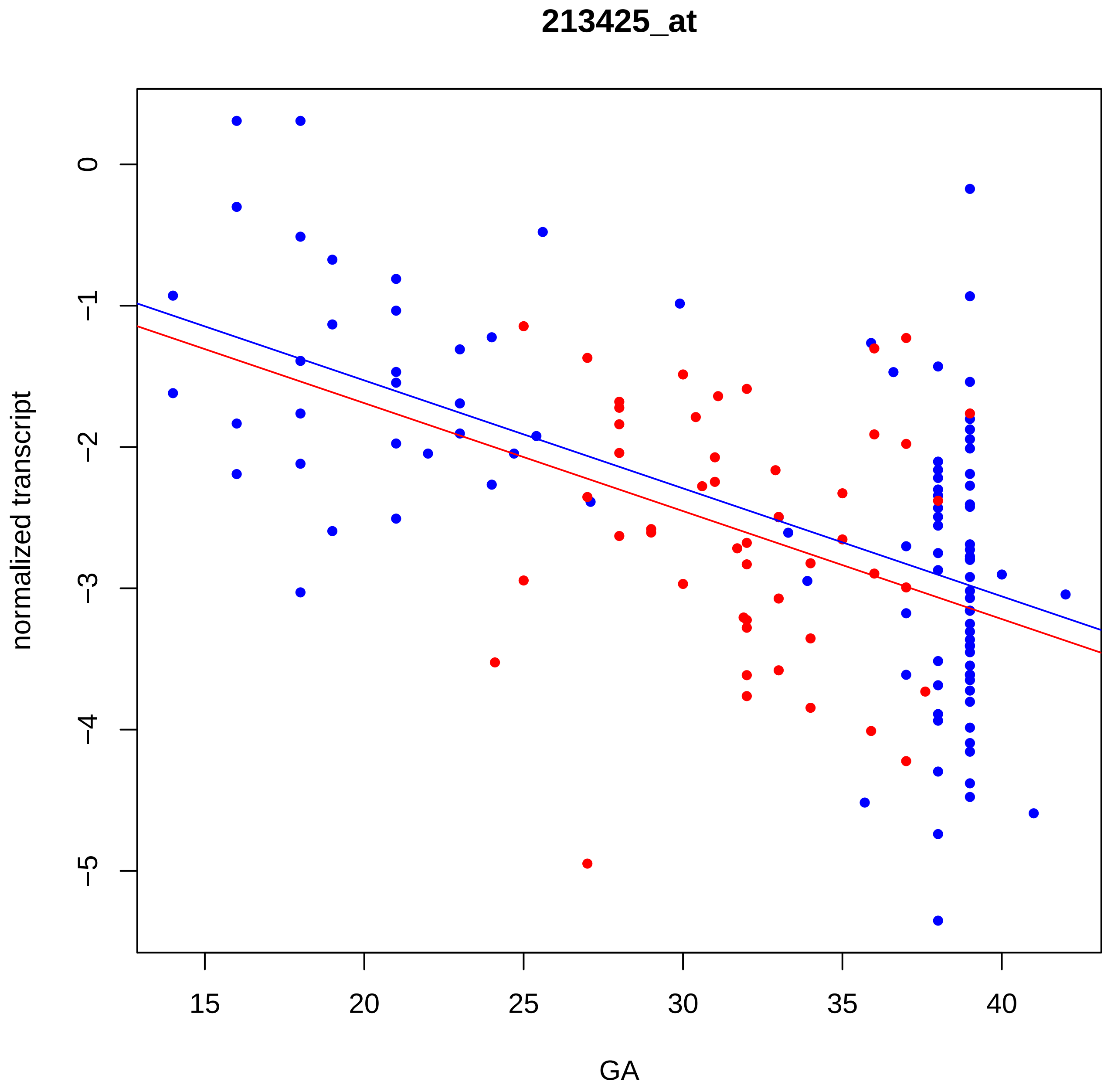

213425_at
0
−1
−2
−3
−4
−5
normalized transcript
15
20
25
30
35
40
GA

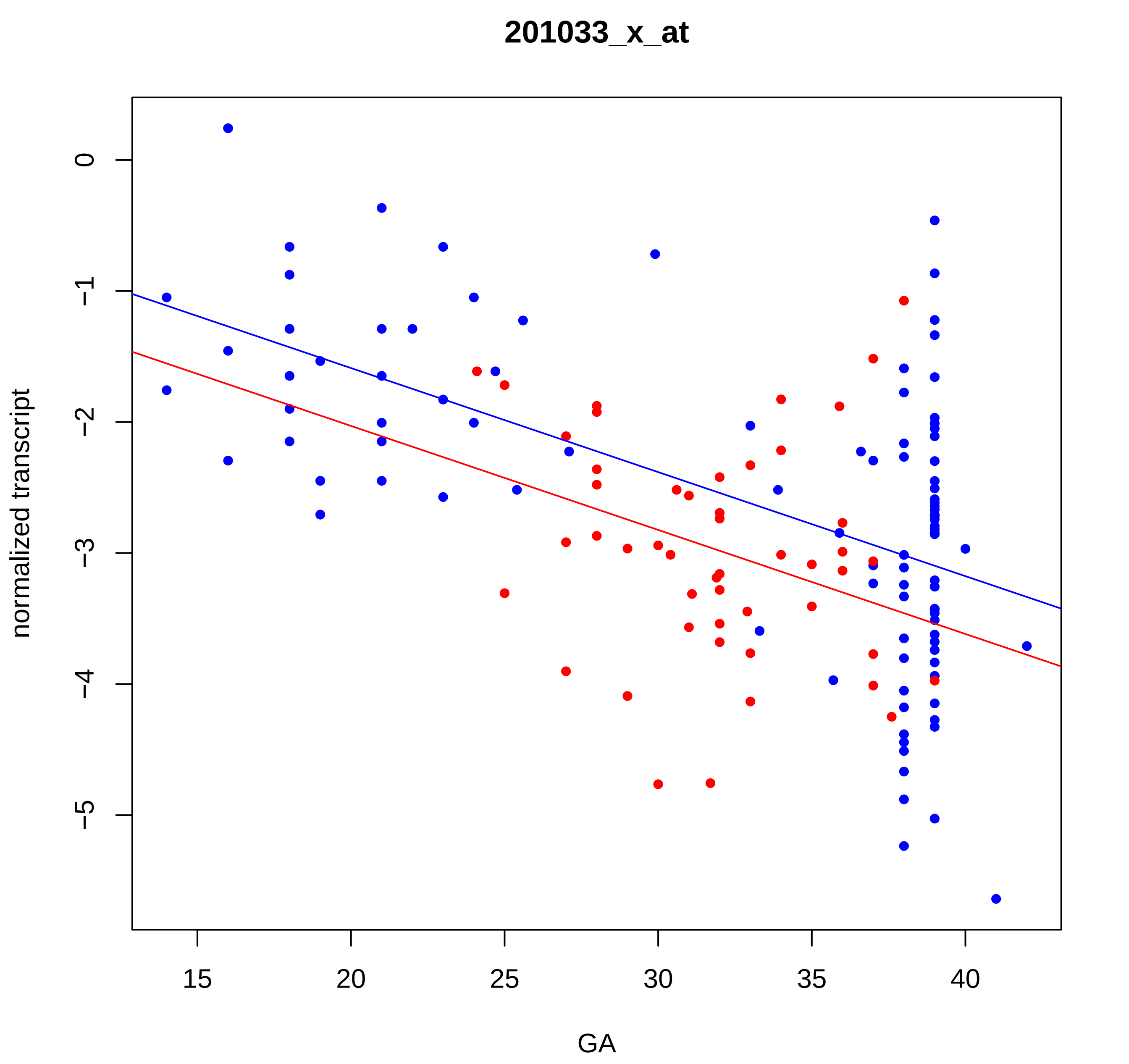

201033_x_at
normalized transcript
GA
0
−1
−2
−3
−4
−5
15
20
25
30
35
40

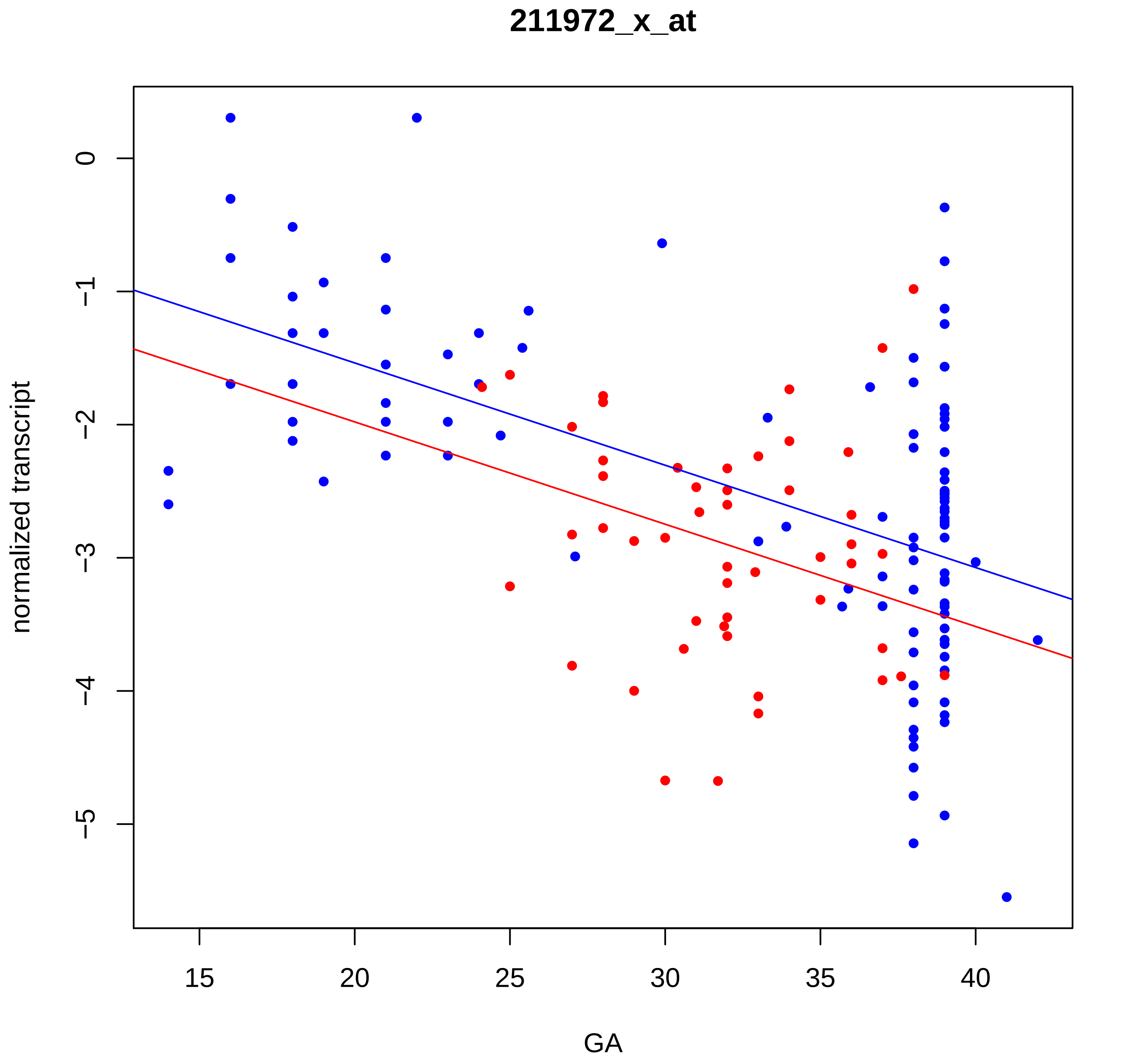

211972_x_at
0
−1
−2
−3
−4
−5
normalized transcript
15
20
25
30
35
40
GA

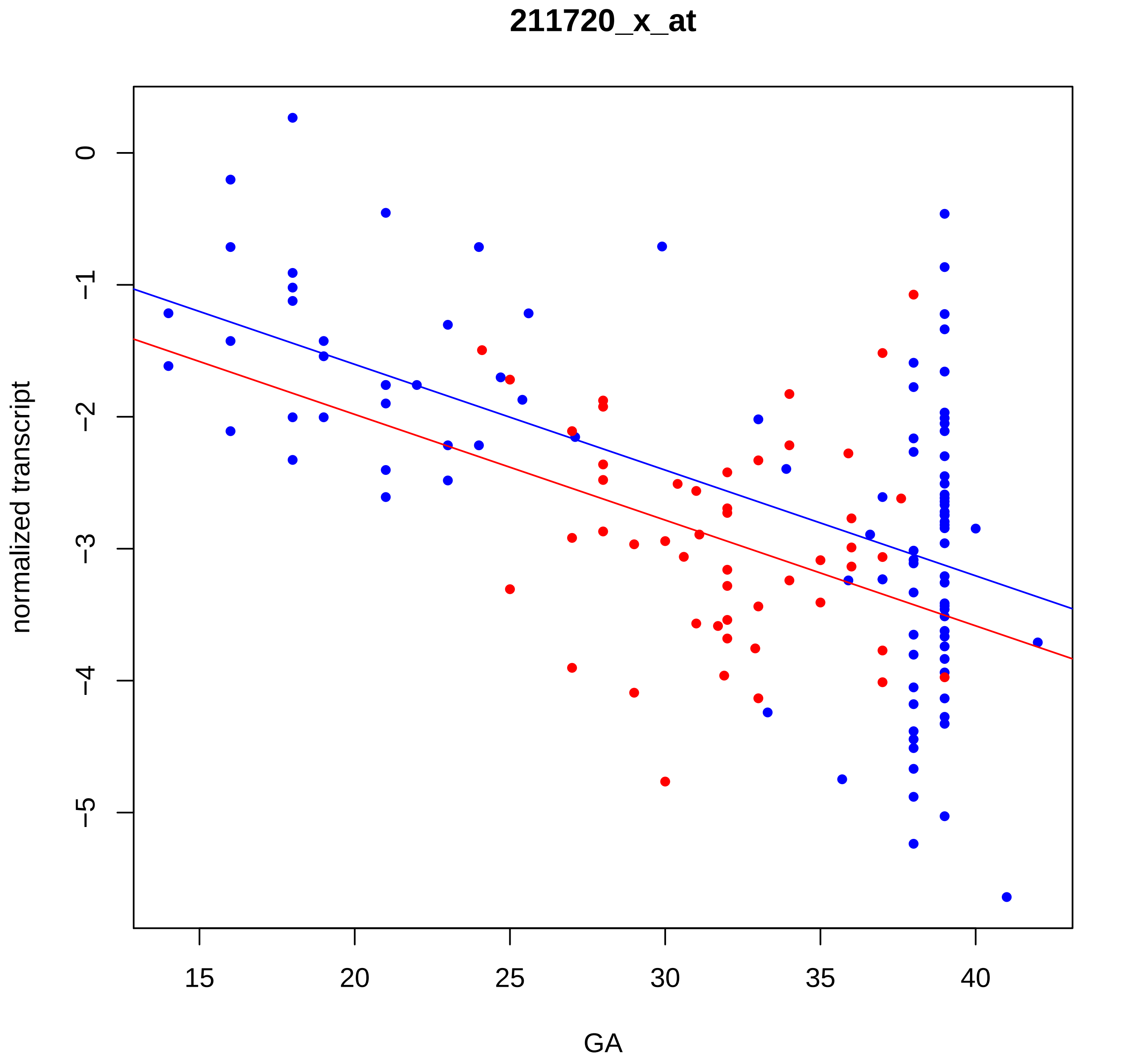

211720_x_at
normalized transcript
GA
0
−1
−2
−3
−4
−5
15
20
25
30
35
40

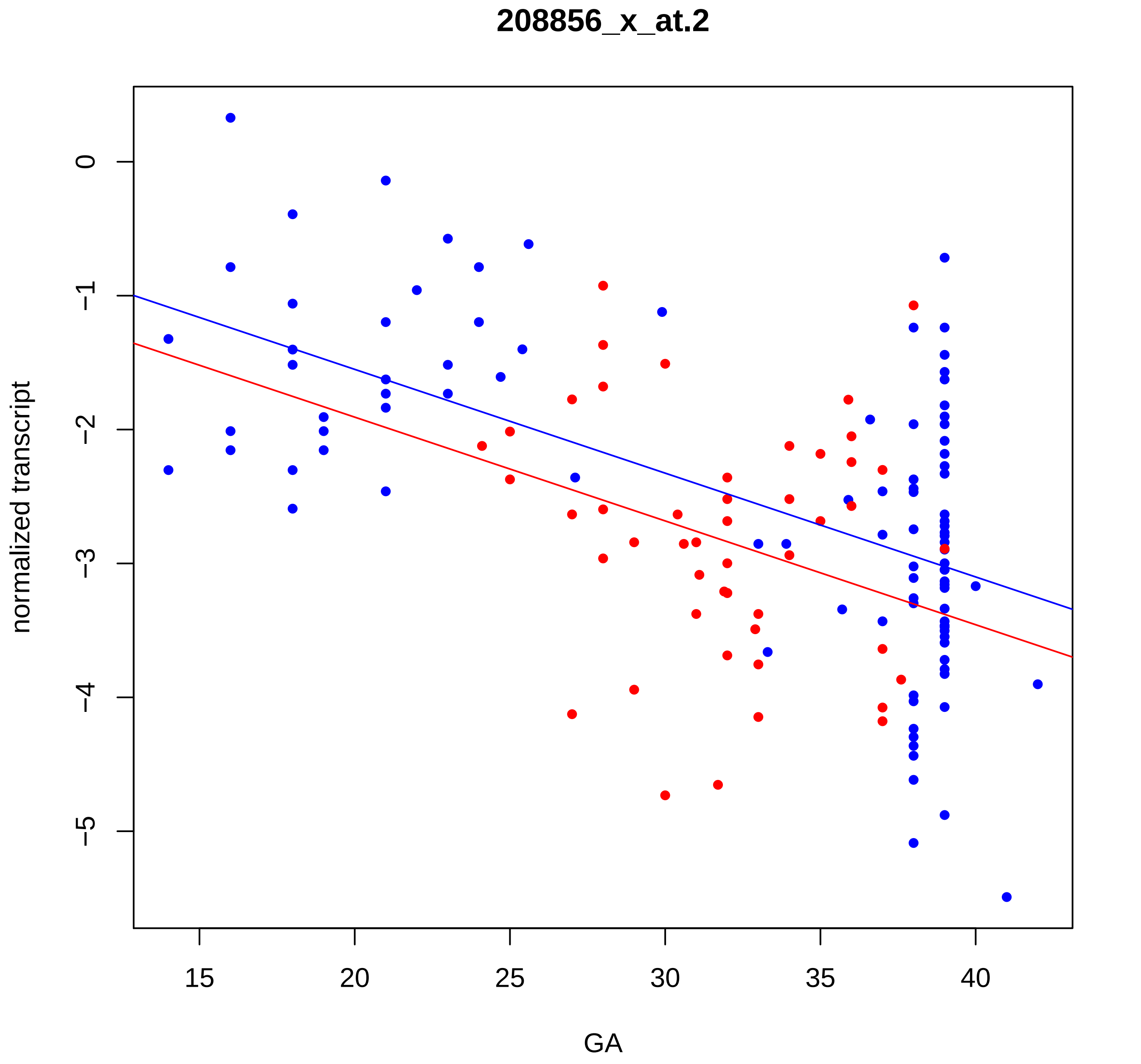

208856_x_at.2
normalized transcript
GA
0
−1
−2
−3
−4
−5
15
20
25
30
35
40

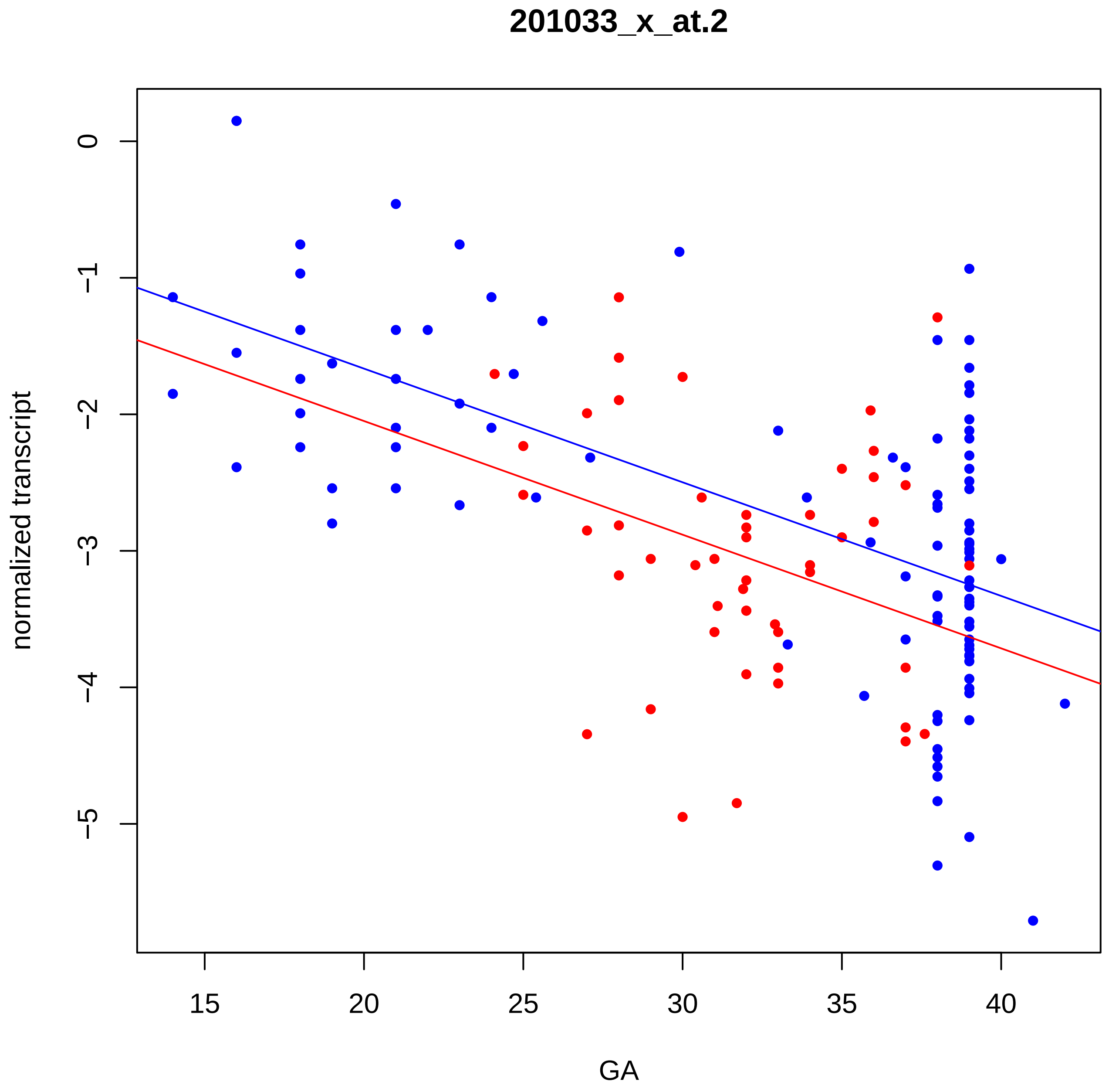
201033_x_at.2
0
−1
−2
−3
−4
−5
normalized transcript
15
20
25
30
35
40
GA

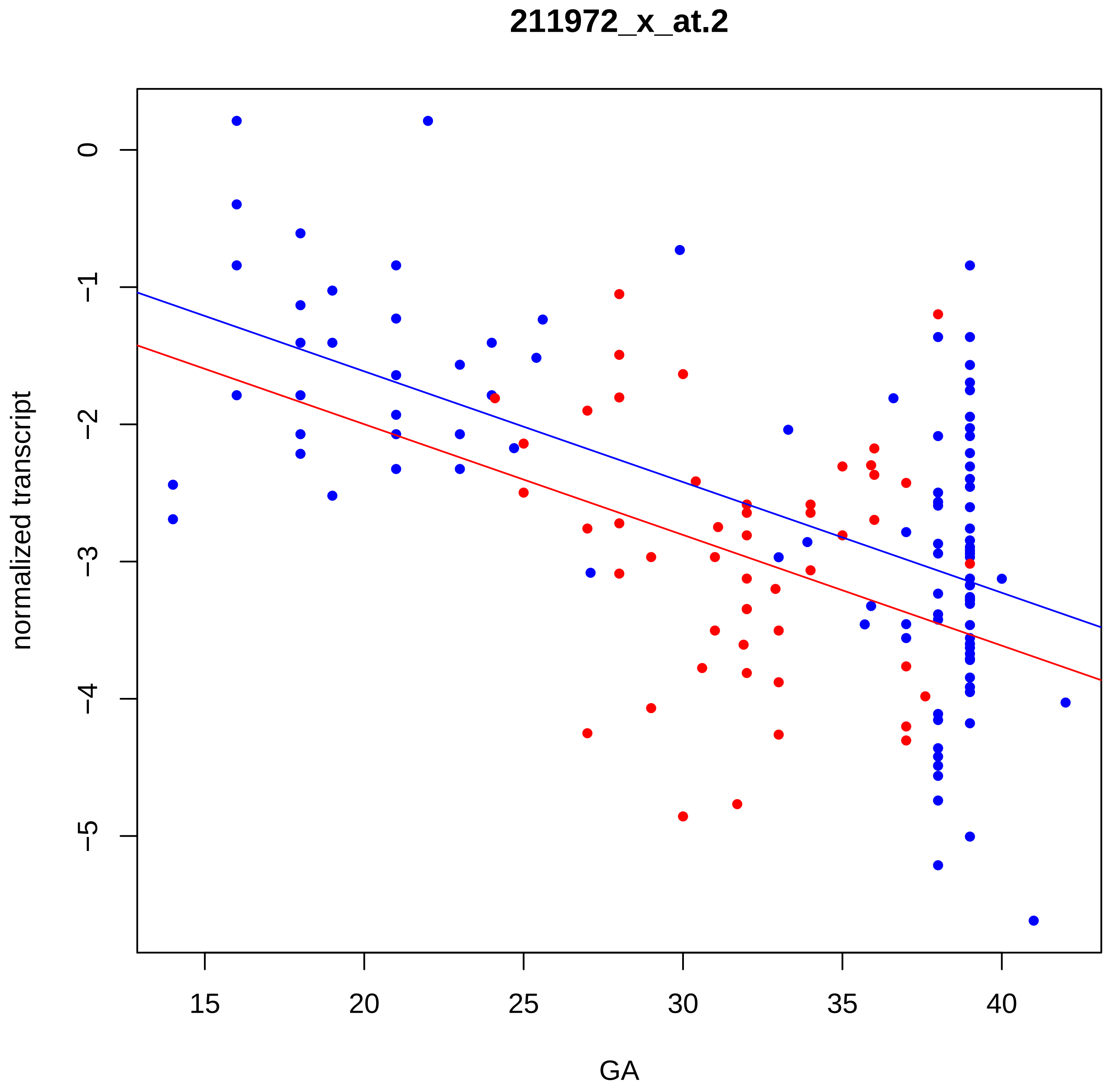
211972_x_at.2
normalized transcript
GA
0
−1
−2
−3
−4
−5
15
20
25
30
35
40

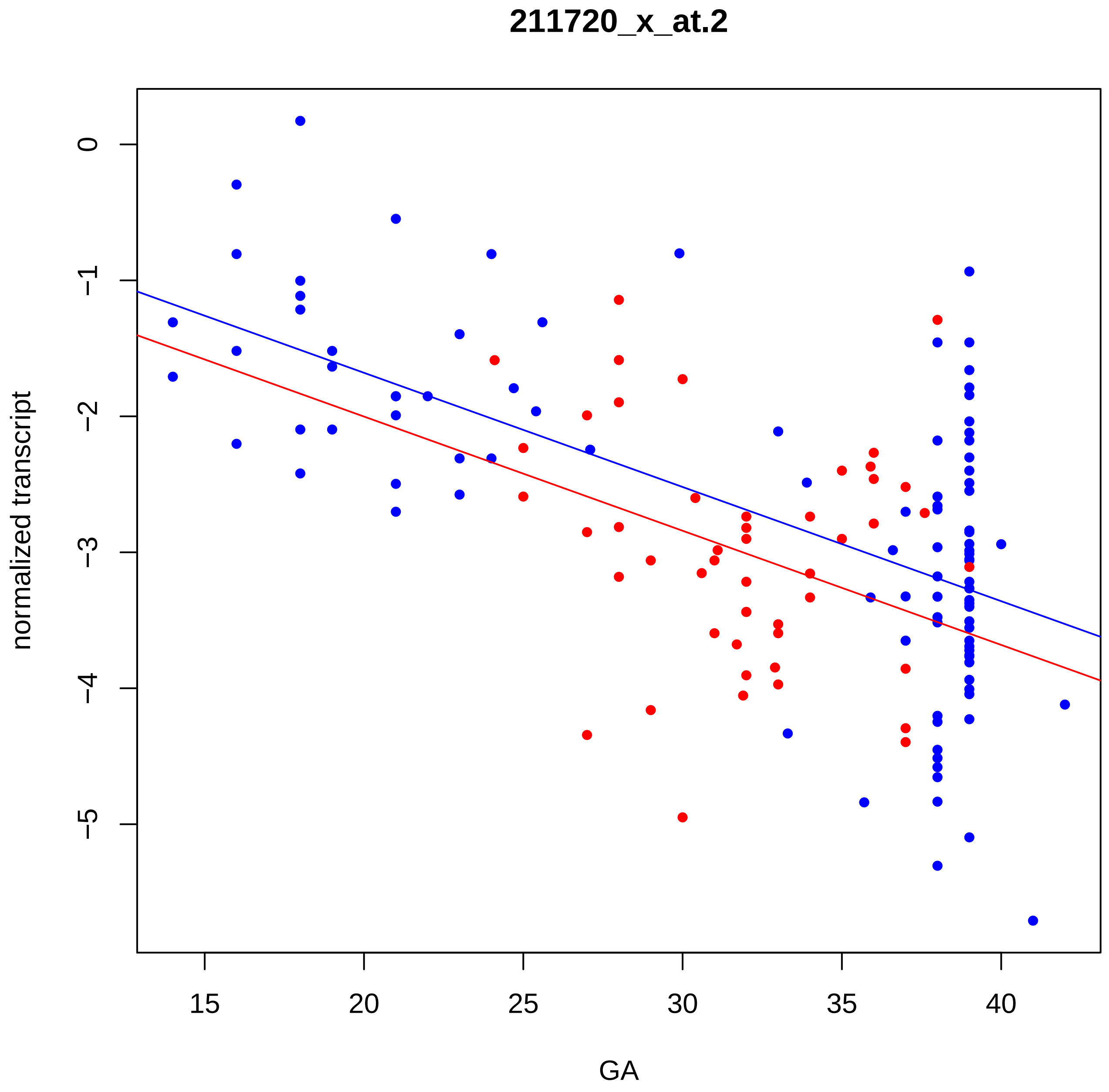

211720_x_at.2
normalized transcript
GA
0
−1
−2
−3
−4
−5
15
20
25
30
35
40

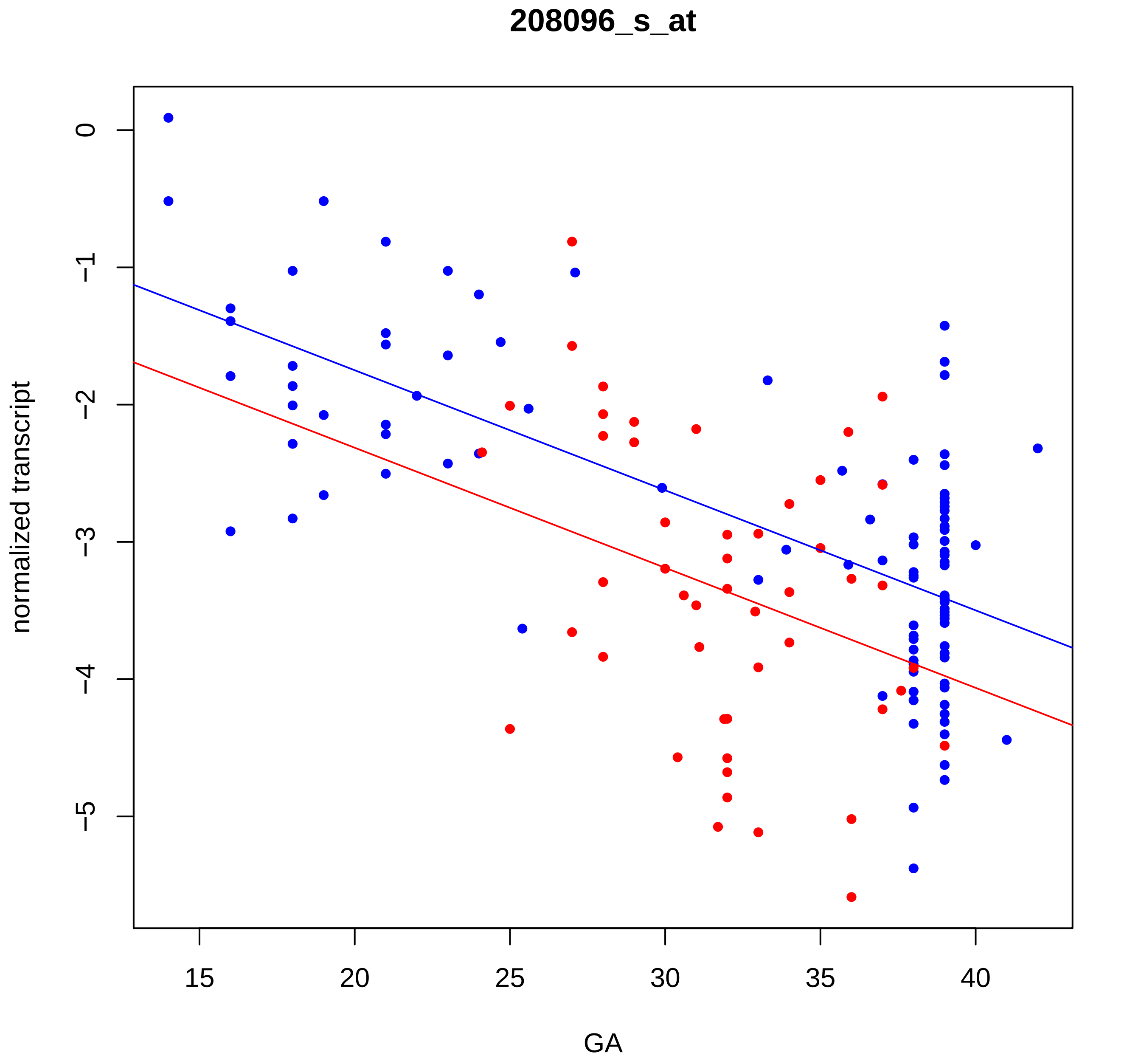
208096_s_at
normalized transcript
0
−1
−2
−3
−4
−5
15
20
25
30
35
40
GA

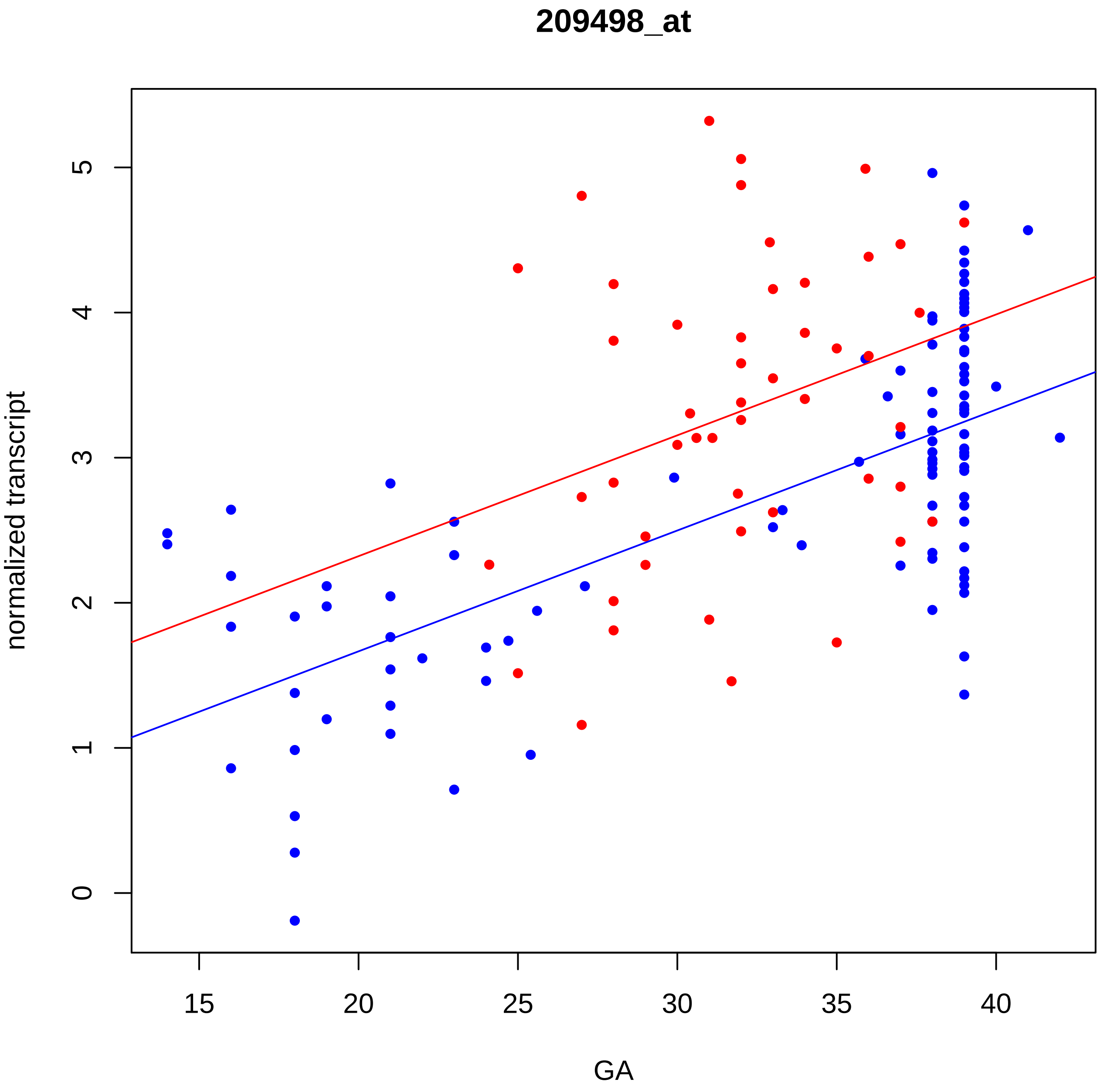

209498_at
normalized transcript
GA
0
1
2
3
4
5
15
20
25
30
35
40

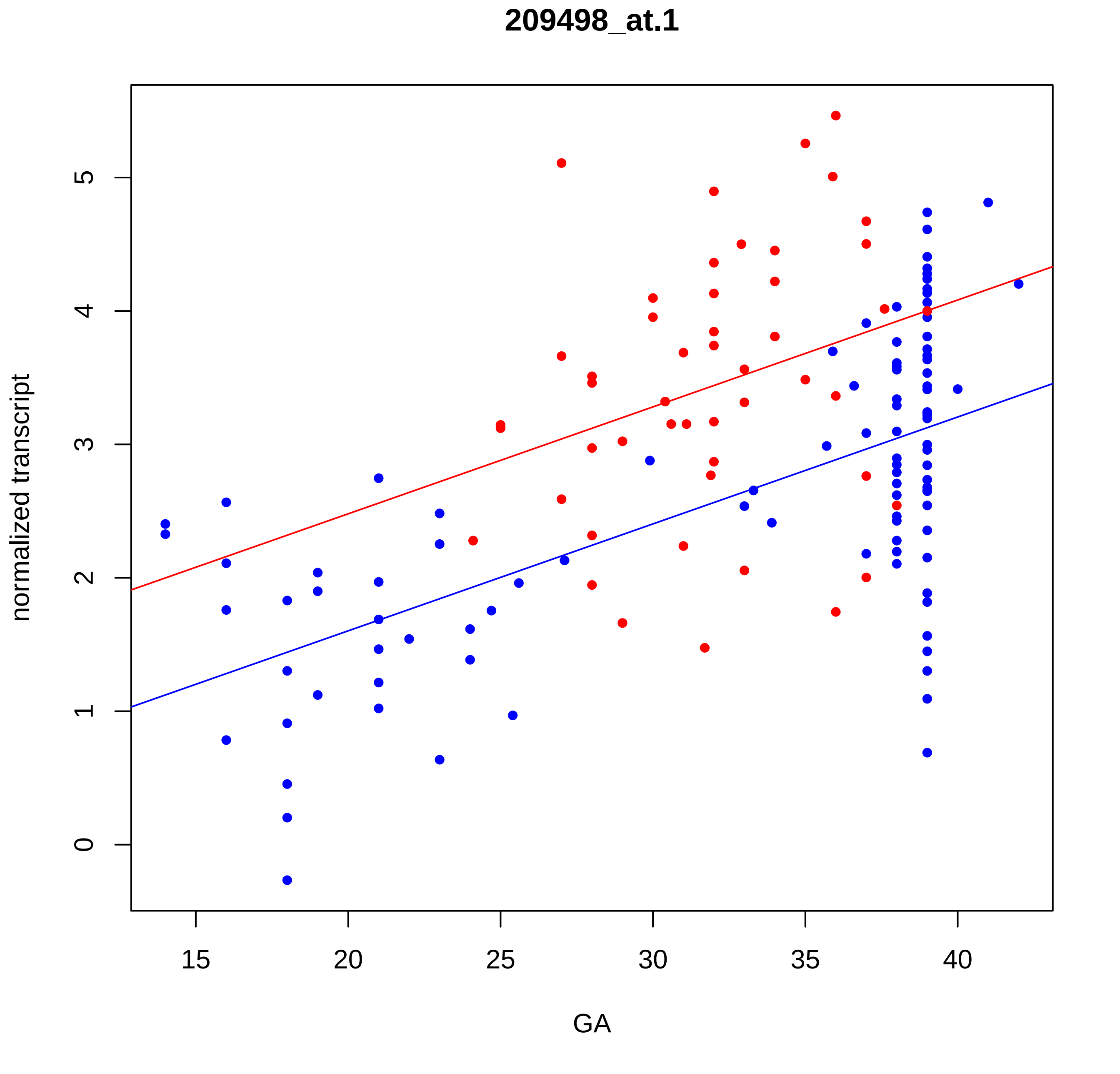

209498_at.1
normalized transcript
GA
0
1
2
3
4
5
15
20
25
30
35
40

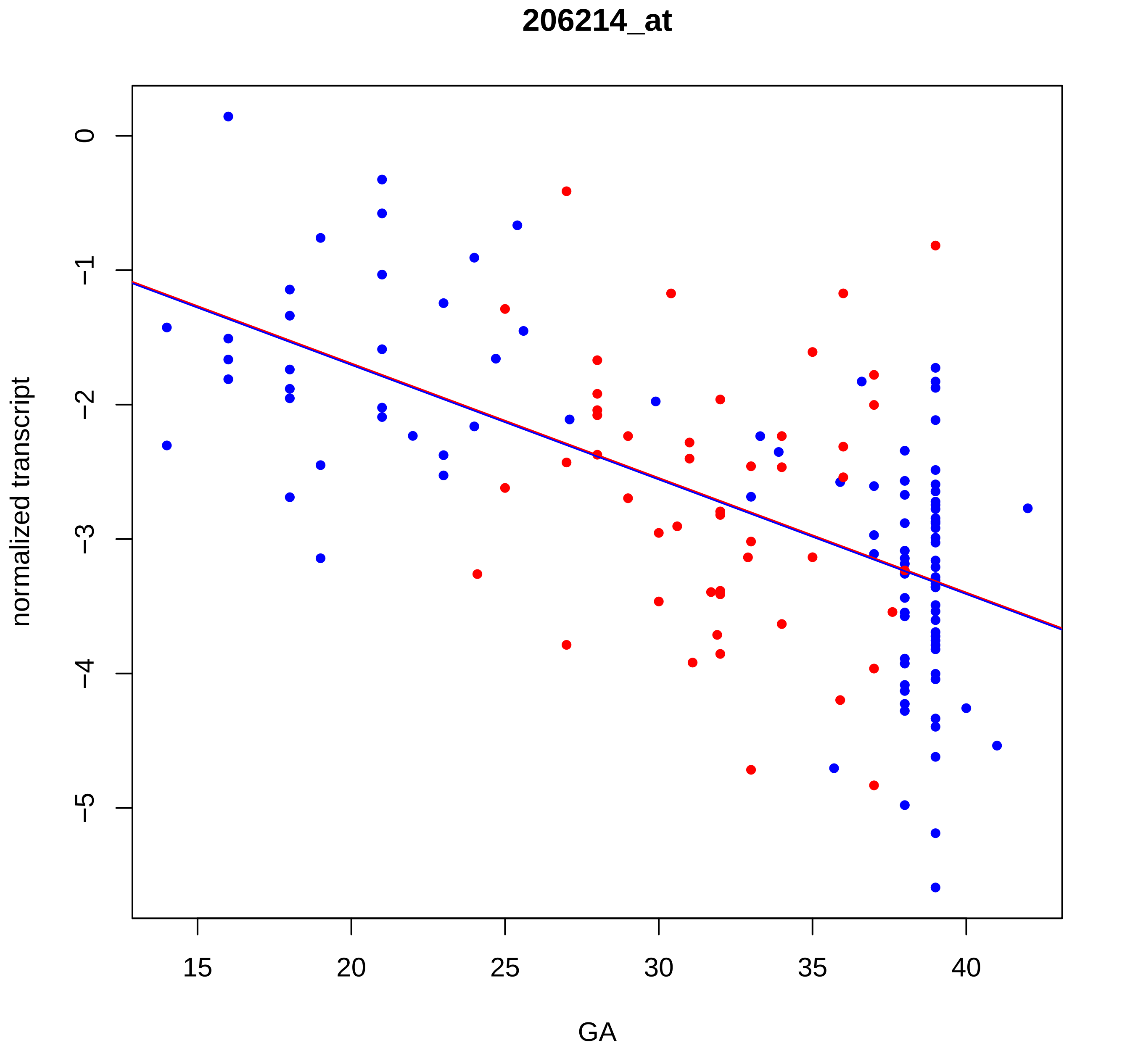
206214_at
normalized transcript
0
−1
−2
−3
−4
−5
15
20
25
30
35
40
GA

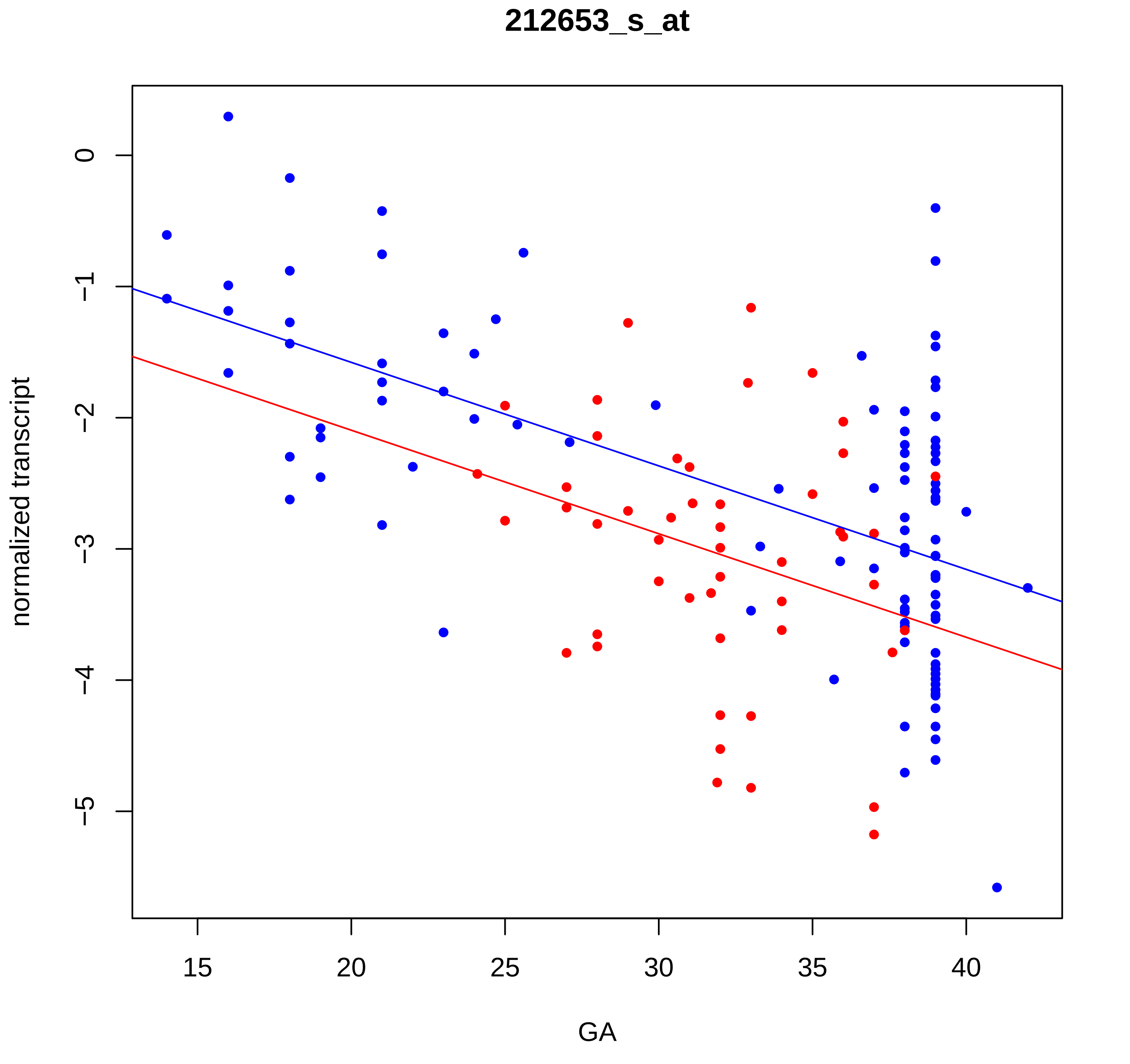

212653_s_at
normalized transcript
GA
0
−1
−2
−3
−4
−5
15
20
25
30
35
40

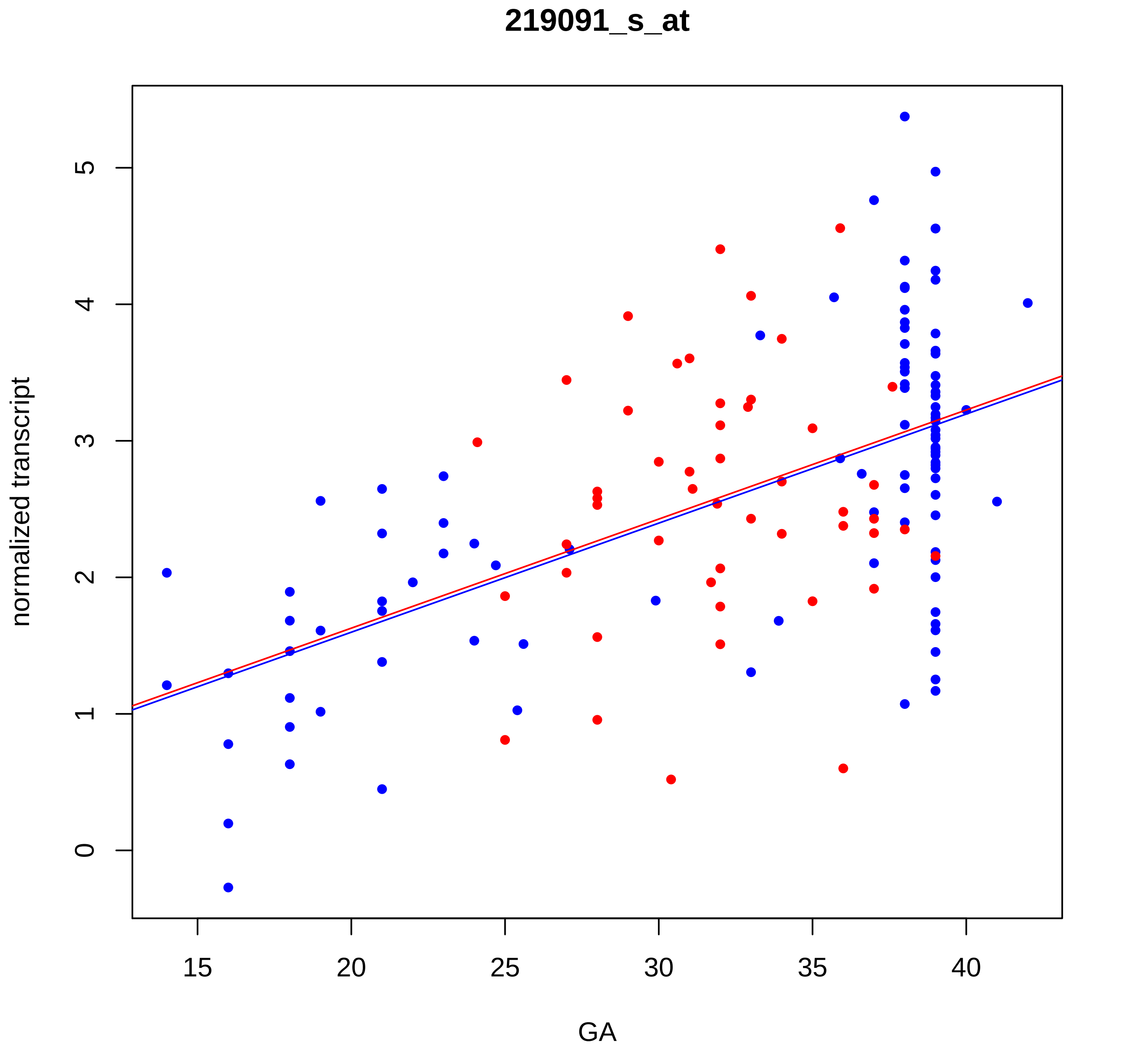

219091_s_at
normalized transcript
GA
0
1
2
3
4
5
15
20
25
30
35
40

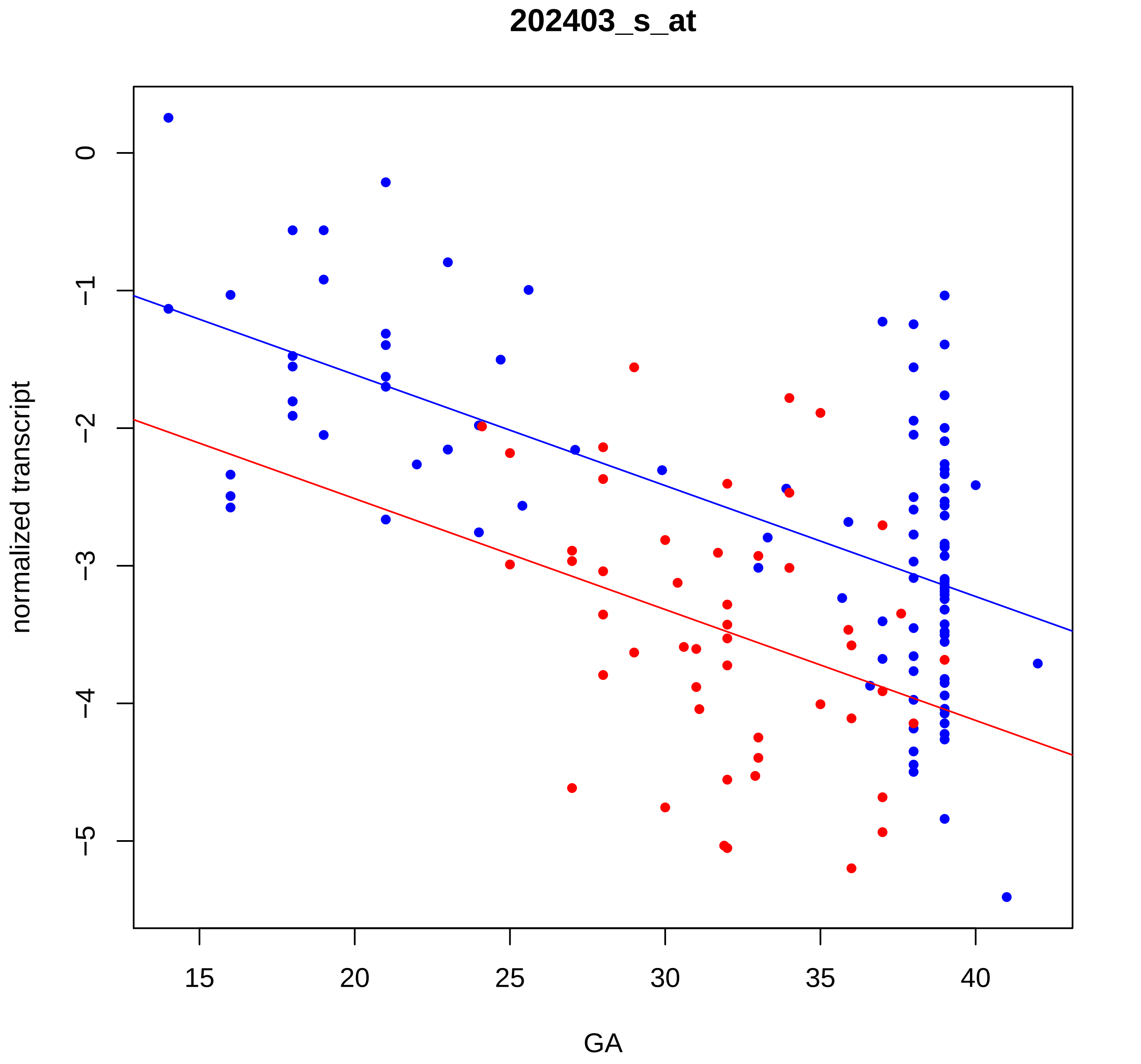

202403_s_at
0
−1
−2
−3
−4
−5
normalized transcript
15
20
25
30
35
40
GA

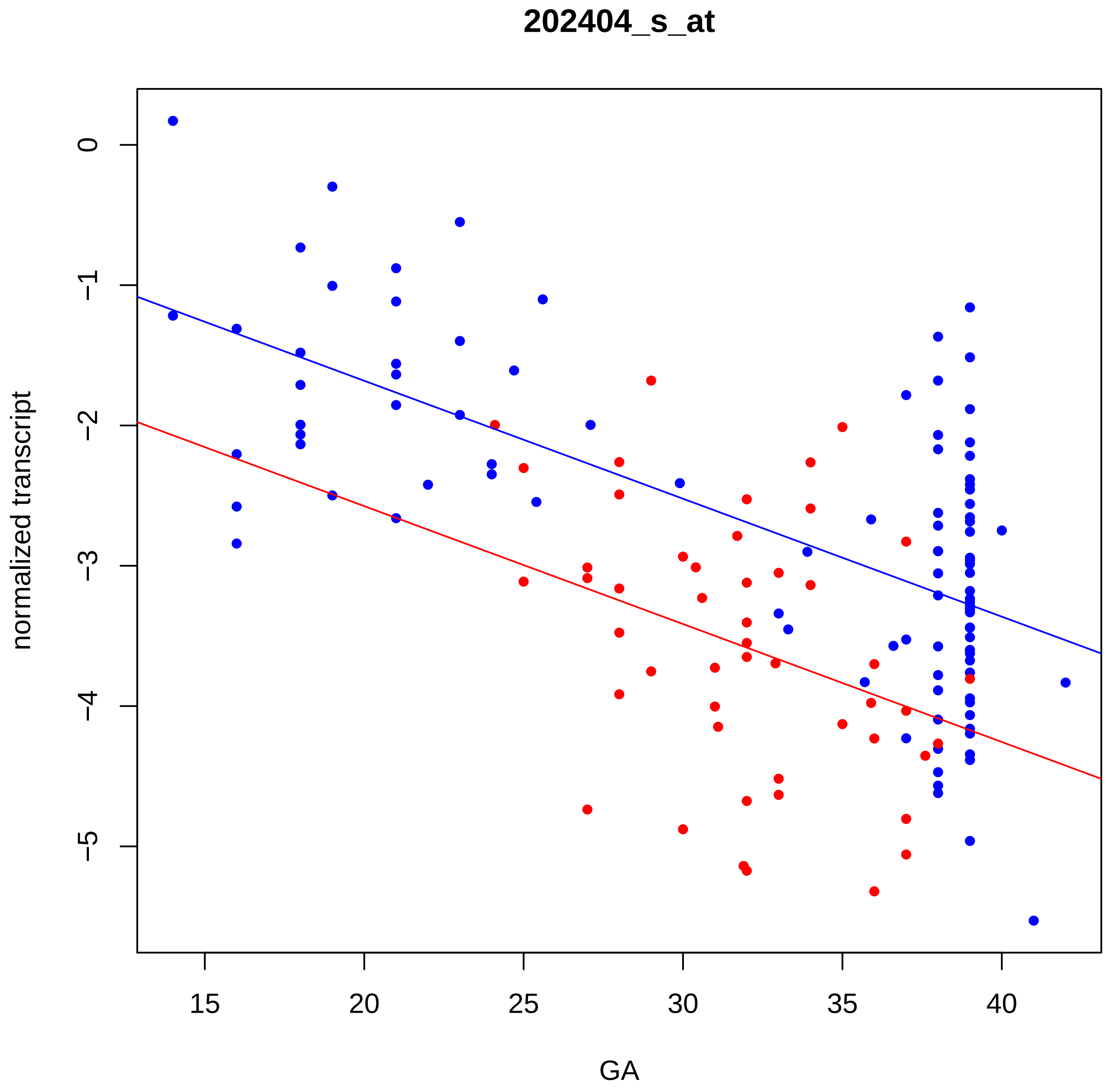

202404_s_at
normalized transcript
GA
0
−1
−2
−3
−4
−5
15
20
25
30
35
40

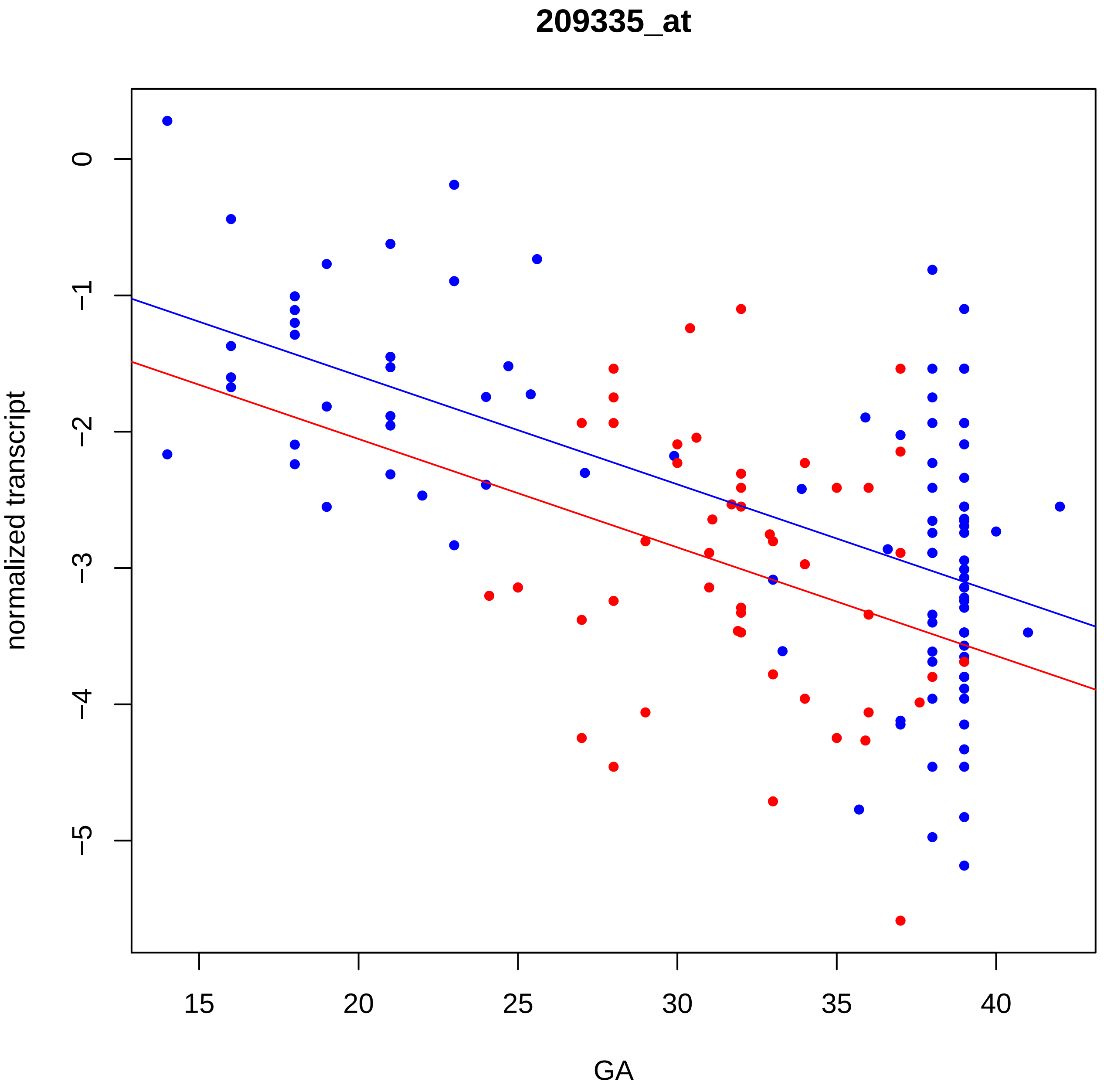

209335_at
normalized transcript
GA
0
−1
−2
−3
−4
−5
15
20
25
30
35
40

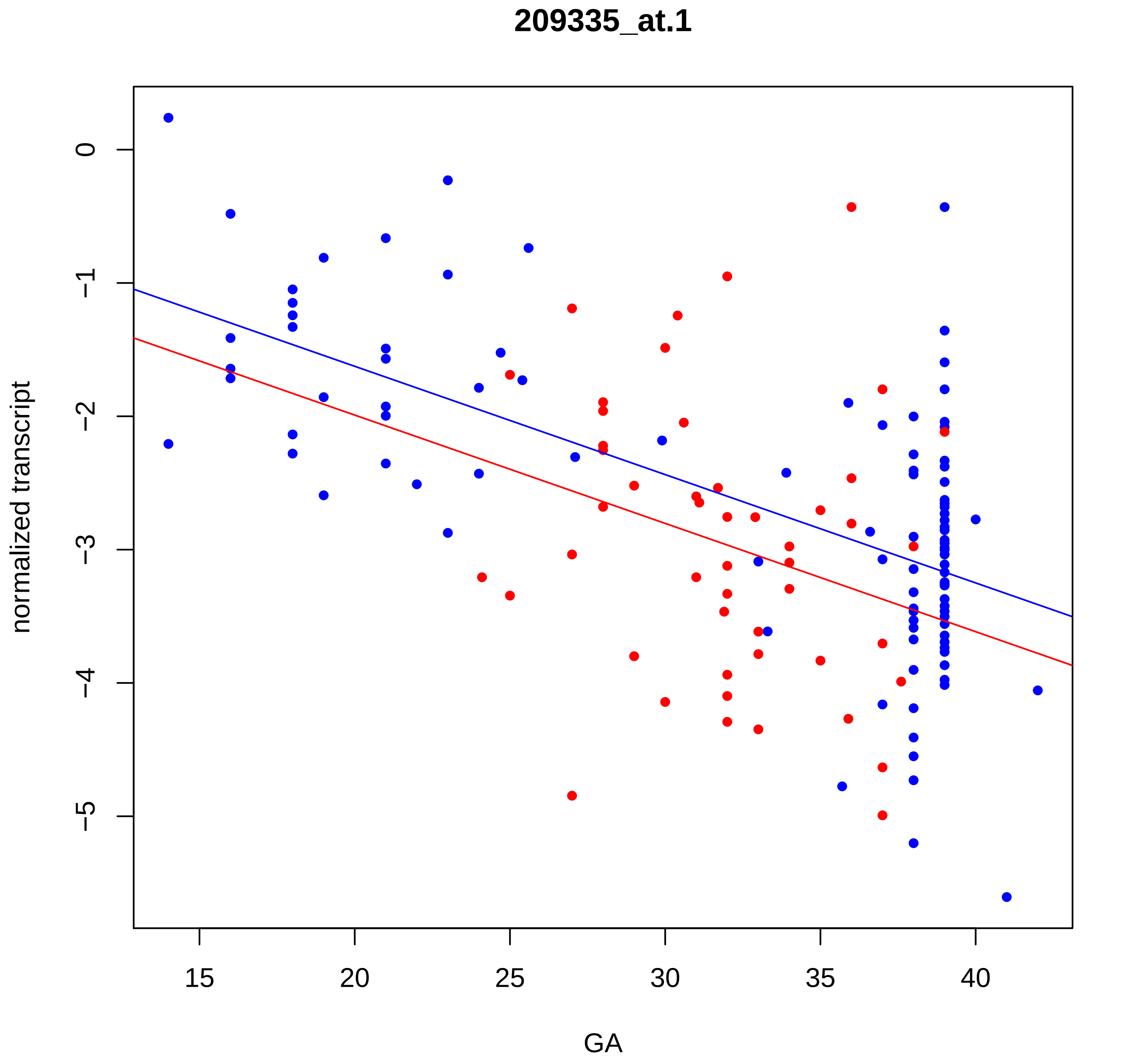
209335_at.1
normalized transcript
0
−1
−2
−3
−4
−5
15
20
25
30
35
40
GA

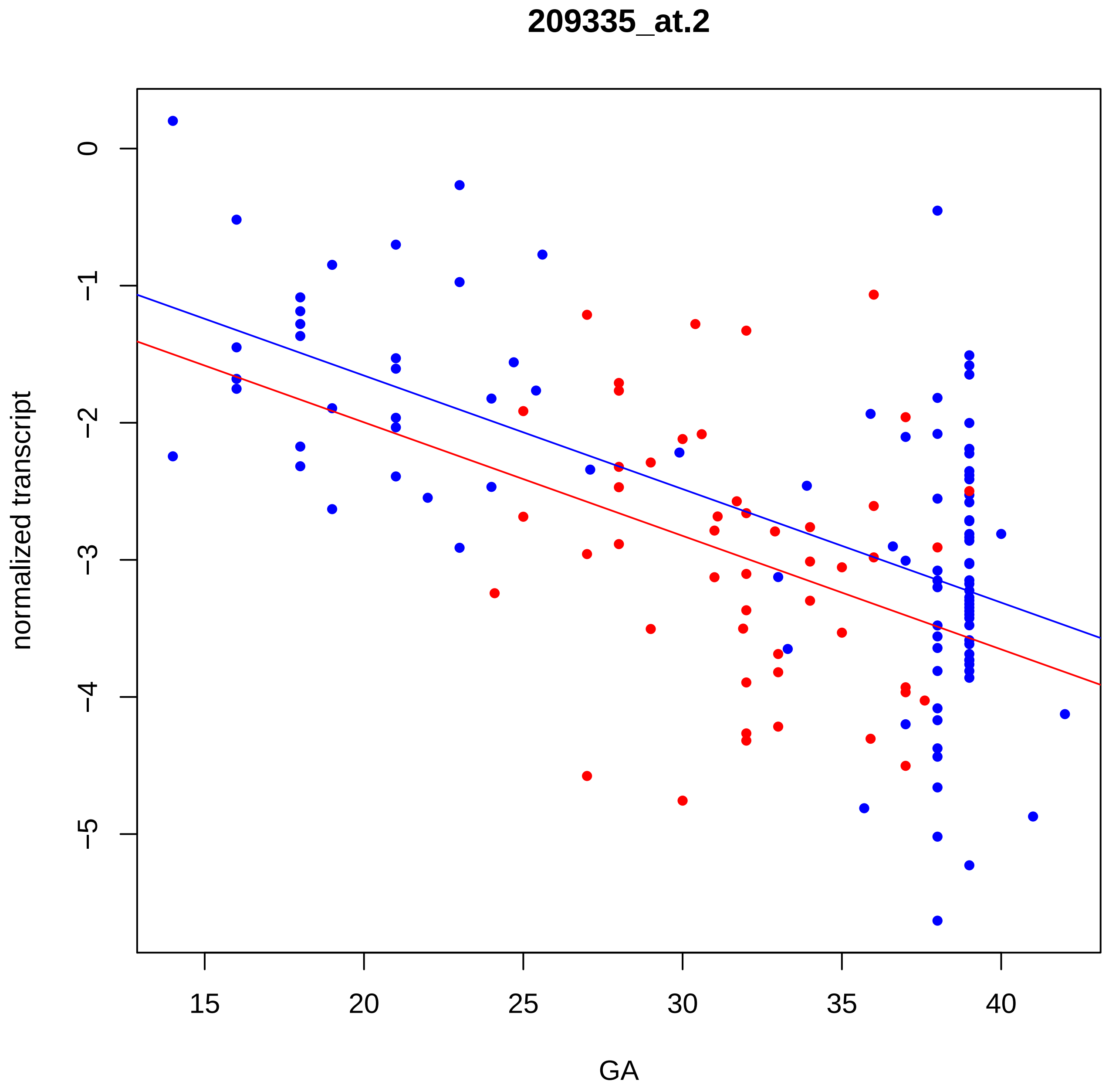

209335_at.2
normalized transcript
GA
0
−1
−2
−3
−4
−5
15
20
25
30
35
40

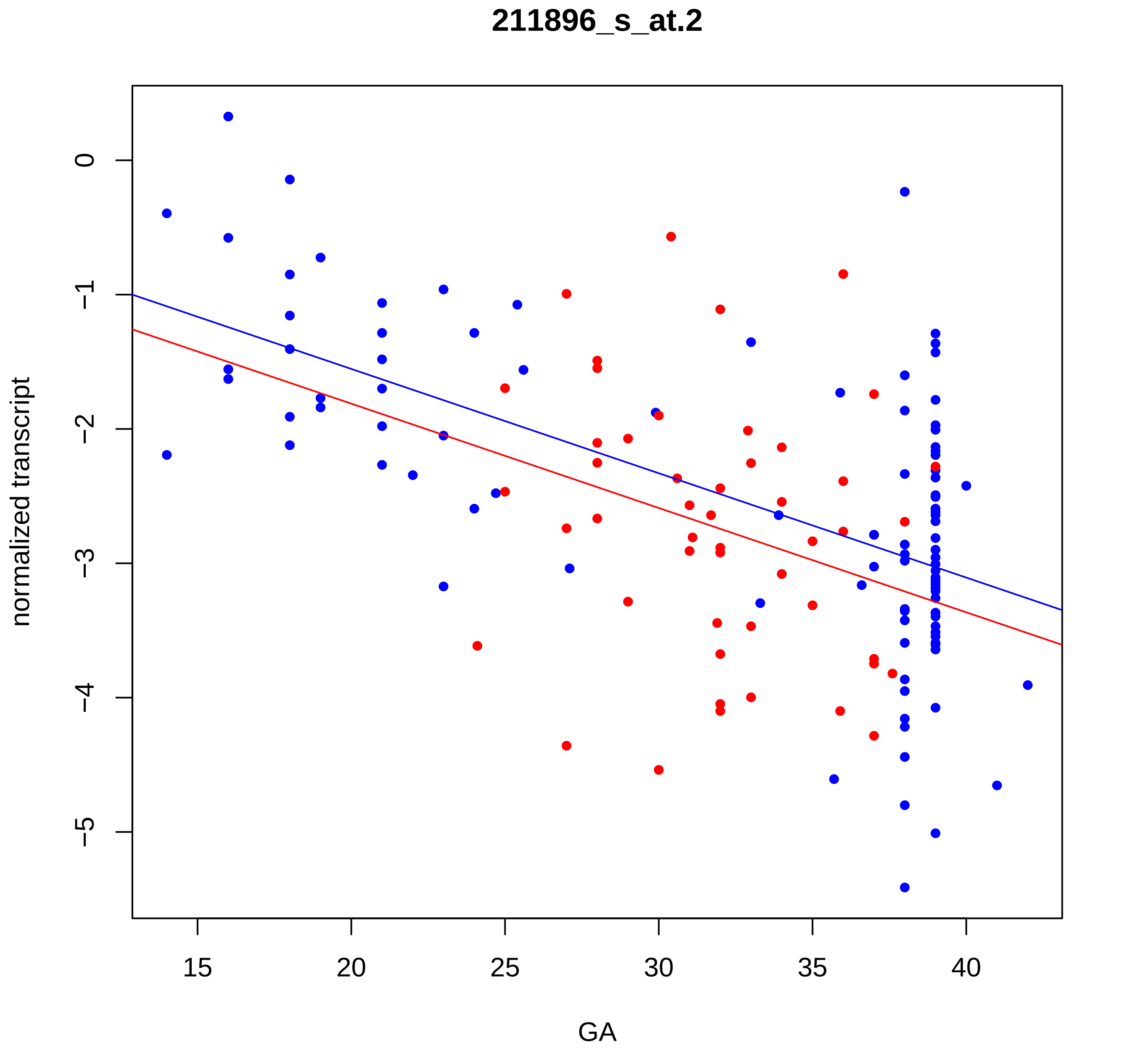

211896_s_at.2
normalized transcript
0
−1
−2
−3
−4
−5
15
20
25
30
35
40
GA

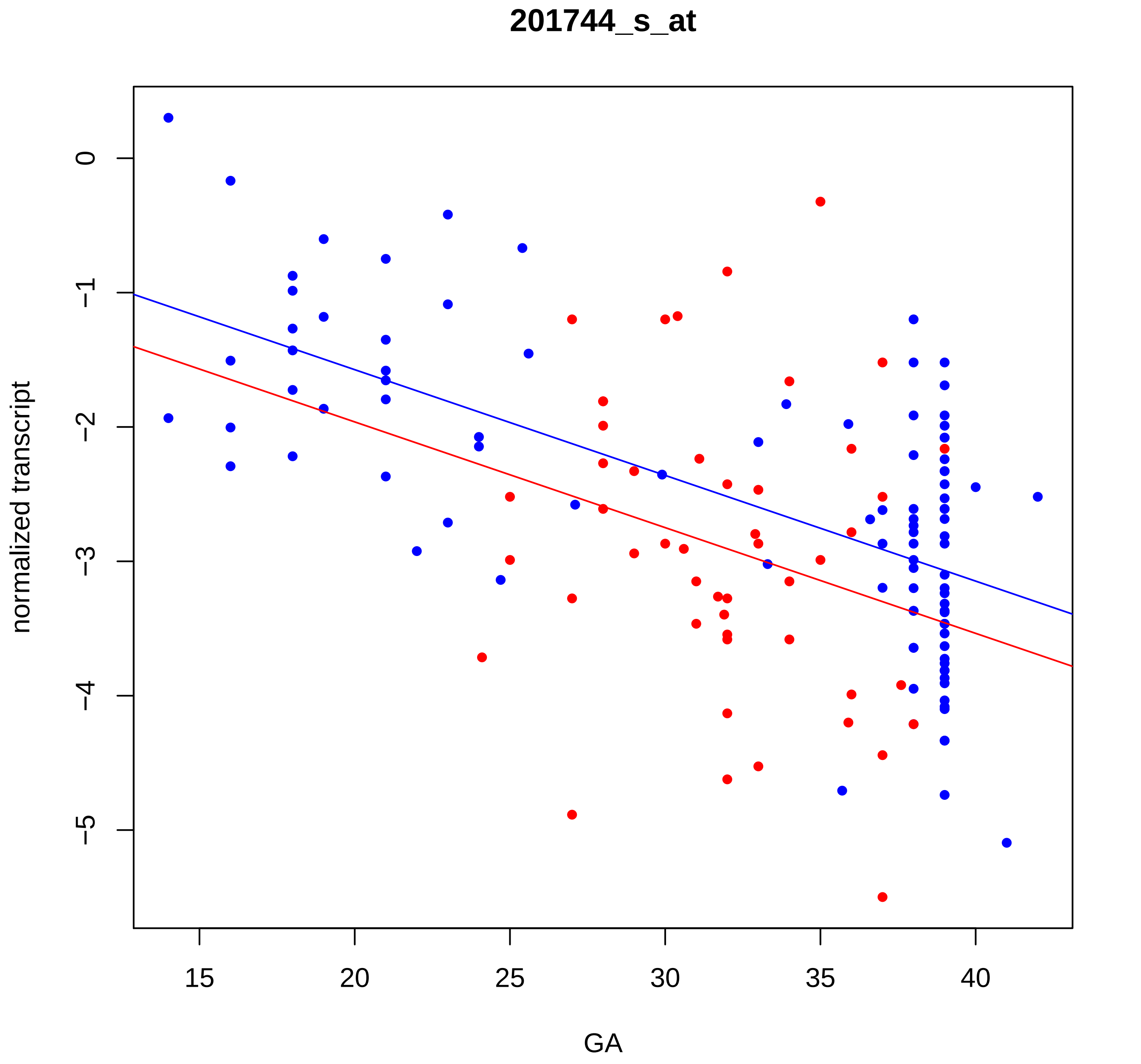

201744_s_at
normalized transcript
0
−1
−2
−3
−4
−5
15
20
25
30
35
40
GA

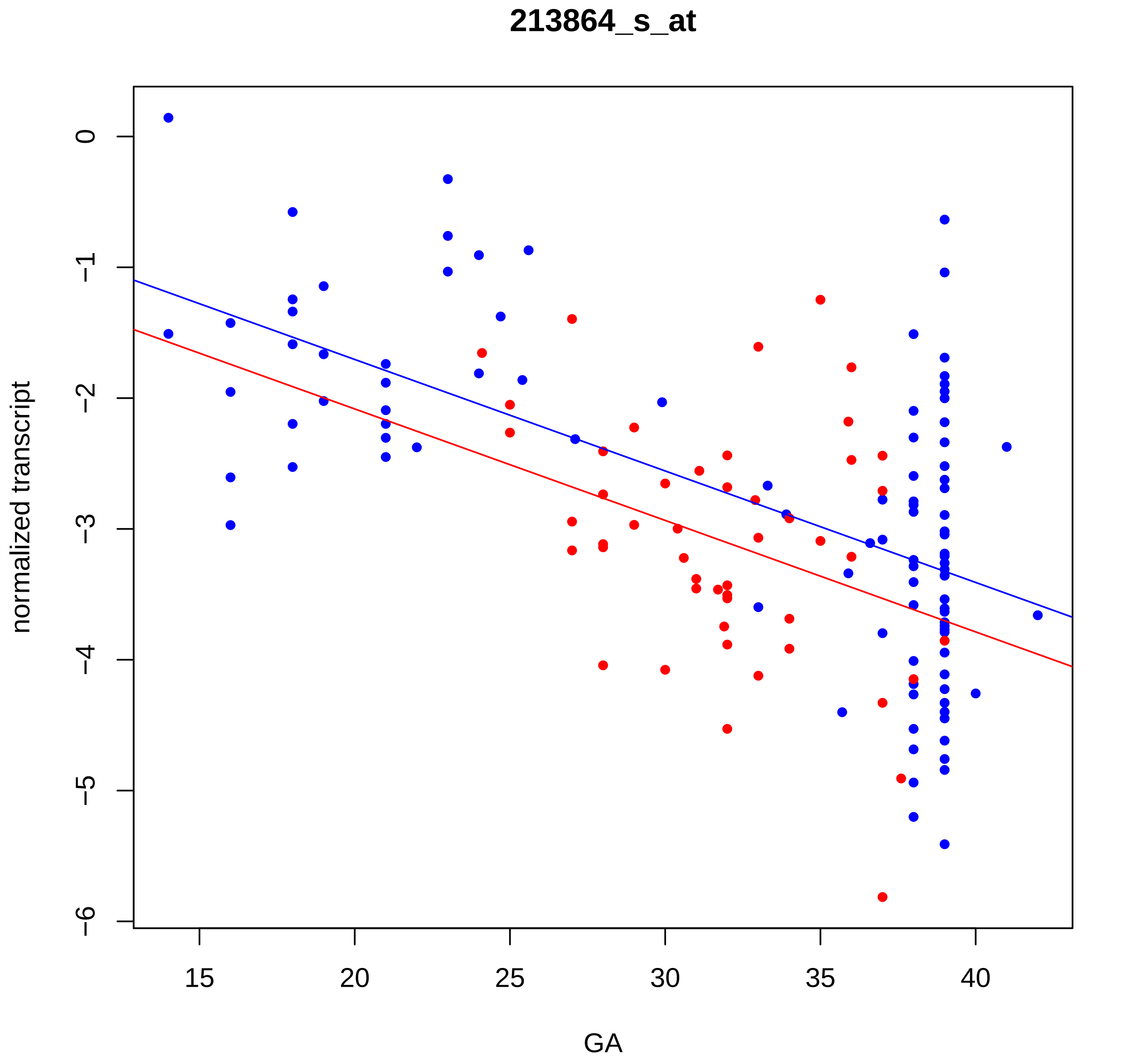

213864_s_at
normalized transcript
GA
0
−1
−2
−3
−4
−5
−6
15
20
25
30
35
40

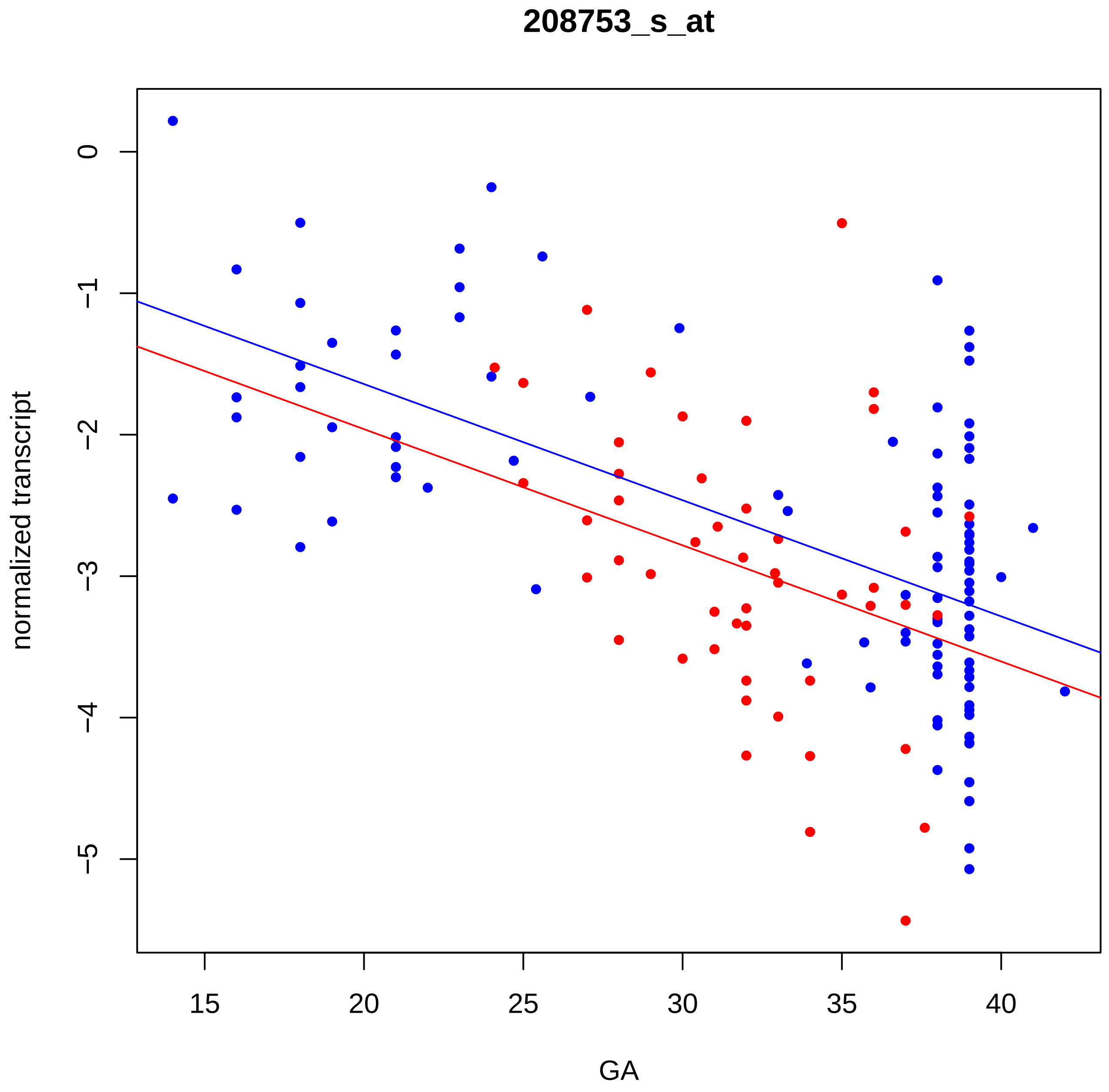

208753_s_at
normalized transcript
GA
0
−1
−2
−3
−4
−5
15
20
25
30
35
40

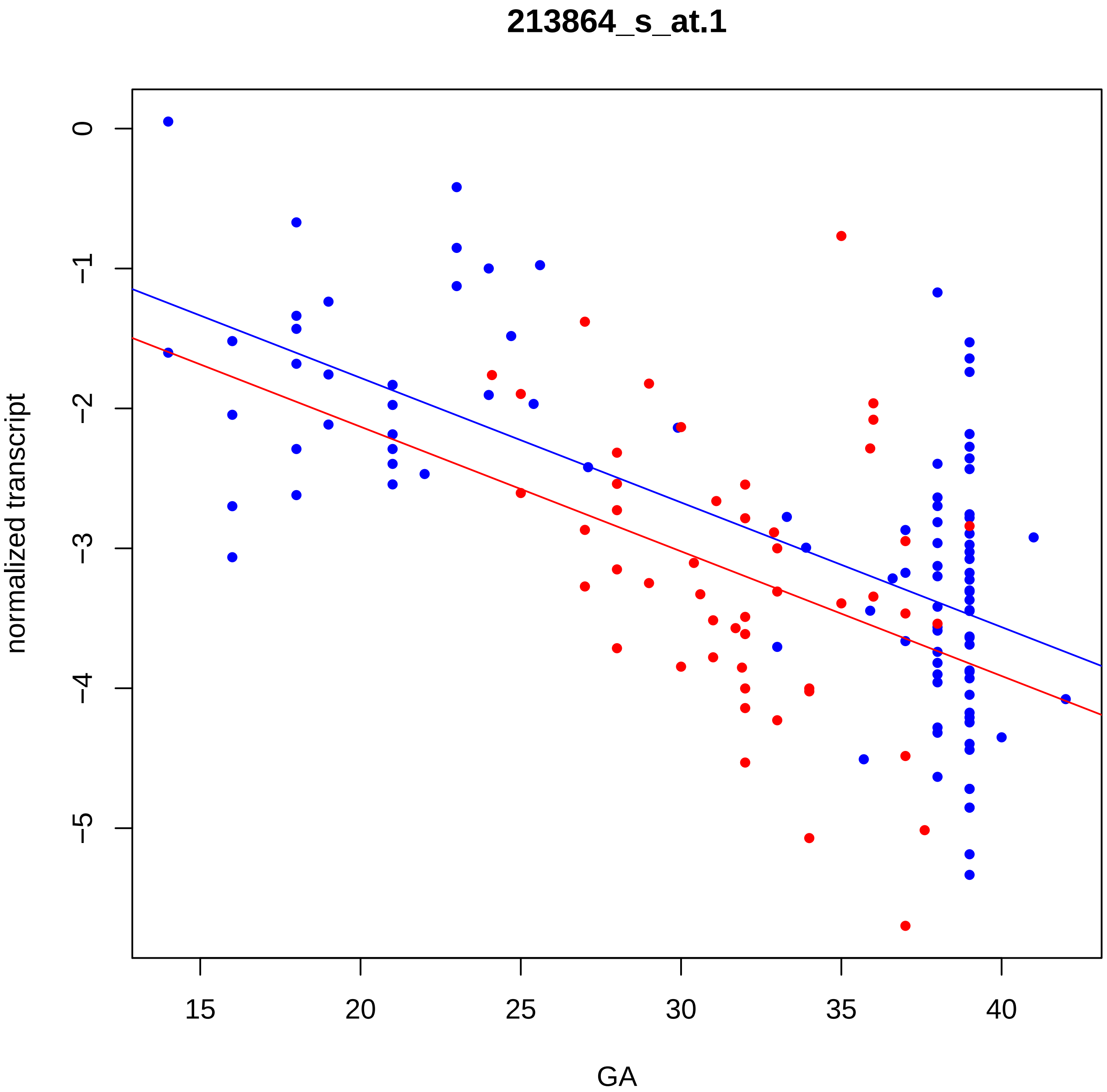

213864_s_at.1
normalized transcript
0
−1
−2
−3
−4
−5
15
20
25
30
35
40
GA

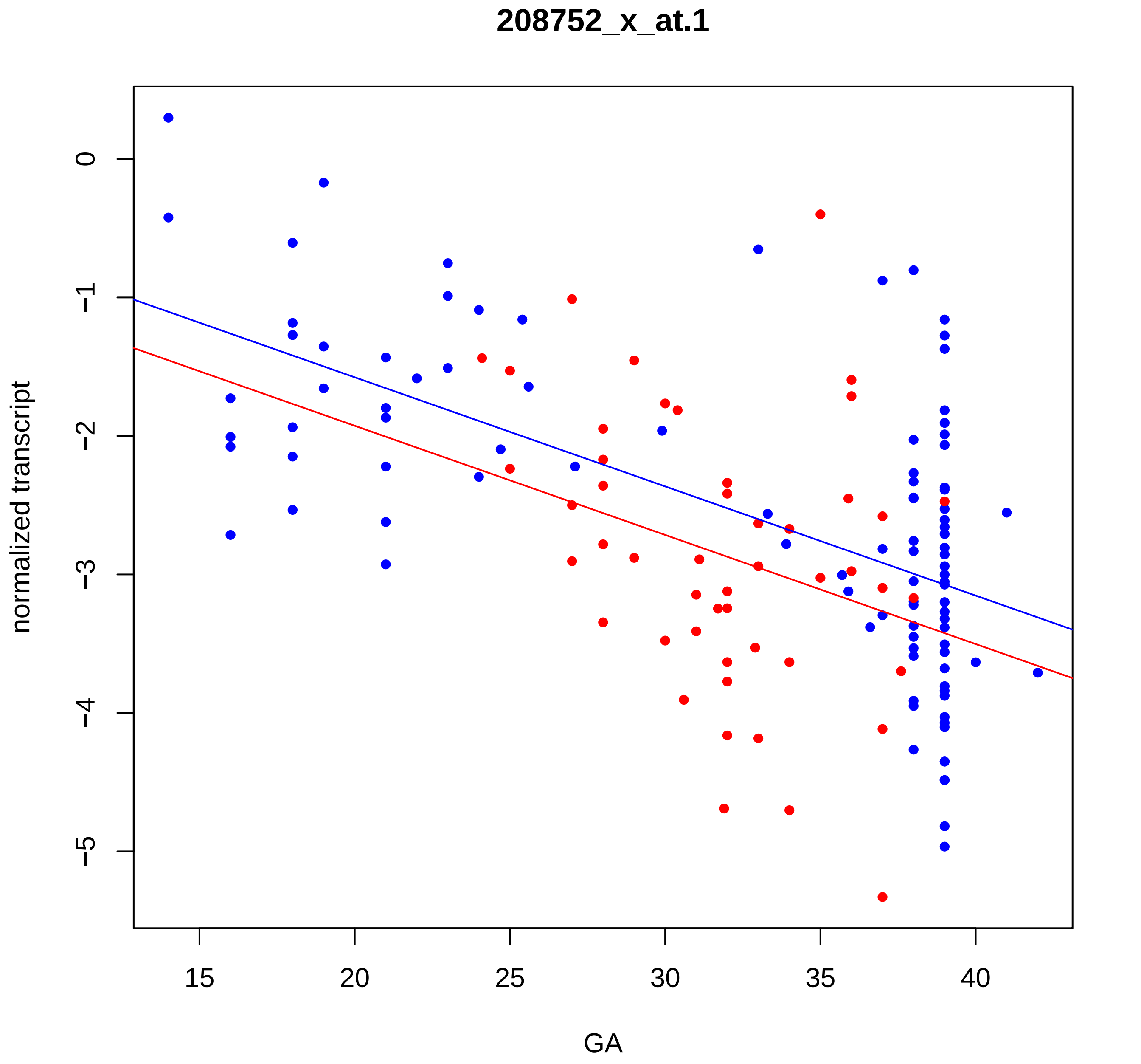

208752_x_at.1
normalized transcript
GA
0
−1
−2
−3
−4
−5
15
20
25
30
35
40

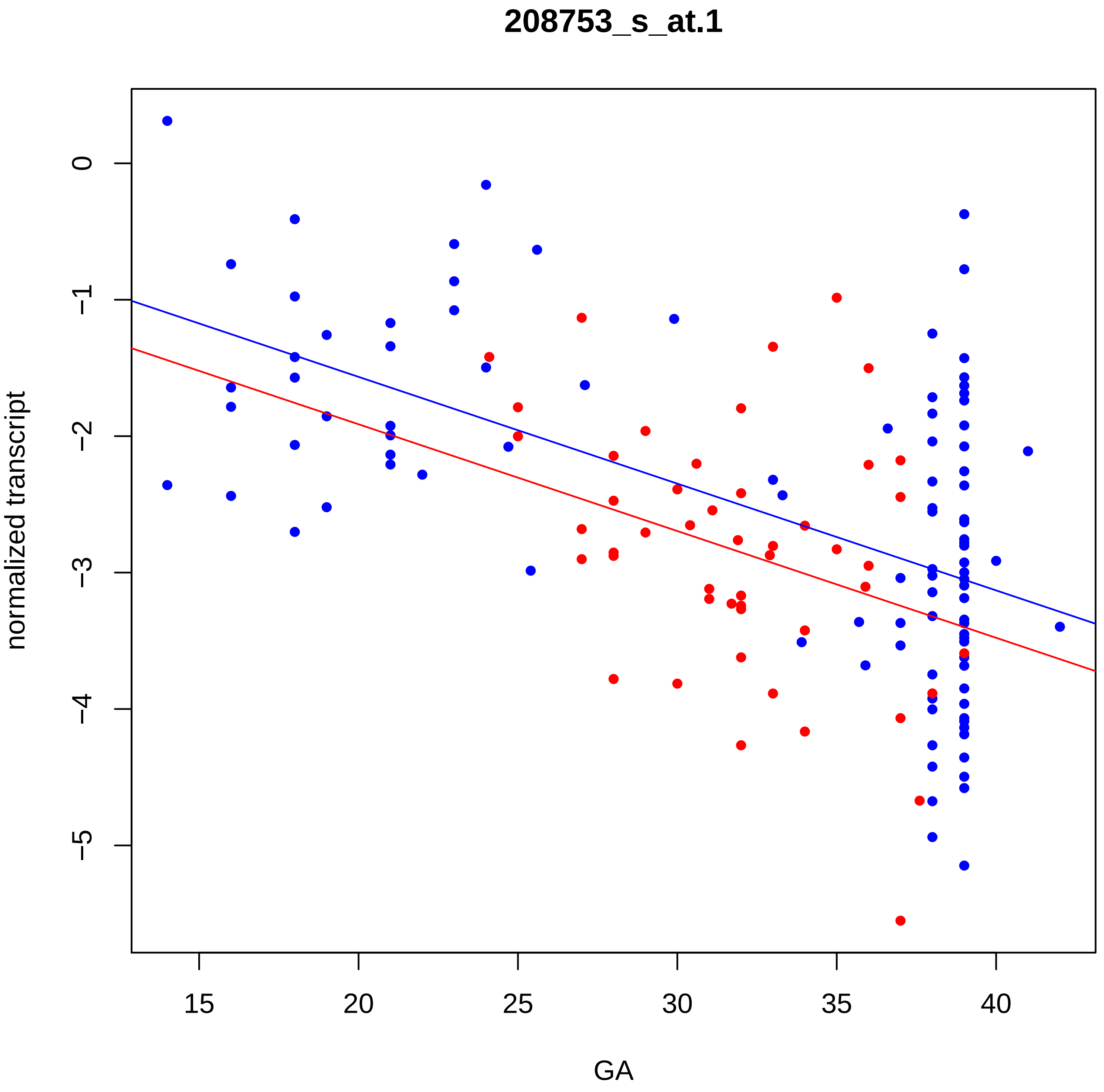
208753_s_at.1
normalized transcript
0
−1
−2
−3
−4
−5
15
20
25
30
35
40
GA

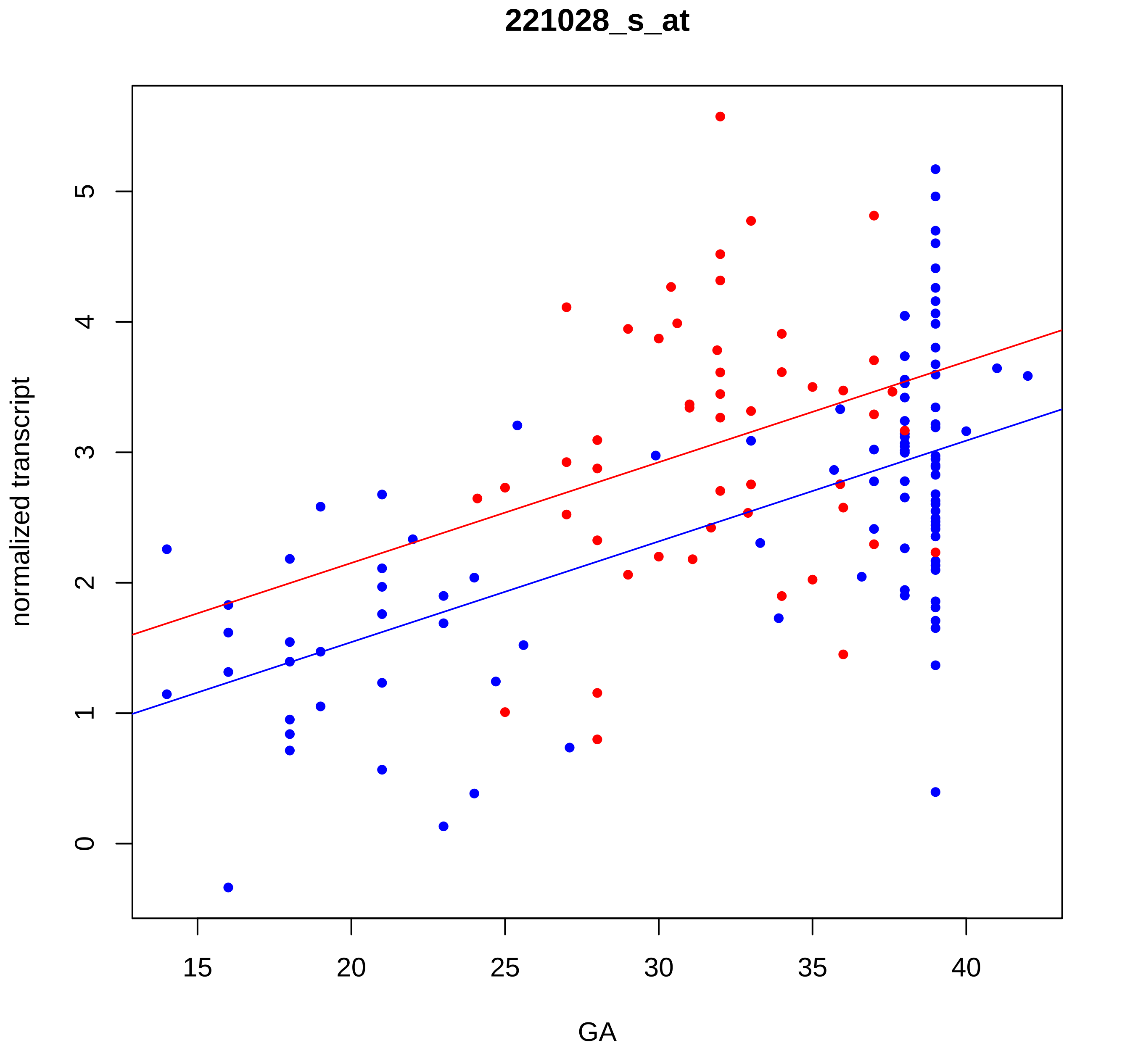

221028_s_at
normalized transcript
GA
0
1
2
3
4
5
15
20
25
30
35
40

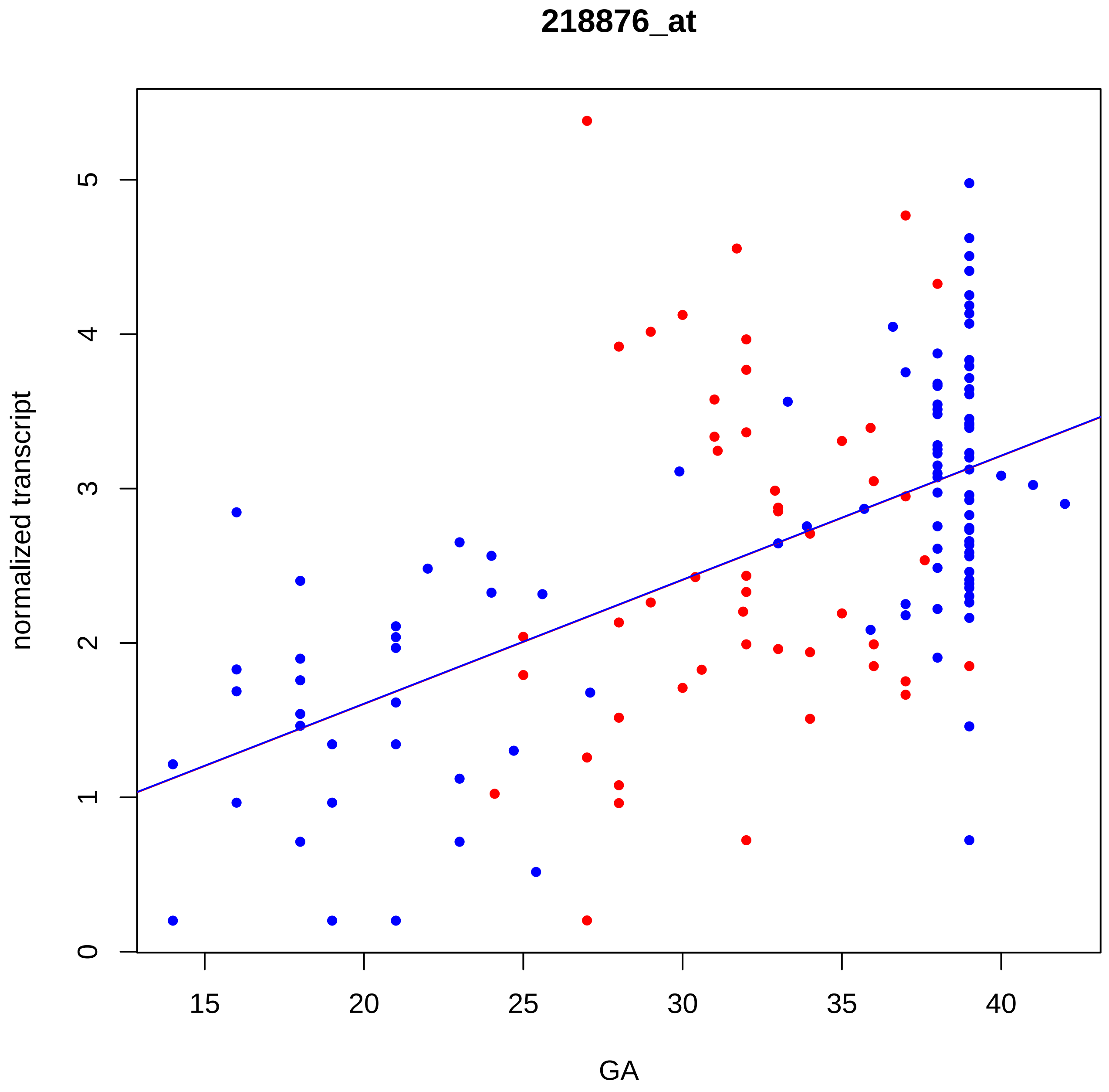
218876_at
normalized transcript
GA
0
1
2
3
4
5
15
20
25
30
35
40

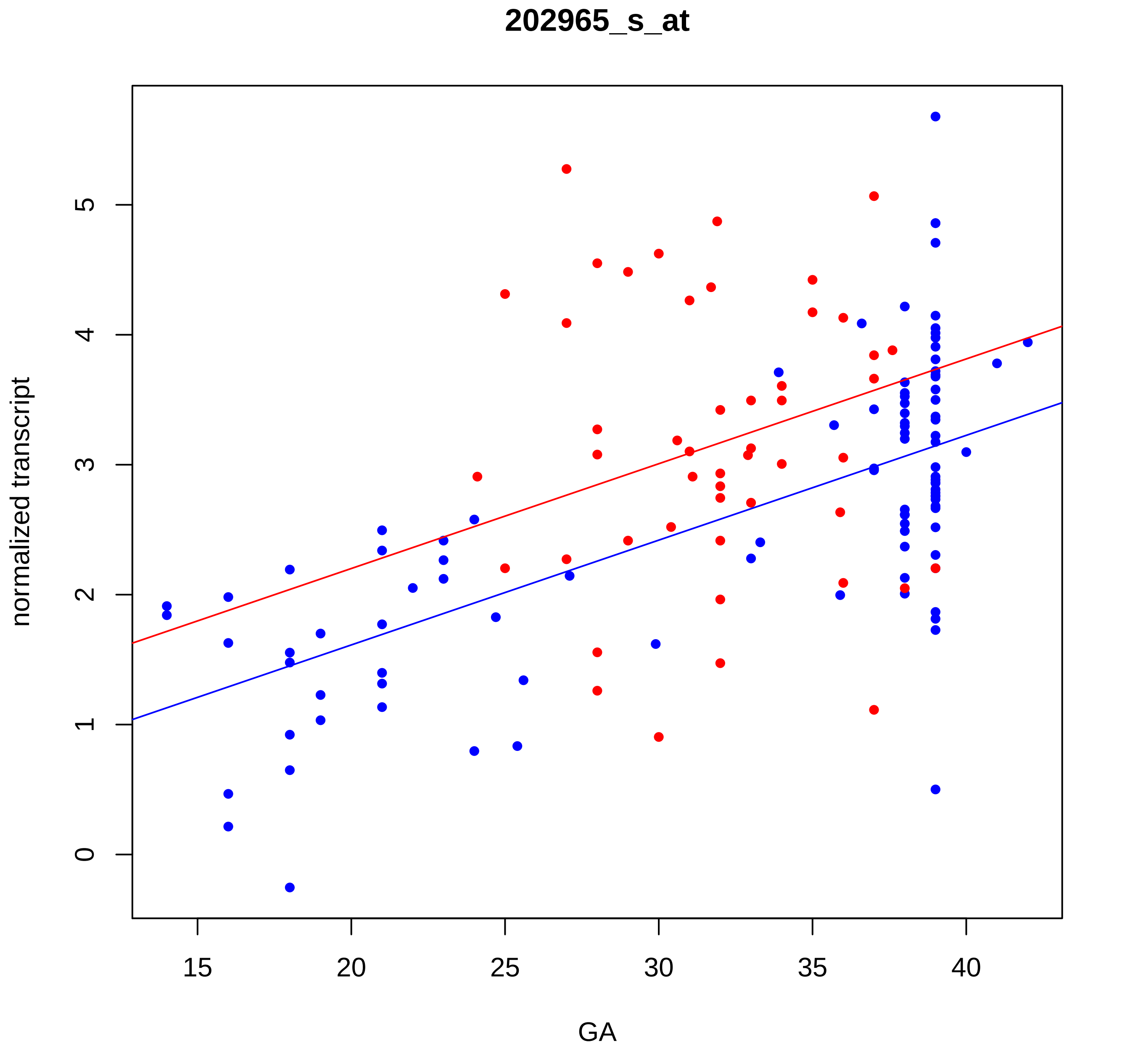
202965_s_at
normalized transcript
GA
0
1
2
3
4
5
15
20
25
30
35
40

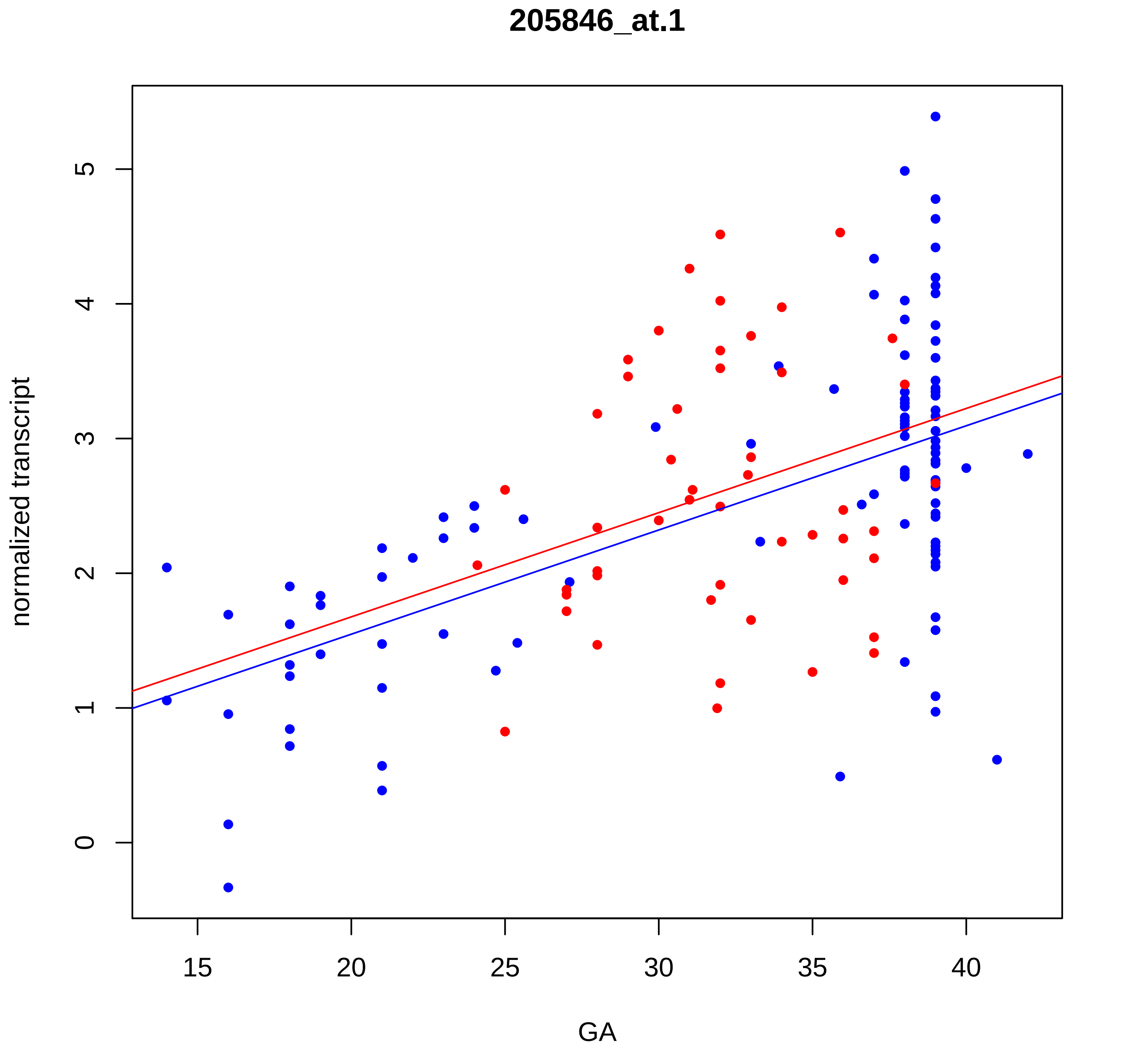
205846_at.1
normalized transcript
GA
0
1
2
3
4
5
15
20
25
30
35
40

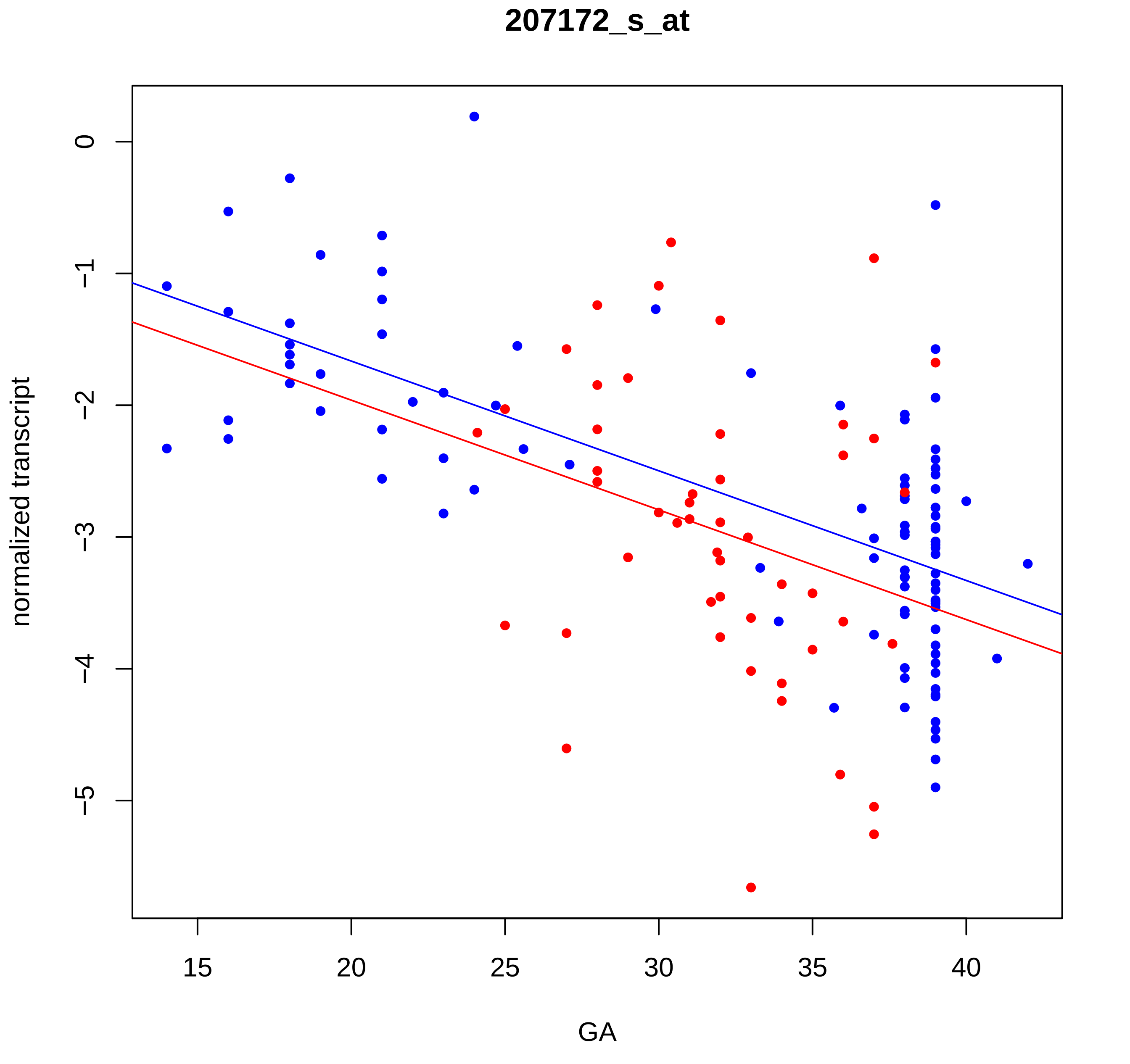

207172_s_at
normalized transcript
GA
0
−1
−2
−3
−4
−5
15
20
25
30
35
40

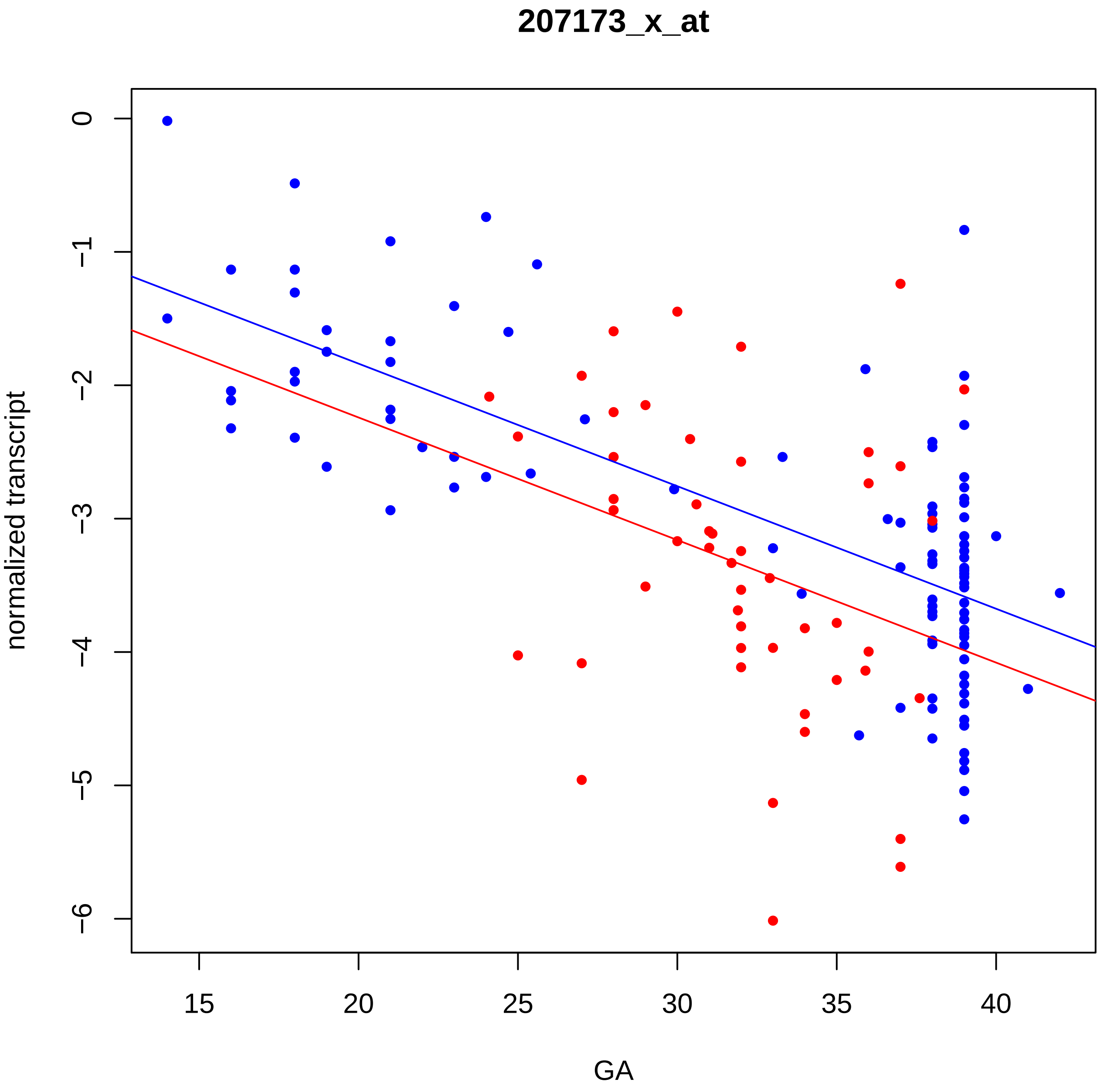
207173_x_at
normalized transcript
GA
0
−1
−2
−3
−4
−5
−6
15
20
25
30
35
40

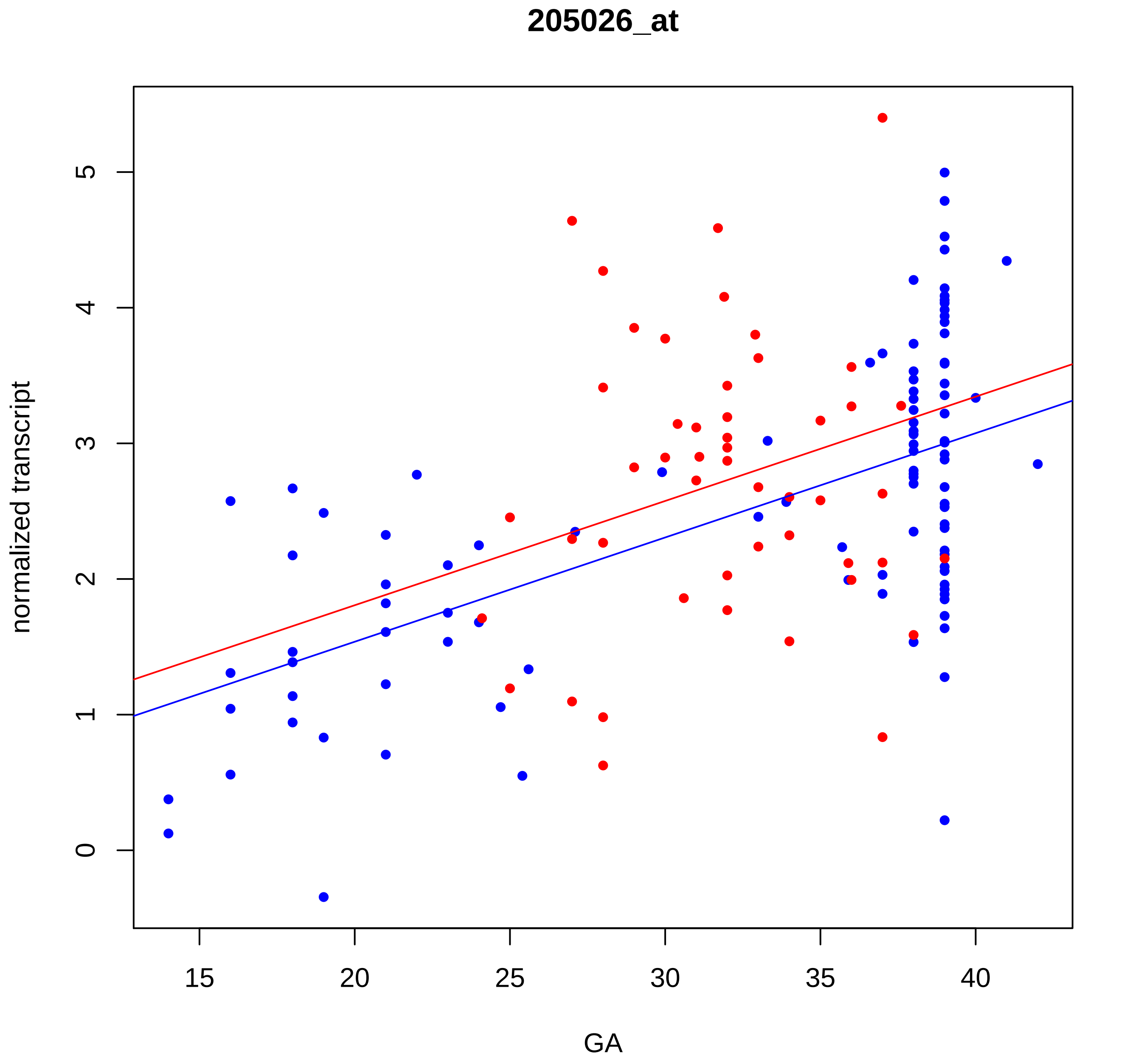
205026_at
normalized transcript
0
1
2
3
4
5
15
20
25
30
35
40
GA

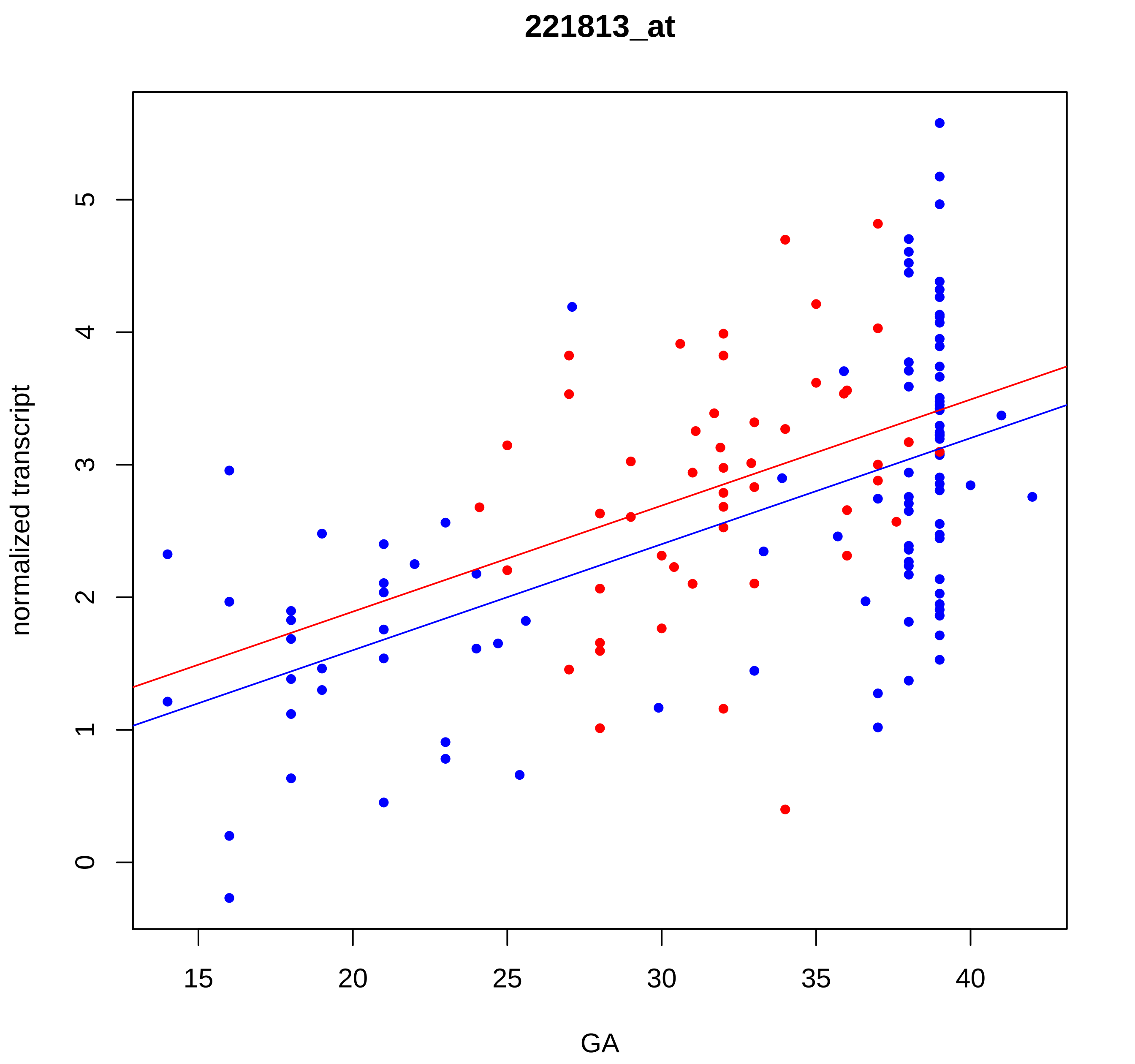

221813_at
normalized transcript
GA
0
1
2
3
4
5
15
20
25
30
35
40

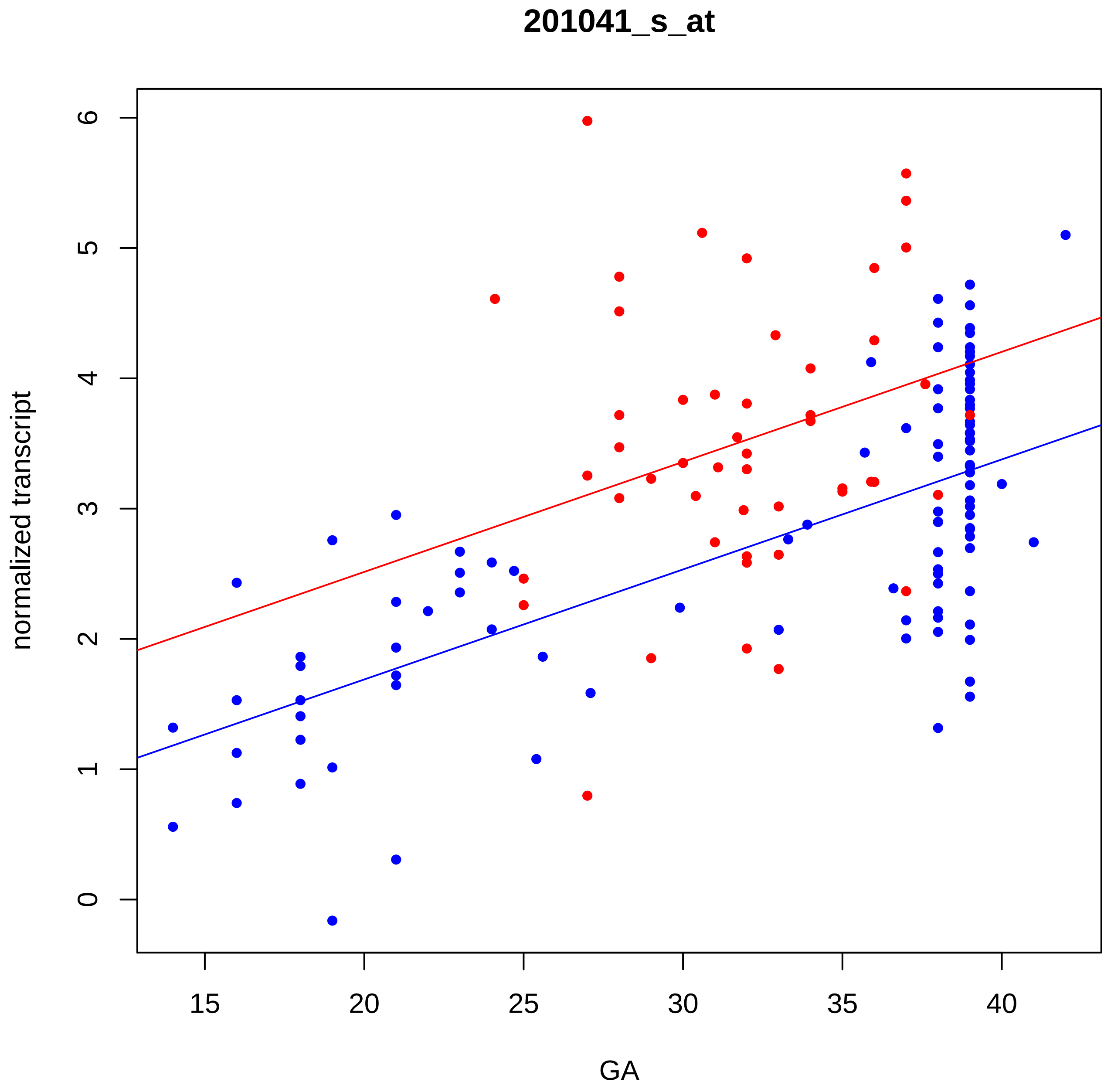
201041_s_at
normalized transcript
GA
0
1
2
3
4
5
6
15
20
25
30
35
40

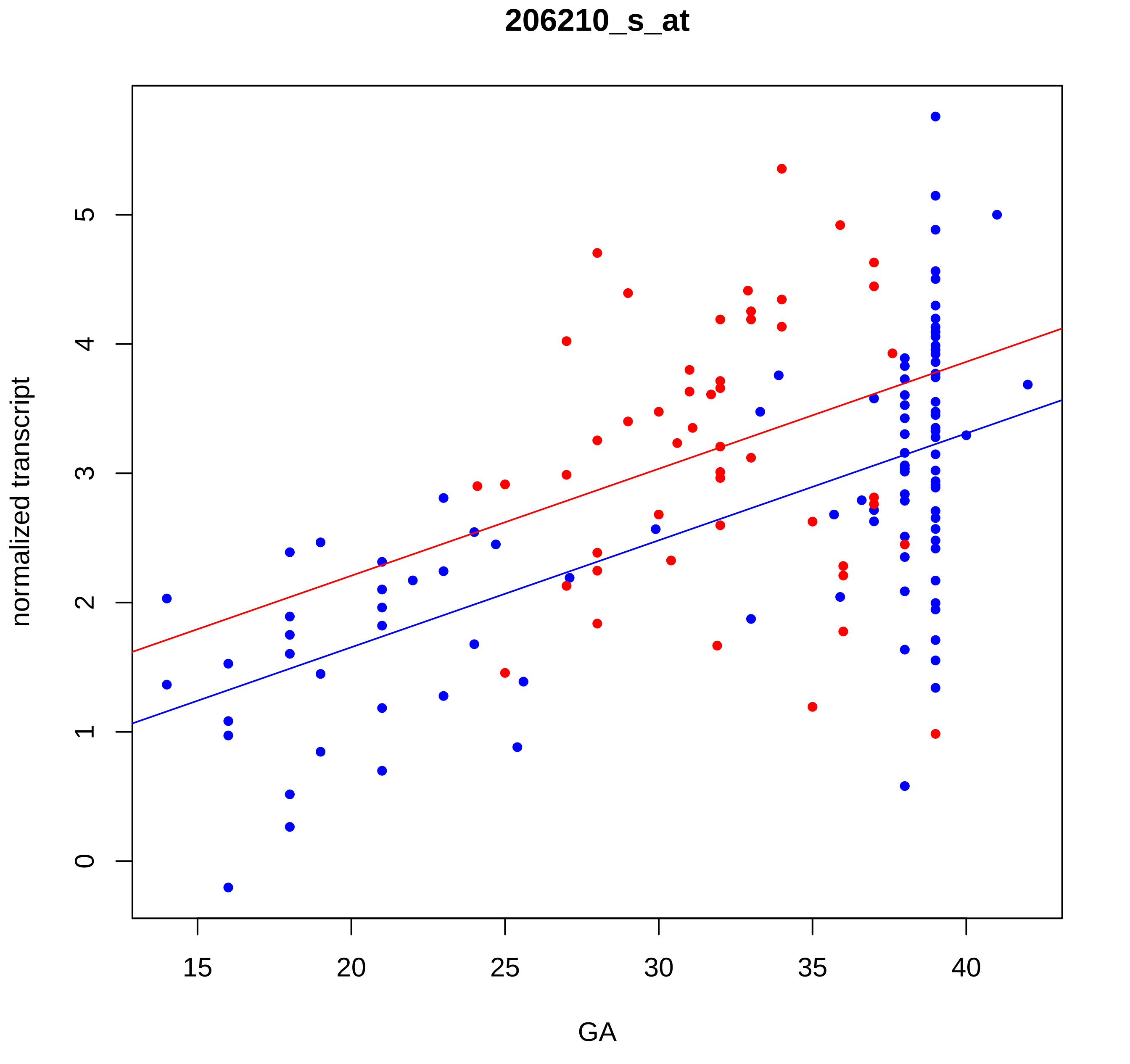
206210_s_at
normalized transcript
GA
0
1
2
3
4
5
15
20
25
30
35
40

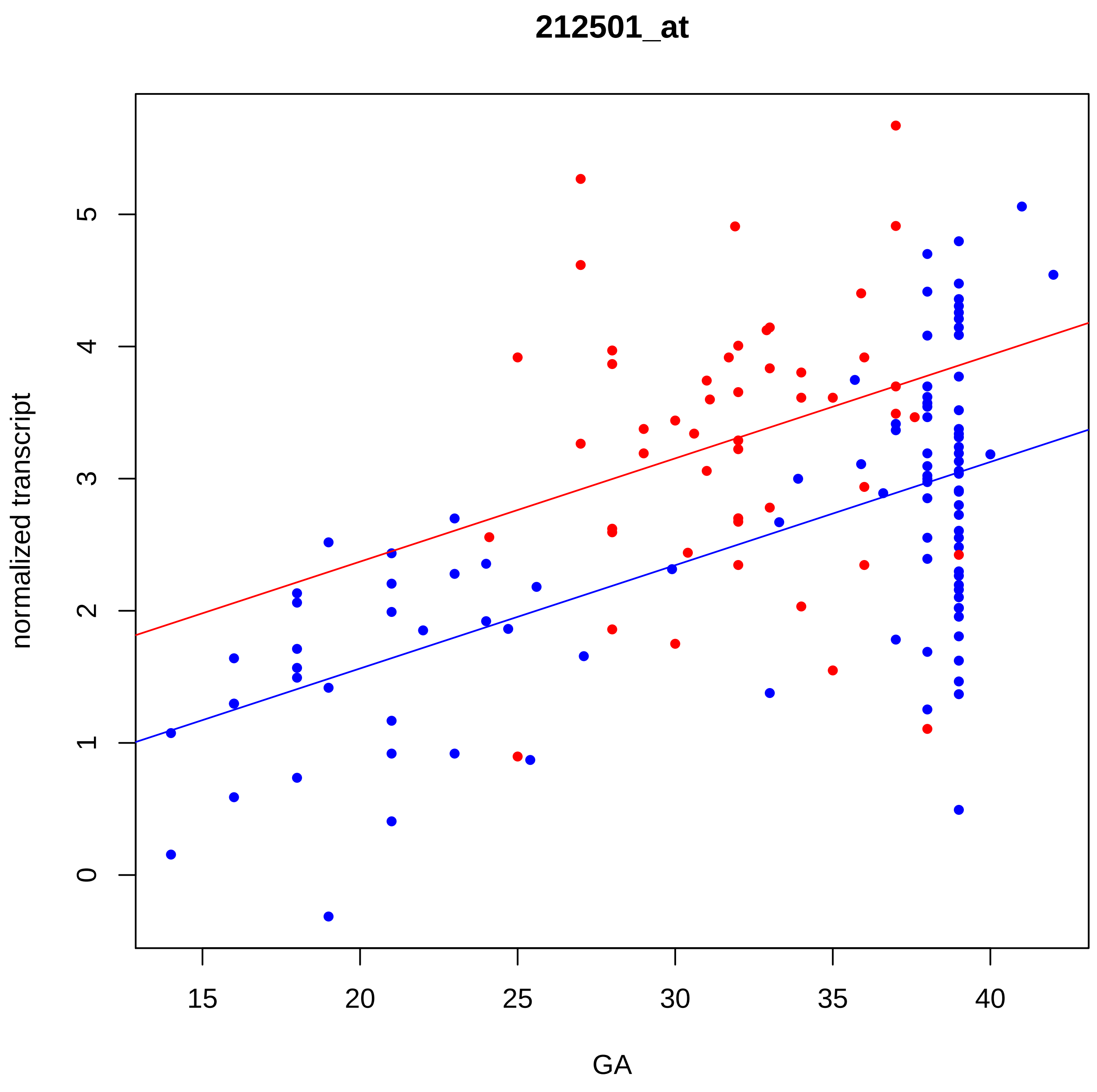
212501_at
normalized transcript
GA
0
1
2
3
4
5
15
20
25
30
35
40

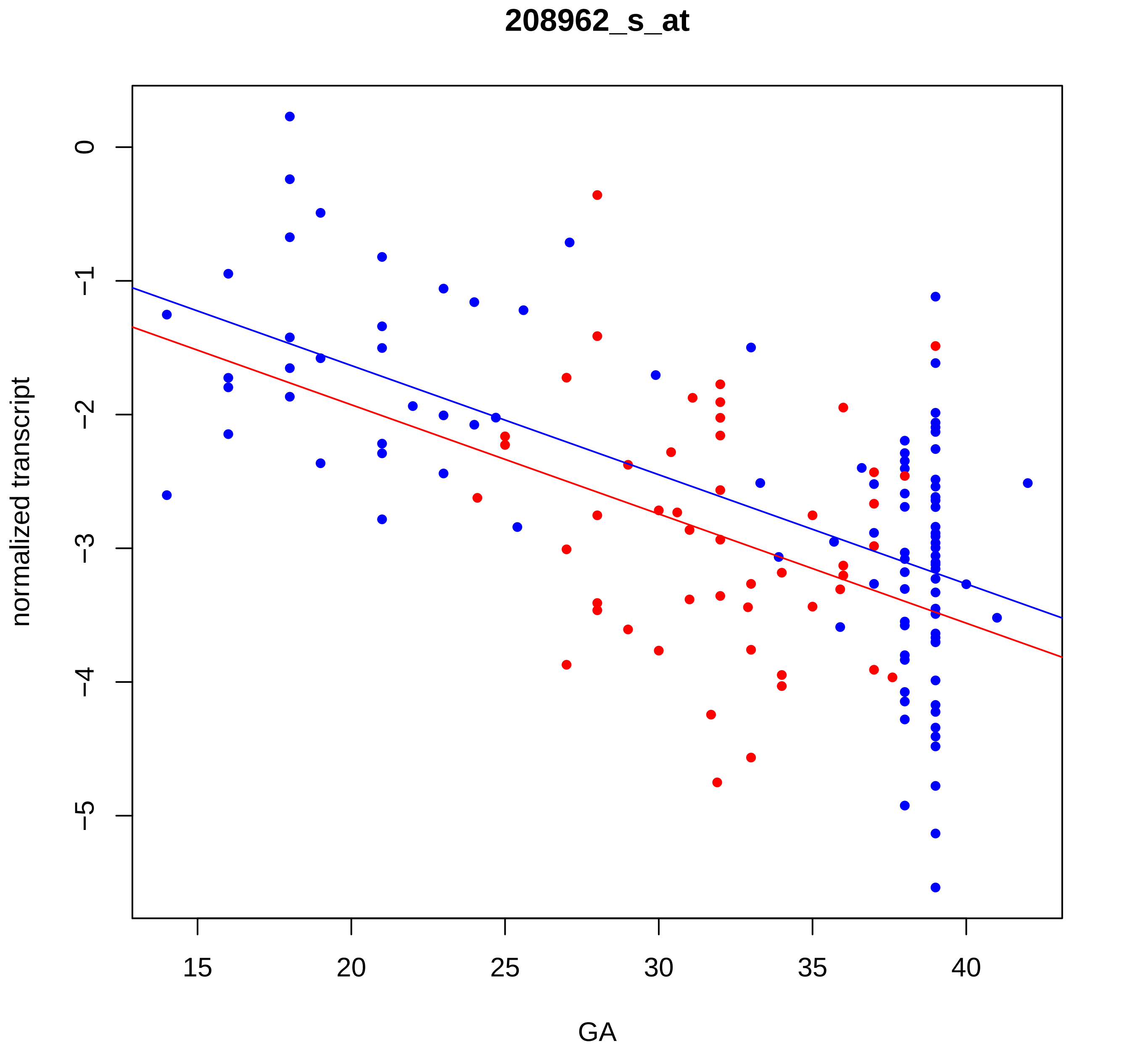

208962_s_at
0
−1
−2
−3
−4
−5
normalized transcript
15
20
25
30
35
40
GA

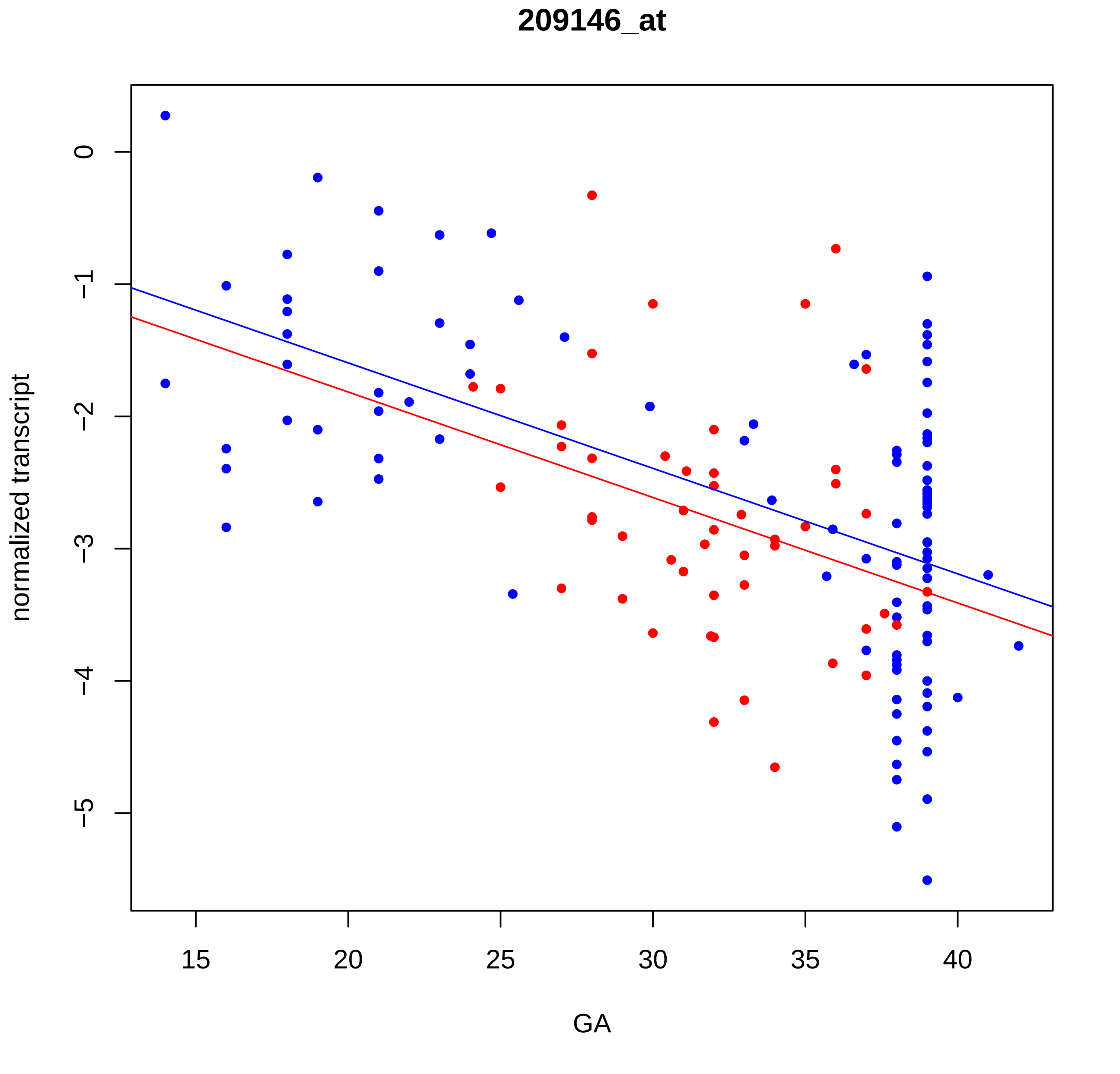

209146_at
normalized transcript
0
−1
−2
−3
−4
−5
15
20
25
30
35
40
GA

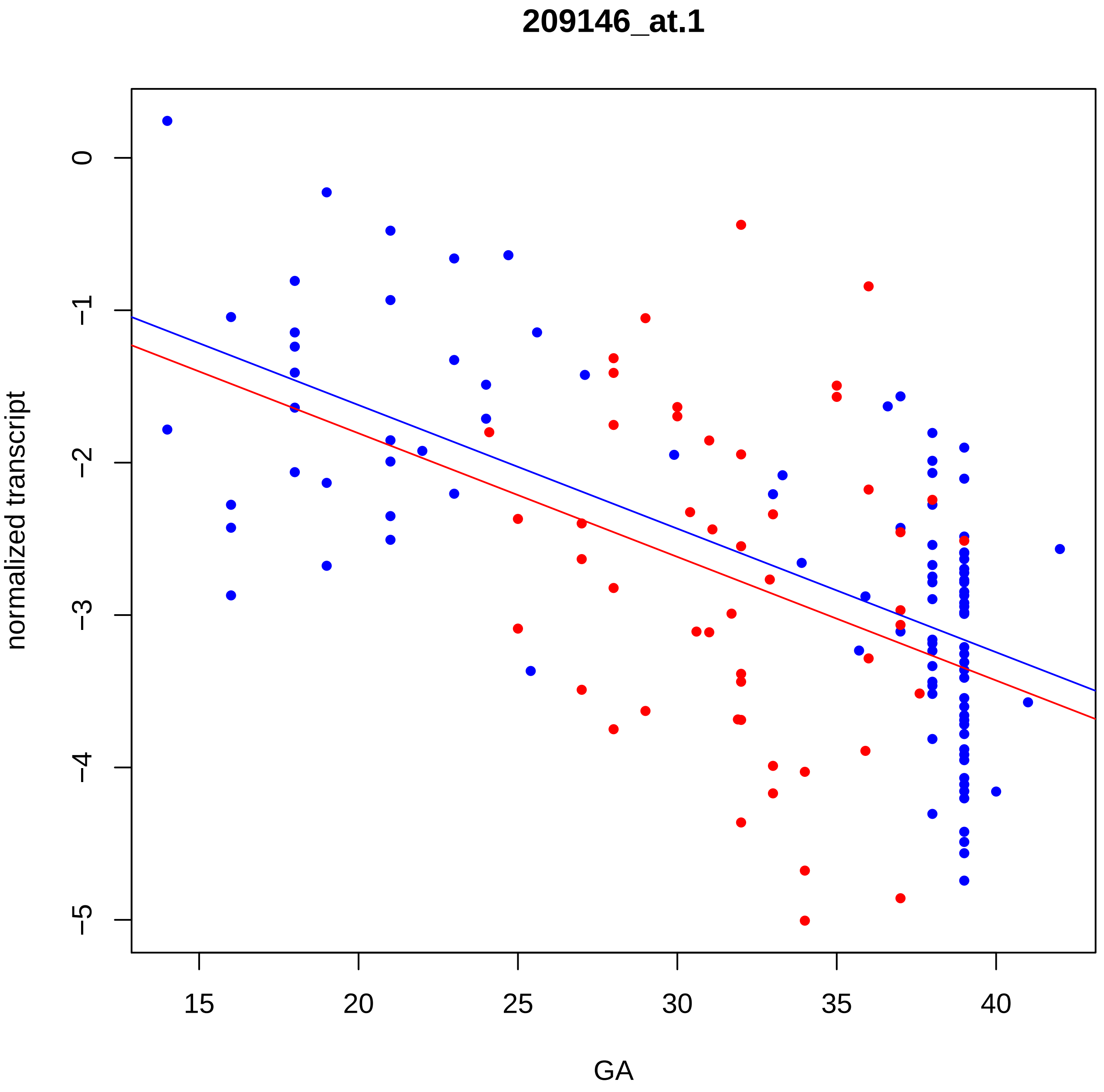

209146_at.1
normalized transcript
0
−1
−2
−3
−4
−5
15
20
25
30
35
40
GA

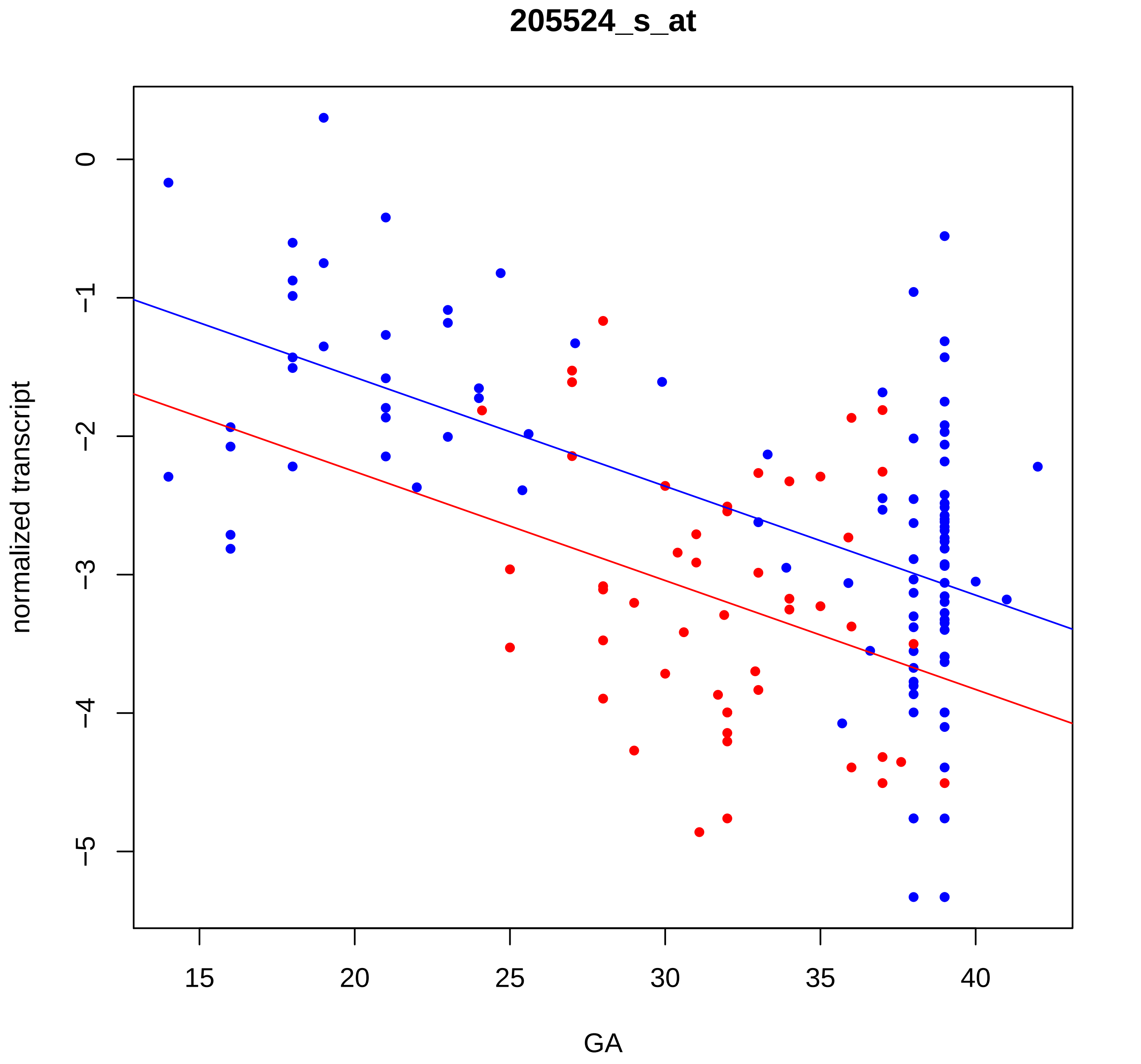

205524_s_at
0
−1
−2
−3
−4
−5
normalized transcript
15
20
25
30
35
40
GA

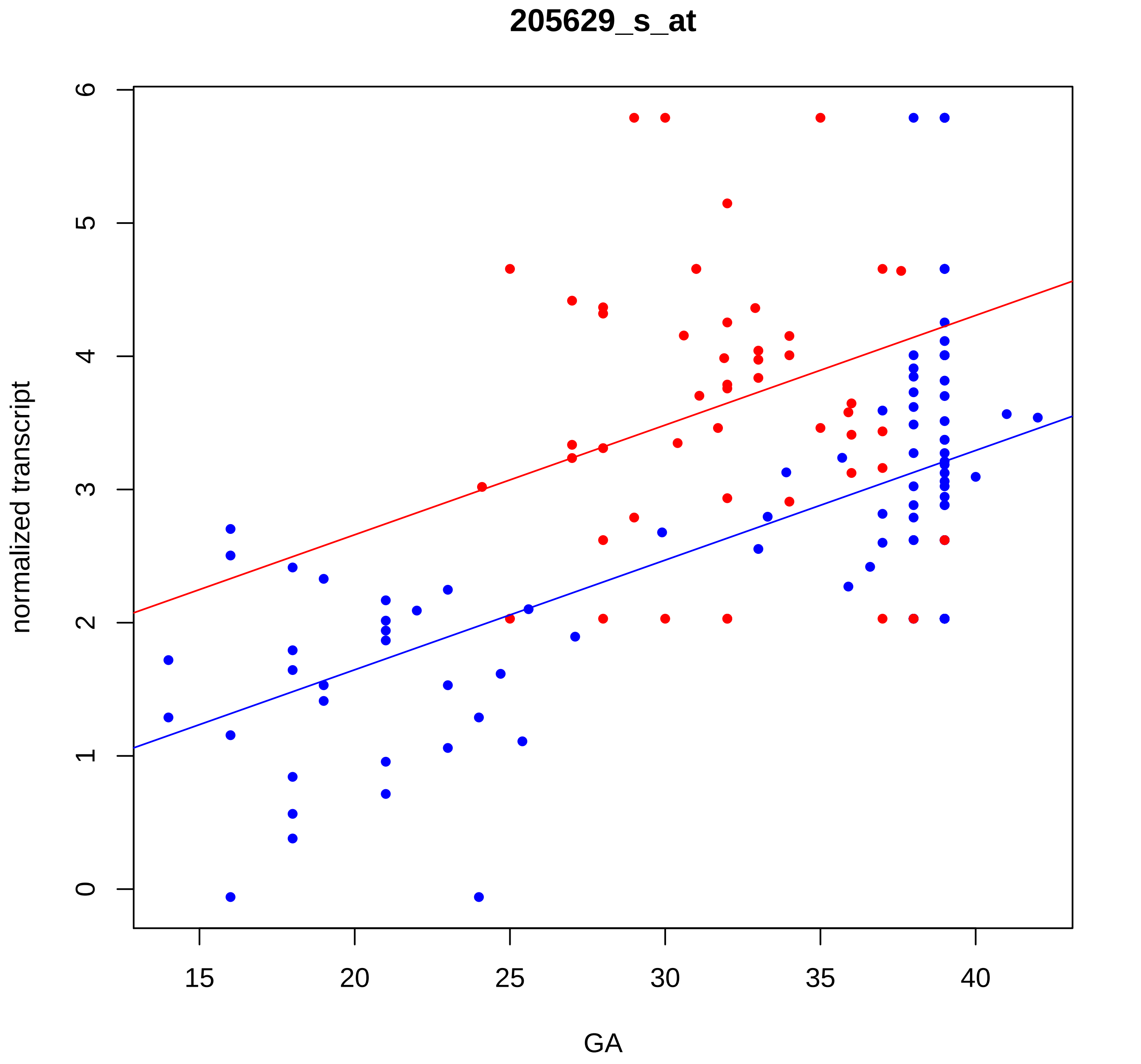

205629_s_at
normalized transcript
GA
0
1
2
3
4
5
6
15
20
25
30
35
40

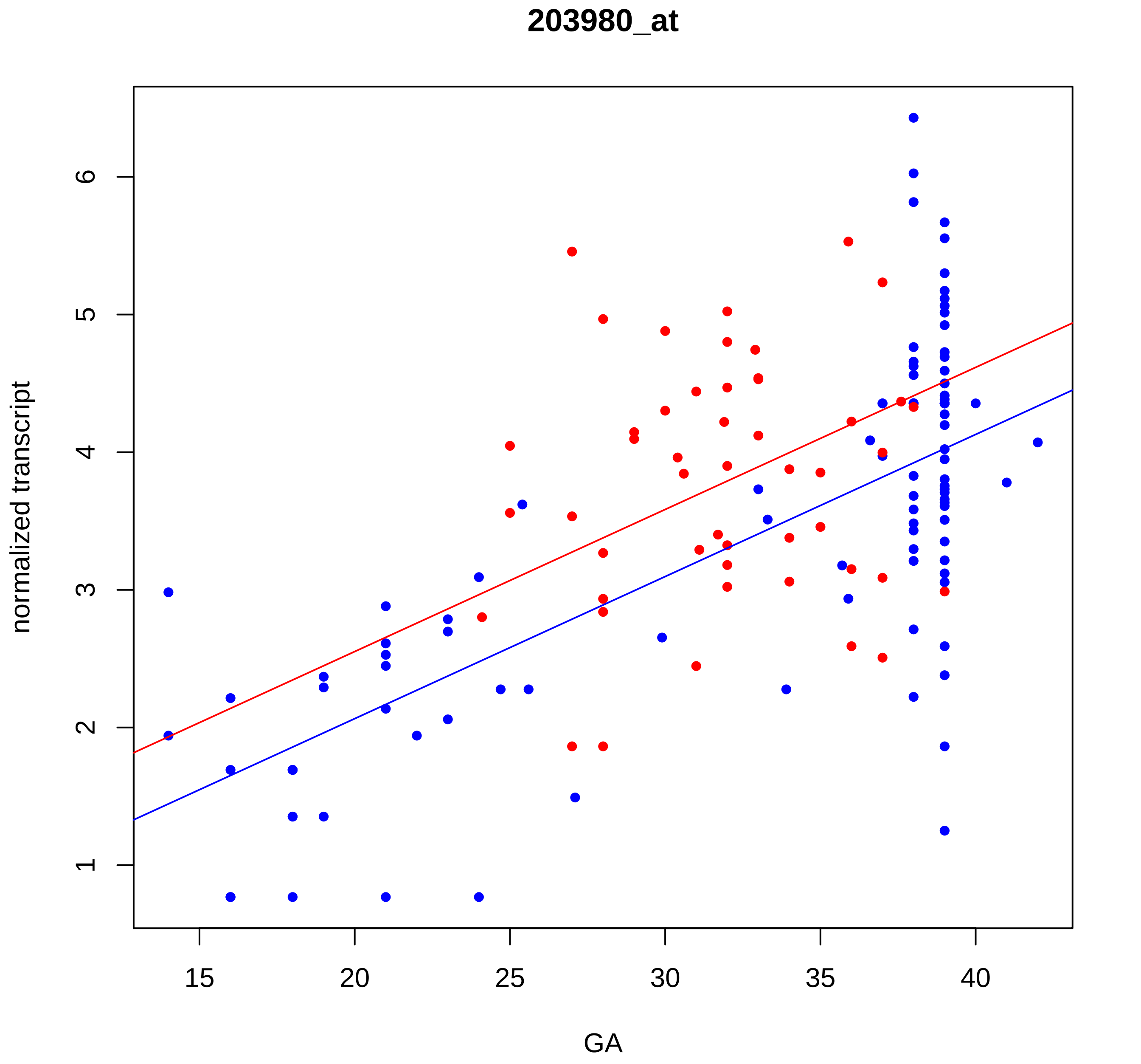

203980_at
normalized transcript
GA
1
2
3
4
5
6
15
20
25
30
35
40

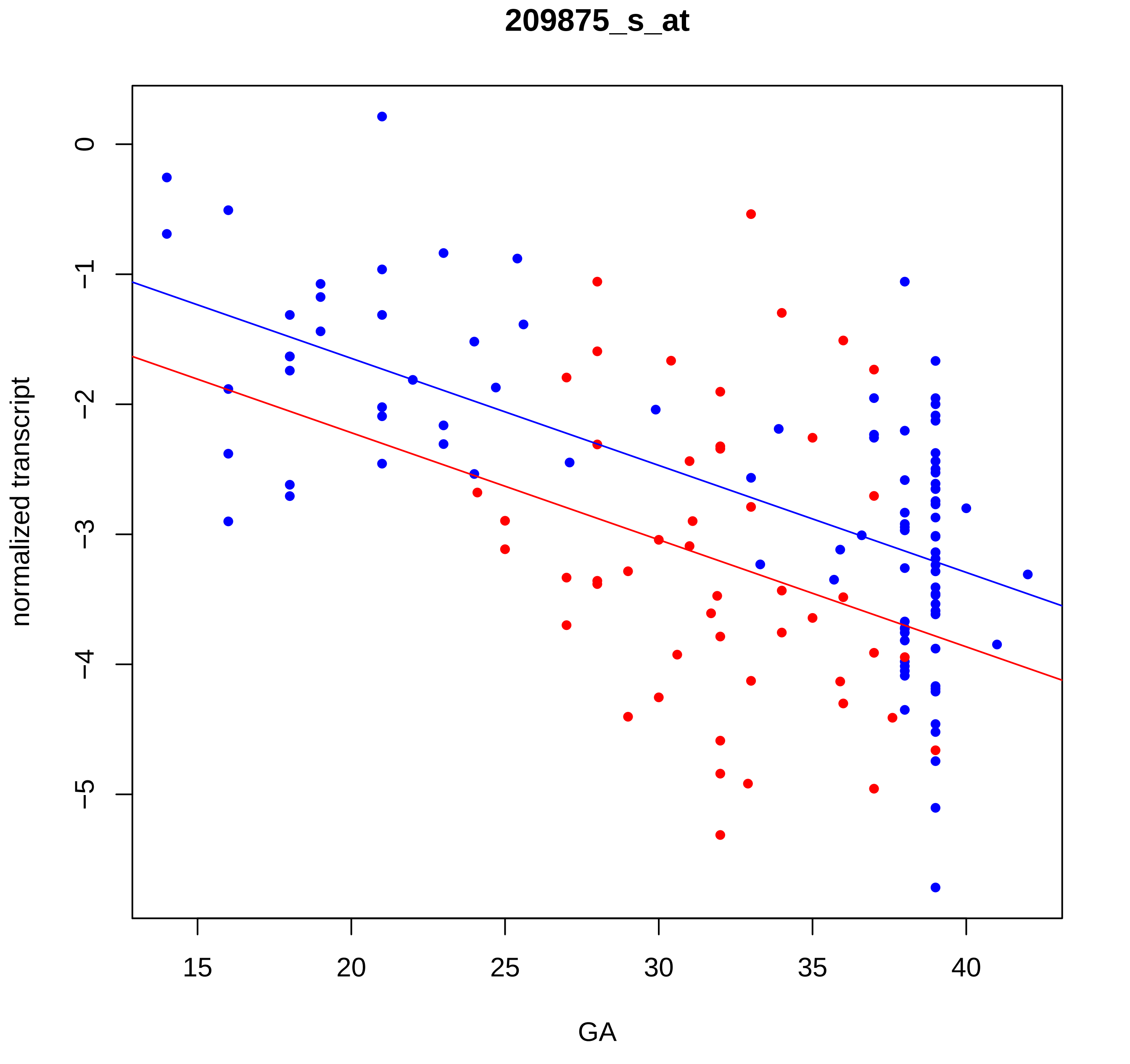

209875_s_at
normalized transcript
0
−1
−2
−3
−4
−5
15
20
25
30
35
40
GA

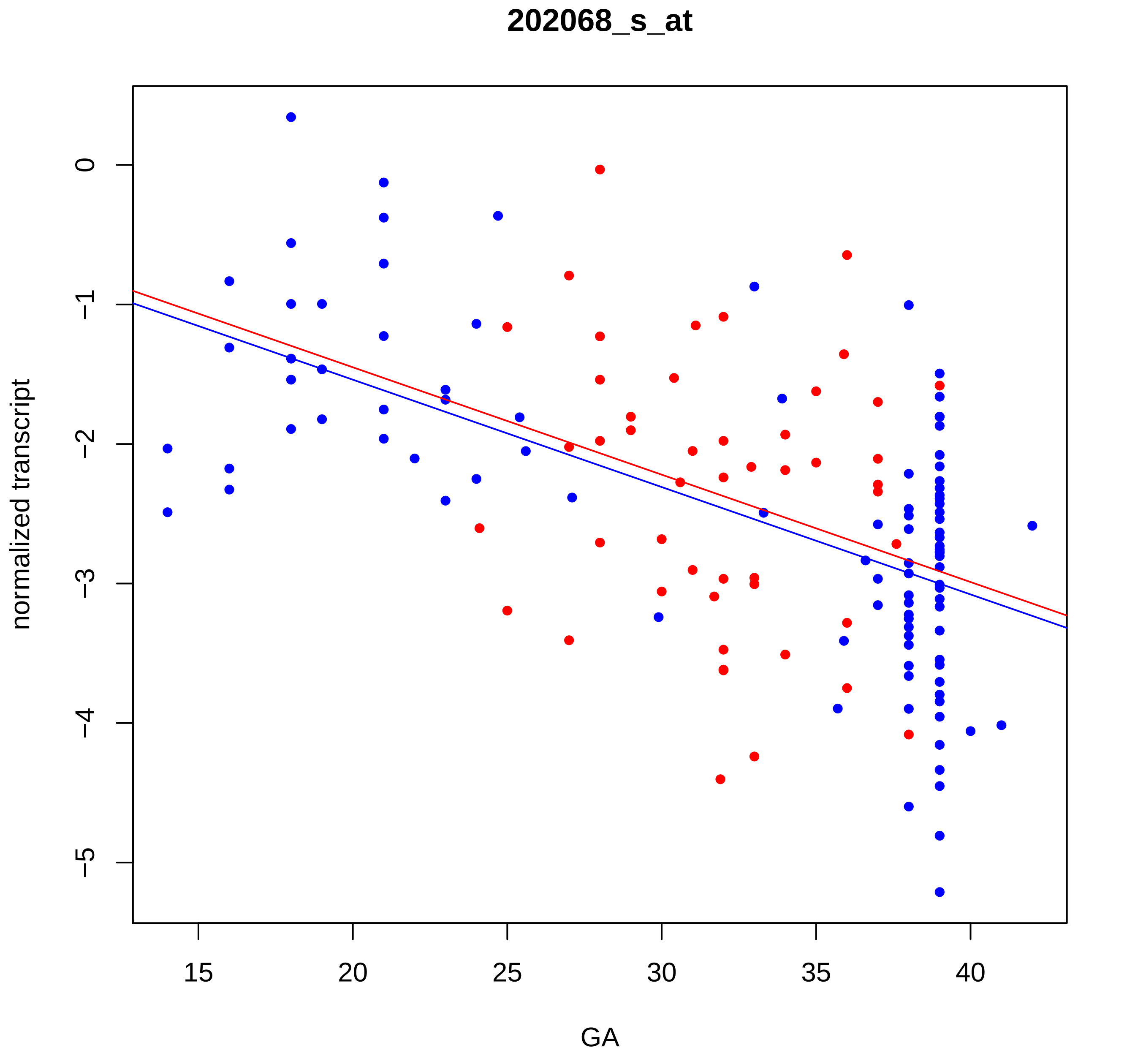
202068_s_at
normalized transcript
GA
0
−1
−2
−3
−4
−5
15
20
25
30
35
40

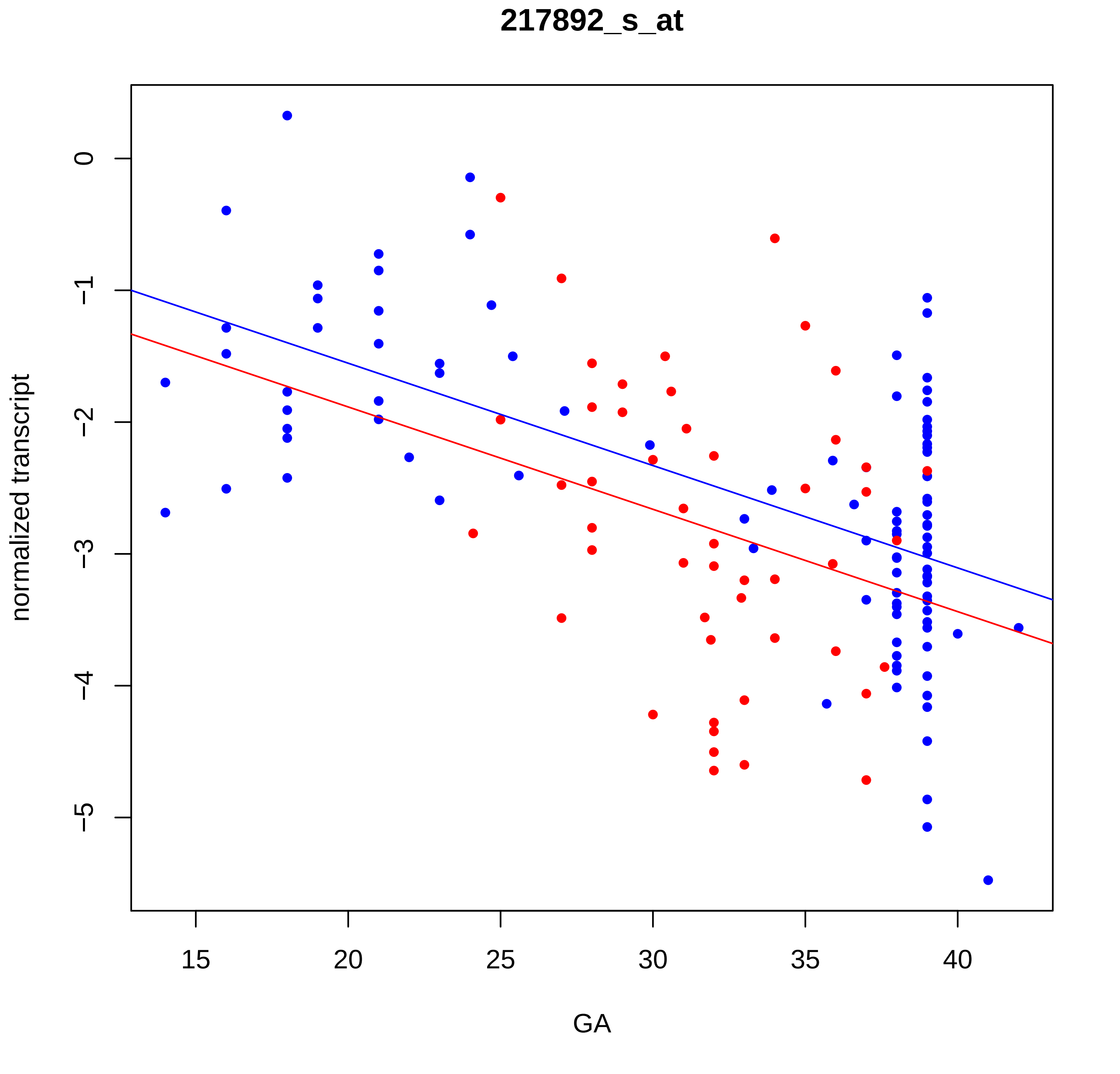
217892_s_at
normalized transcript
GA
0
−1
−2
−3
−4
−5
15
20
25
30
35
40

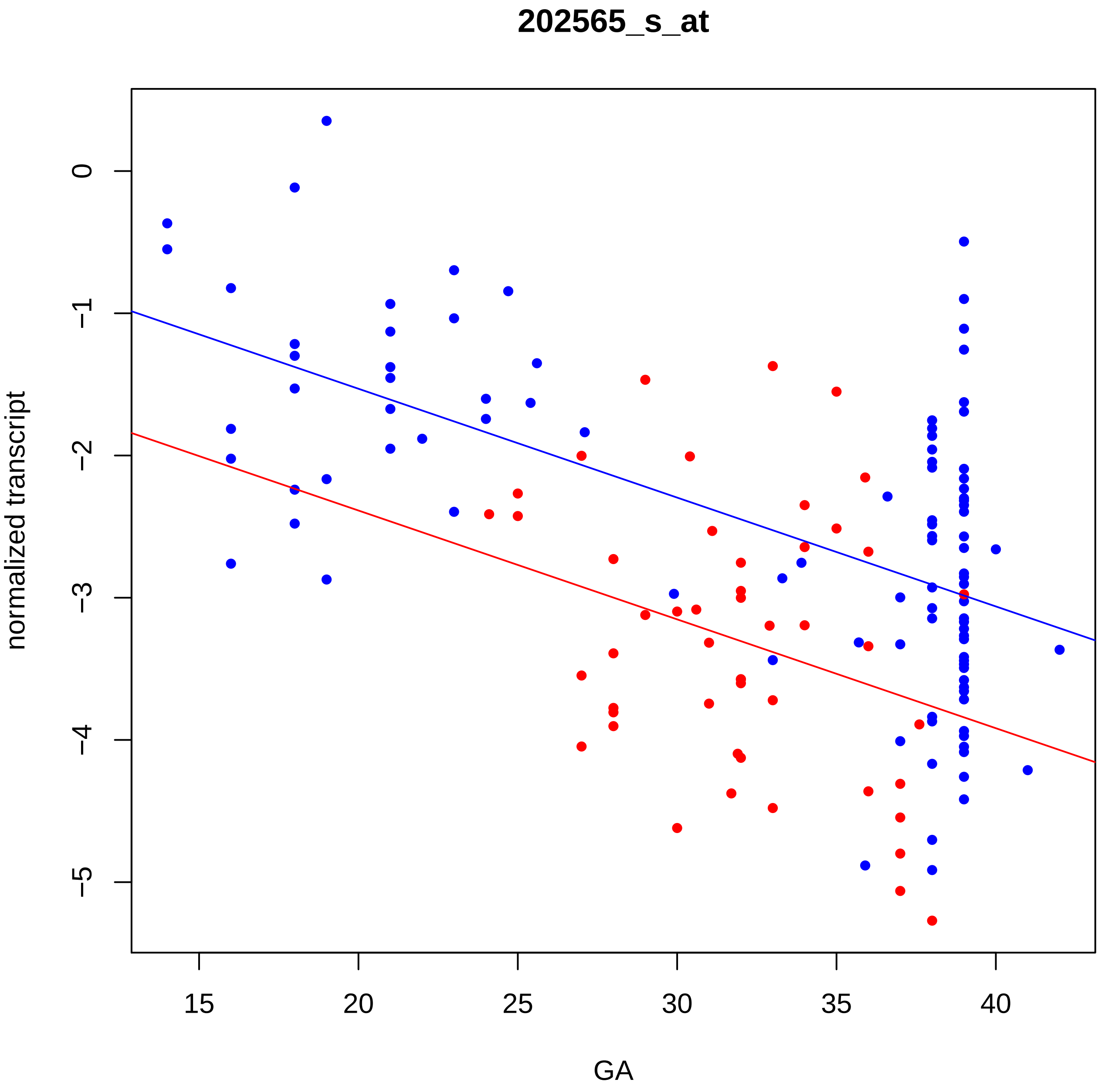
202565_s_at
normalized transcript
GA
0
−1
−2
−3
−4
−5
15
20
25
30
35
40

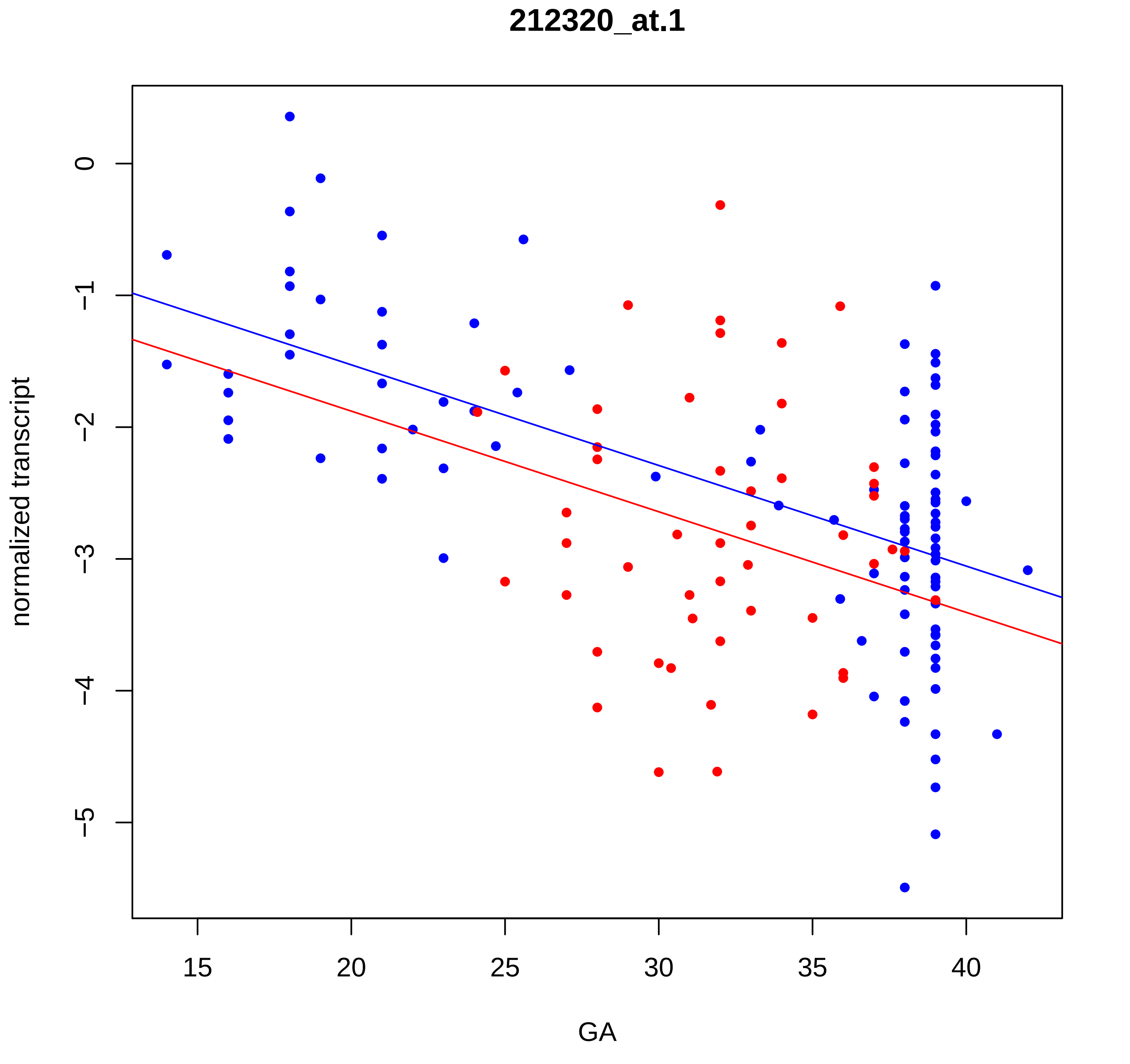

212320_at.1
normalized transcript
GA
0
−1
−2
−3
−4
−5
15
20
25
30
35
40

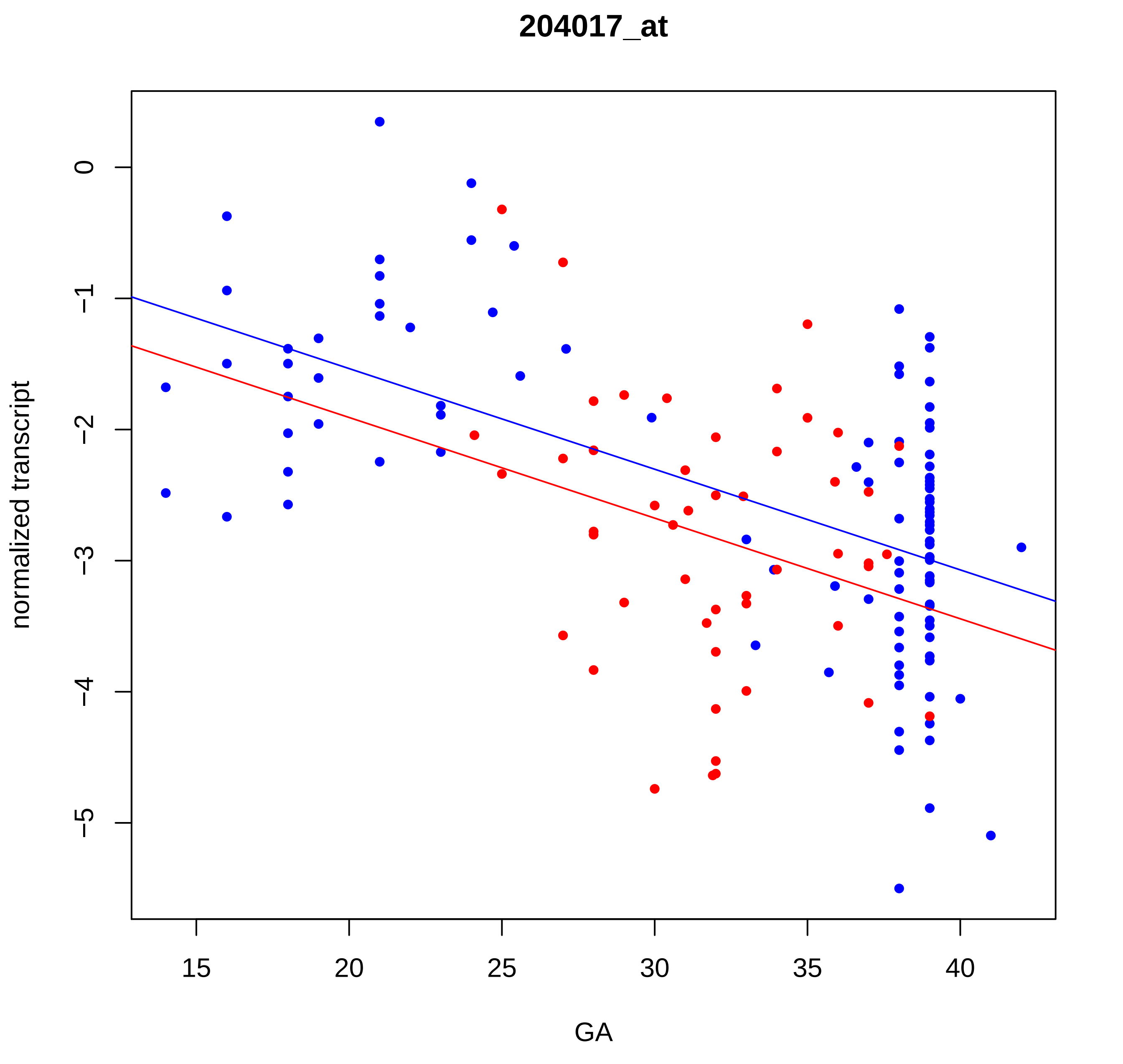
204017_at
normalized transcript
GA
0
−1
−2
−3
−4
−5
15
20
25
30
35
40

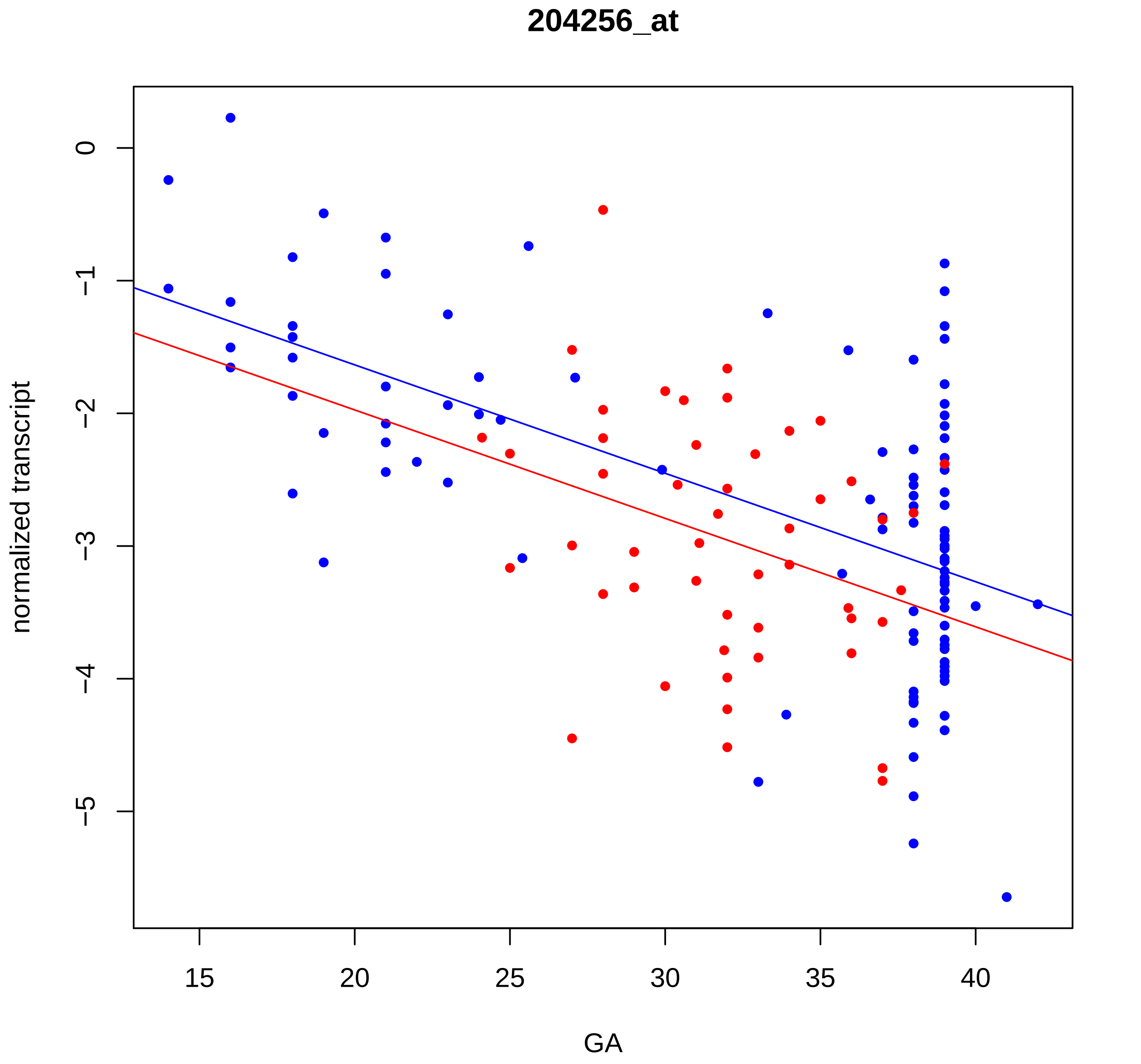

204256_at
0
−1
−2
−3
−4
−5
normalized transcript
15
20
25
30
35
40
GA

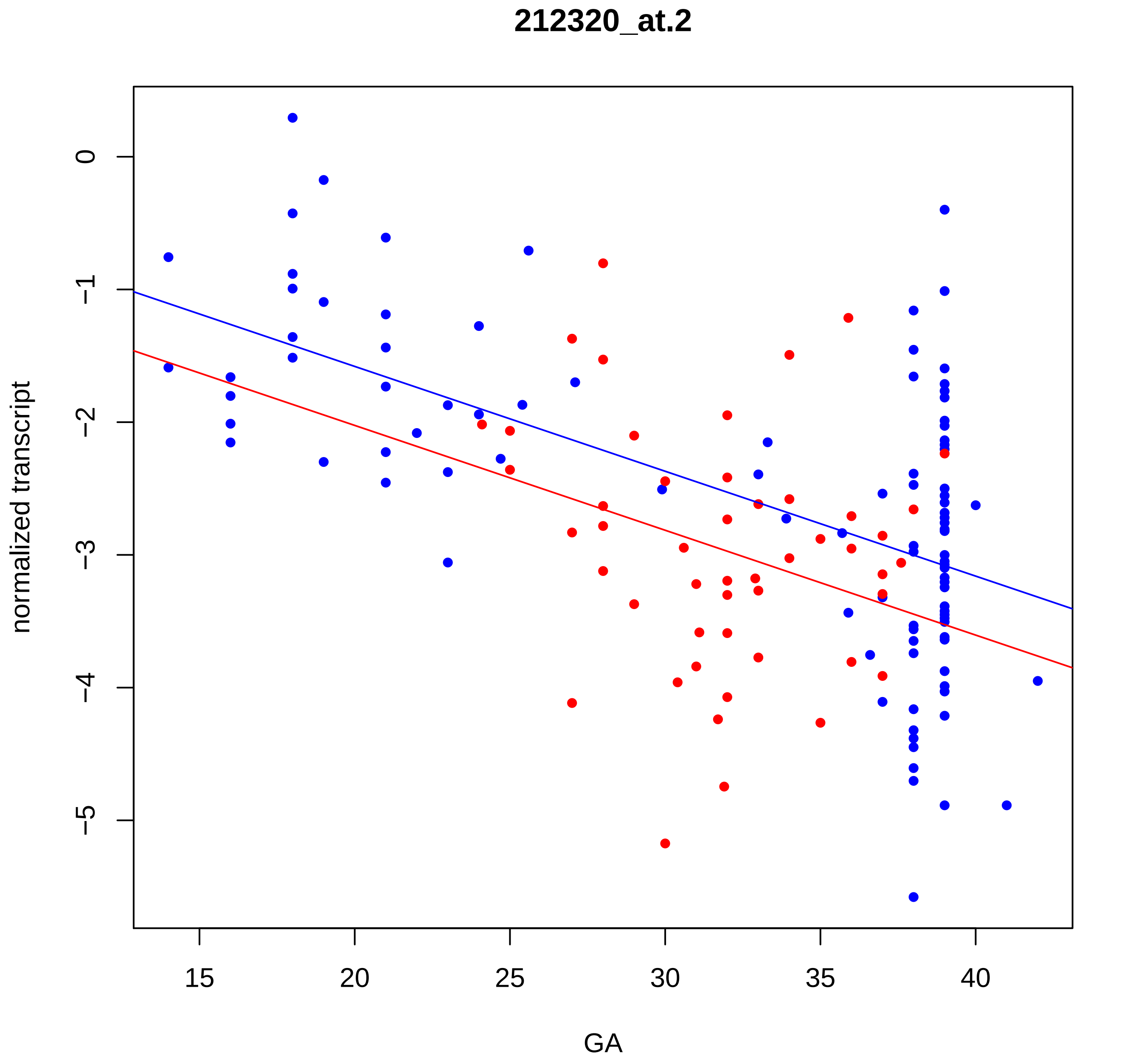
212320_at.2
normalized transcript
GA
0
−1
−2
−3
−4
−5
15
20
25
30
35
40

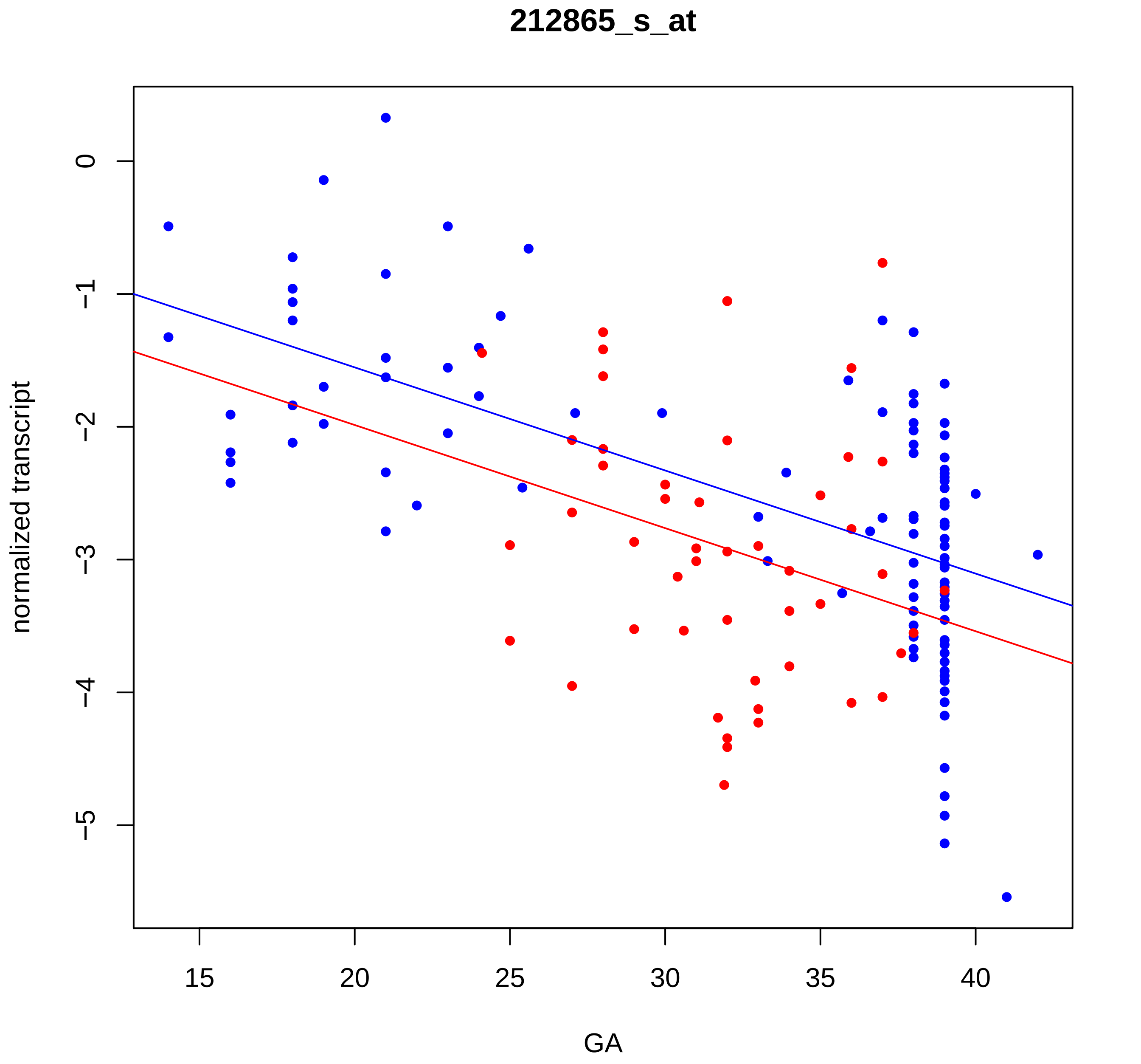

212865_s_at
0
−1
−2
−3
−4
−5
normalized transcript
15
20
25
30
35
40
GA

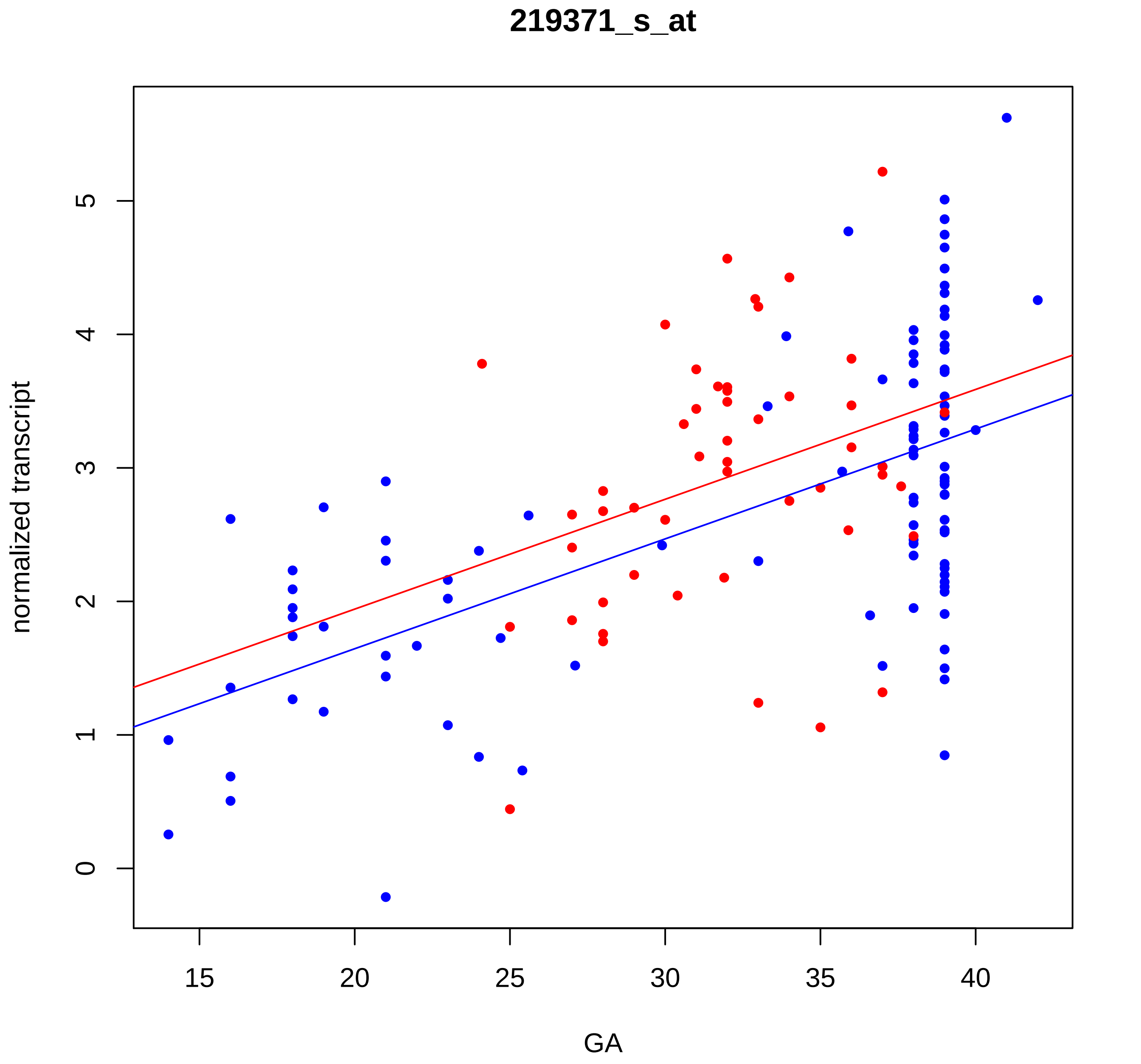

219371_s_at
normalized transcript
GA
0
1
2
3
4
5
15
20
25
30
35
40

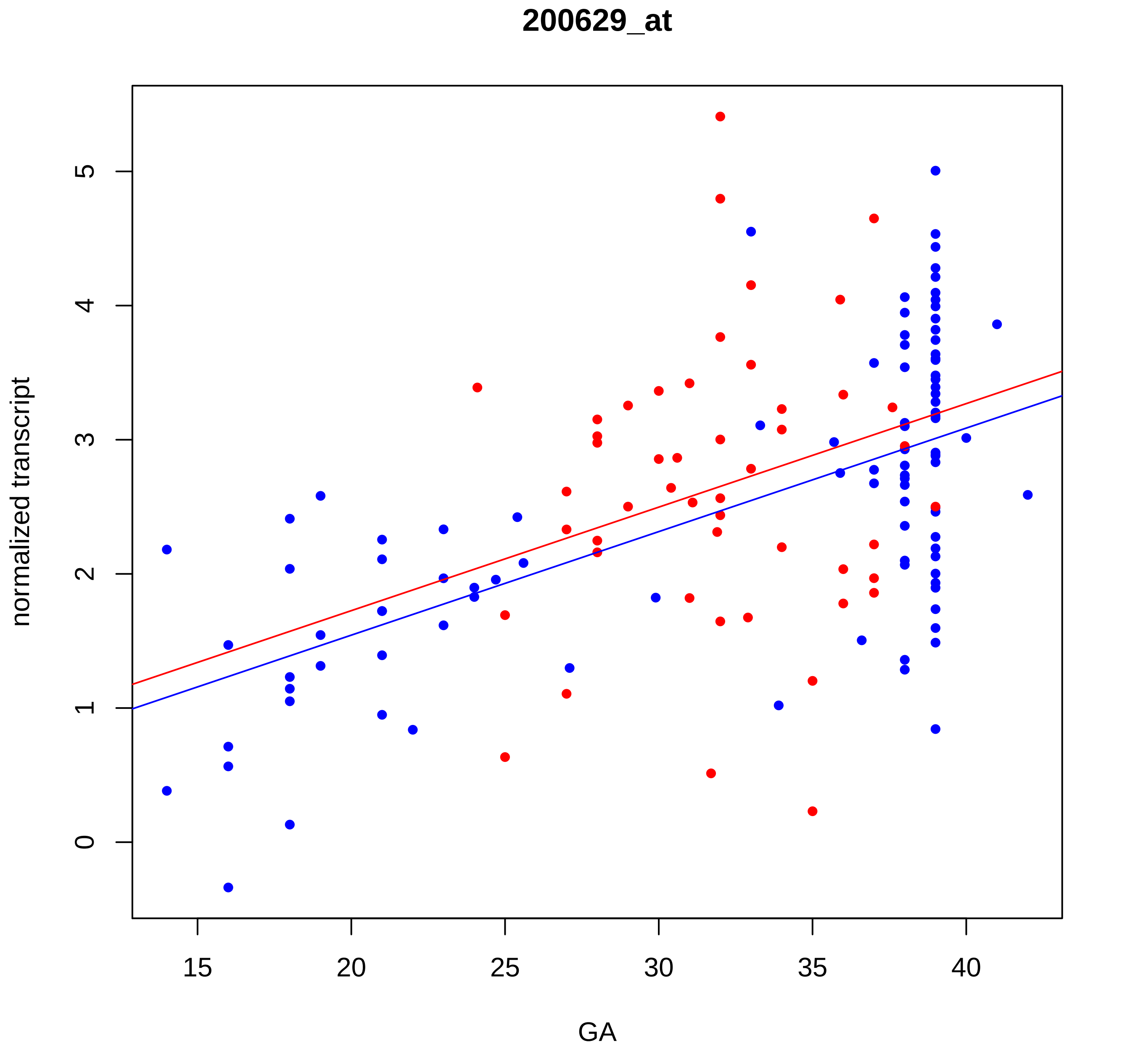

200629_at
normalized transcript
GA
0
1
2
3
4
5
15
20
25
30
35
40

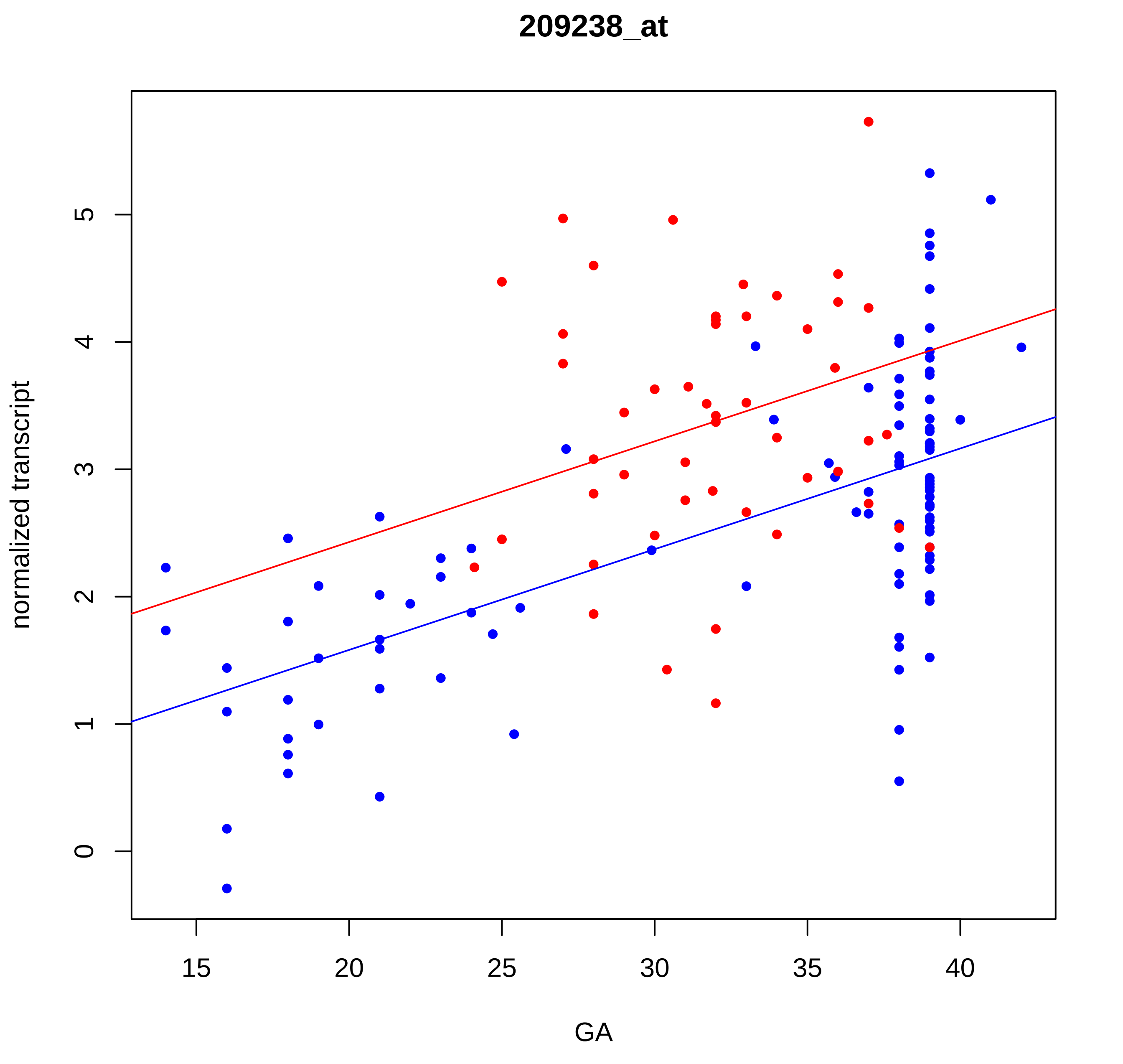

209238_at
normalized transcript
GA
0
1
2
3
4
5
15
20
25
30
35
40

201576_s_at

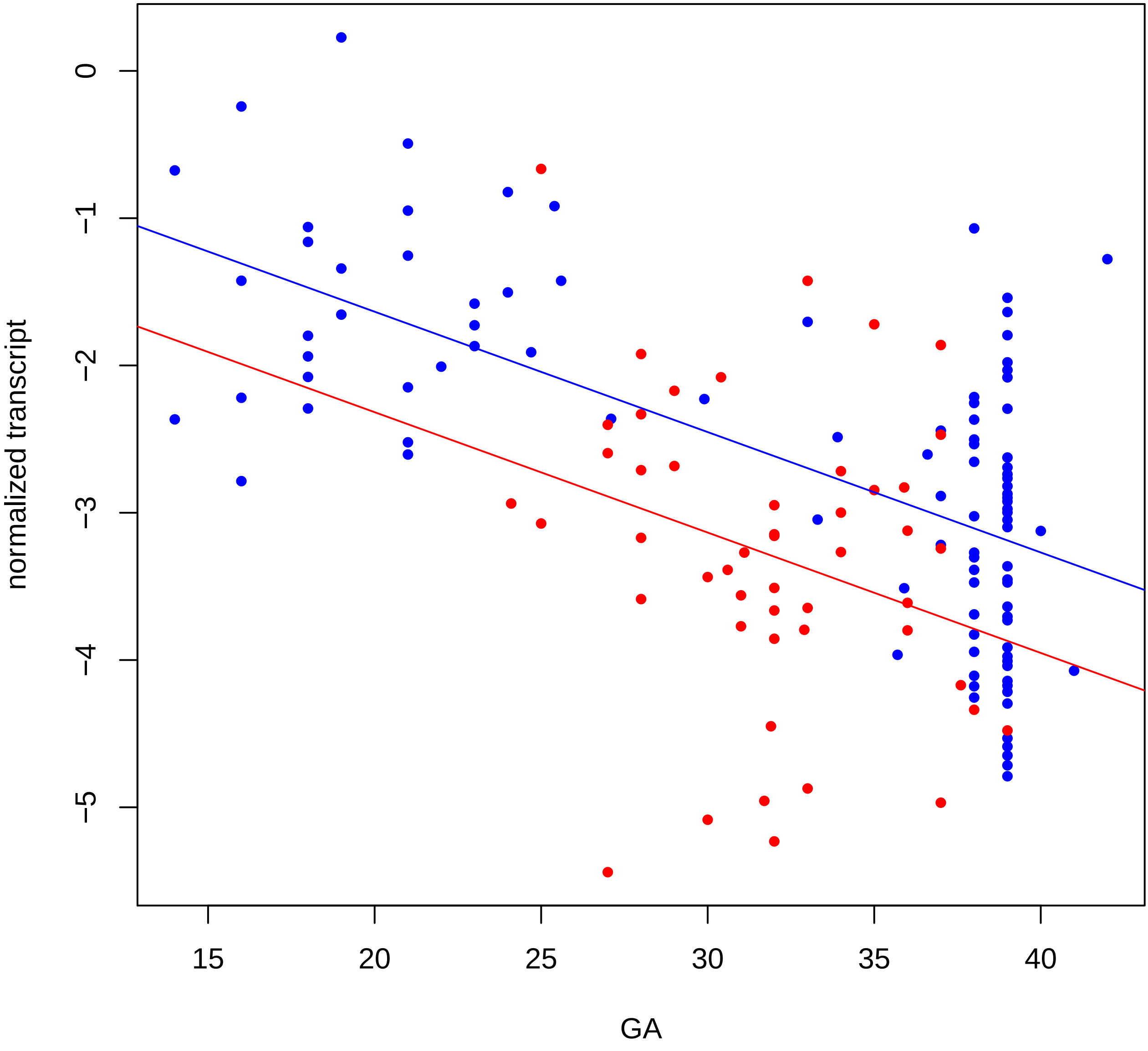

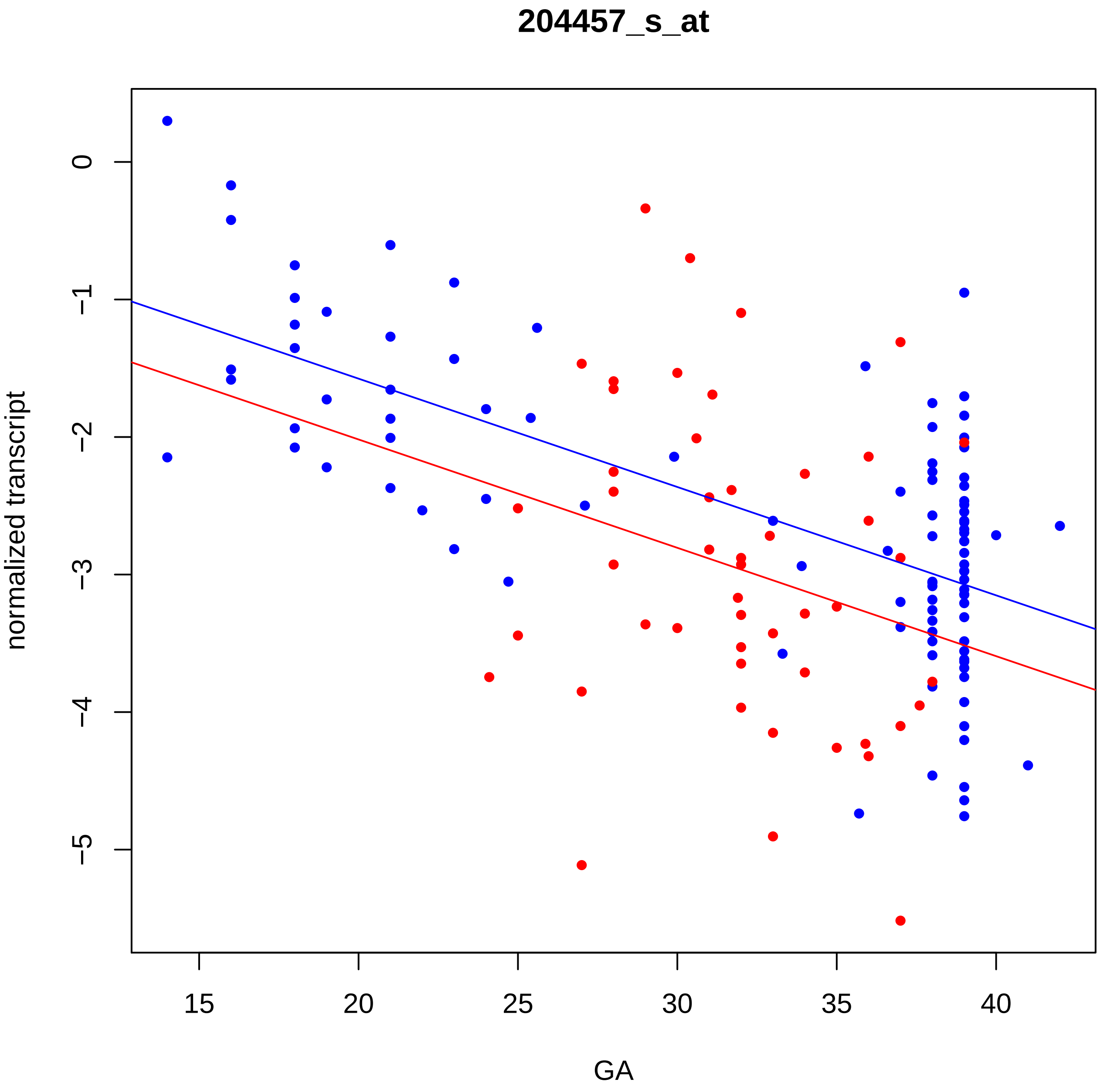

204457_s_at
normalized transcript
0
−1
−2
−3
−4
−5
15
20
25
30
35
40
GA

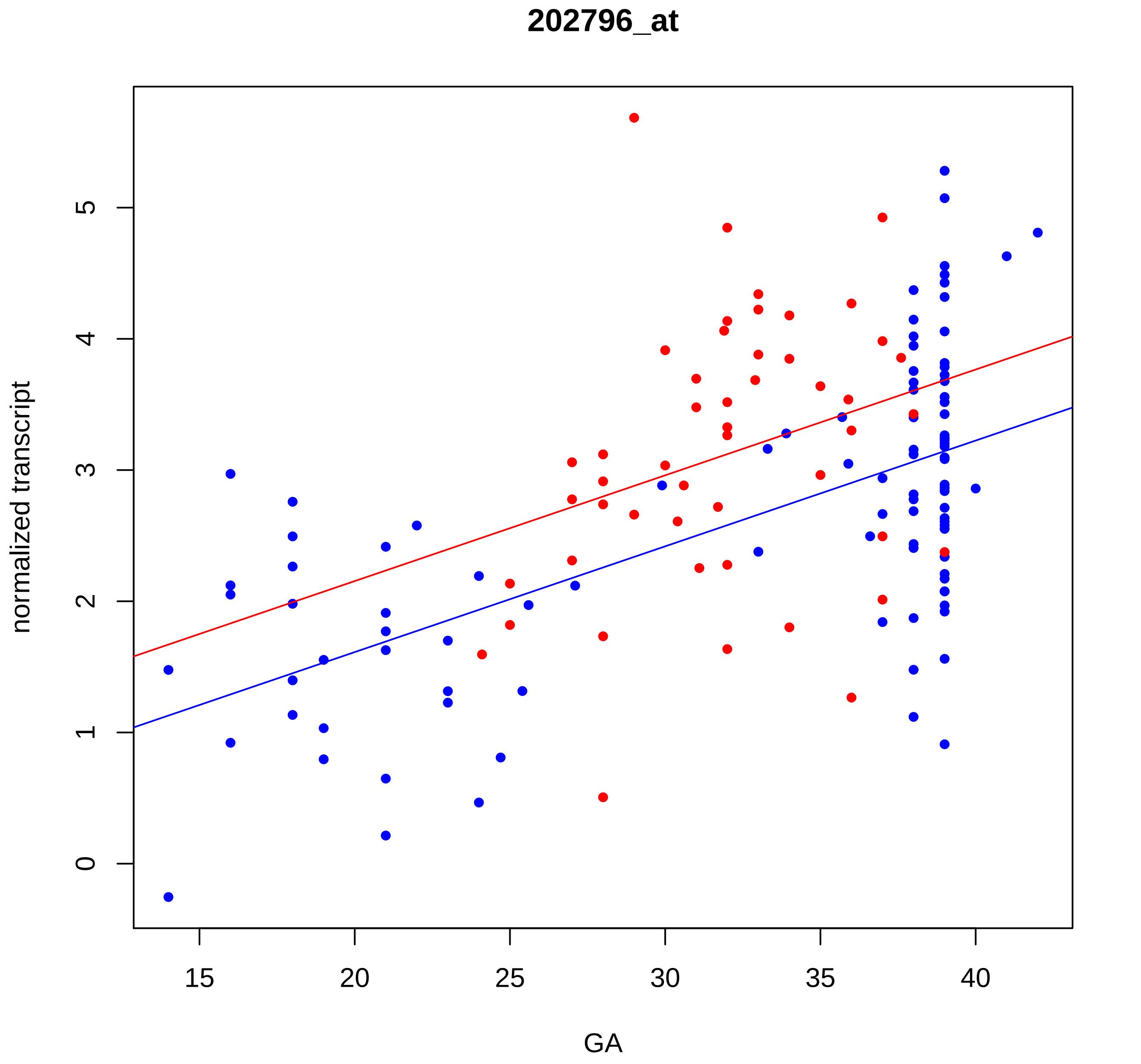

202796_at
normalized transcript
GA
0
1
2
3
4
5
15
20
25
30
35
40

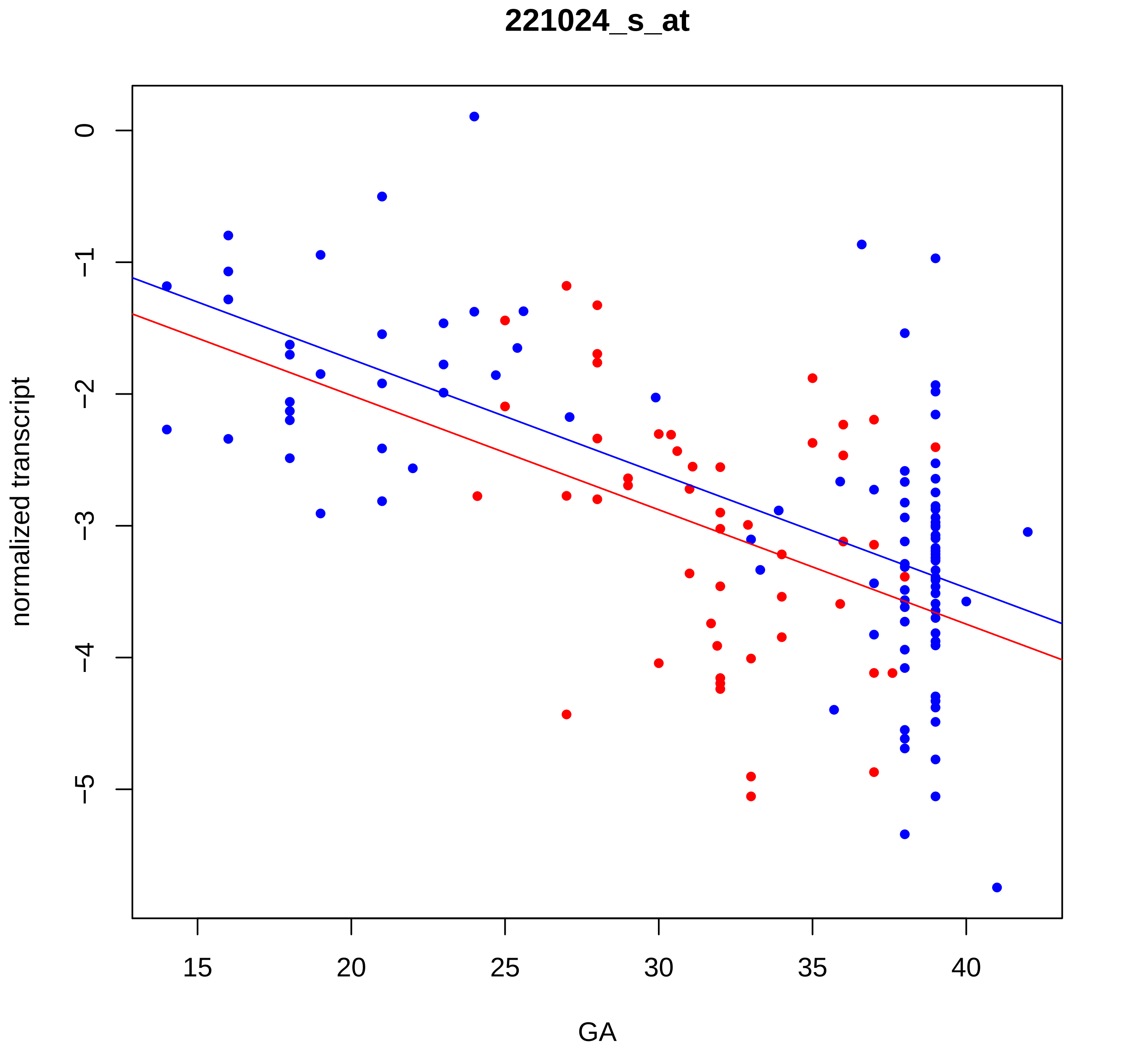
221024_s_at
normalized transcript
GA
0
−1
−2
−3
−4
−5
15
20
25
30
35
40

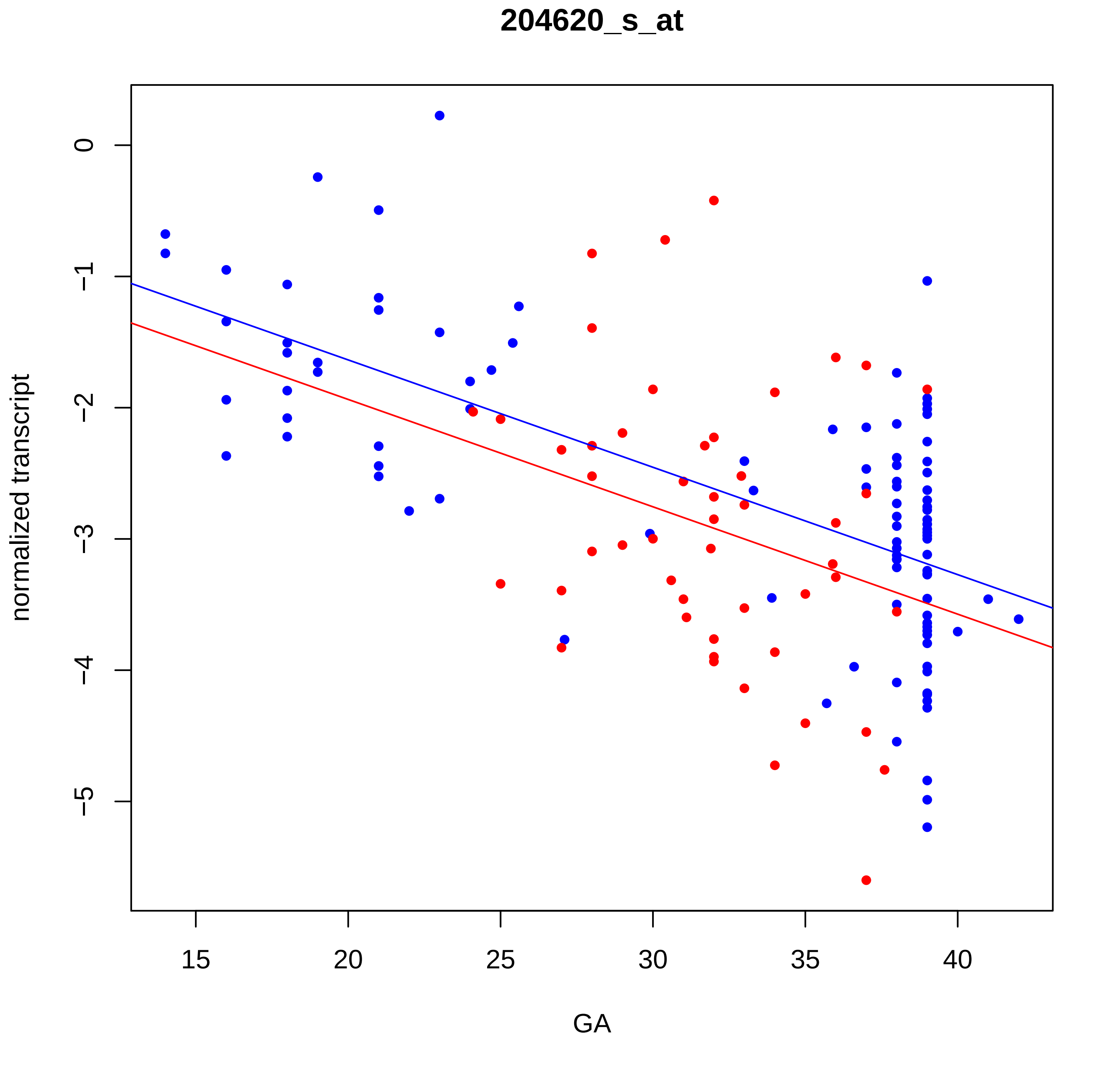
204620_s_at
normalized transcript
0
−1
−2
−3
−4
−5
15
20
25
30
35
40
GA

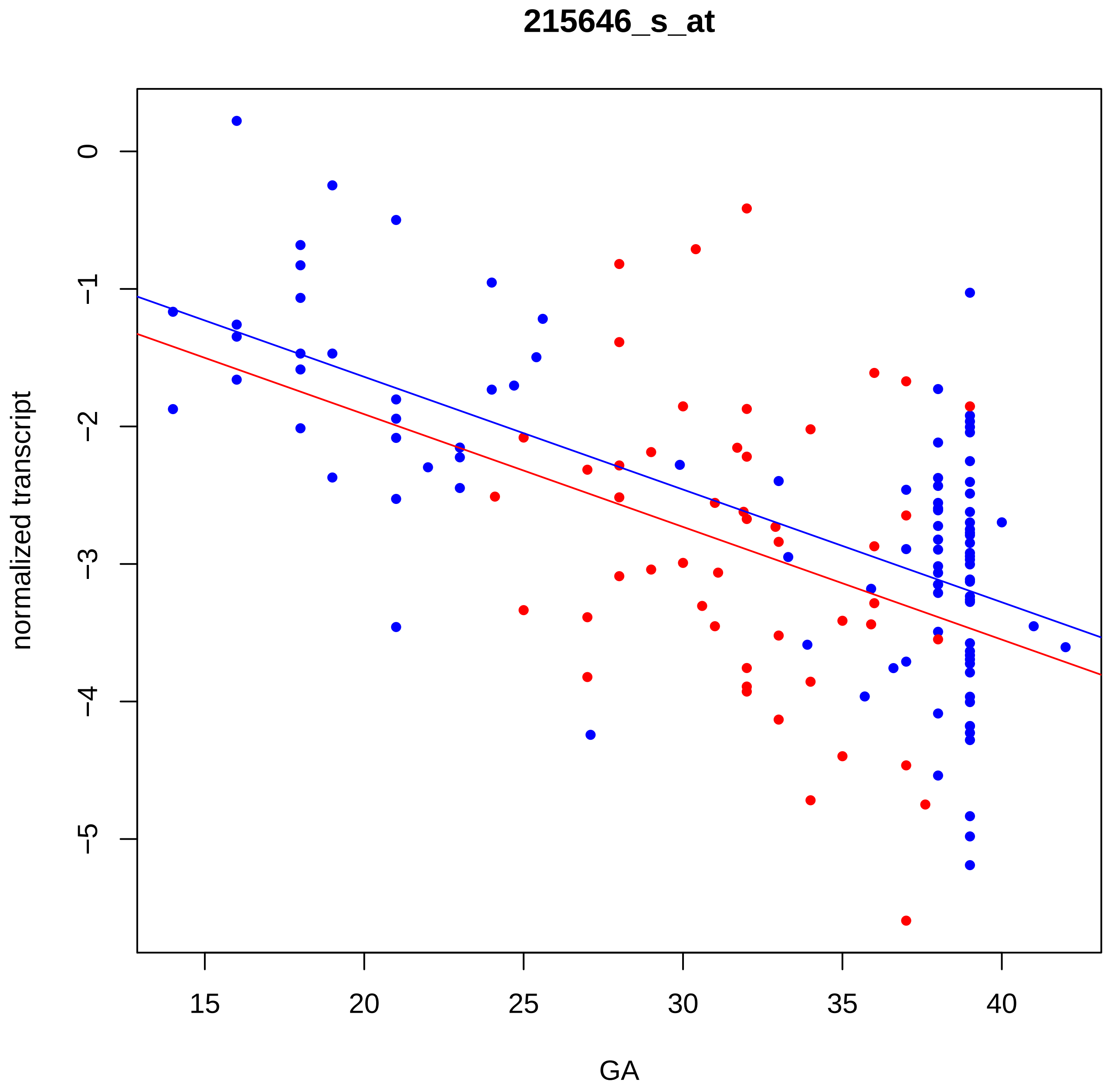

215646_s_at
normalized transcript
GA
0
−1
−2
−3
−4
−5
15
20
25
30
35
40

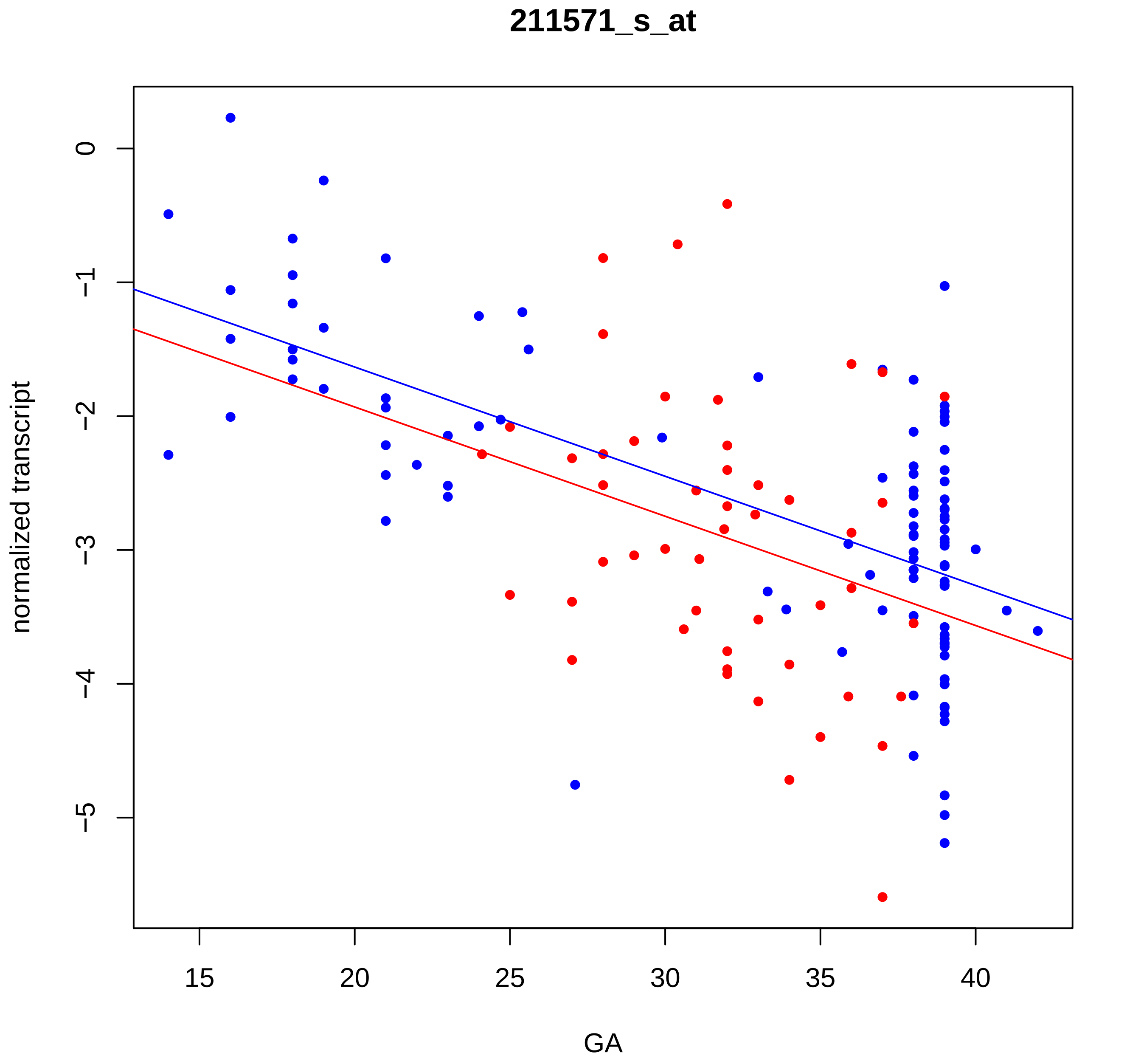

211571_s_at
0
−1
−2
−3
−4
−5
normalized transcript
15
20
25
30
35
40
GA

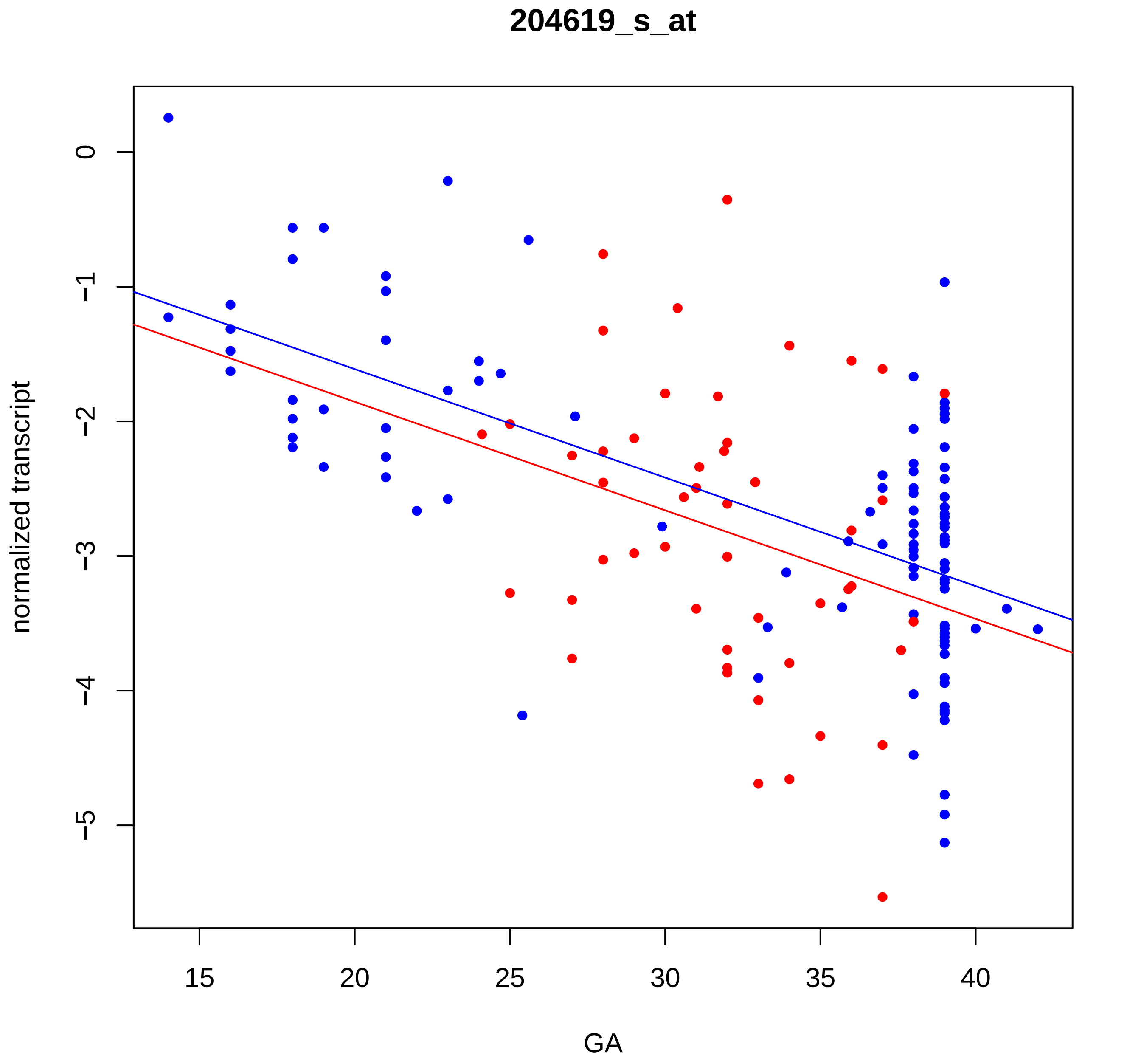

204619_s_at
0
−1
−2
−3
−4
−5
normalized transcript
15
20
25
30
35
40
GA

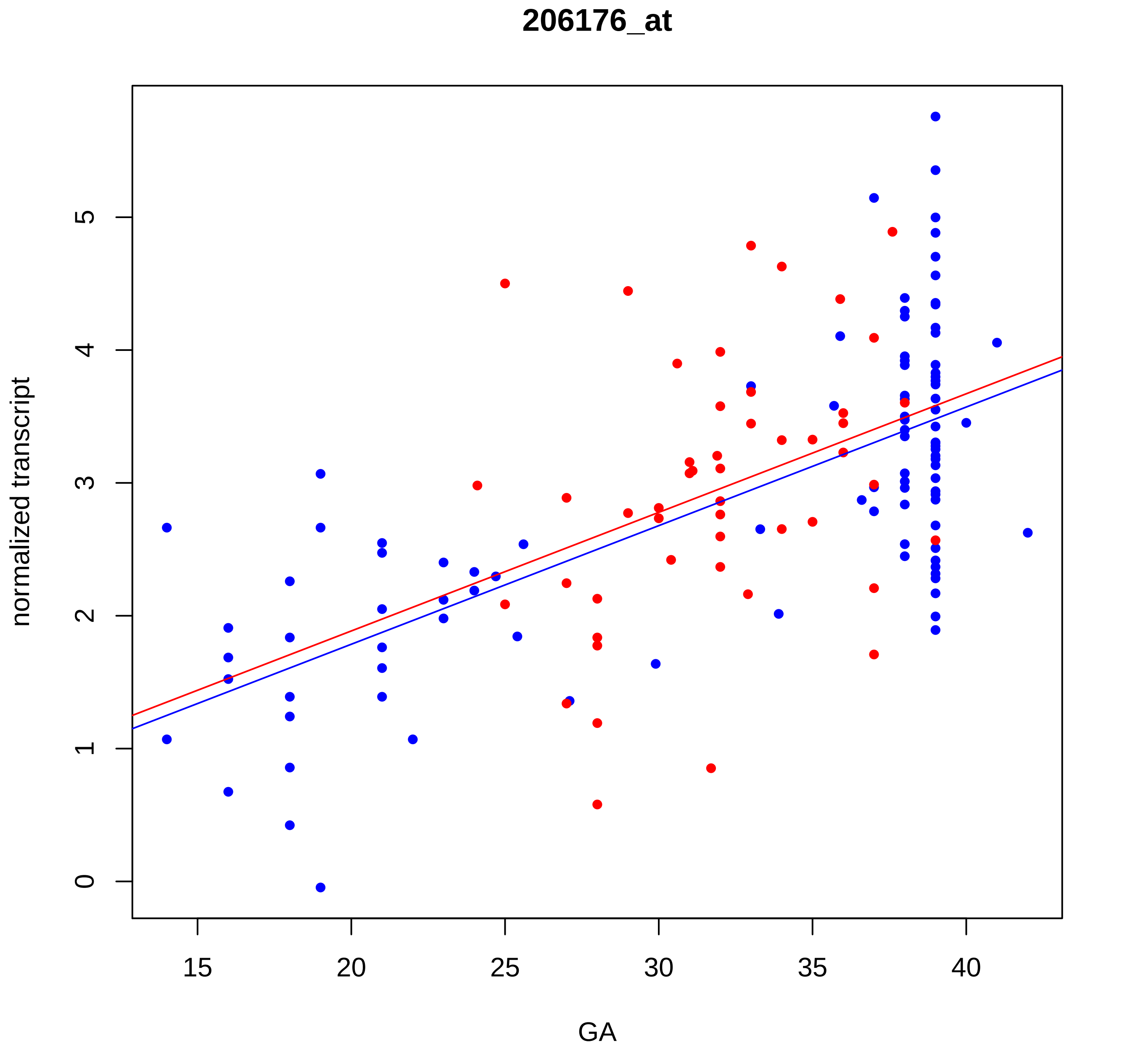

206176_at
normalized transcript
GA
0
1
2
3
4
5
15
20
25
30
35
40

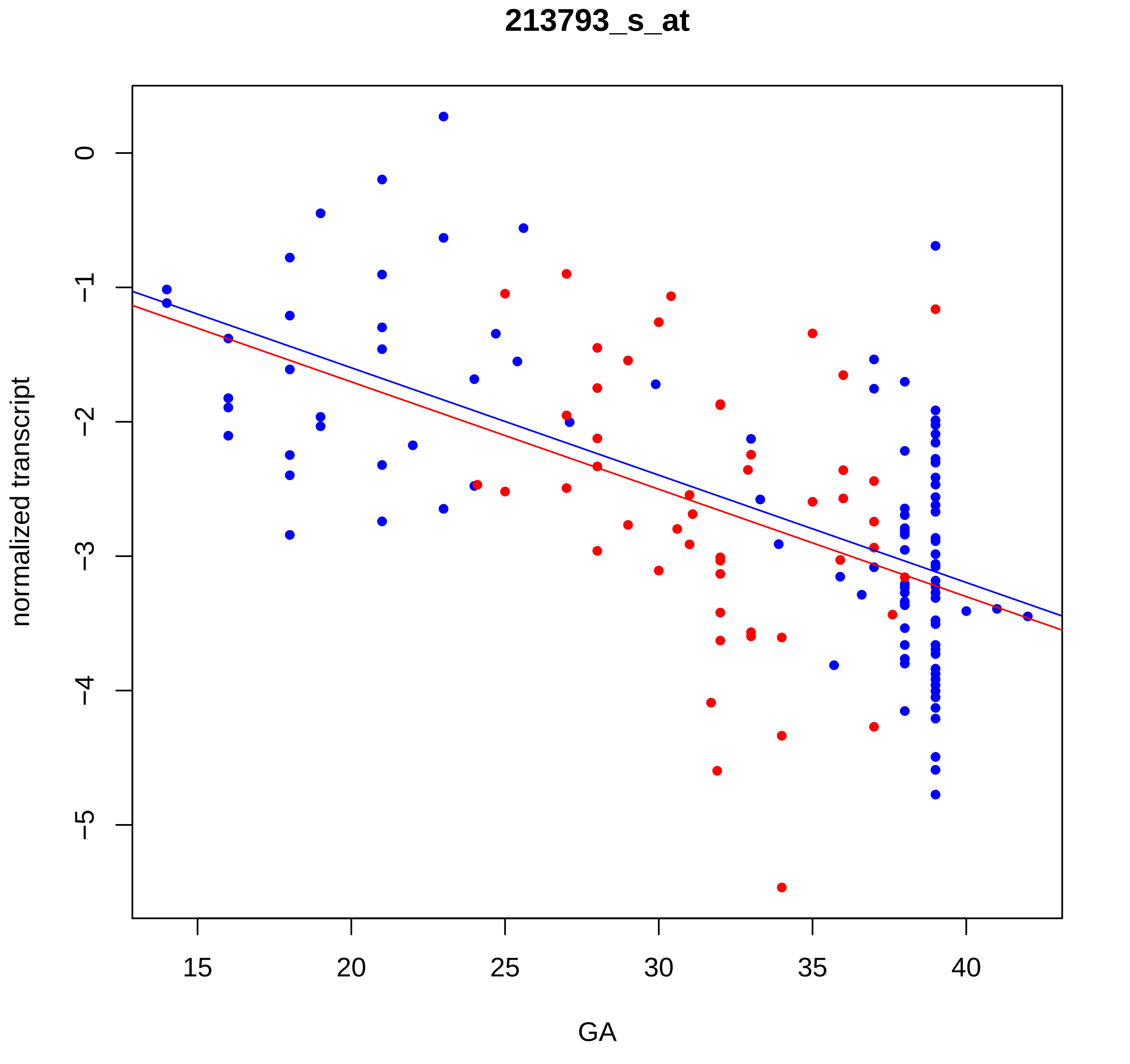

213793_s_at
normalized transcript
GA
0
−1
−2
−3
−4
−5
15
20
25
30
35
40

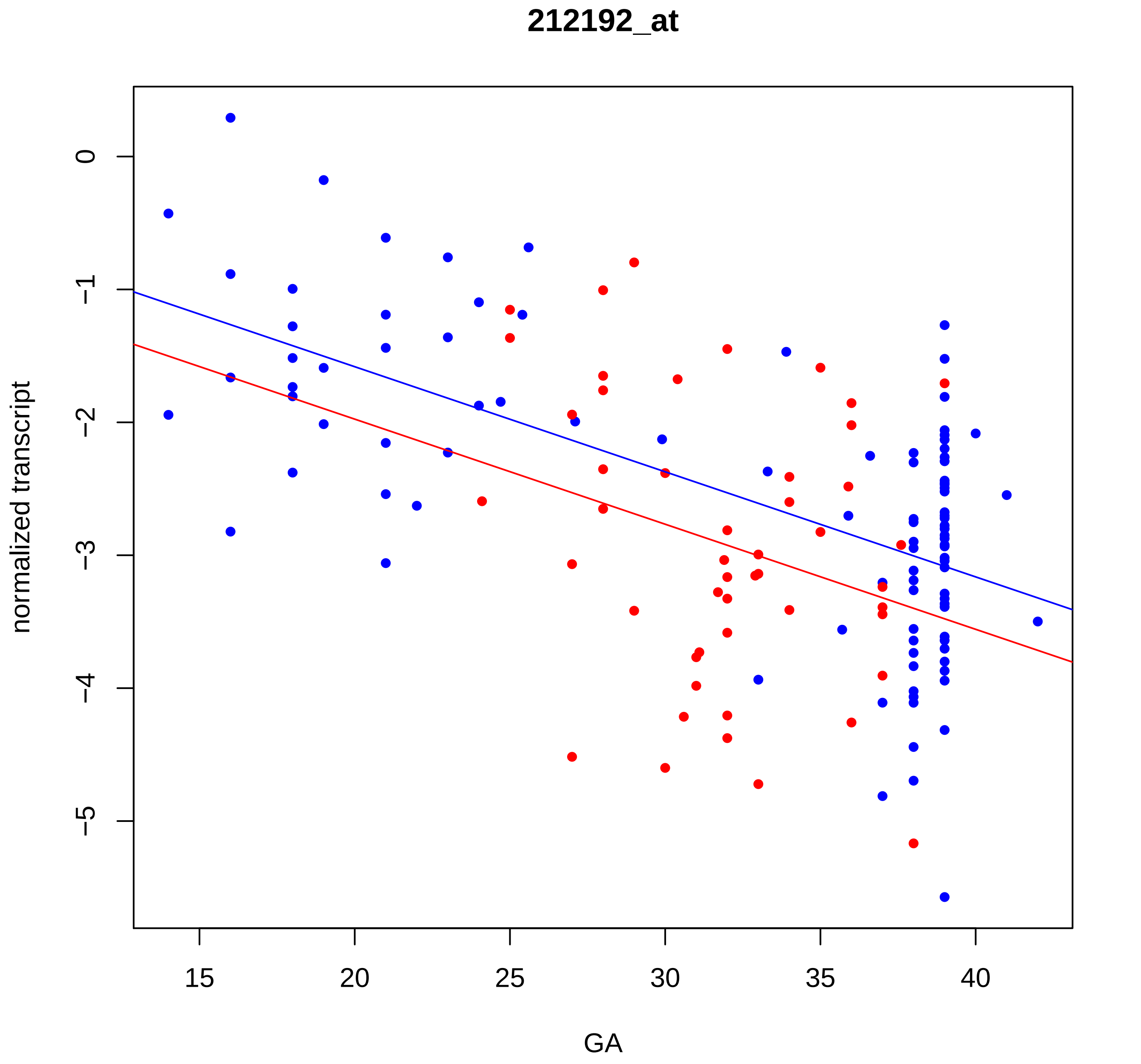

212192_at
normalized transcript
0
−1
−2
−3
−4
−5
15
20
25
30
35
40
GA

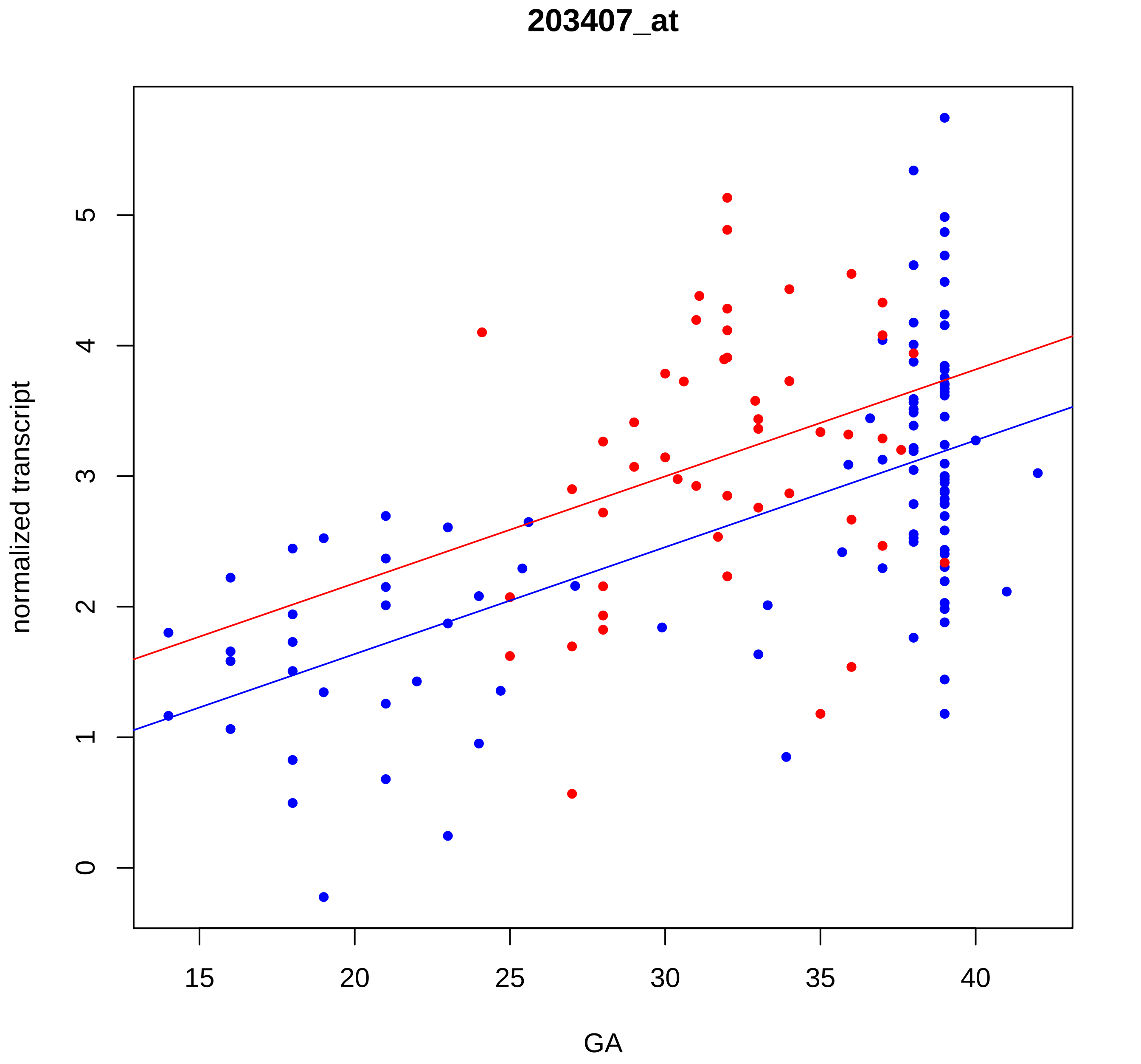
203407_at
normalized transcript
GA
0
1
2
3
4
5
15
20
25
30
35
40

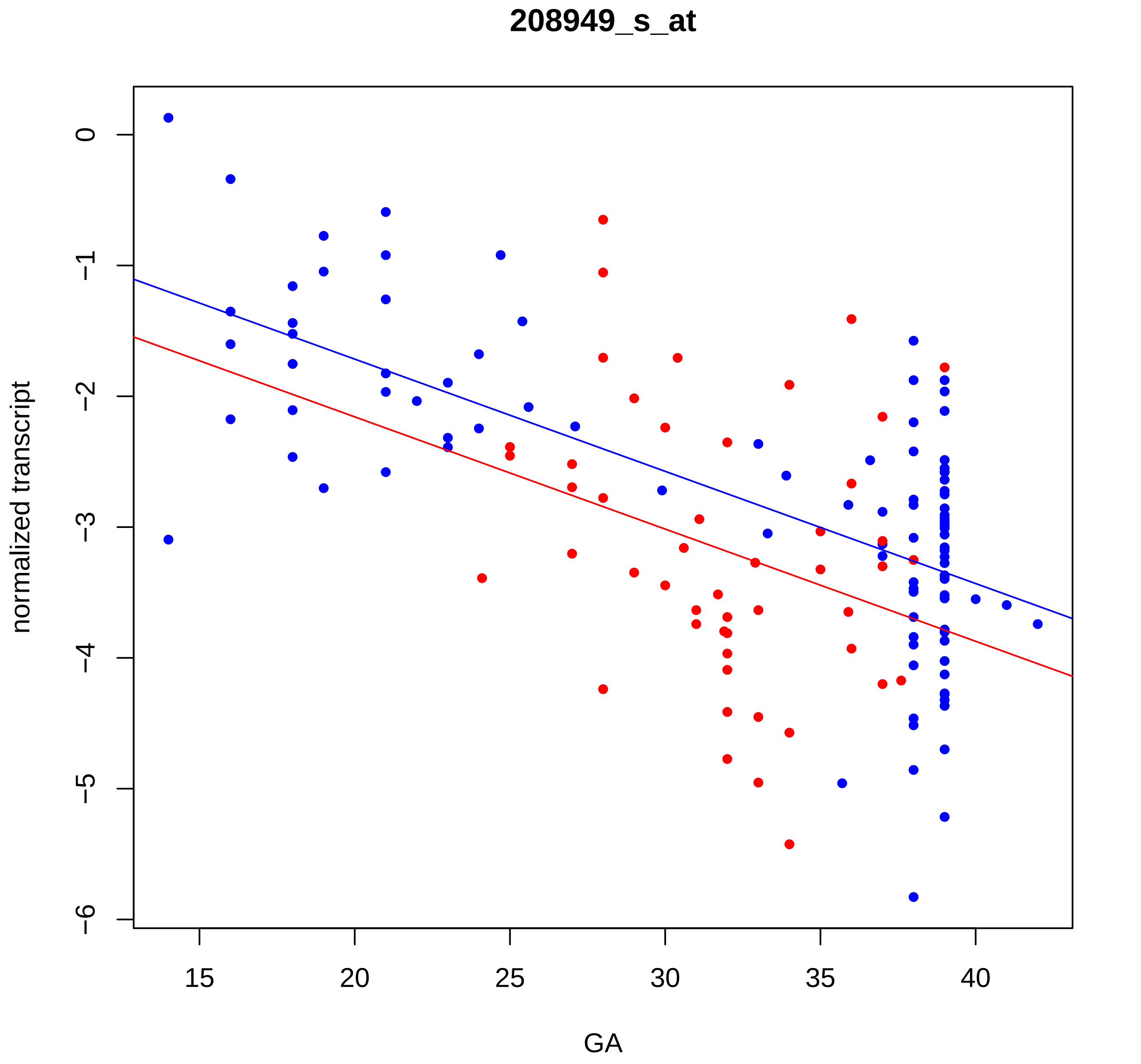

208949_s_at
0
−1
−2
−3
−4
−5
−6
normalized transcript
15
20
25
30
35
40
GA

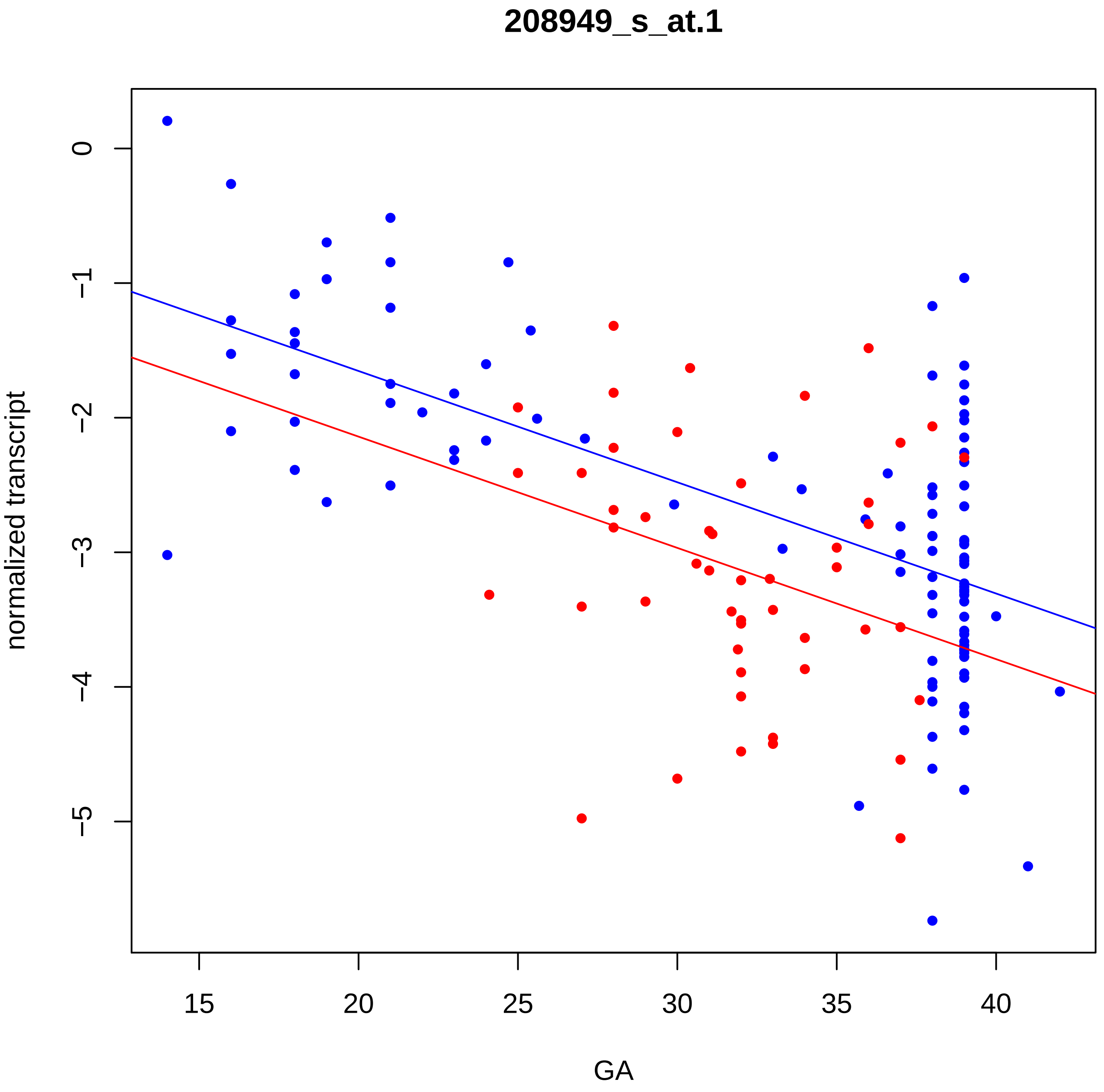
208949_s_at.1
normalized transcript
GA
0
−1
−2
−3
−4
−5
15
20
25
30
35
40

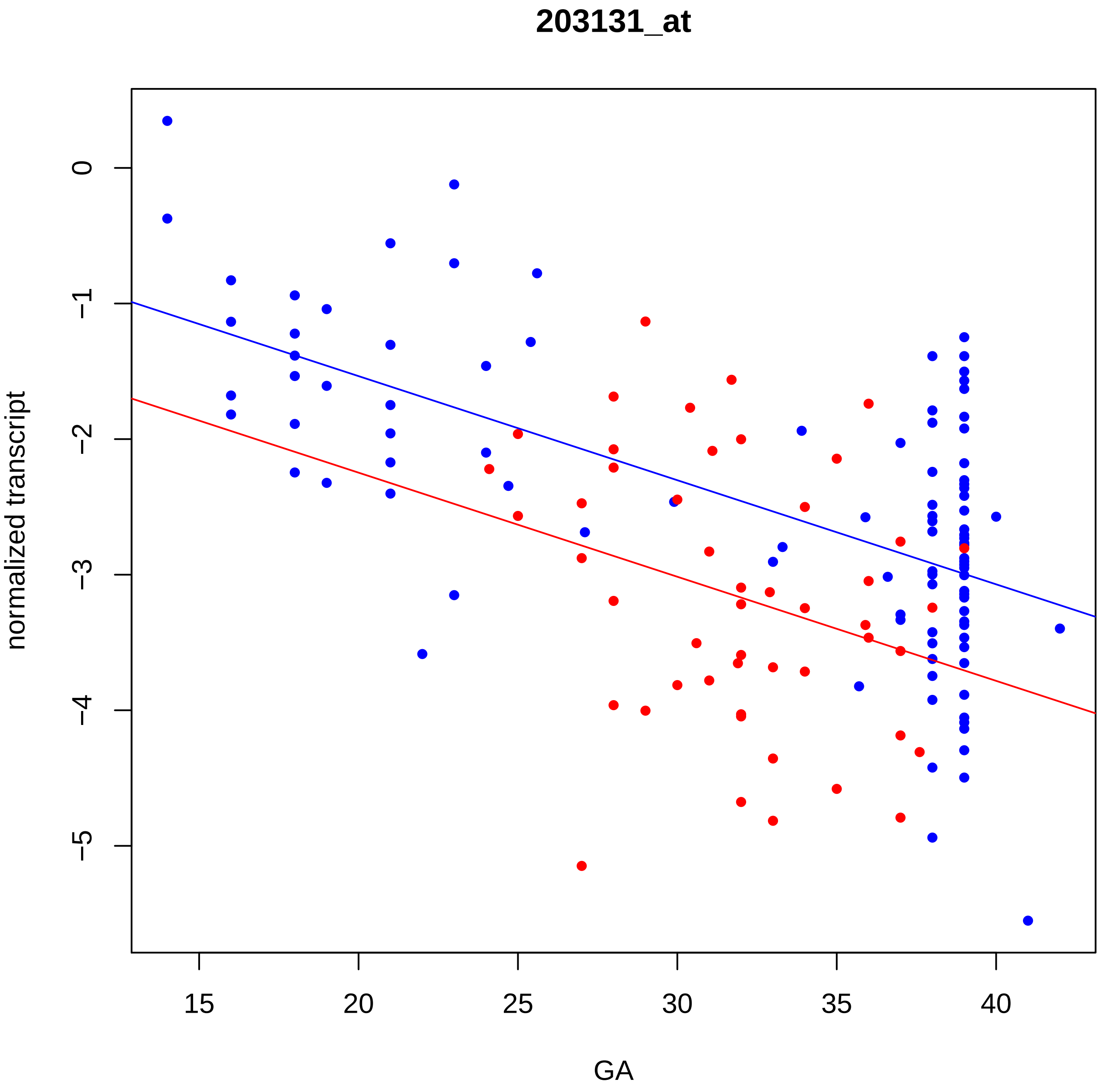

203131_at
normalized transcript
GA
0
−1
−2
−3
−4
−5
15
20
25
30
35
40

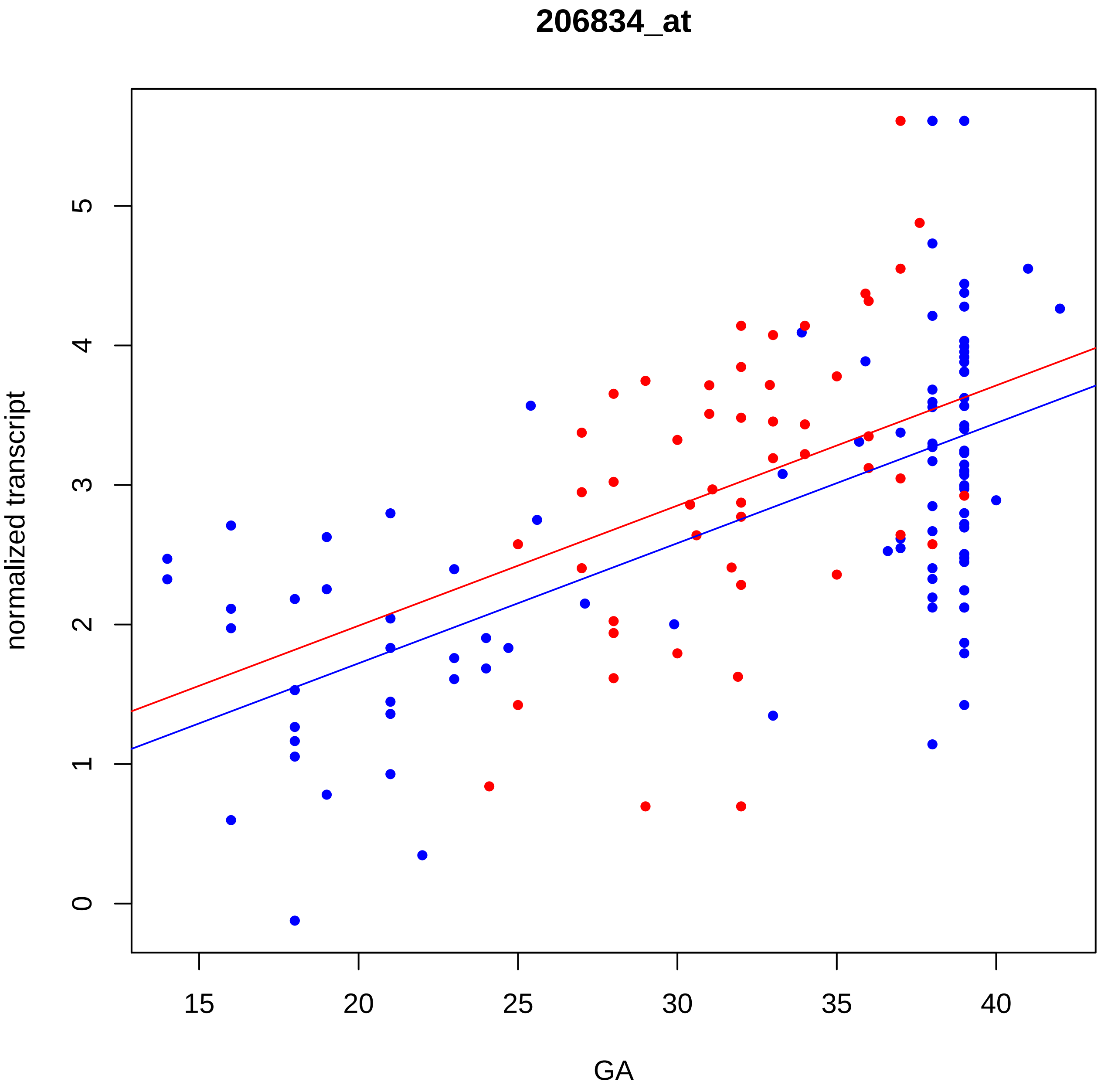

206834_at
normalized transcript
0
1
2
3
4
5
15
20
25
30
35
40
GA

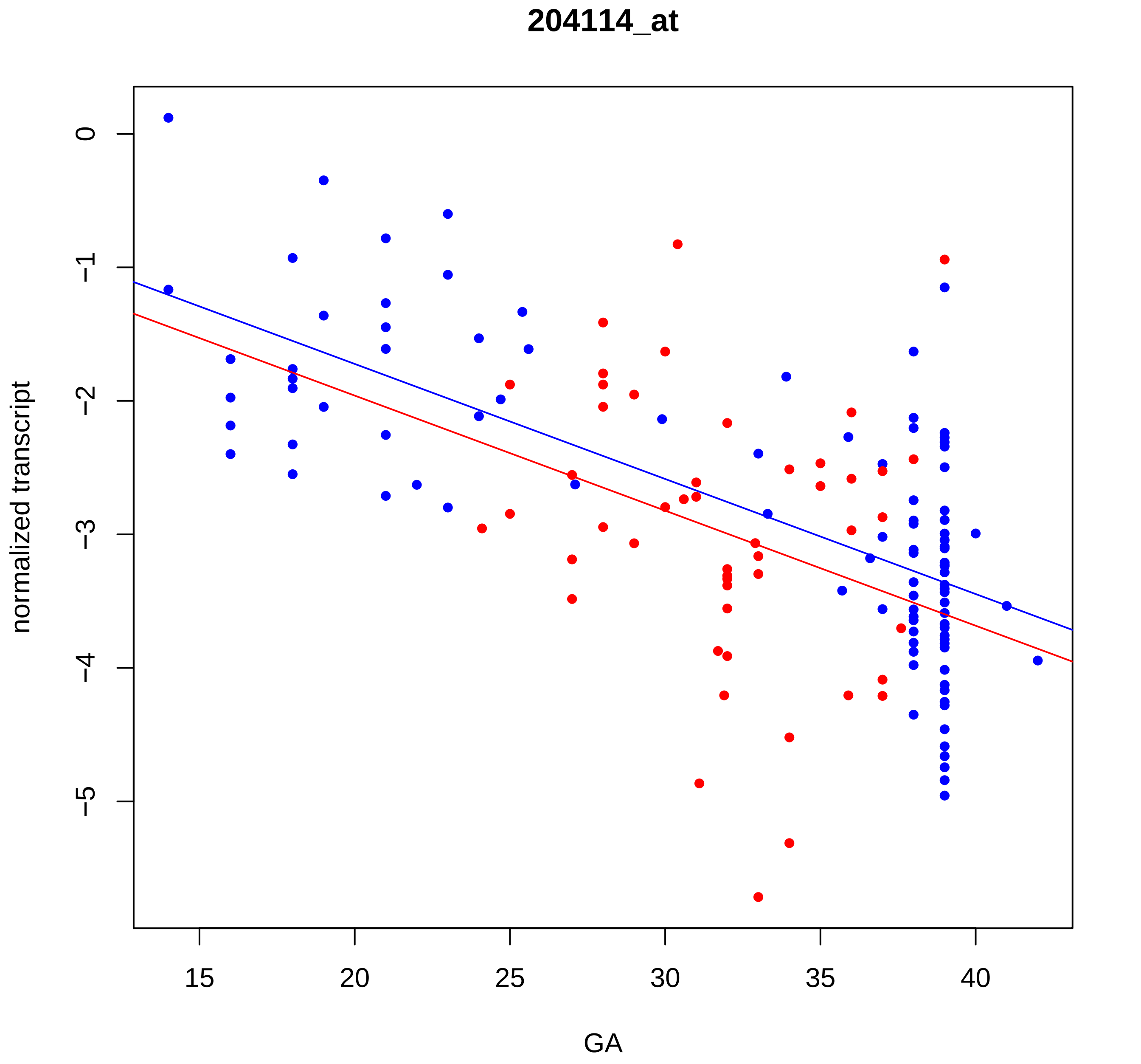
204114_at
normalized transcript
0
−1
−2
−3
−4
−5
15
20
25
30
35
40
GA

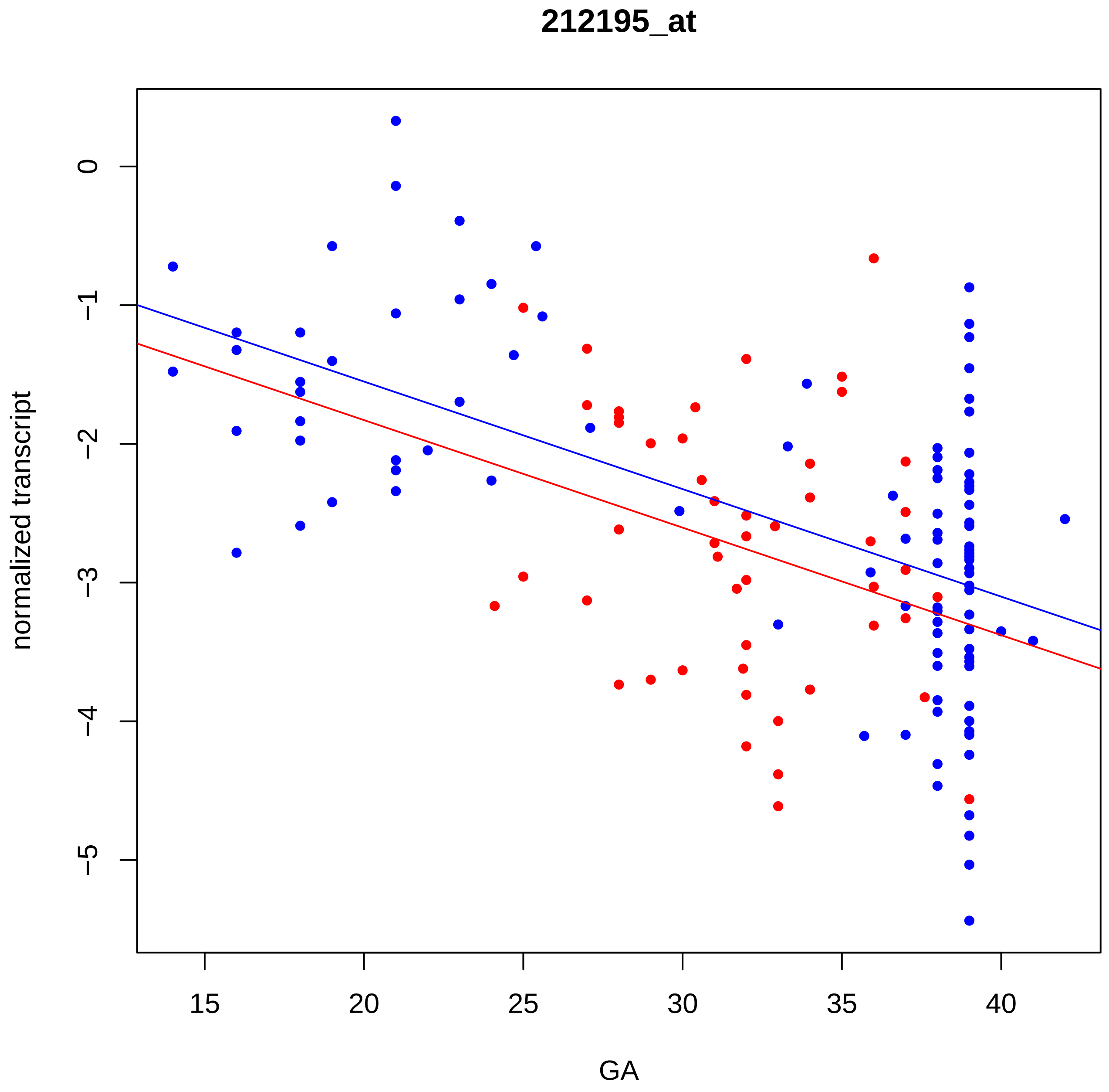
212195_at
normalized transcript
GA
0
−1
−2
−3
−4
−5
15
20
25
30
35
40

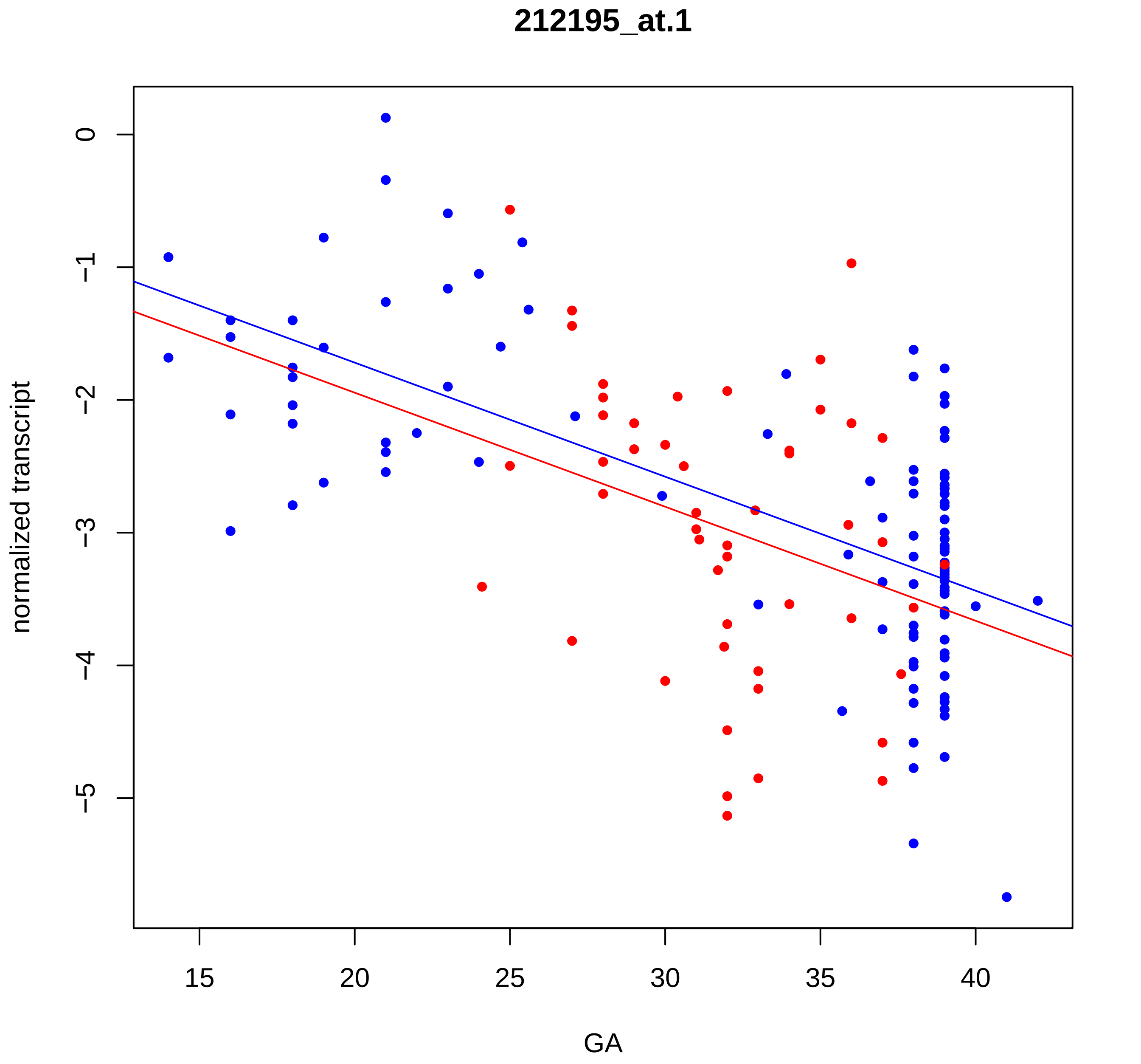
212195_at.1
normalized transcript
GA
0
−1
−2
−3
−4
−5
15
20
25
30
35
40

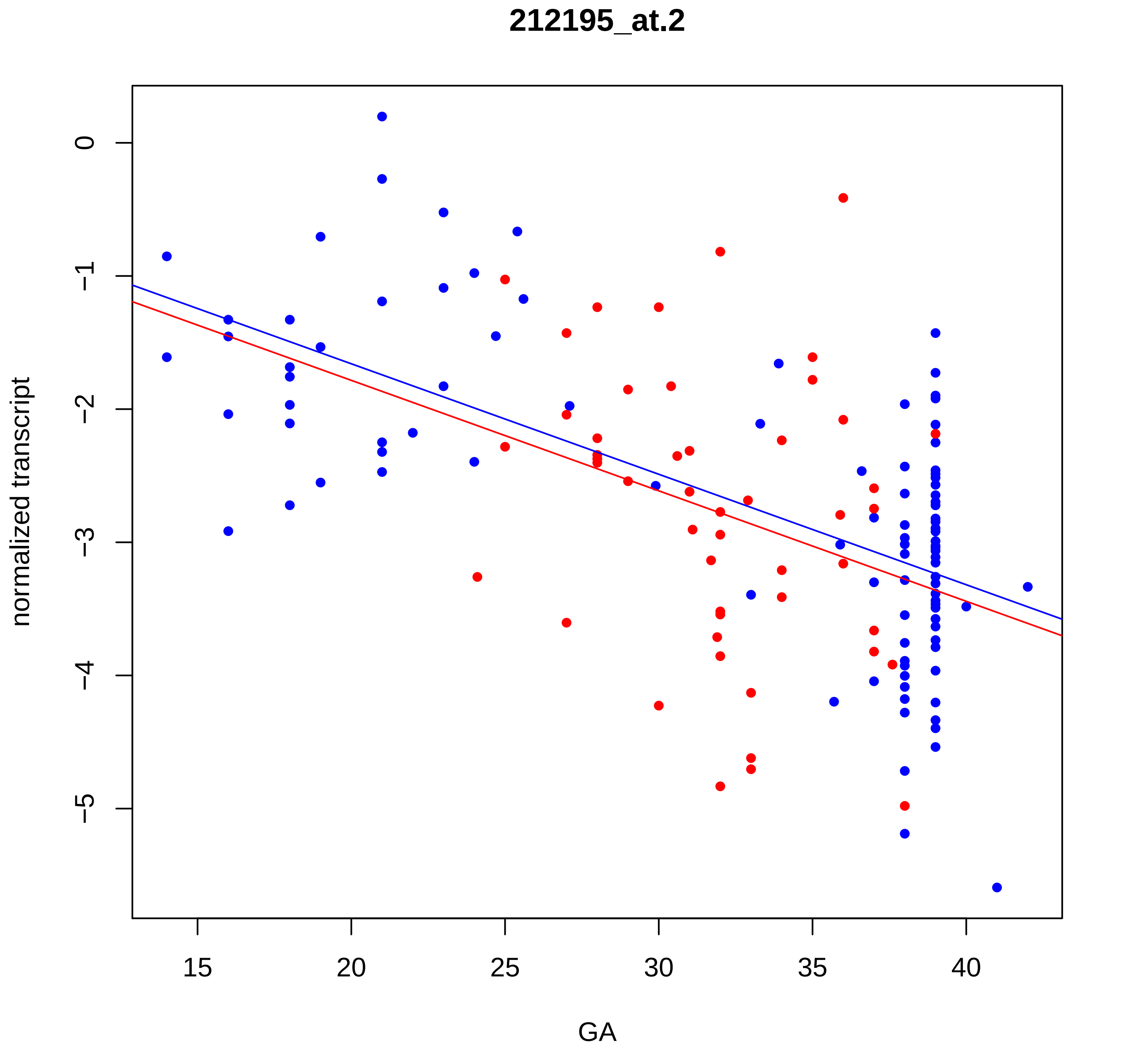

212195_at.2
normalized transcript
GA
0
−1
−2
−3
−4
−5
15
20
25
30
35
40

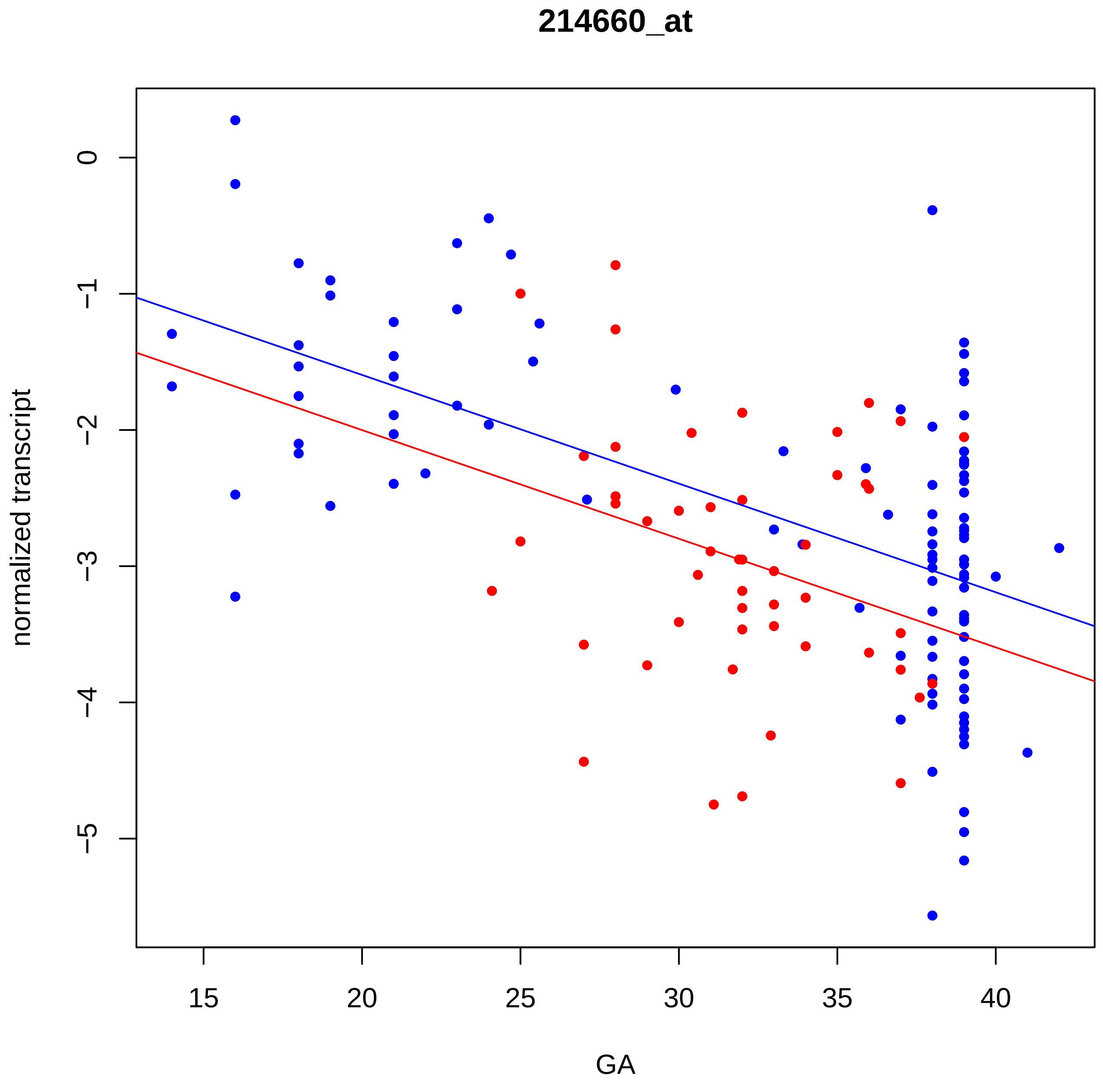

214660_at
normalized transcript
GA
0
−1
−2
−3
−4
−5
15
20
25
30
35
40

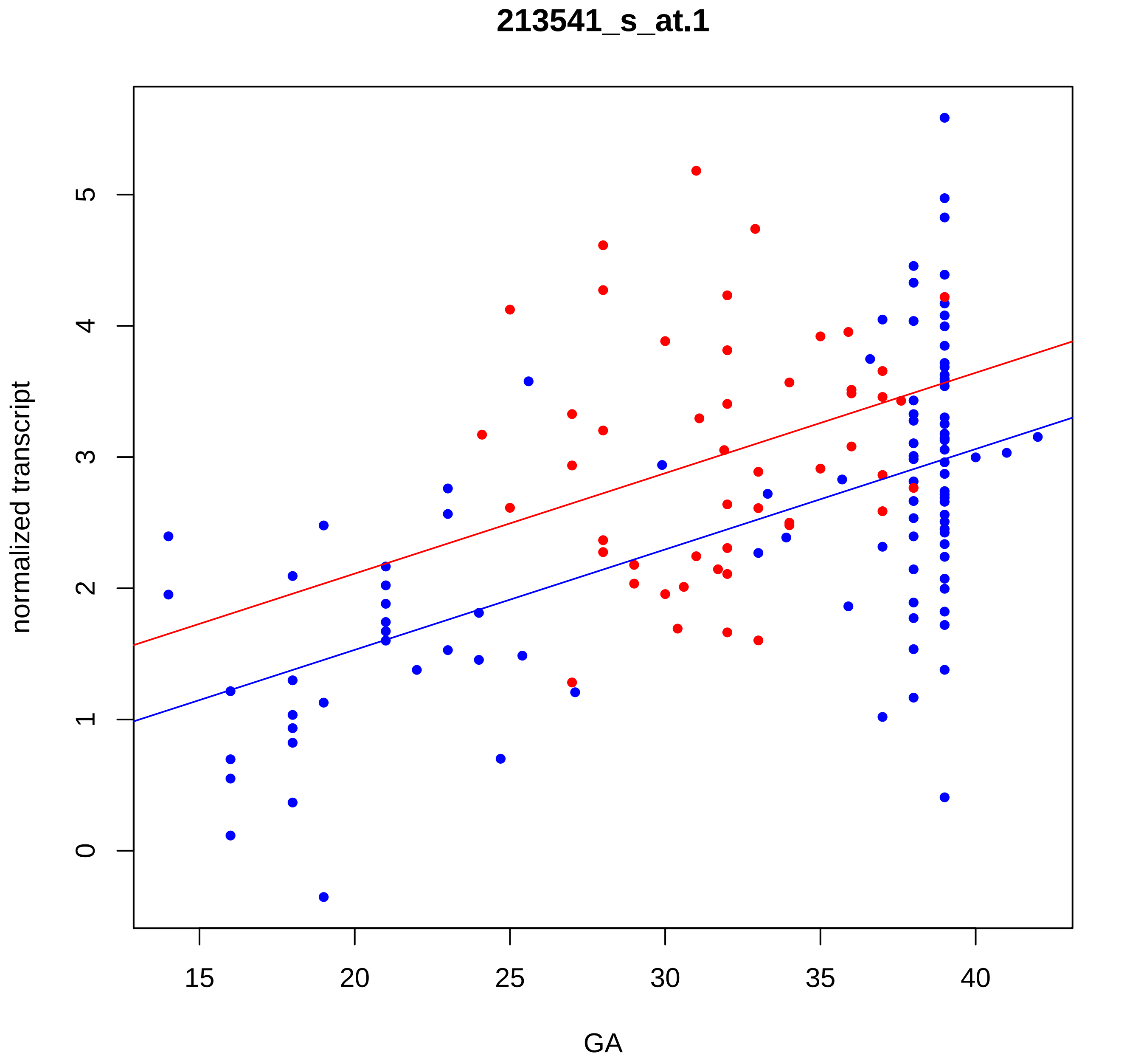

213541_s_at.1
normalized transcript
0
1
2
3
4
5
15
20
25
30
35
40
GA

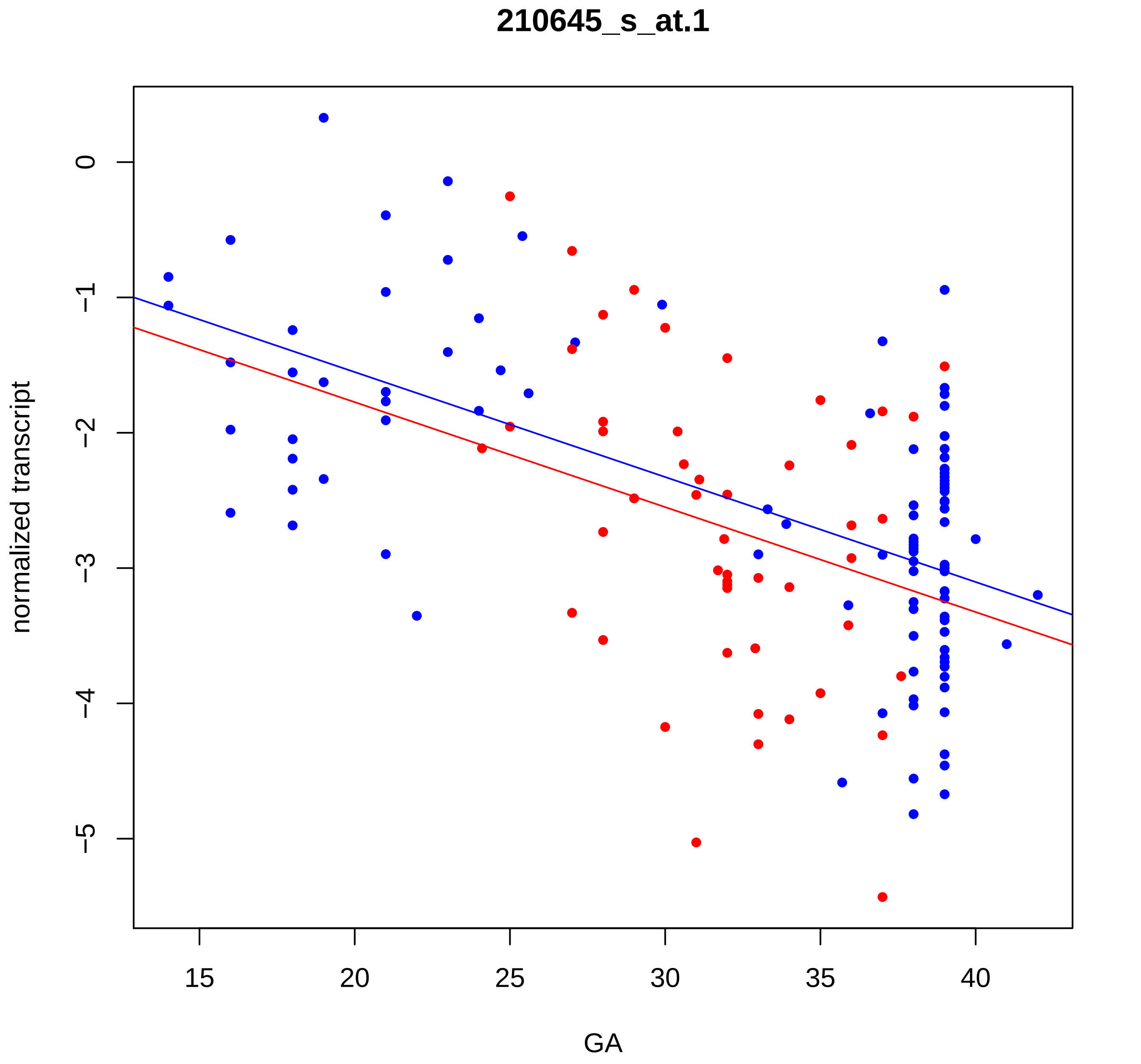

210645_s_at.1
normalized transcript
0
−1
−2
−3
−4
−5
15
20
25
30
35
40
GA

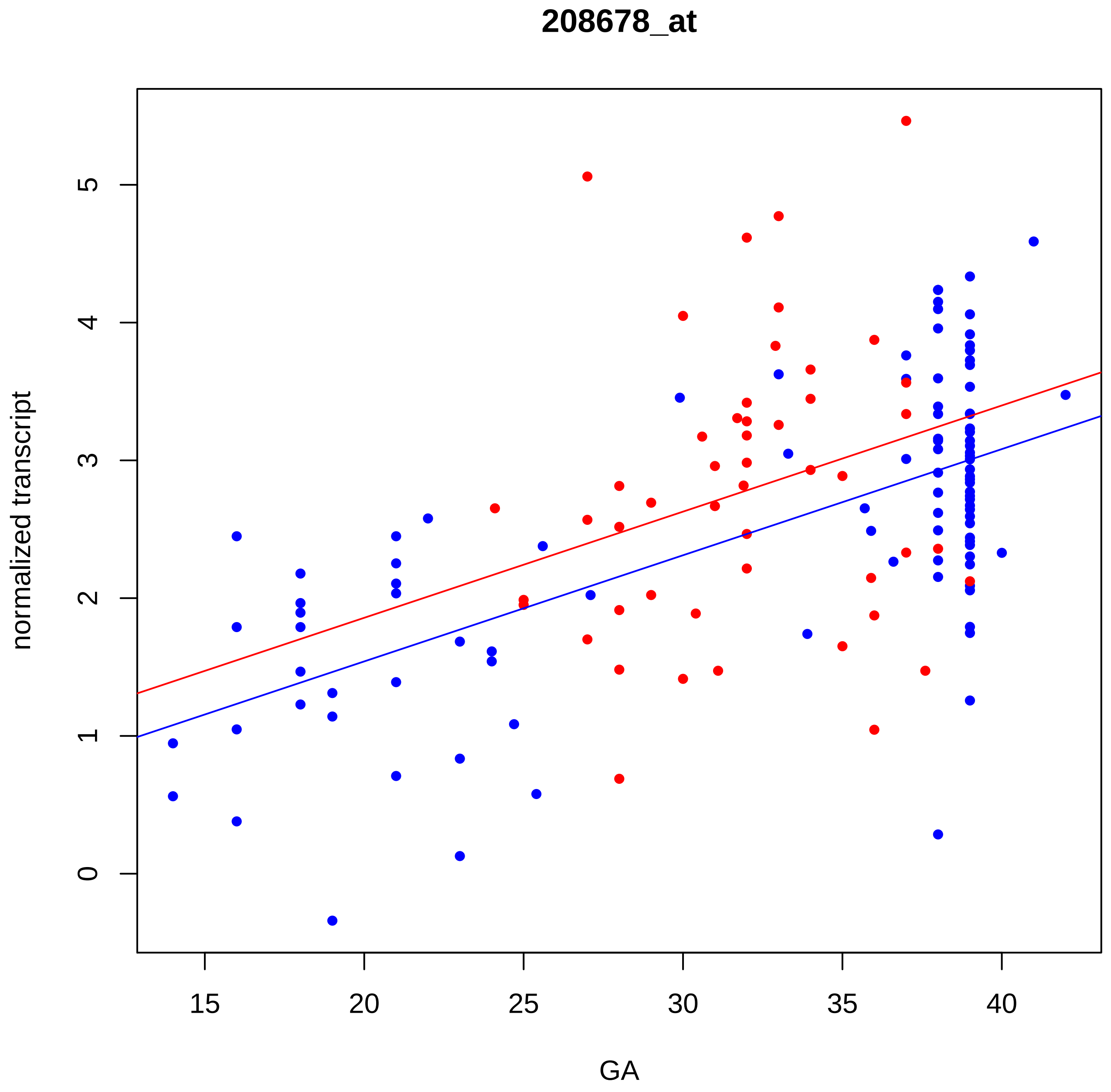

208678_at
normalized transcript
GA
0
1
2
3
4
5
15
20
25
30
35
40

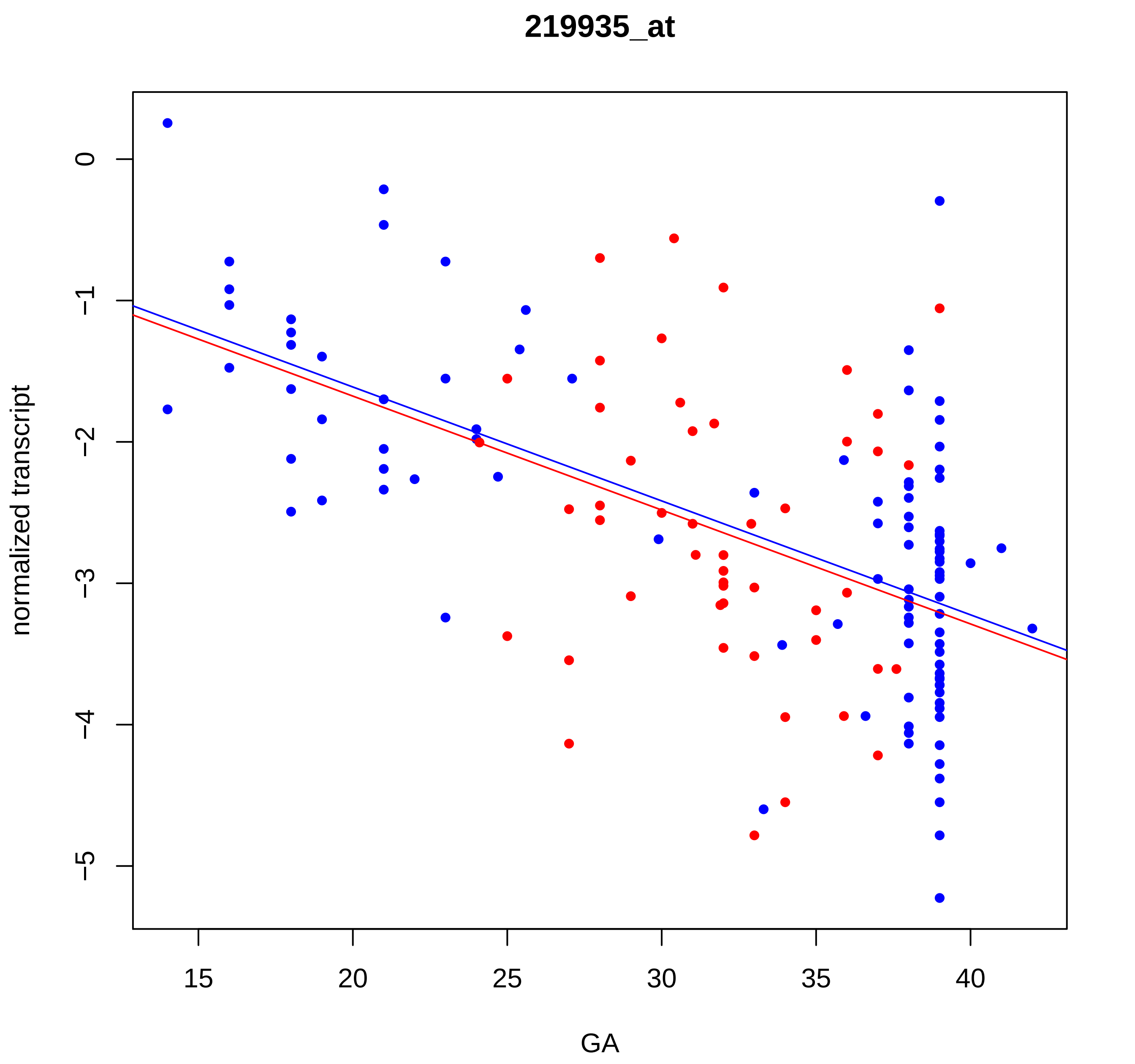

219935_at
normalized transcript
0
−1
−2
−3
−4
−5
15
20
25
30
35
40
GA

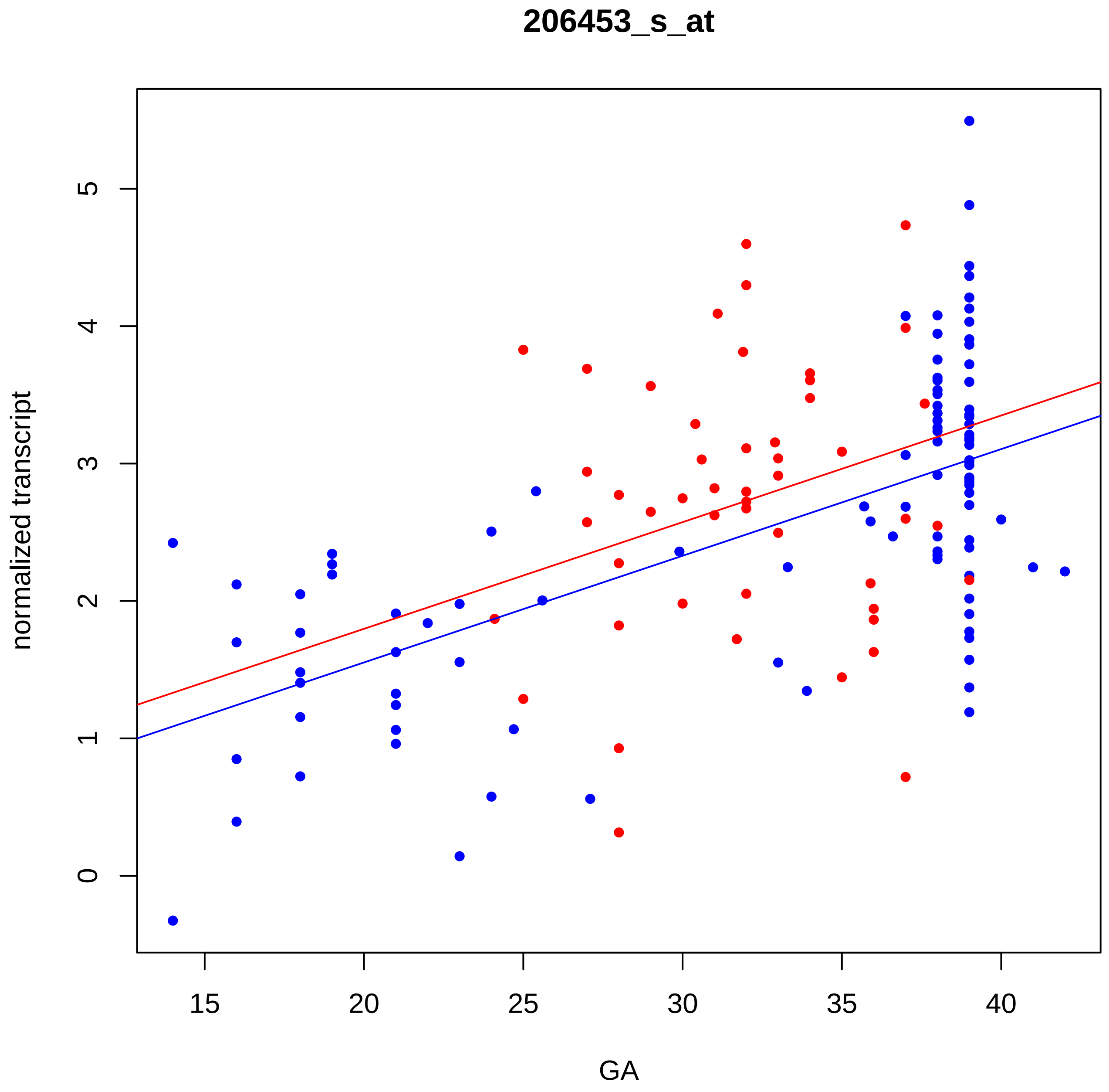
206453_s_at
normalized transcript
GA
0
1
2
3
4
5
15
20
25
30
35
40

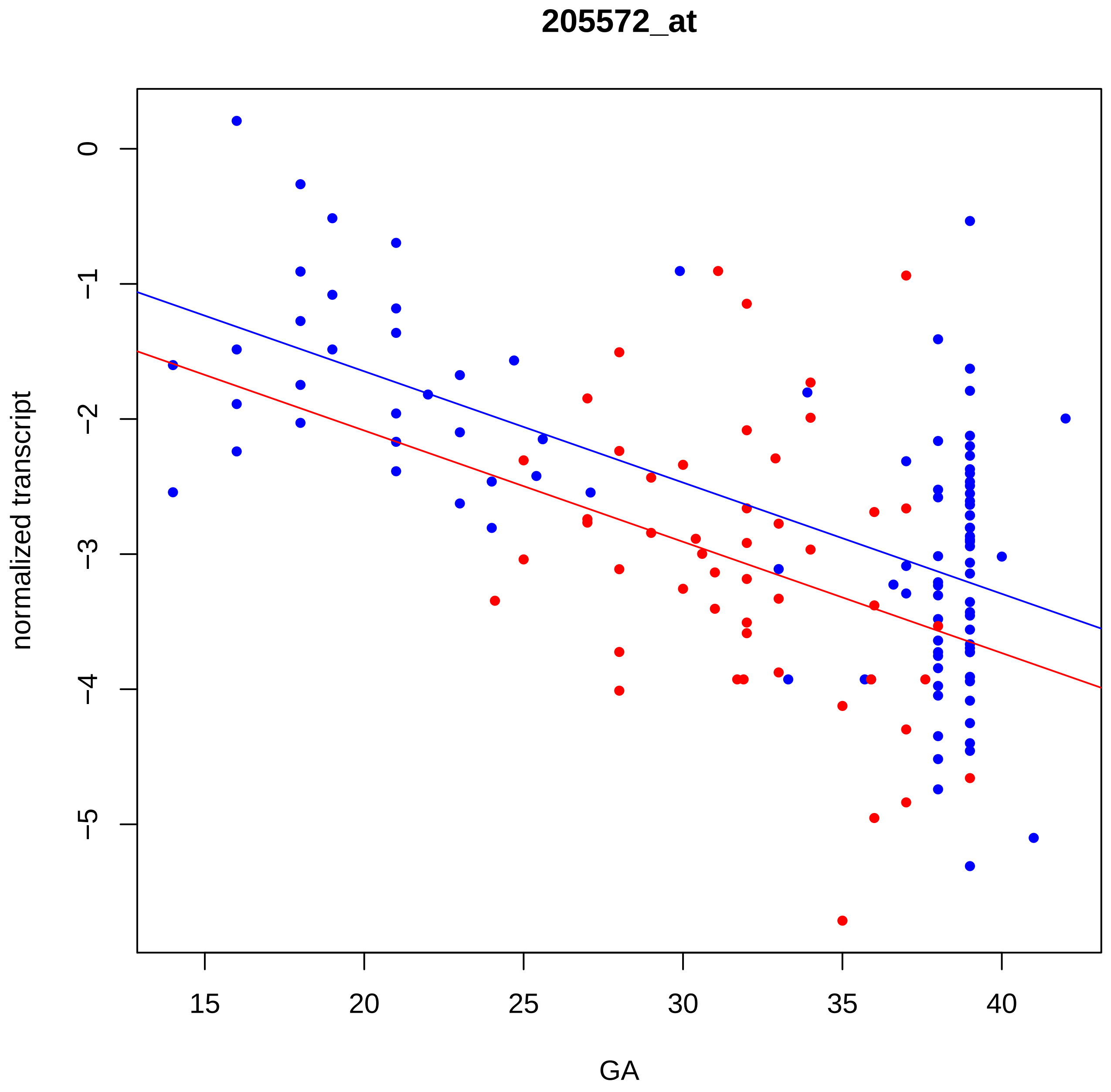

205572_at
0
−1
−2
−3
−4
−5
normalized transcript
15
20
25
30
35
40
GA

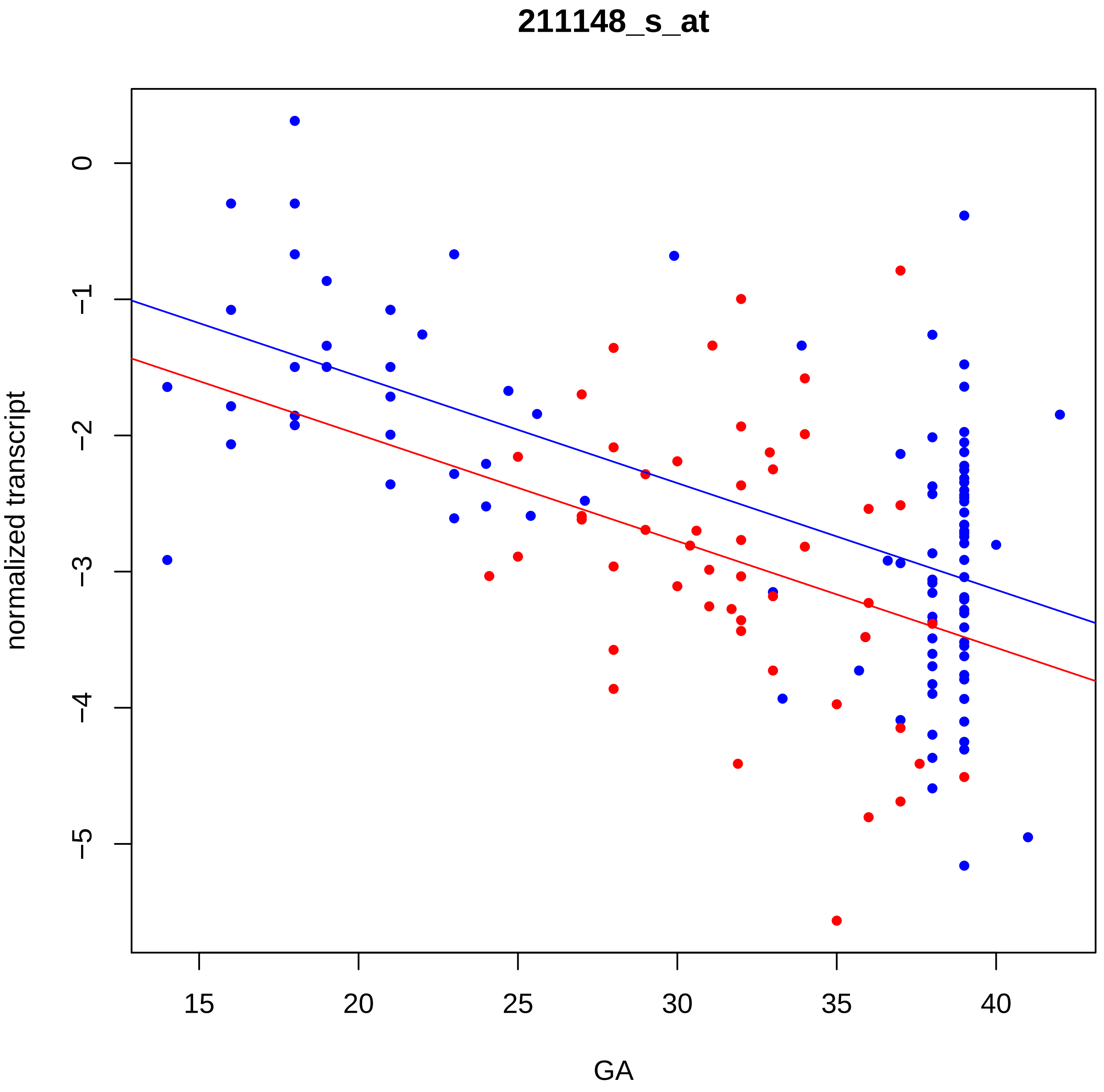

211148_s_at
normalized transcript
0
−1
−2
−3
−4
−5
15
20
25
30
35
40
GA

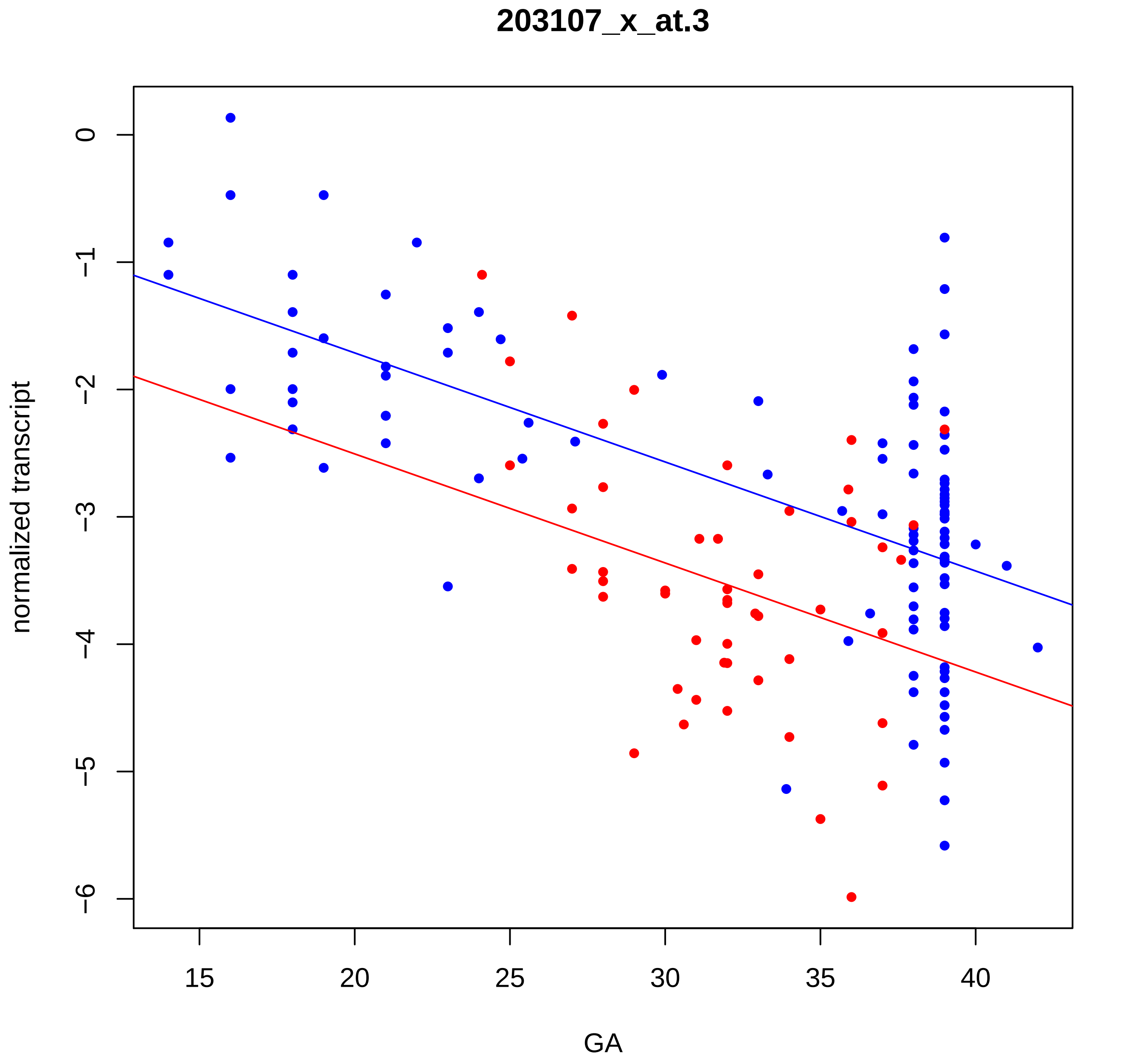

203107_x_at.3
0
−1
−2
−3
−4
−5
−6
normalized transcript
15
20
25
30
35
40
GA

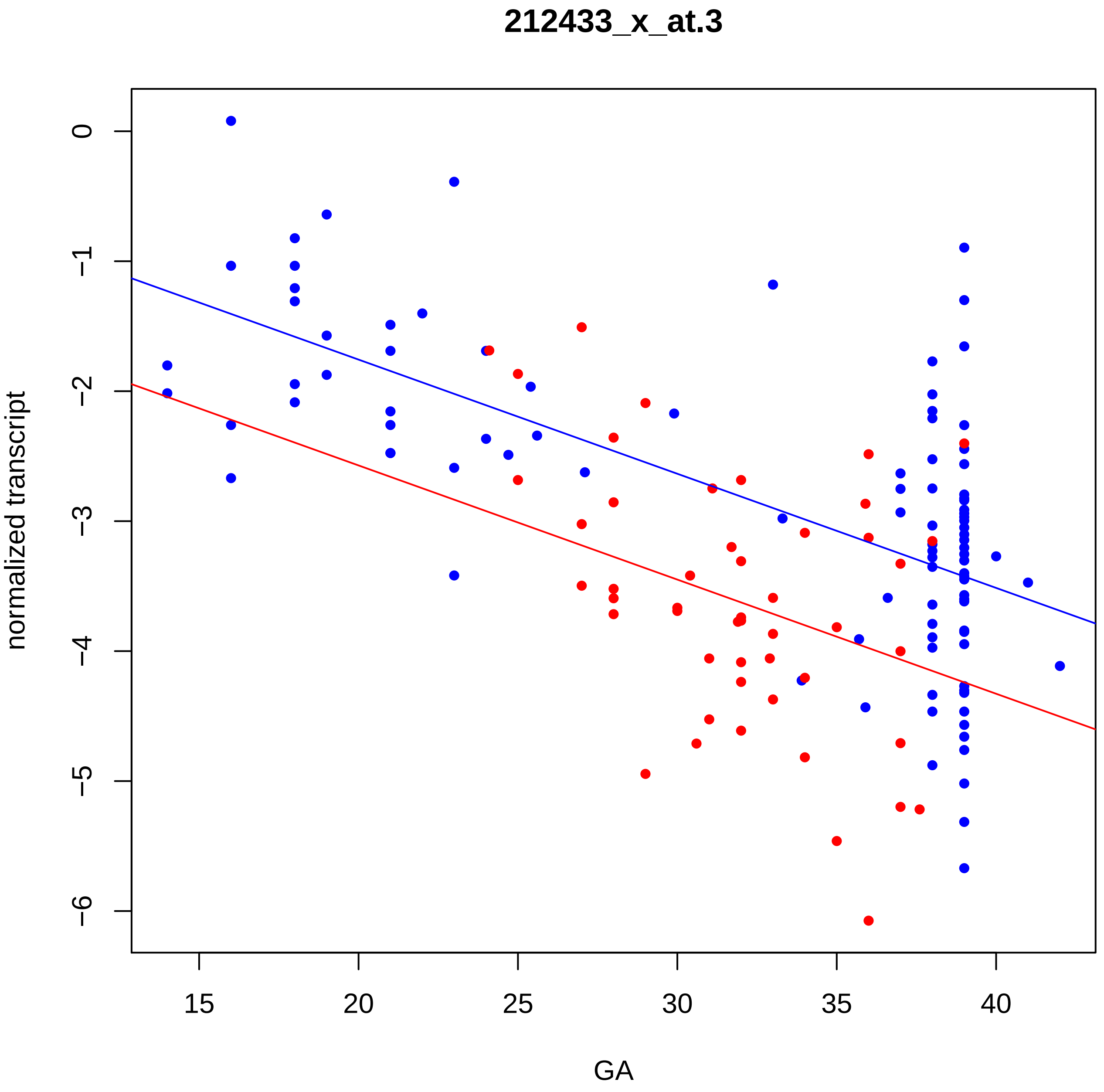
212433_x_at.3
normalized transcript
GA
0
−1
−2
−3
−4
−5
−6
15
20
25
30
35
40

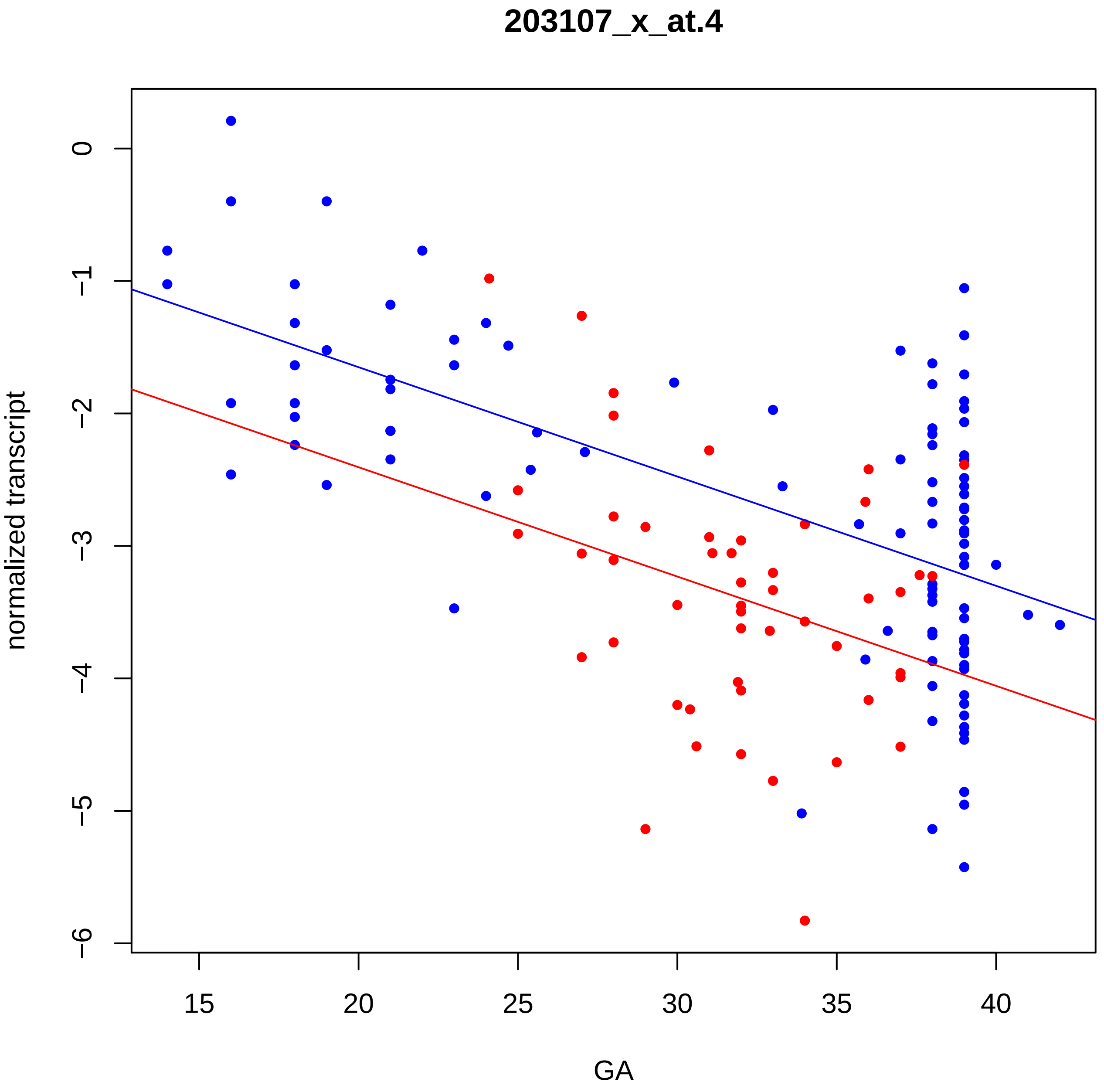

203107_x_at.4
normalized transcript
0
−1
−2
−3
−4
−5
−6
15
20
25
30
35
40
GA

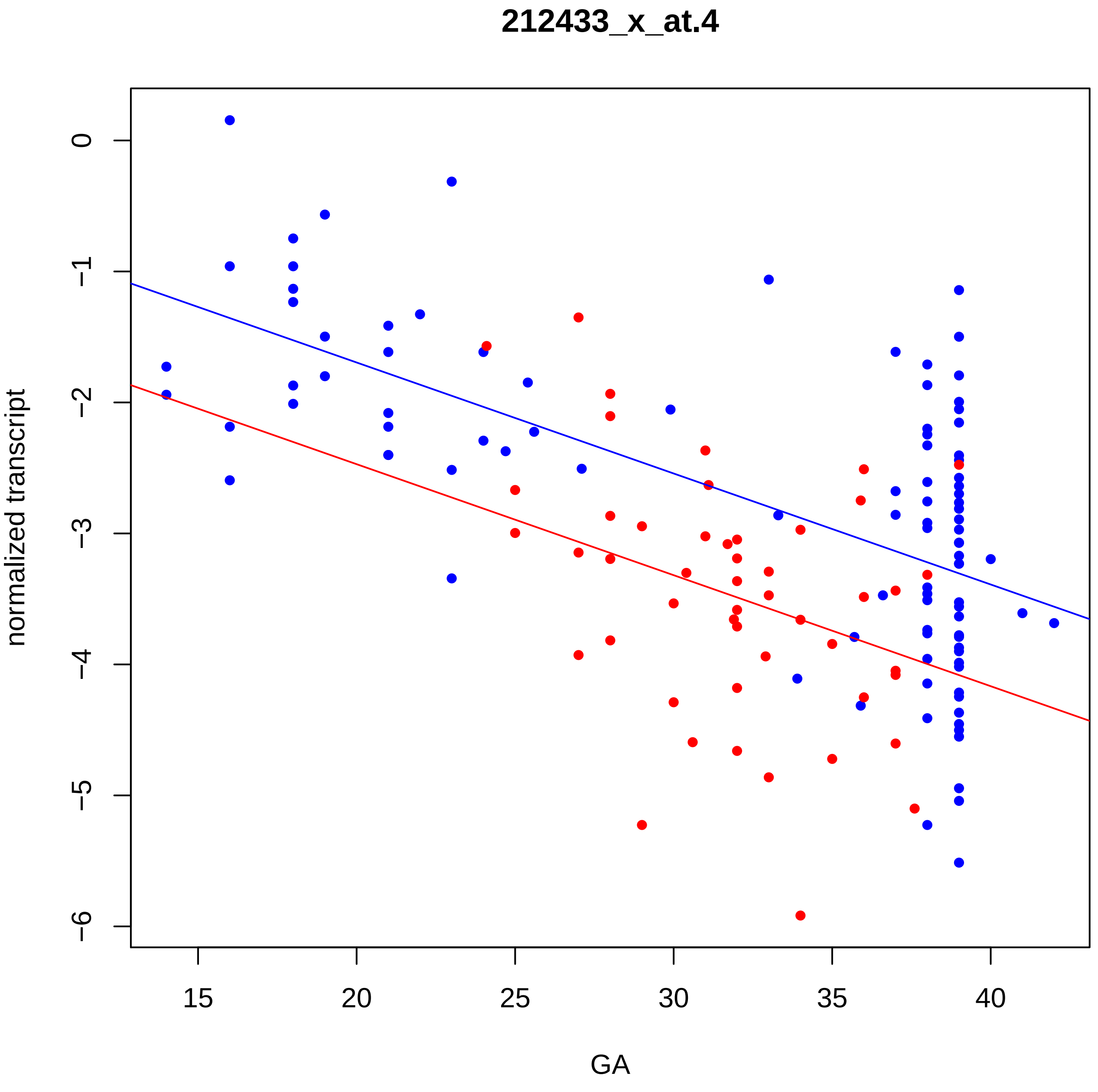

212433_x_at.4
normalized transcript
GA
0
−1
−2
−3
−4
−5
−6
15
20
25
30
35
40

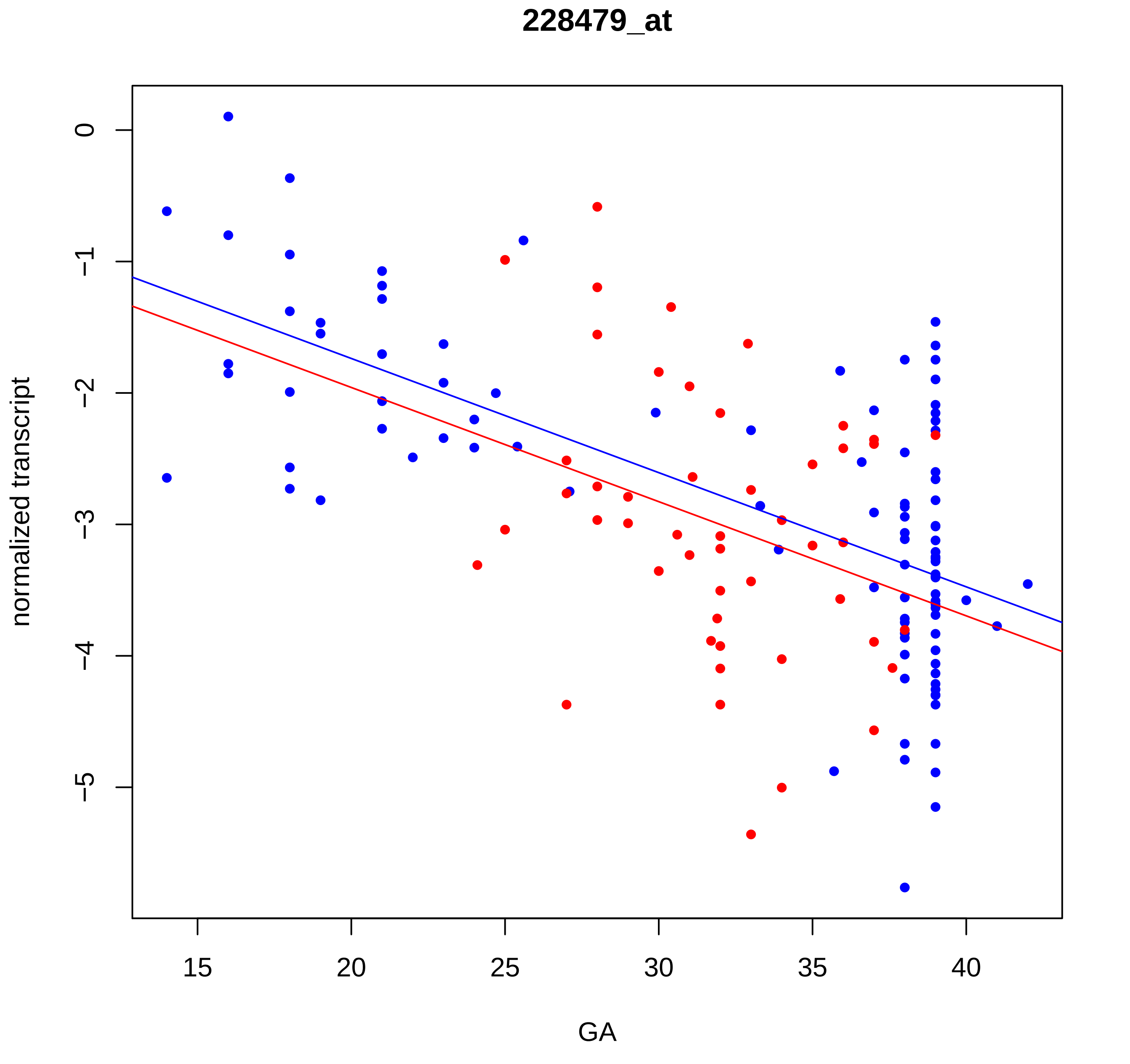
228479_at
normalized transcript
GA
0
−1
−2
−3
−4
−5
15
20
25
30
35
40

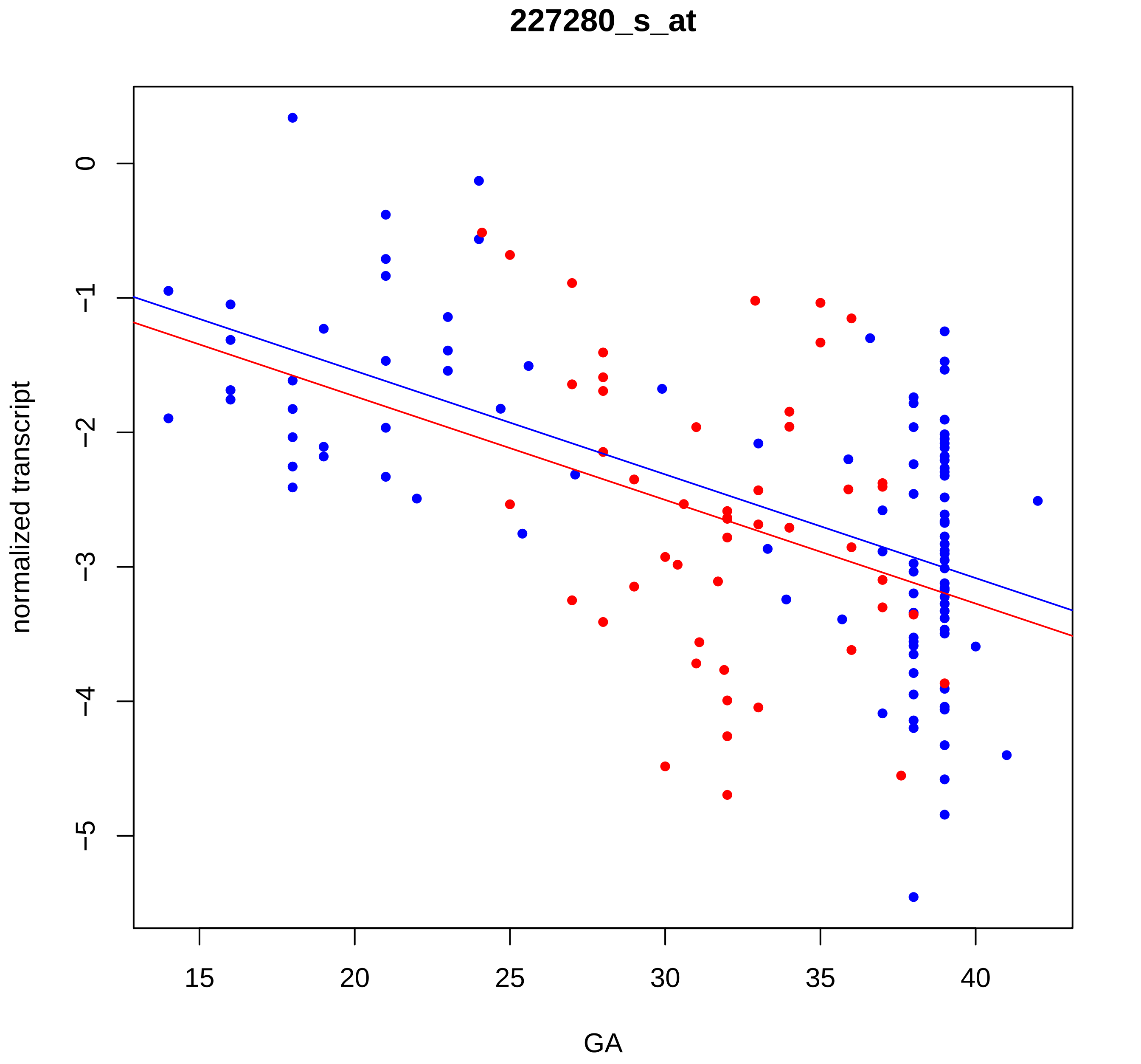

227280_s_at
0
−1
−2
−3
−4
−5
normalized transcript
15
20
25
30
35
40
GA

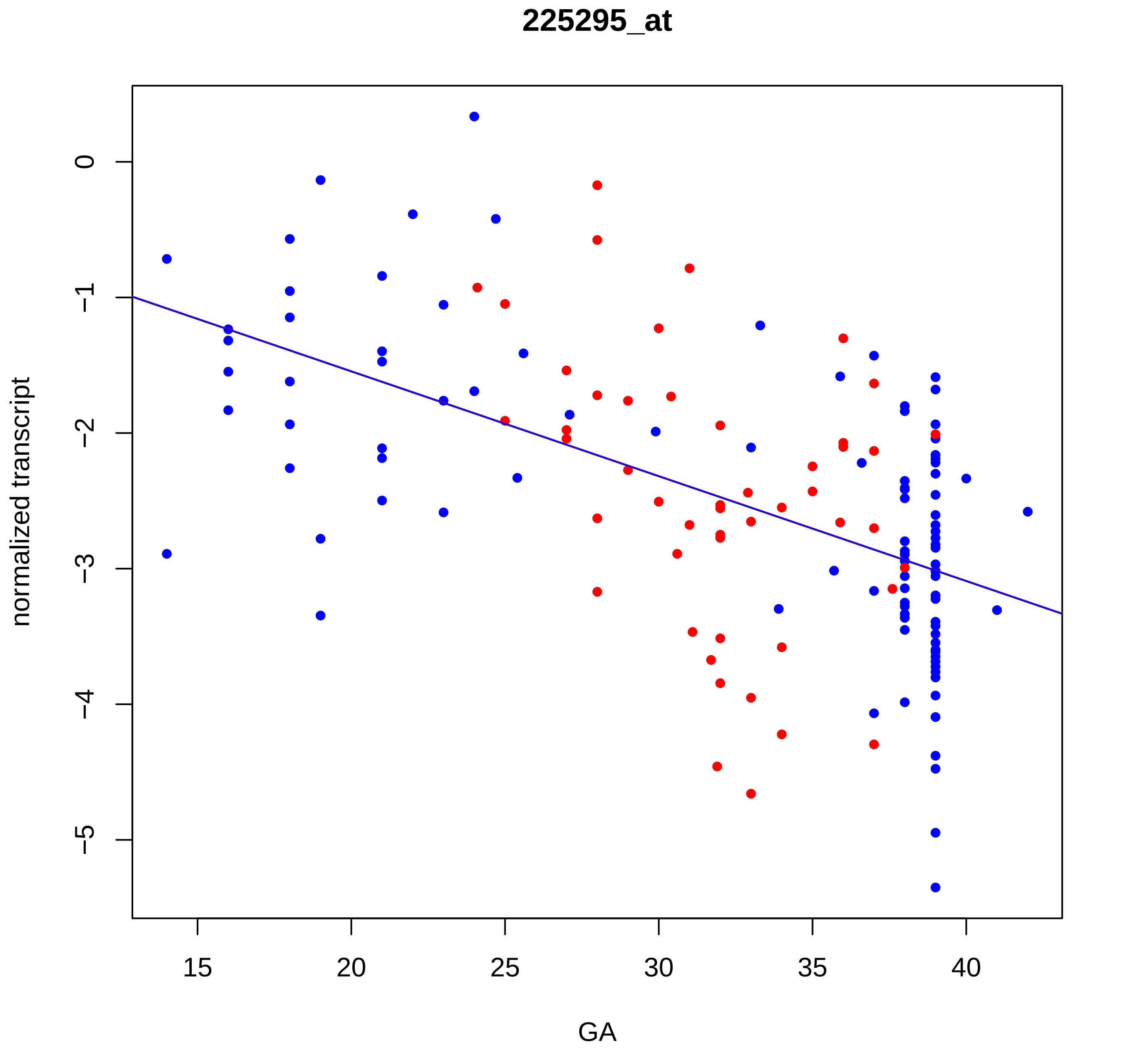
225295_at
normalized transcript
GA
0
−1
−2
−3
−4
−5
15
20
25
30
35
40

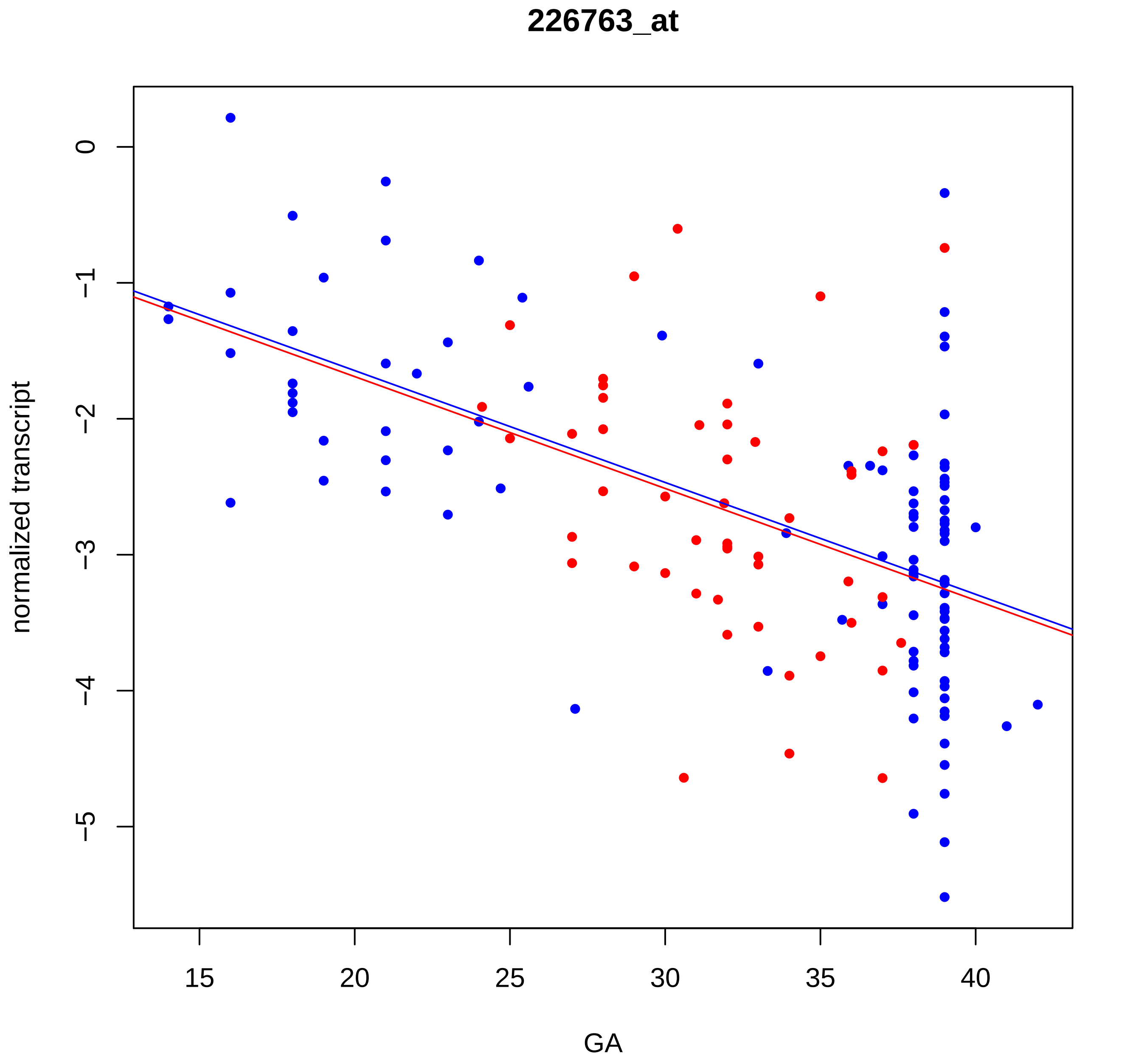

226763_at
normalized transcript
0
−1
−2
−3
−4
−5
15
20
25
30
35
40
GA

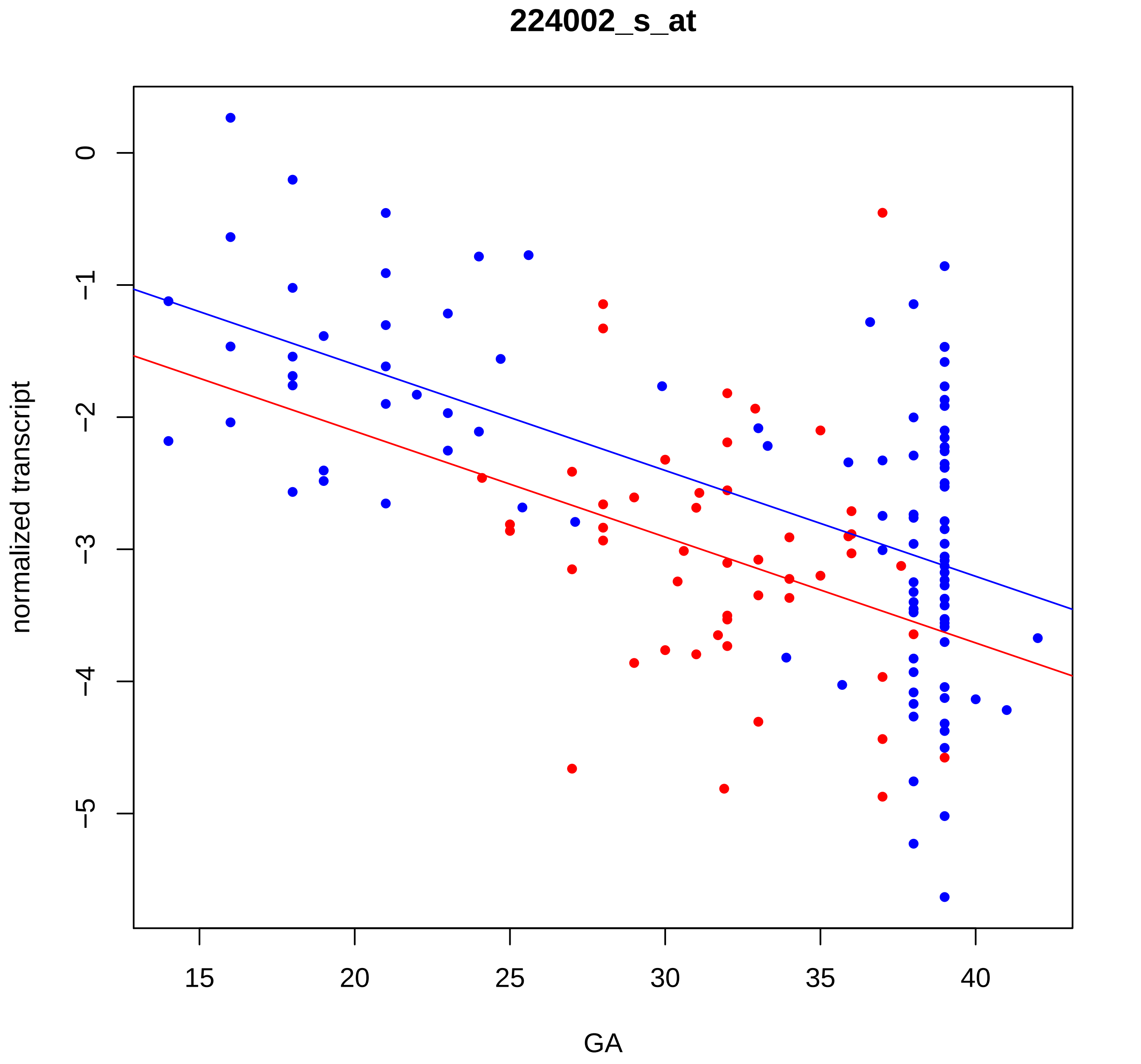

224002_s_at
normalized transcript
GA
0
−1
−2
−3
−4
−5
15
20
25
30
35
40

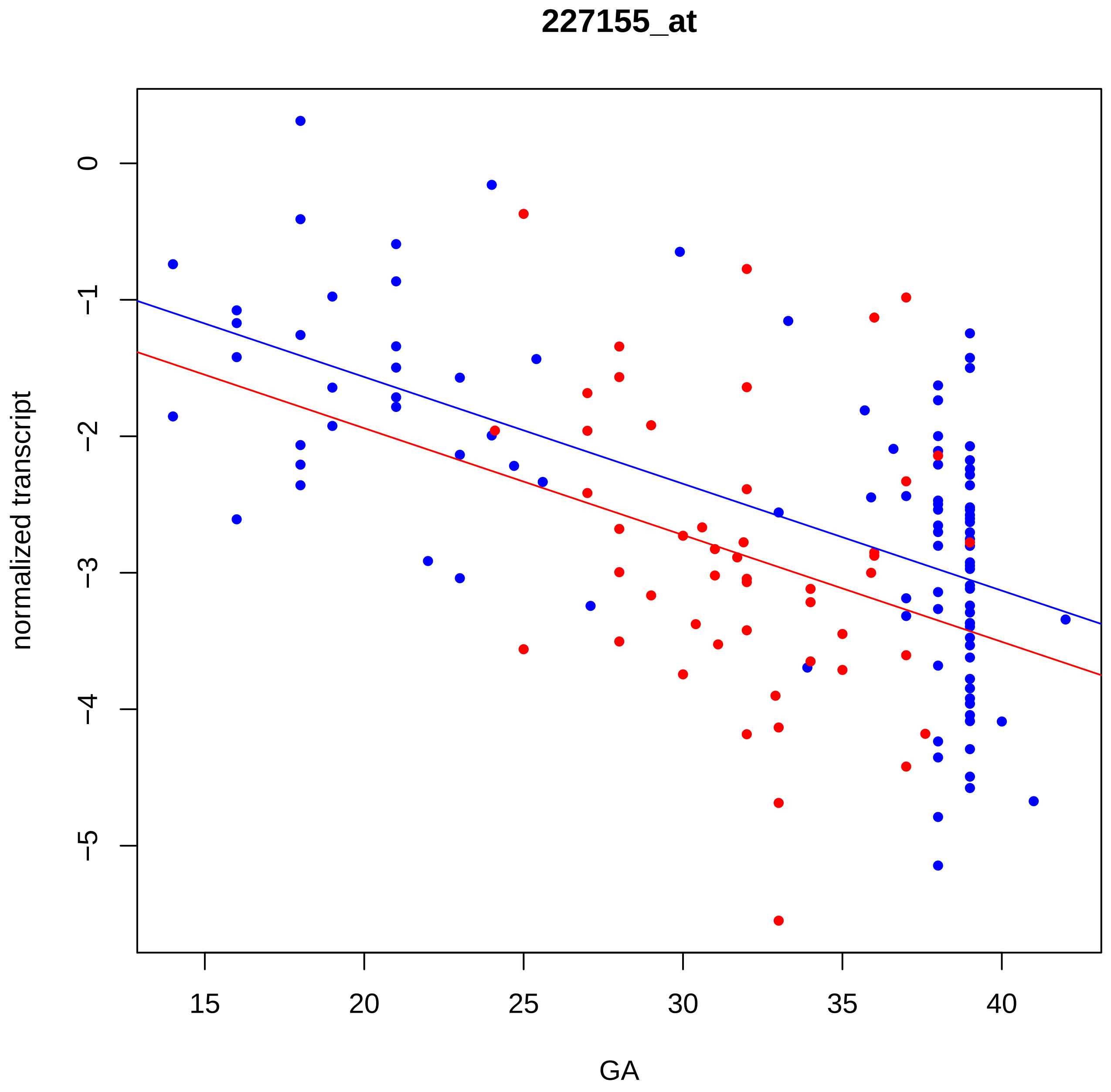
227155_at
normalized transcript
GA
0
−1
−2
−3
−4
−5
15
20
25
30
35
40

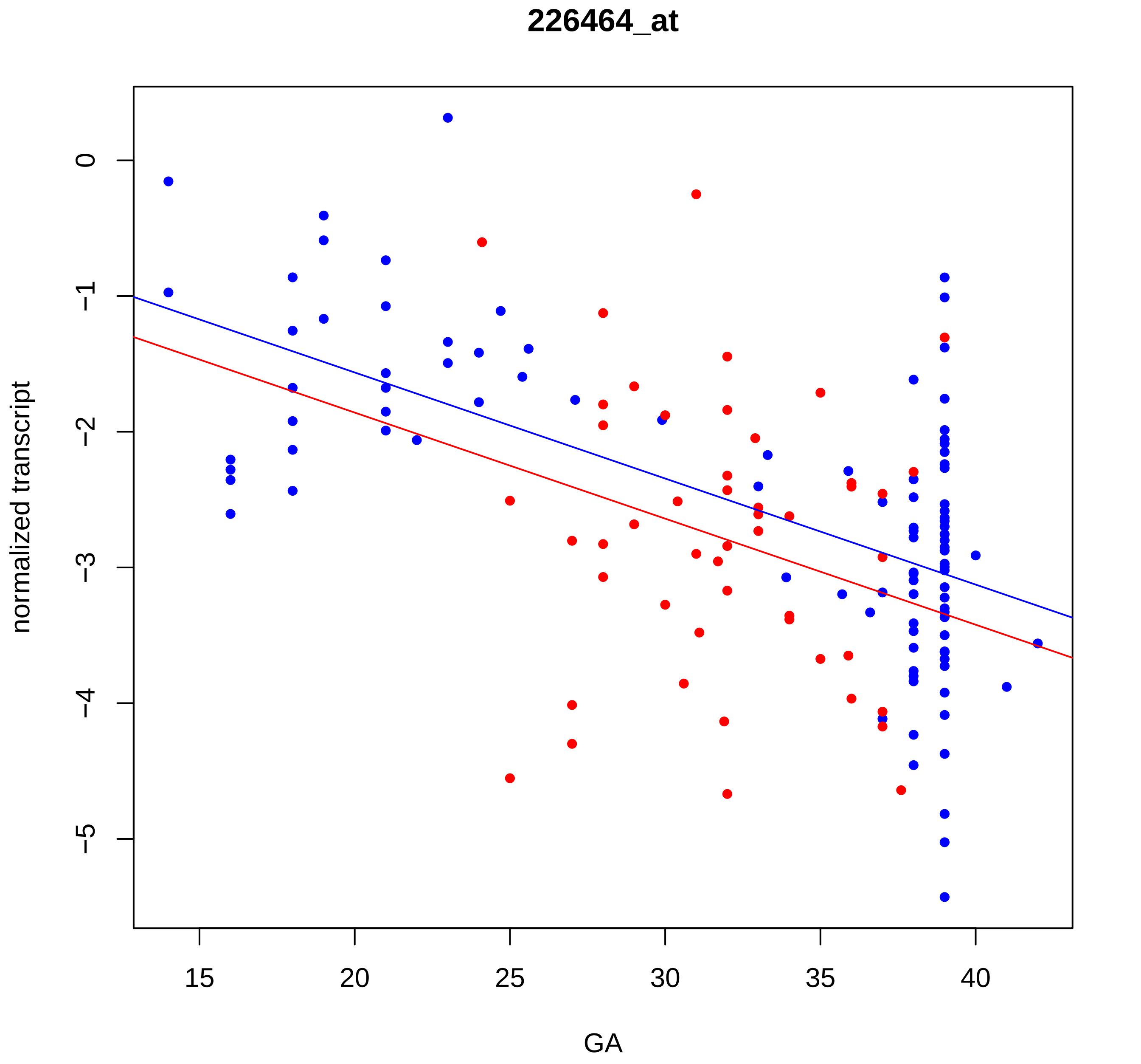

226464_at
normalized transcript
0
−1
−2
−3
−4
−5
15
20
25
30
35
40
GA

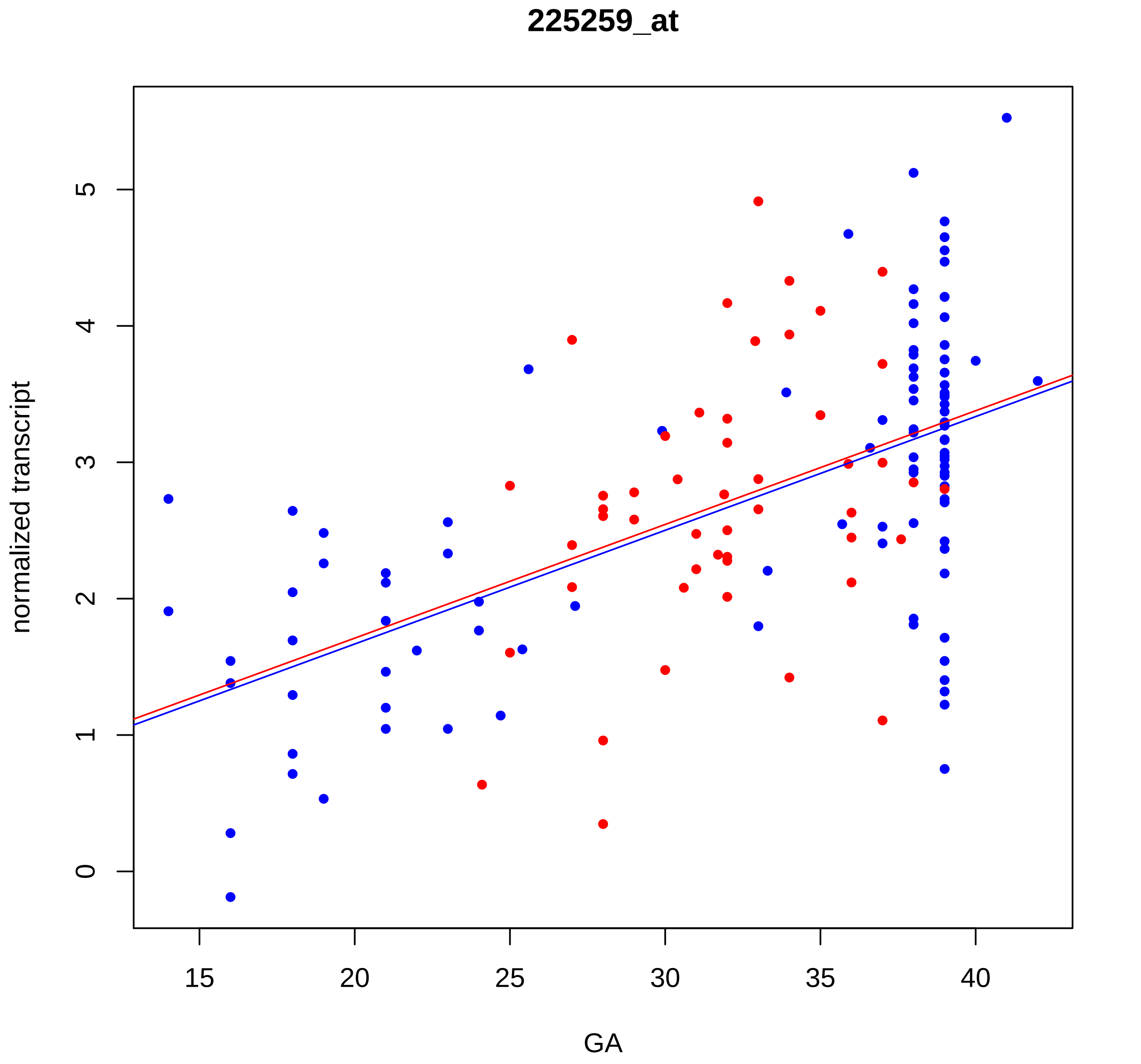
225259_at
normalized transcript
GA
0
1
2
3
4
5
15
20
25
30
35
40

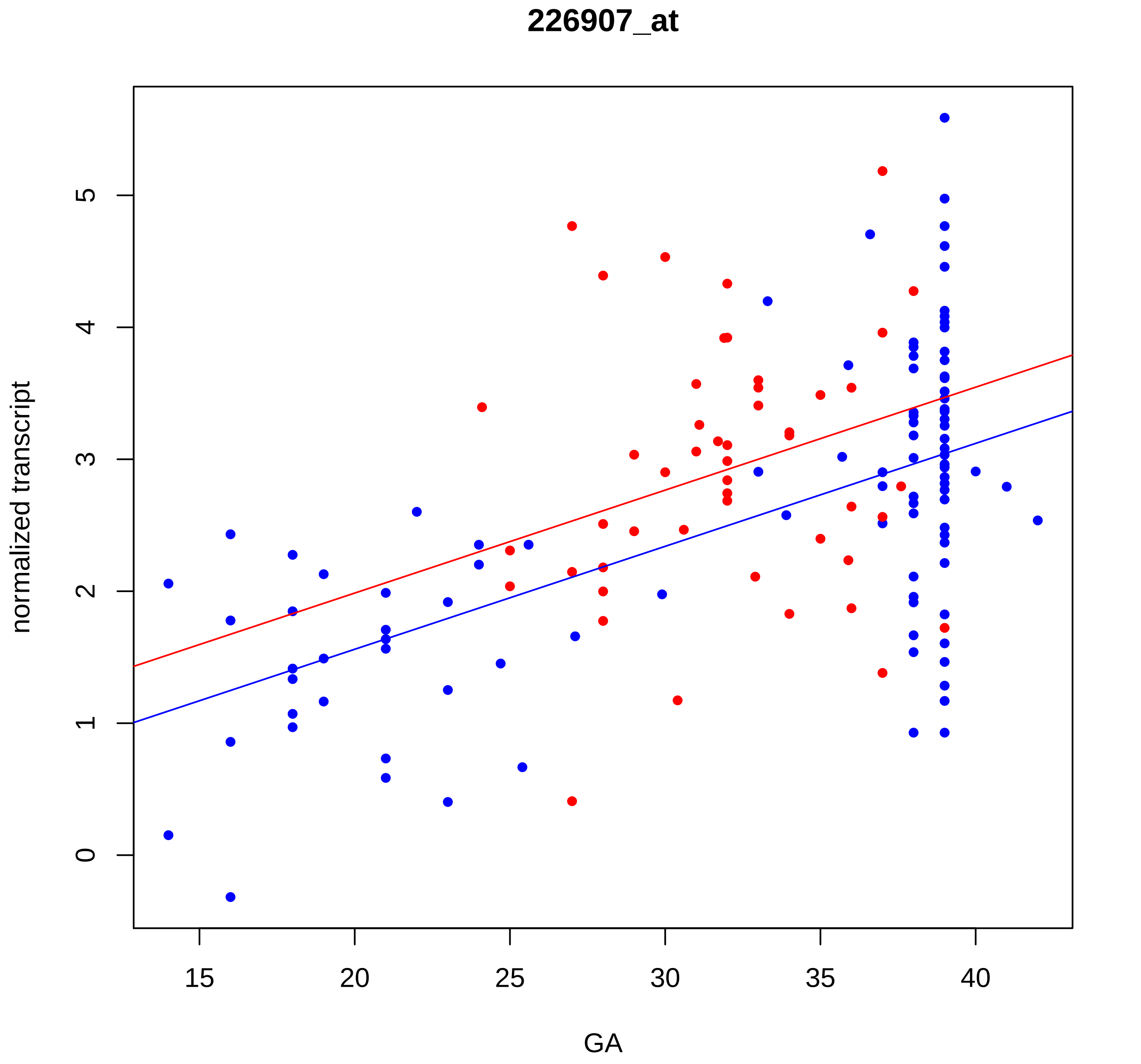

226907_at
normalized transcript
GA
0
1
2
3
4
5
15
20
25
30
35
40

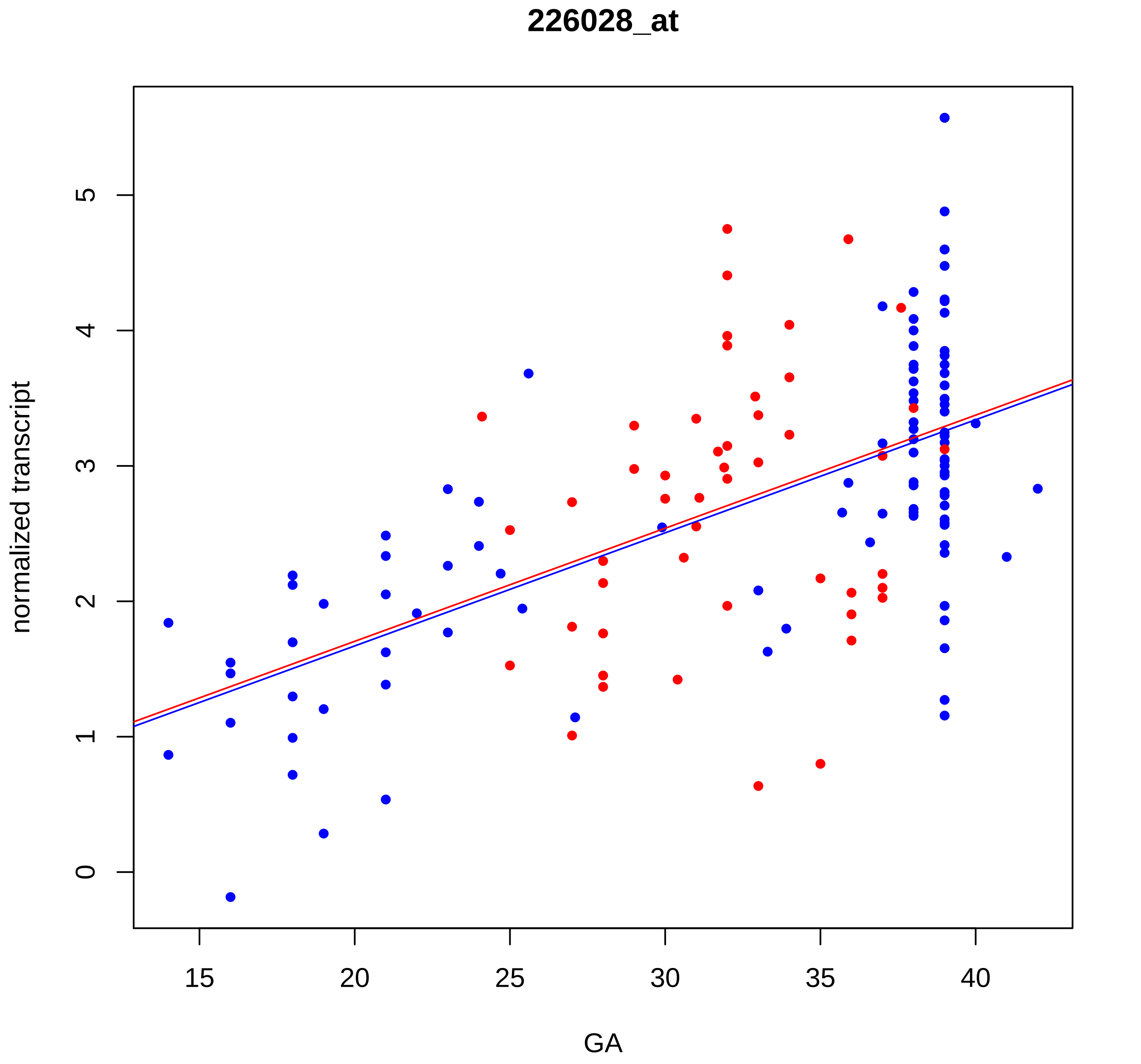

226028_at
normalized transcript
GA
0
1
2
3
4
5
15
20
25
30
35
40

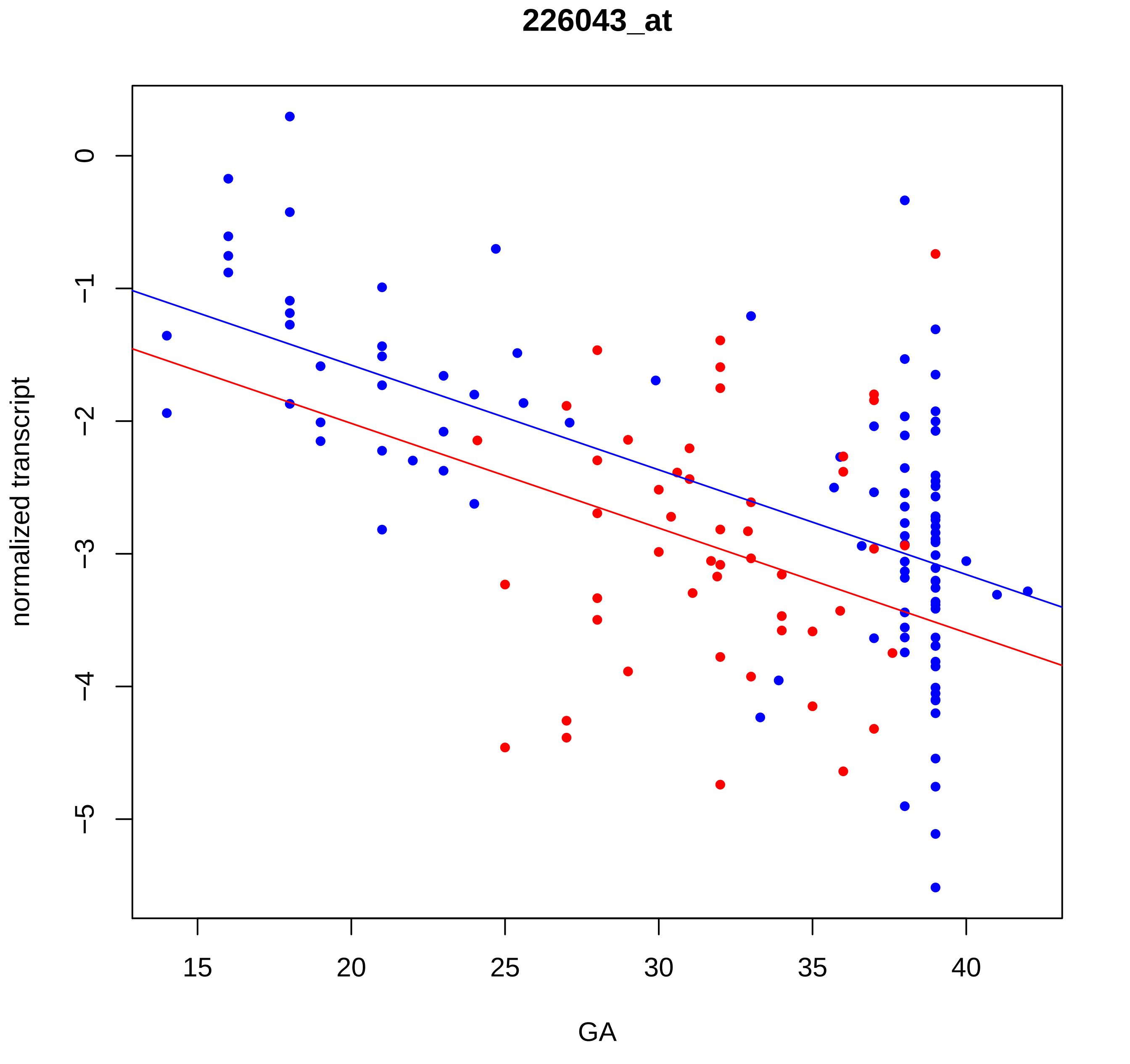

226043_at
0
−1
−2
−3
−4
−5
normalized transcript
15
20
25
30
35
40
GA

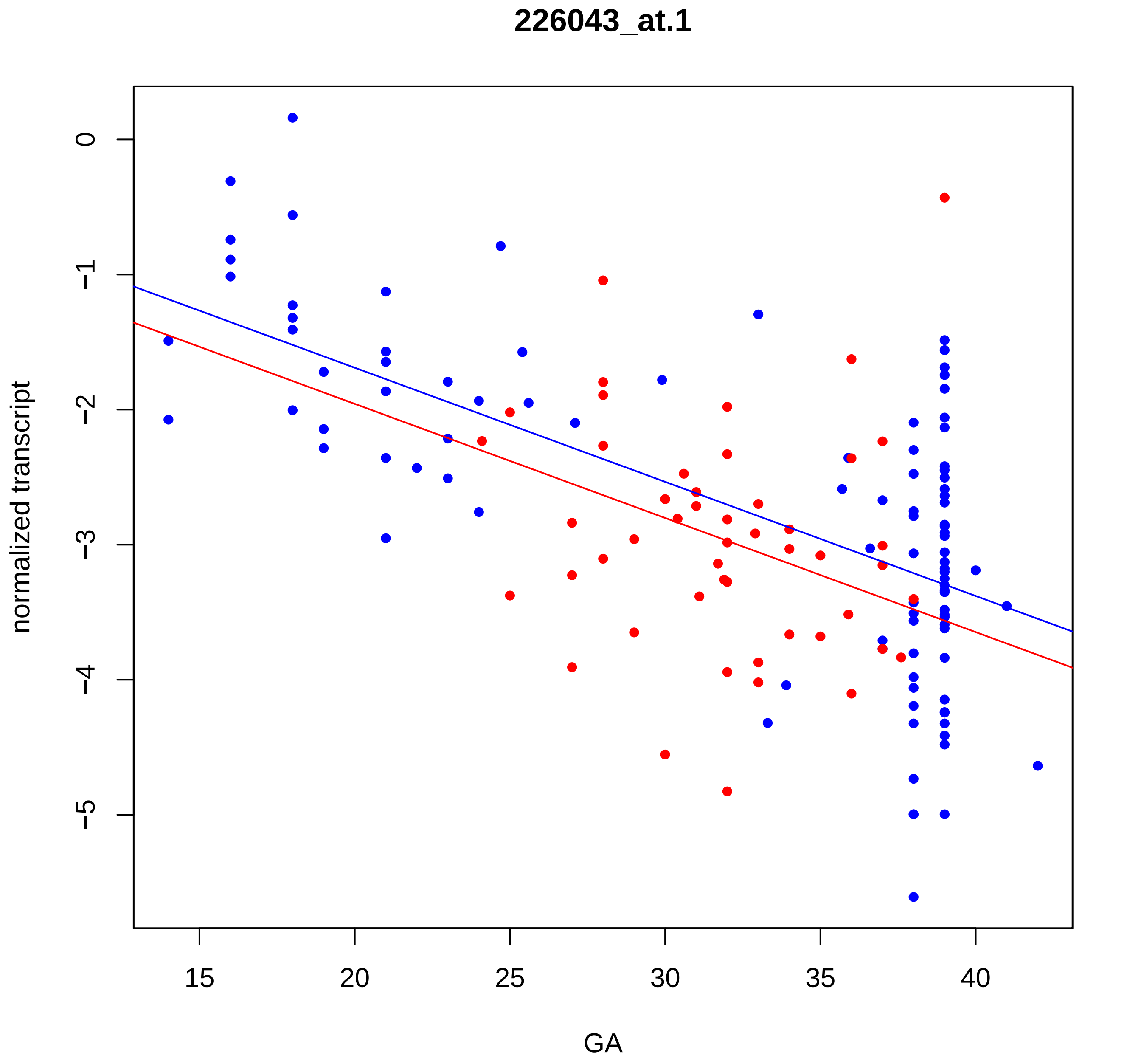
226043_at.1
normalized transcript
GA
0
−1
−2
−3
−4
−5
15
20
25
30
35
40

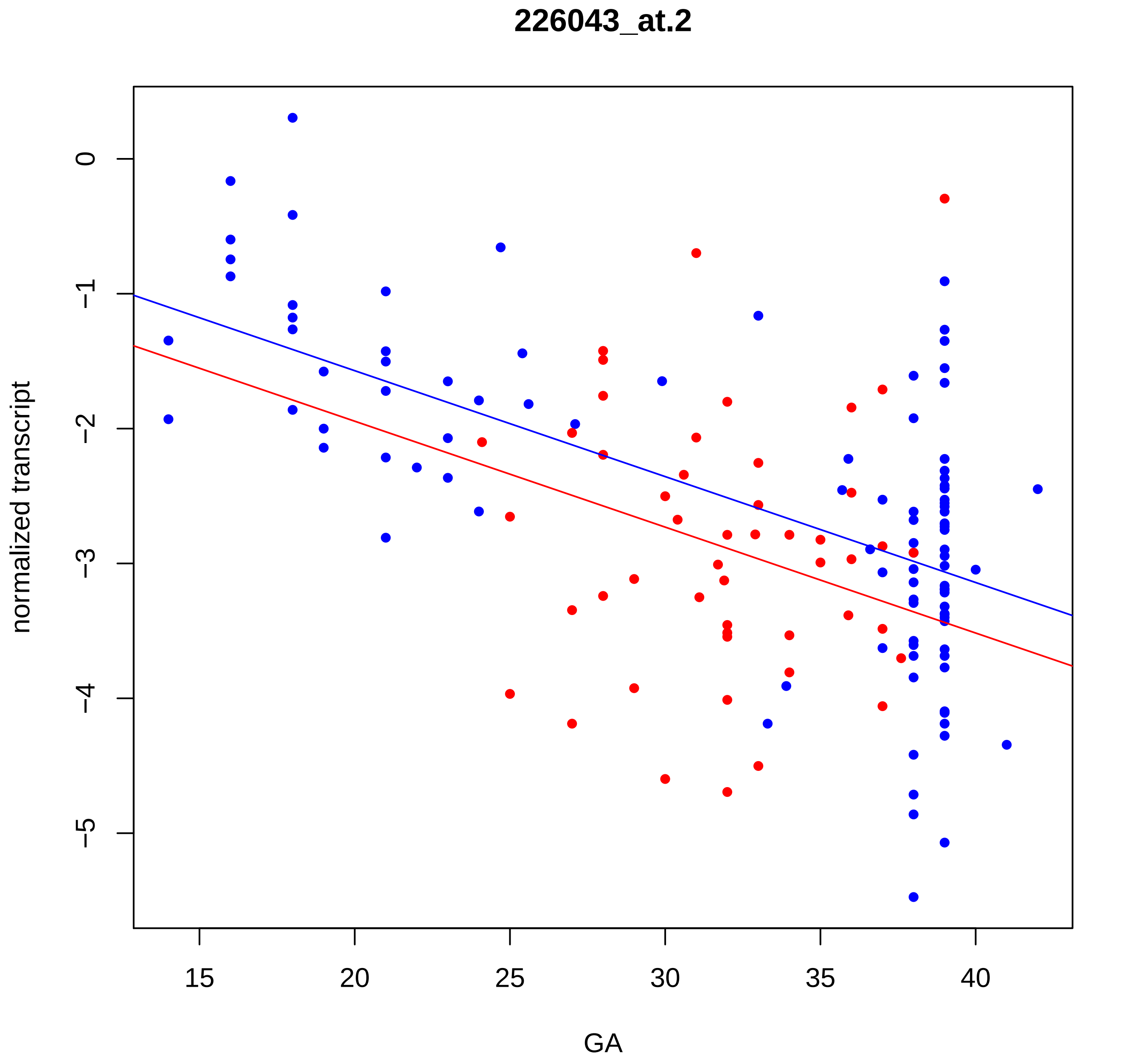

226043_at.2
normalized transcript
0
−1
−2
−3
−4
−5
15
20
25
30
35
40
GA

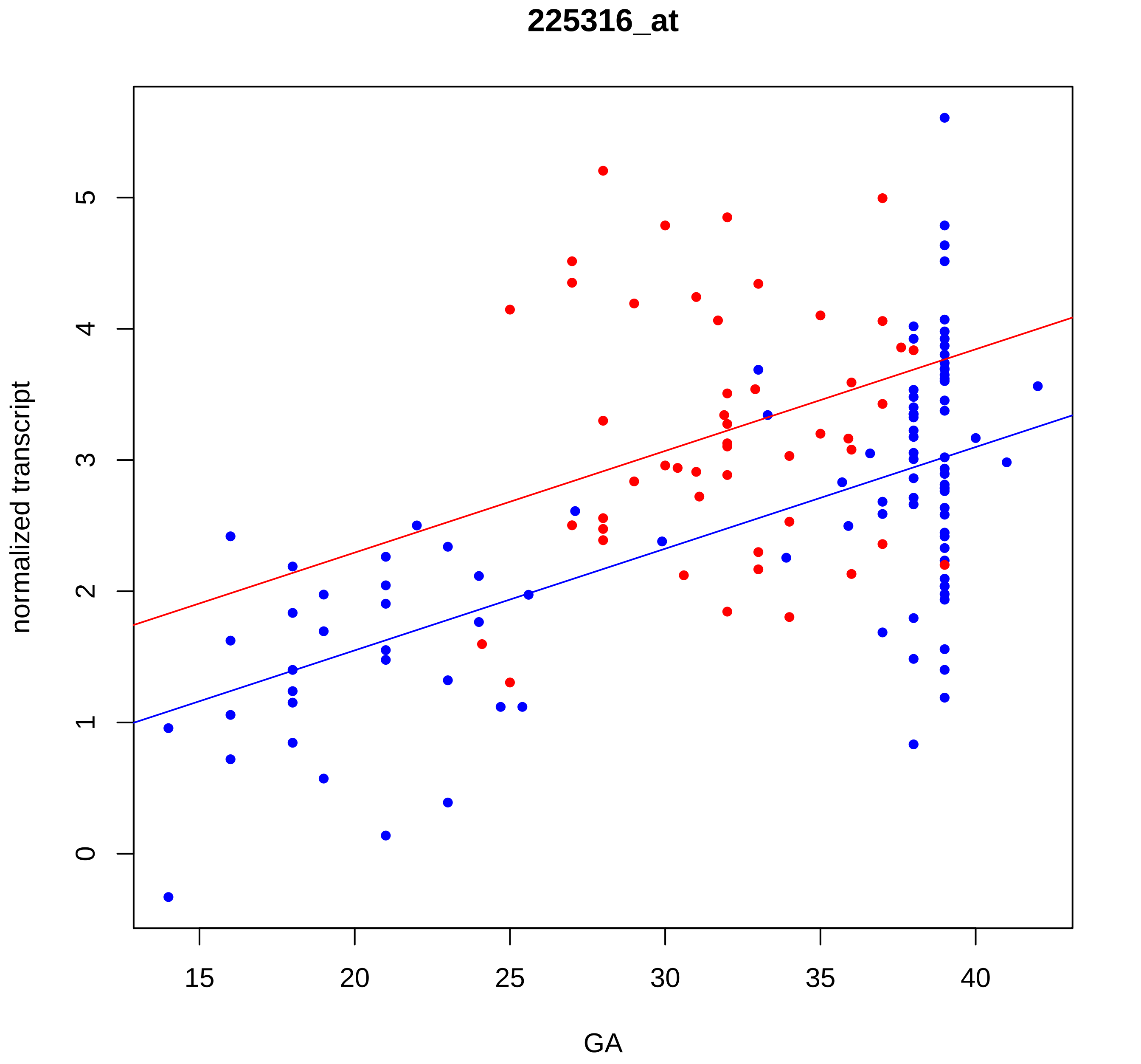
225316_at
normalized transcript
GA
0
1
2
3
4
5
15
20
25
30
35
40

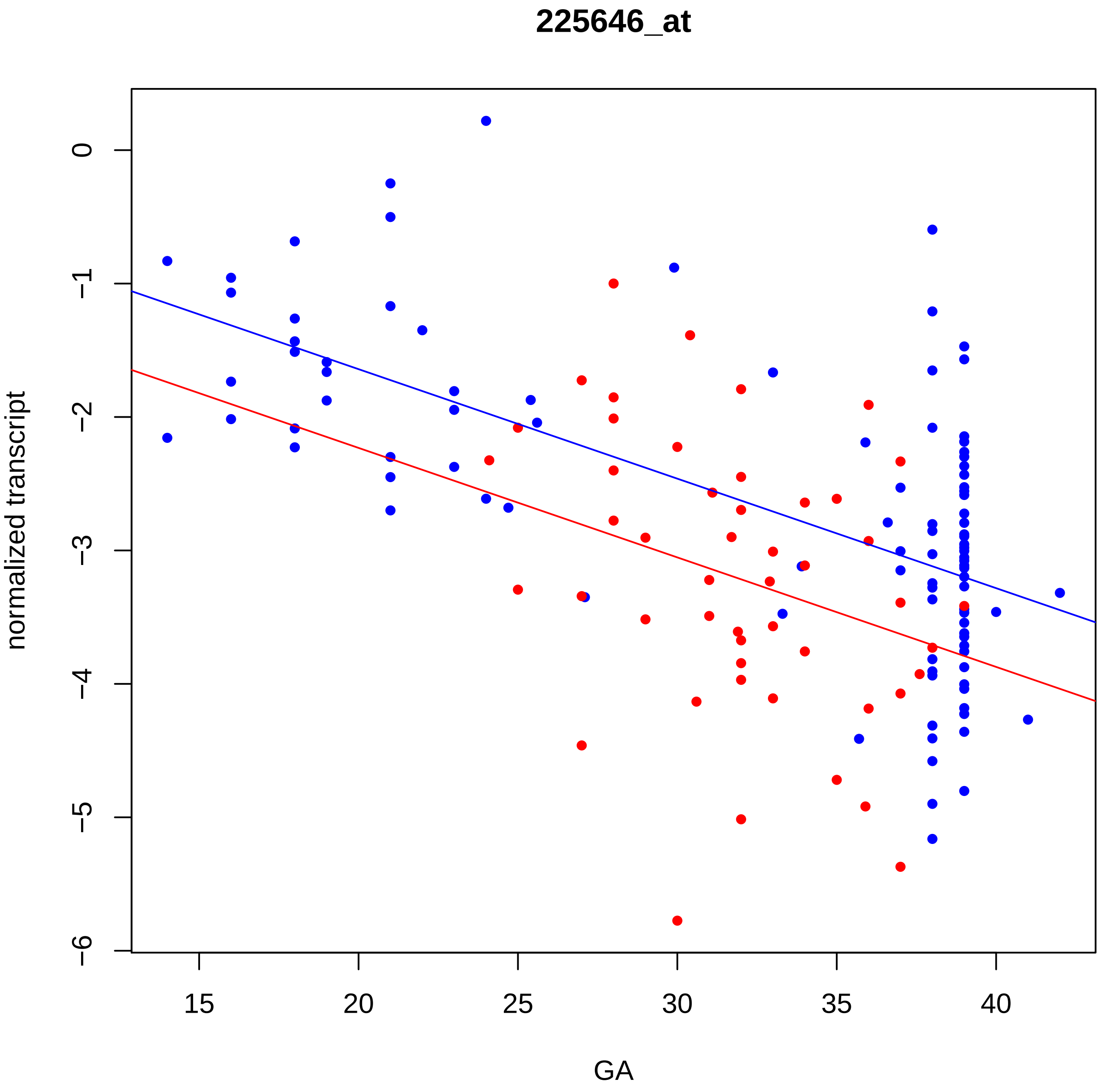

225646_at
normalized transcript
GA
0
−1
−2
−3
−4
−5
−6
15
20
25
30
35
40

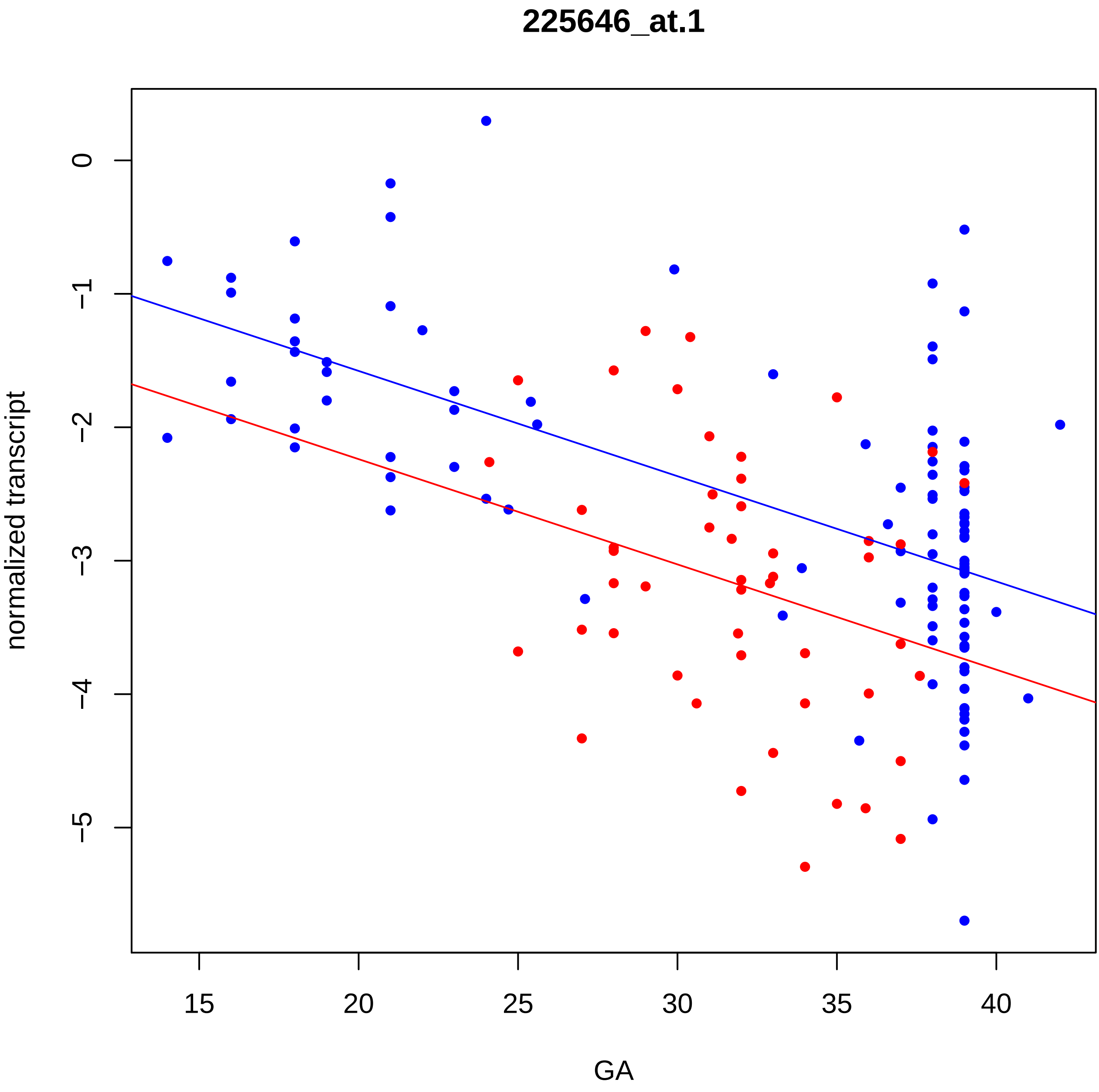
225646_at.1
normalized transcript
0
−1
−2
−3
−4
−5
15
20
25
30
35
40
GA

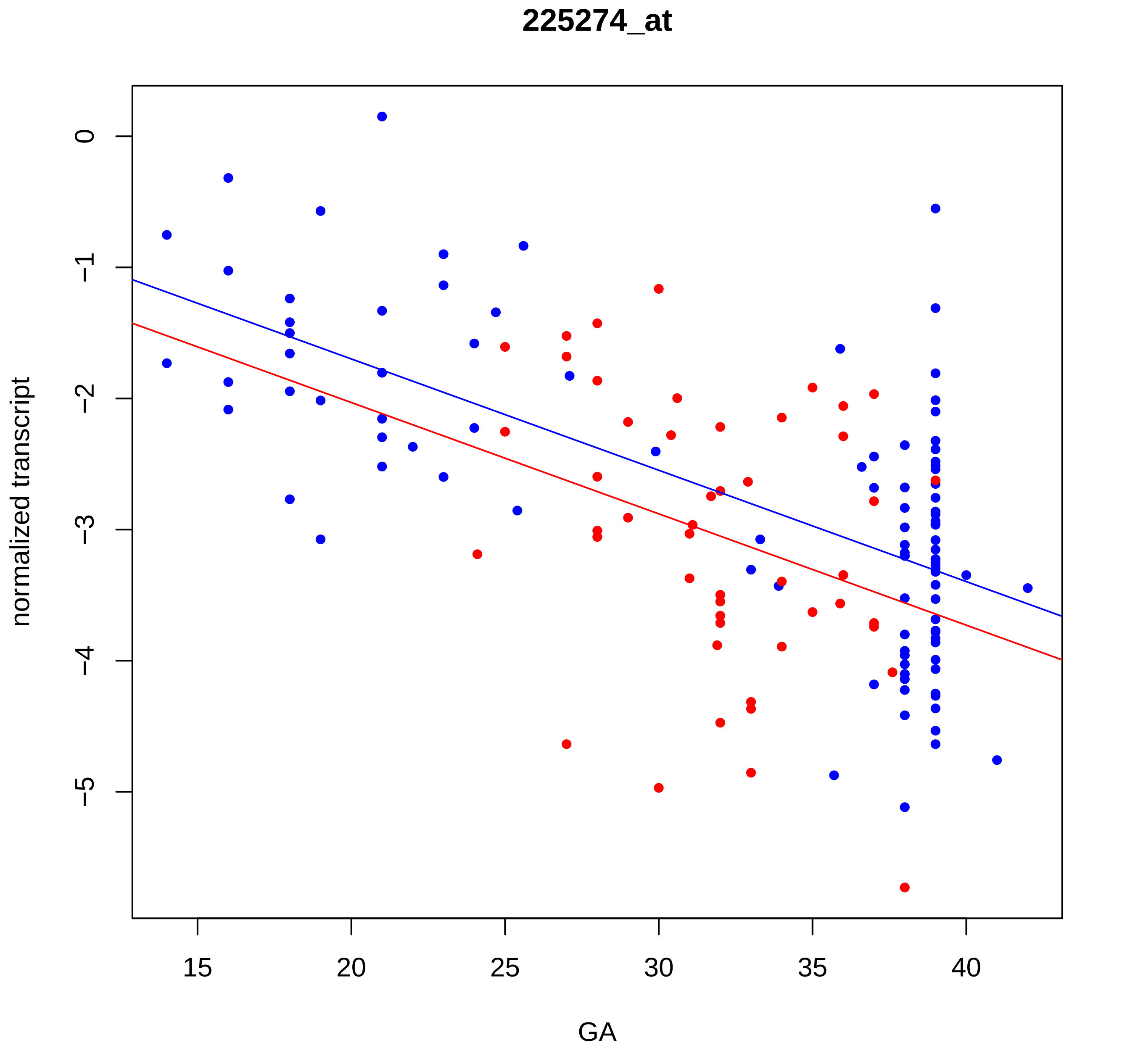
225274_at
normalized transcript
GA
0
−1
−2
−3
−4
−5
15
20
25
30
35
40

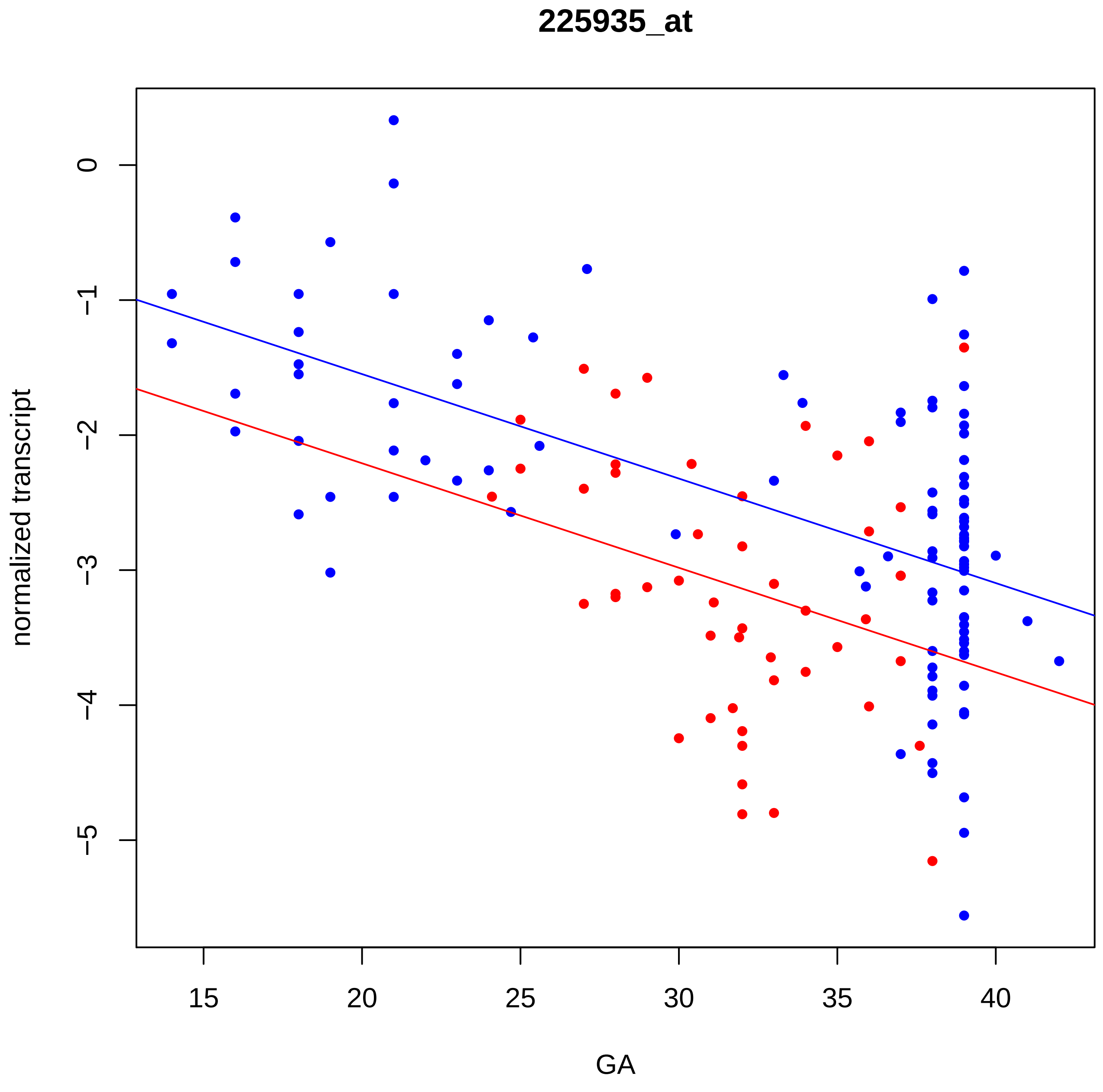
225935_at
normalized transcript
GA
0
−1
−2
−3
−4
−5
15
20
25
30
35
40

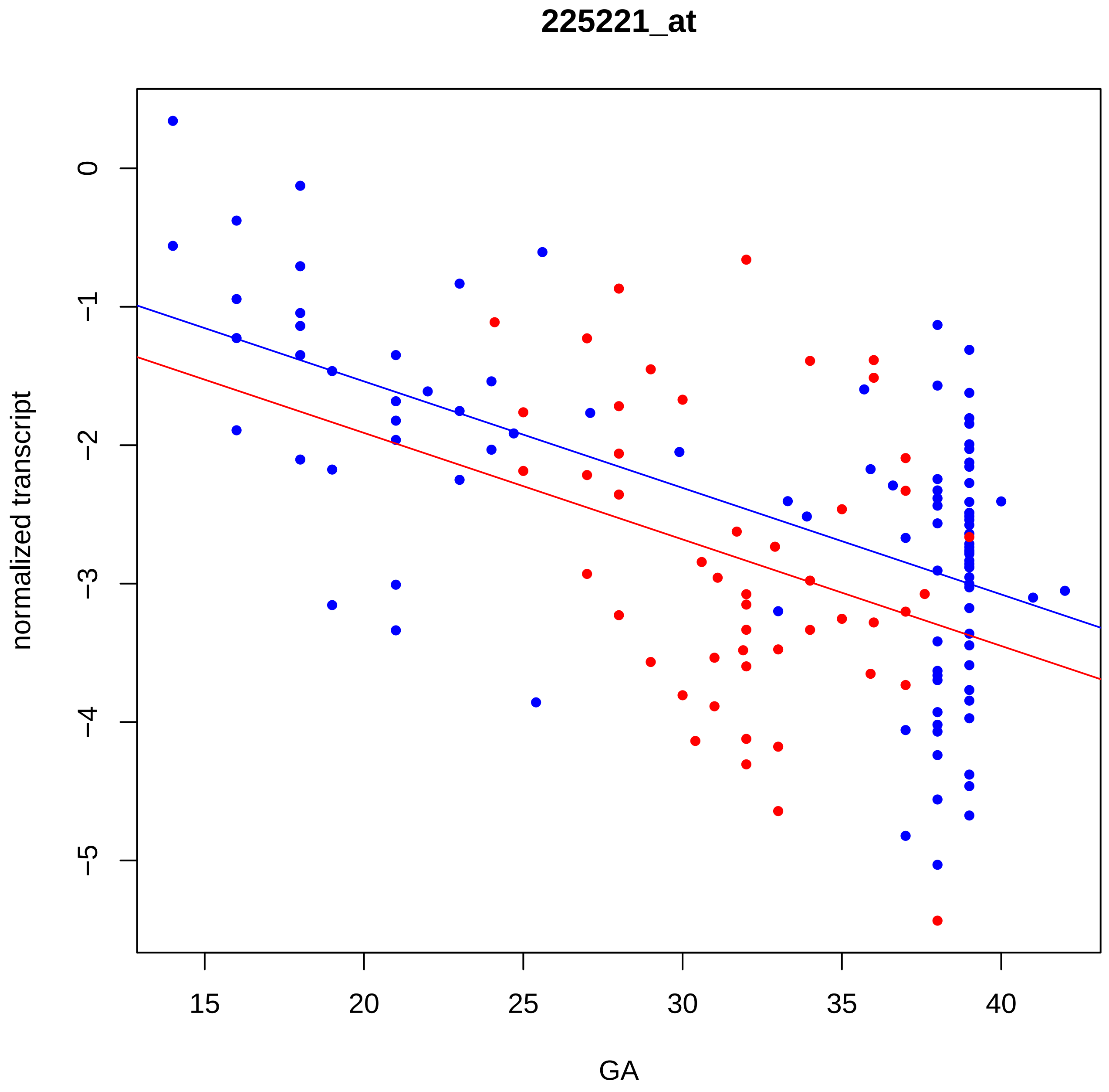
225221_at
normalized transcript
GA
0
−1
−2
−3
−4
−5
15
20
25
30
35
40

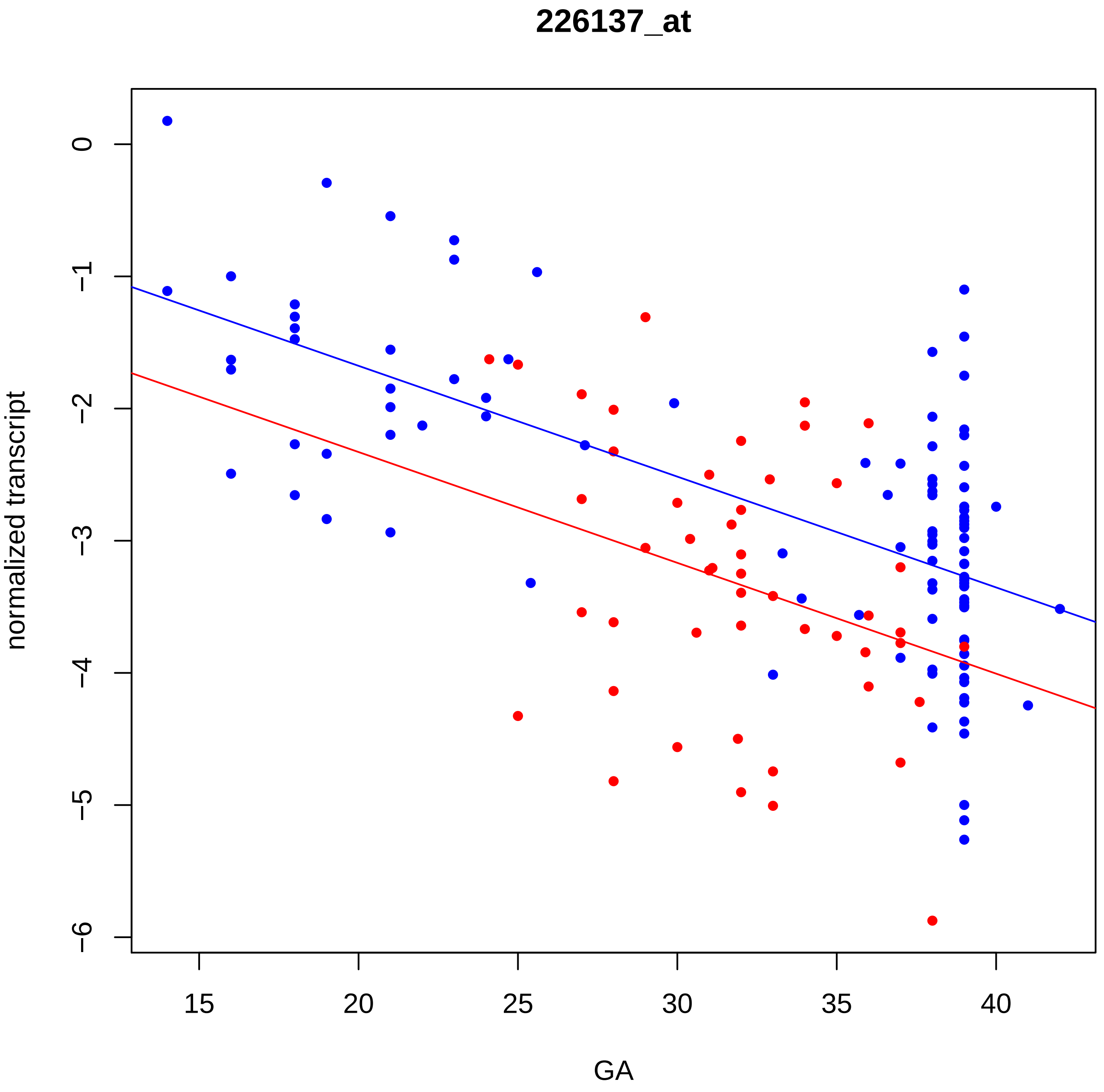
226137_at
normalized transcript
GA
0
−1
−2
−3
−4
−5
−6
15
20
25
30
35
40

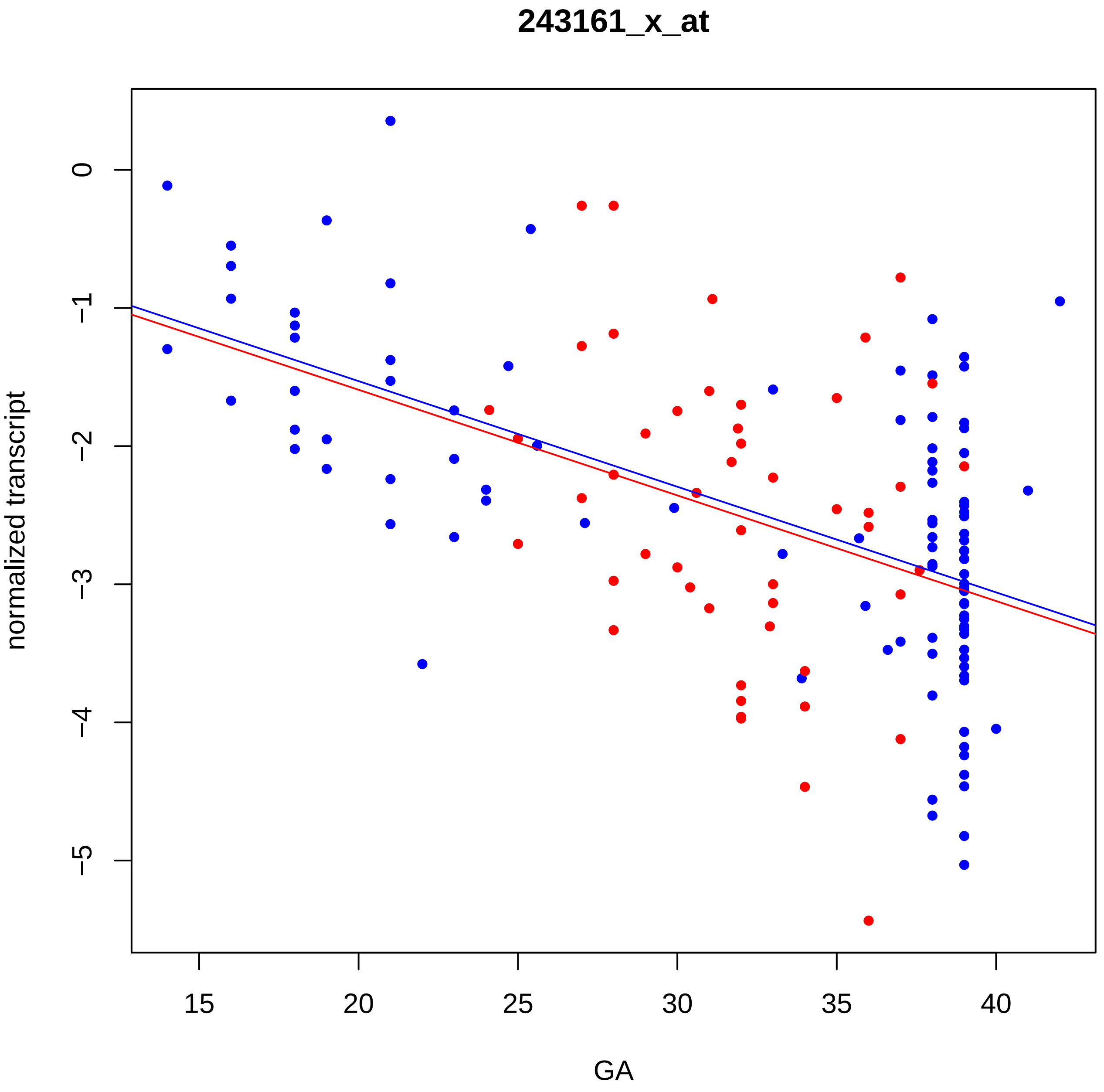

243161_x_at
normalized transcript
0
−1
−2
−3
−4
−5
15
20
25
30
35
40
GA

229623_at

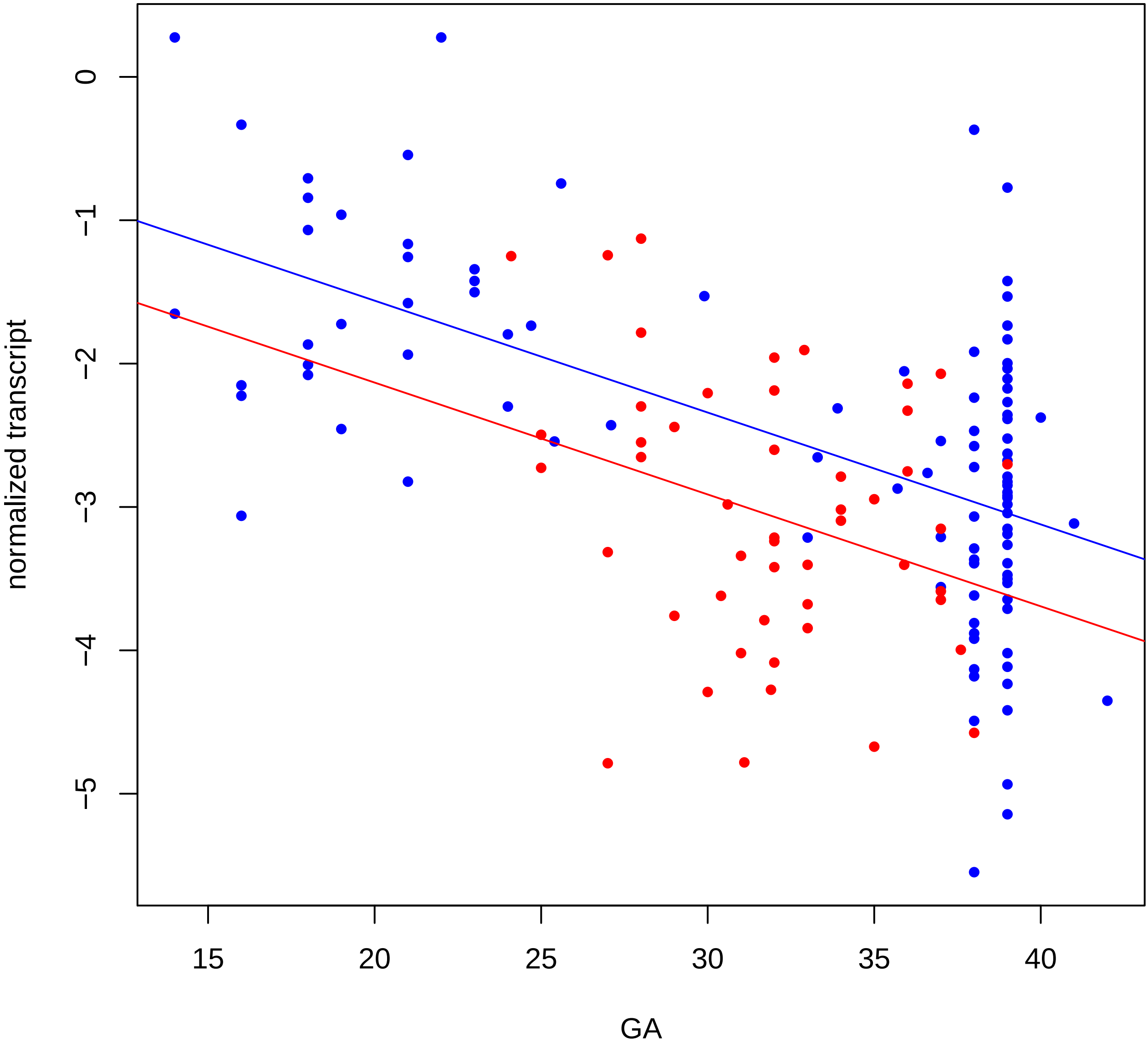

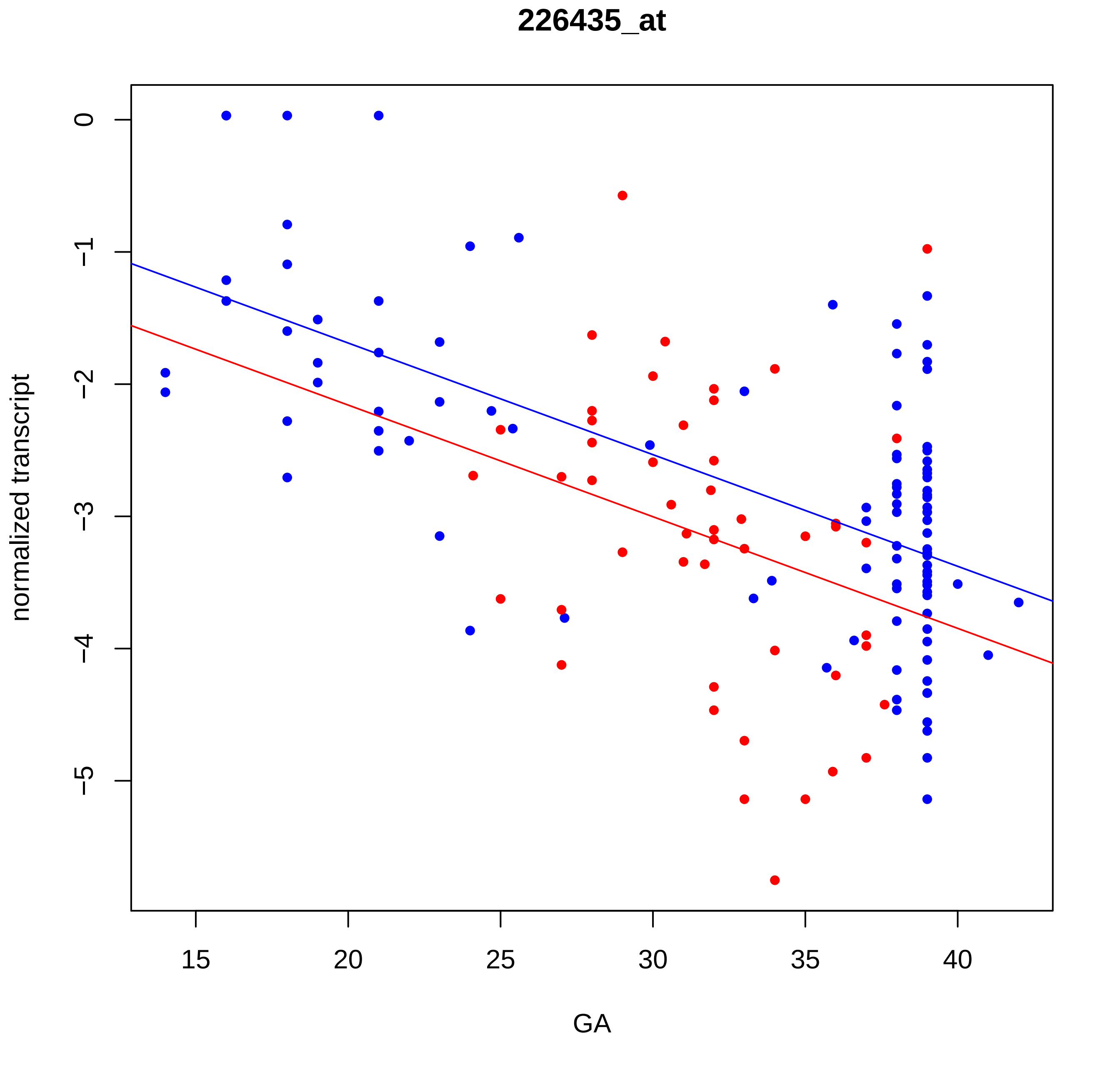
226435_at
normalized transcript
GA
0
−1
−2
−3
−4
−5
15
20
25
30
35
40

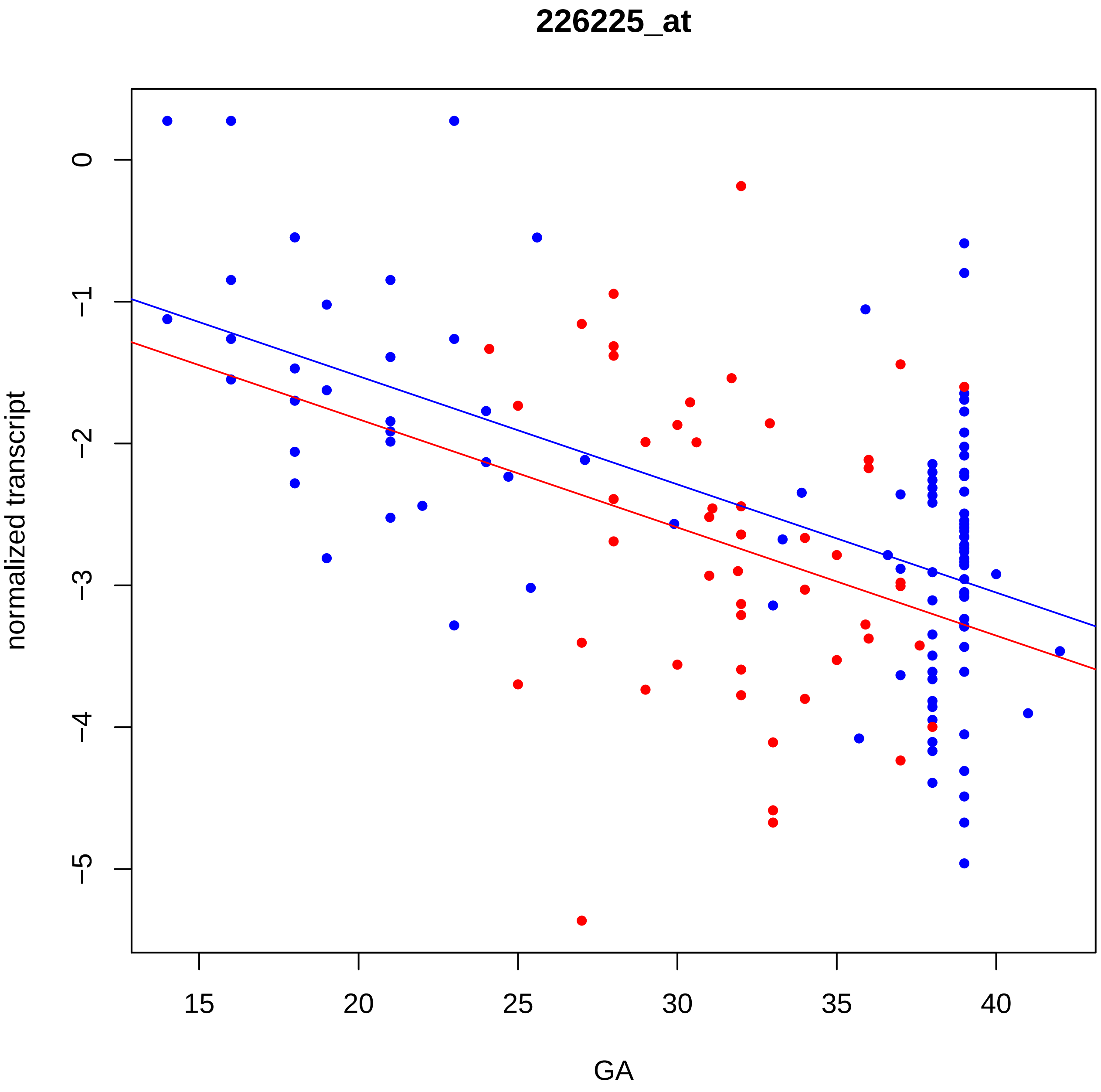

226225_at
normalized transcript
GA
0
−1
−2
−3
−4
−5
15
20
25
30
35
40

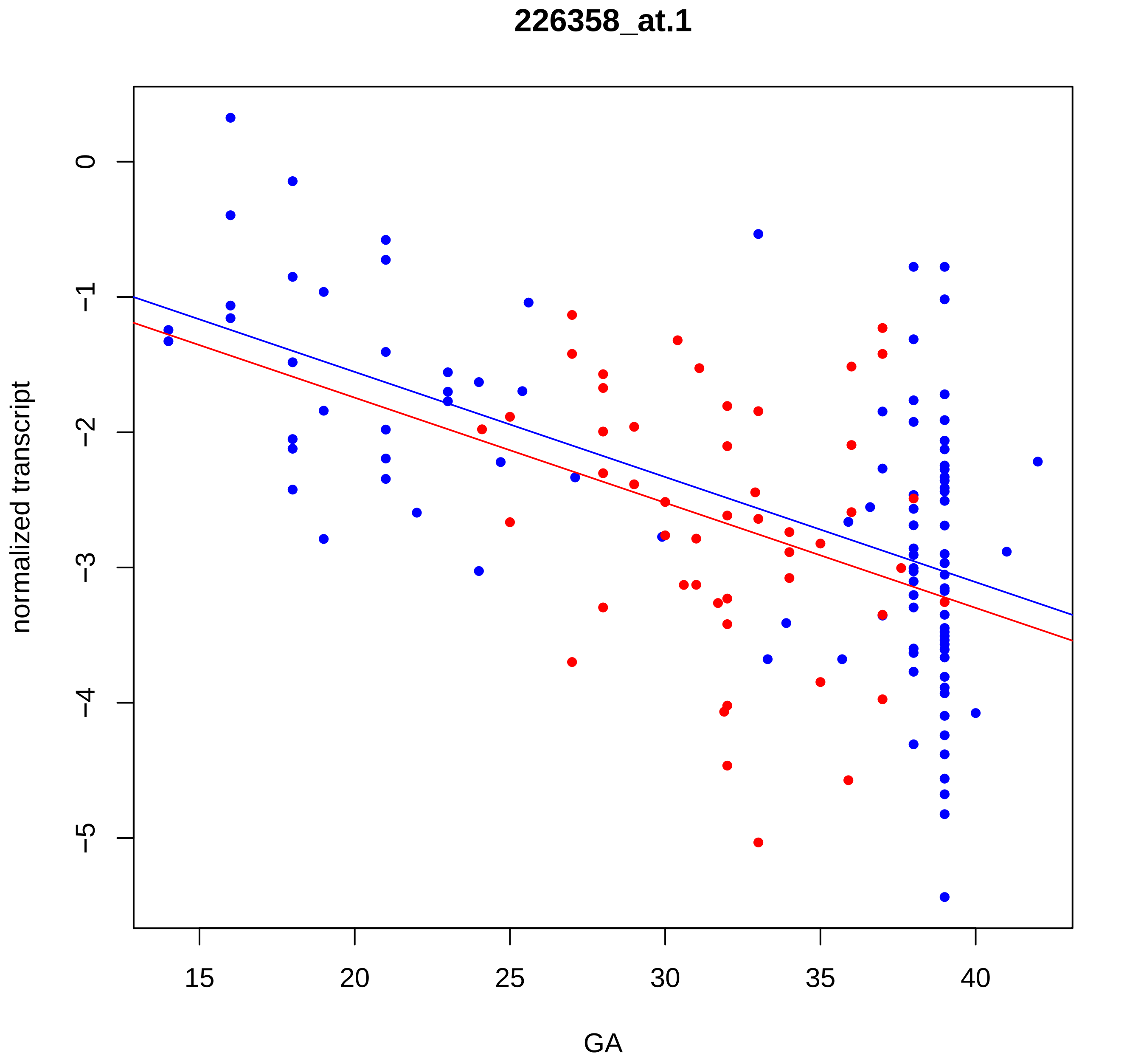

226358_at.1
normalized transcript
GA
0
−1
−2
−3
−4
−5
15
20
25
30
35
40

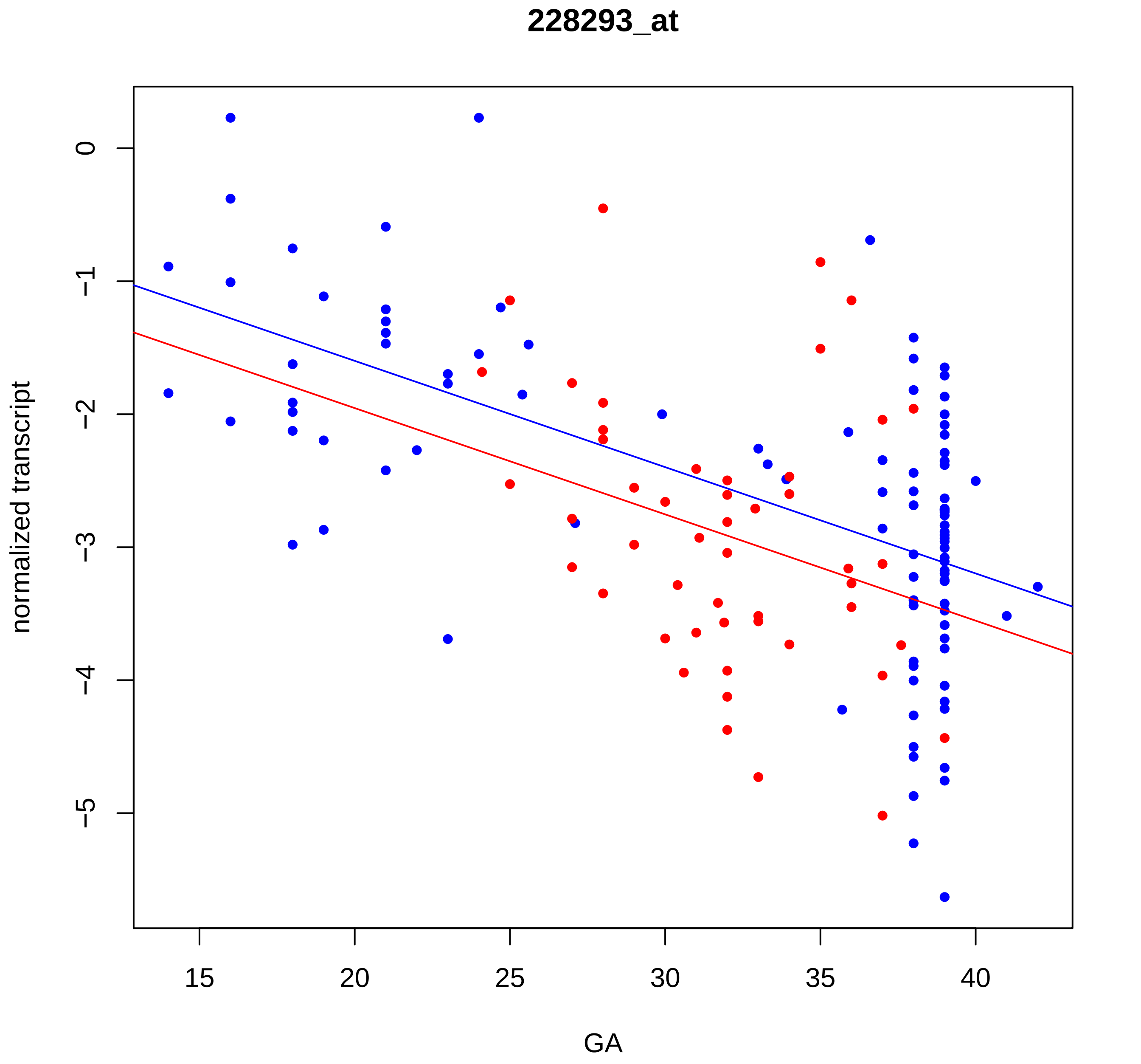

228293_at
normalized transcript
GA
0
−1
−2
−3
−4
−5
15
20
25
30
35
40

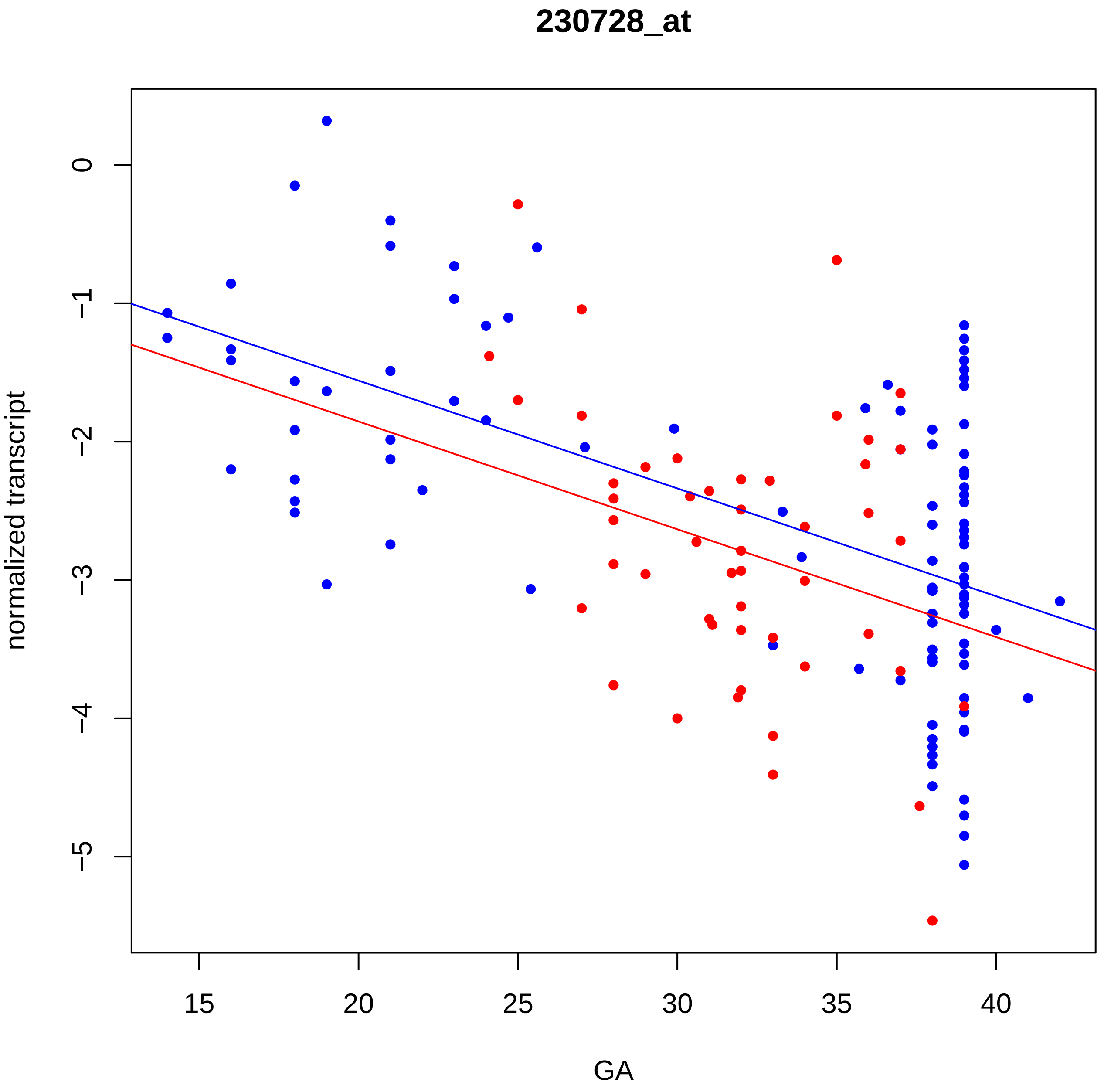

230728_at
normalized transcript
GA
0
−1
−2
−3
−4
−5
15
20
25
30
35
40

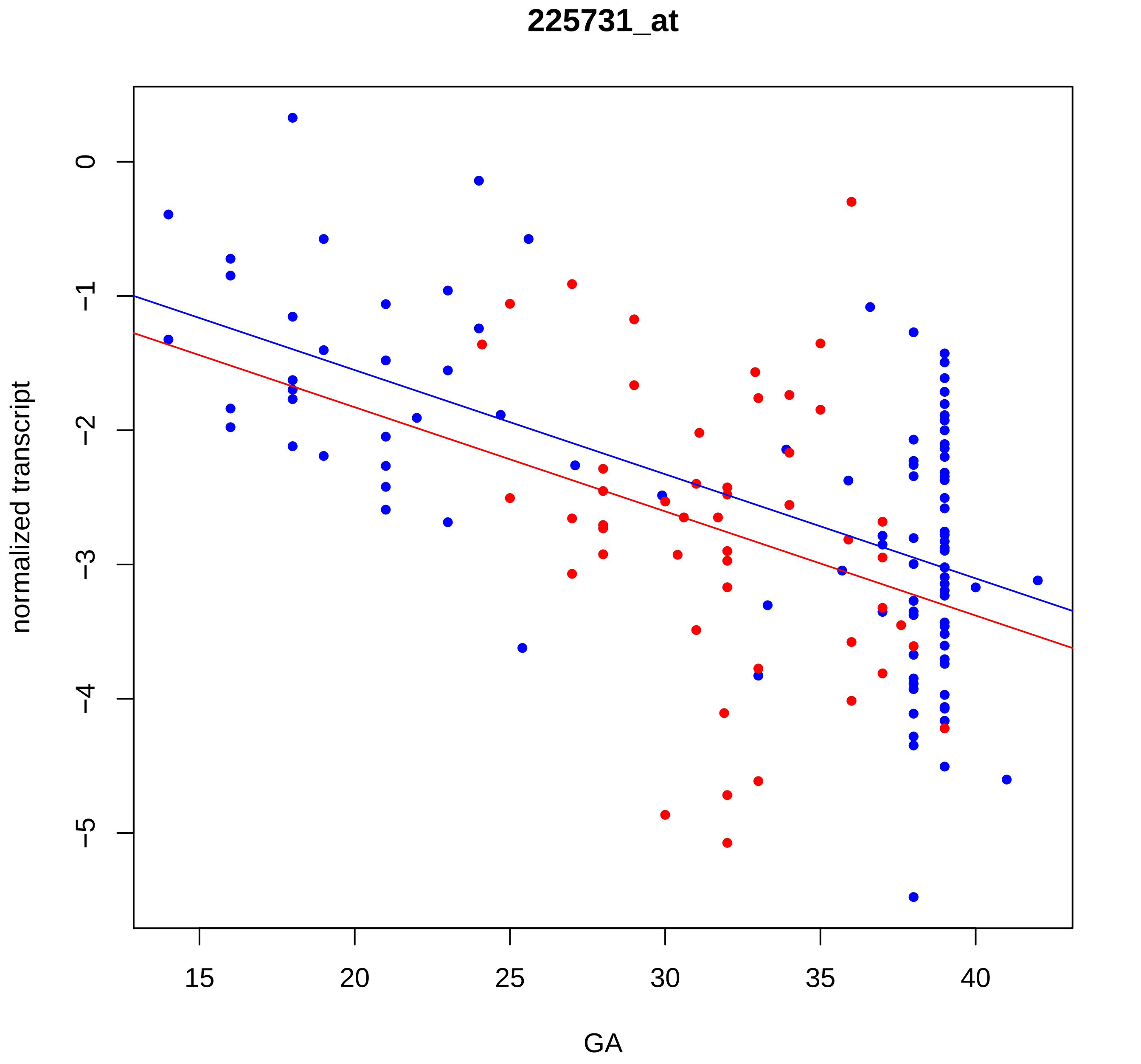

225731_at
0
−1
−2
−3
−4
−5
normalized transcript
15
20
25
30
35
40
GA

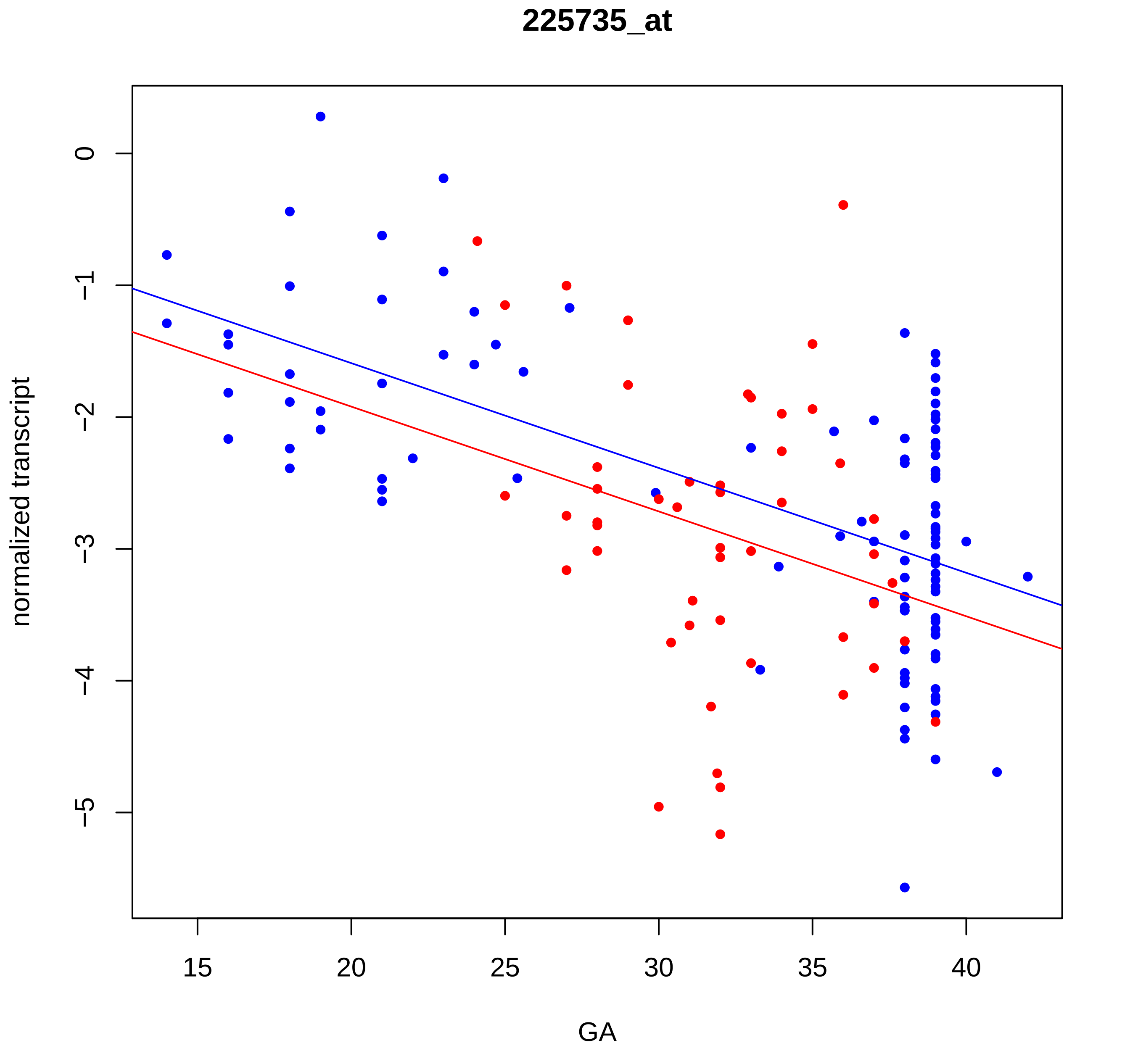

225735_at
0
−1
−2
−3
−4
−5
normalized transcript
15
20
25
30
35
40
GA

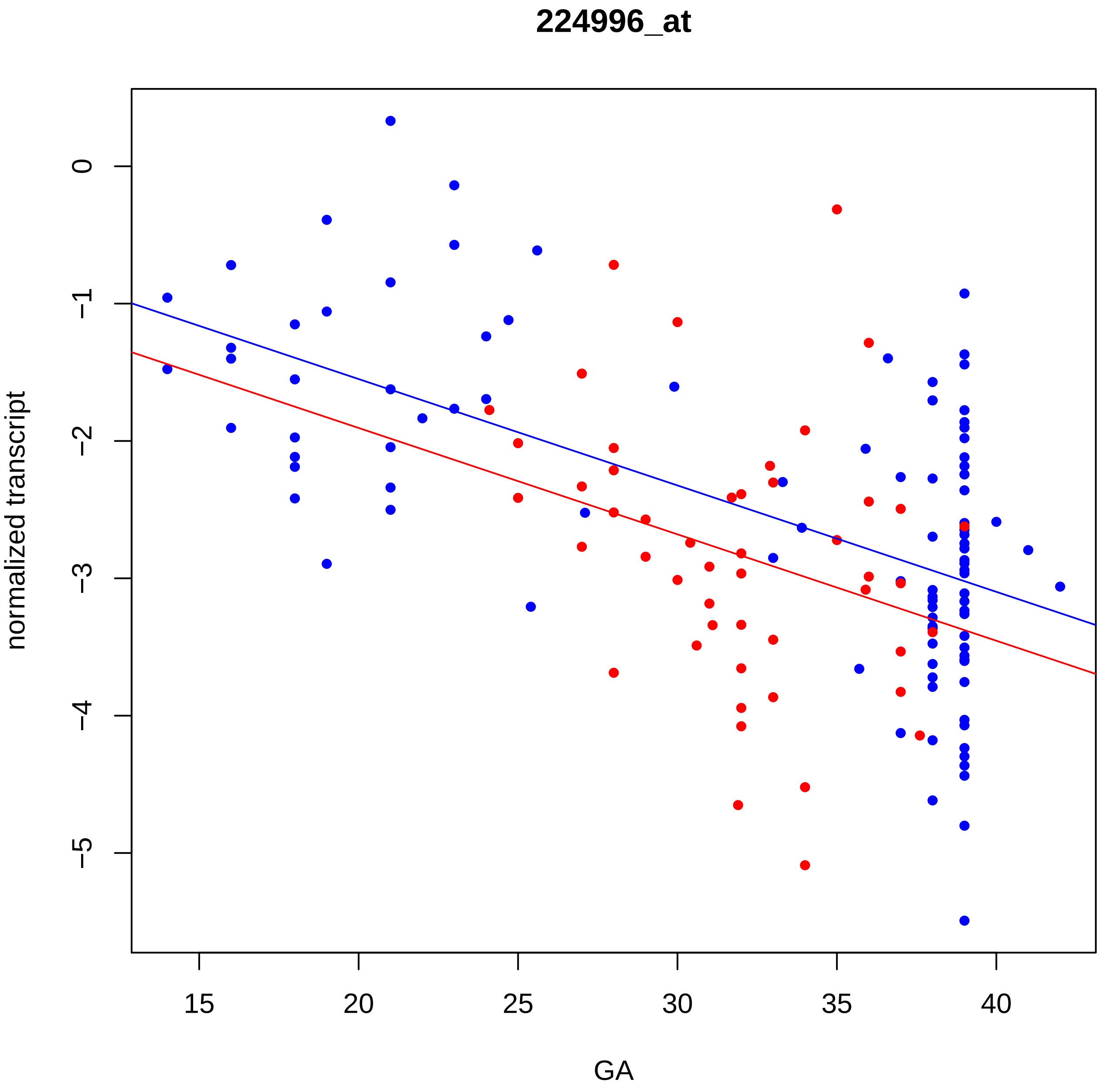

224996_at
0
−1
−2
−3
−4
−5
normalized transcript
15
20
25
30
35
40
GA

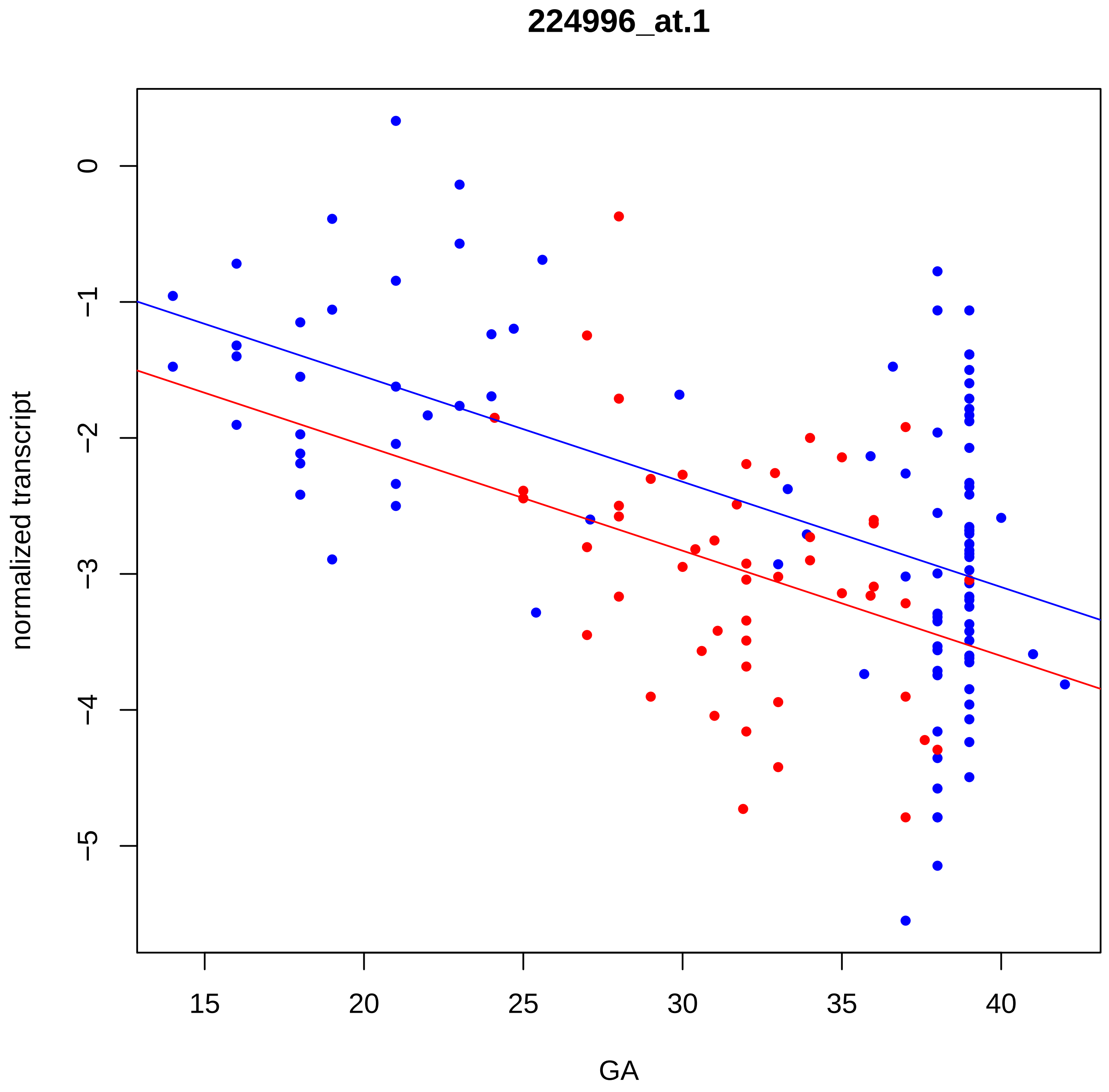

224996_at.1
normalized transcript
GA
0
−1
−2
−3
−4
−5
15
20
25
30
35
40

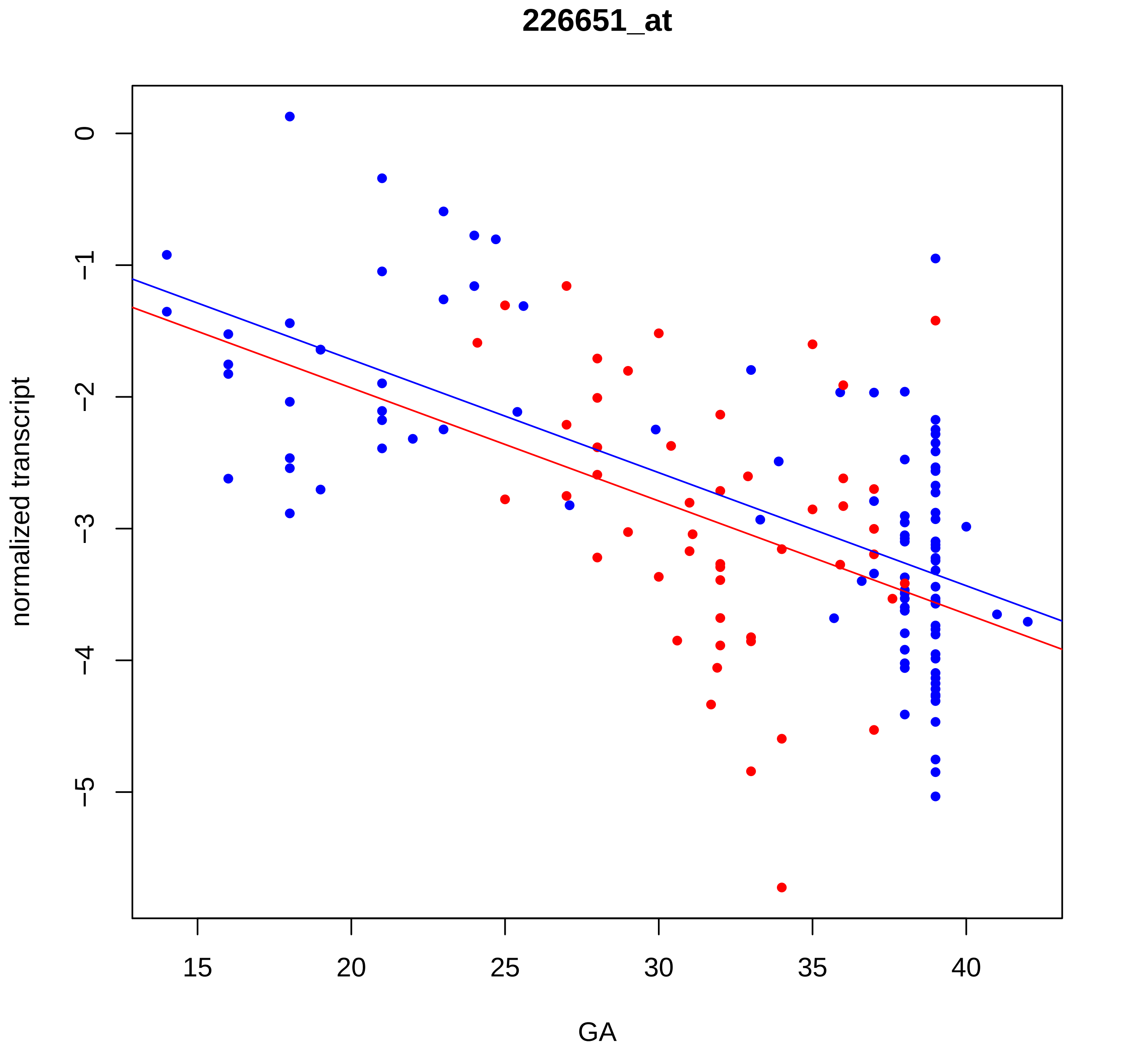
226651_at
normalized transcript
GA
0
−1
−2
−3
−4
−5
15
20
25
30
35
40

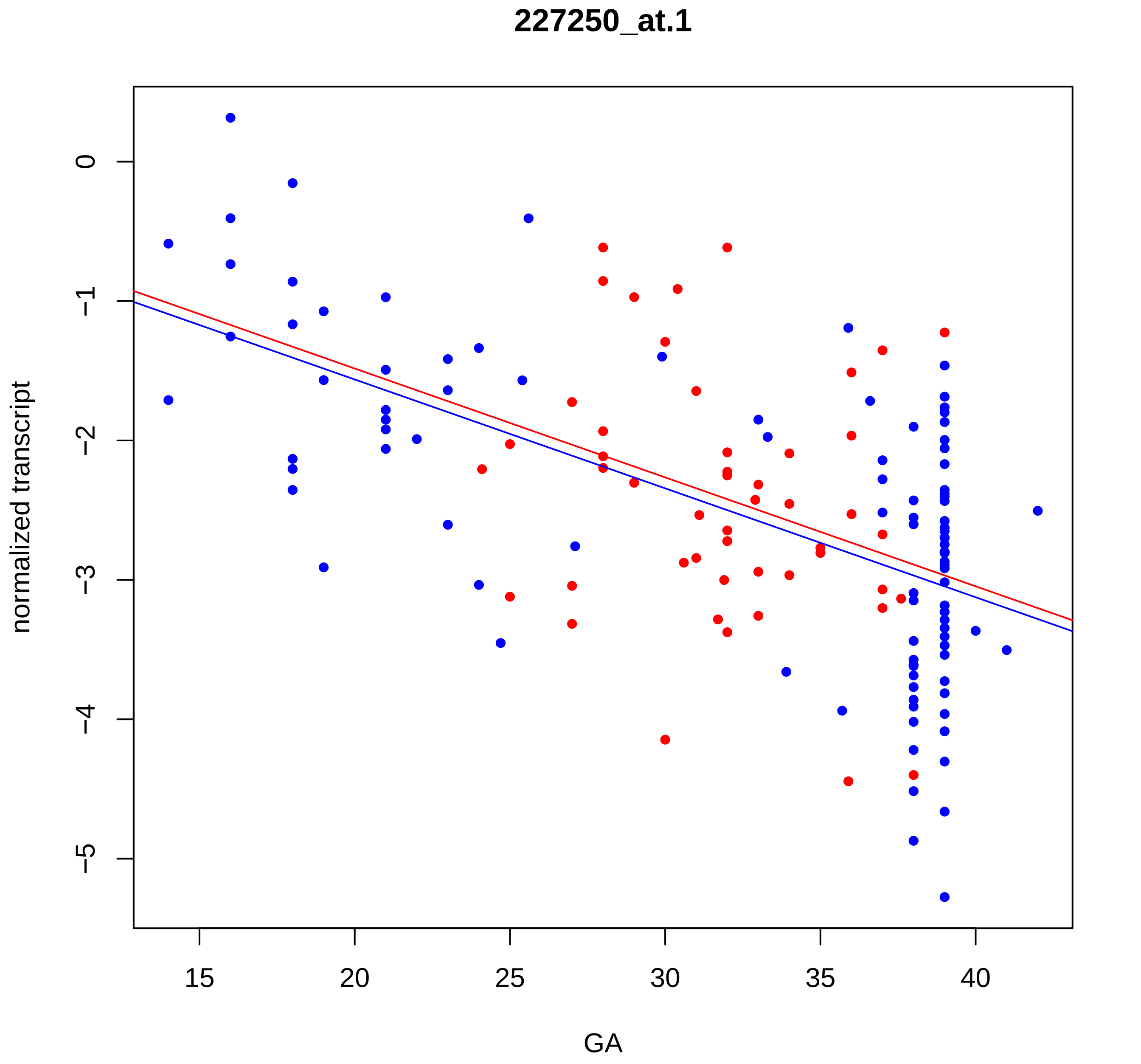
227250_at.1
normalized transcript
GA
0
−1
−2
−3
−4
−5
15
20
25
30
35
40

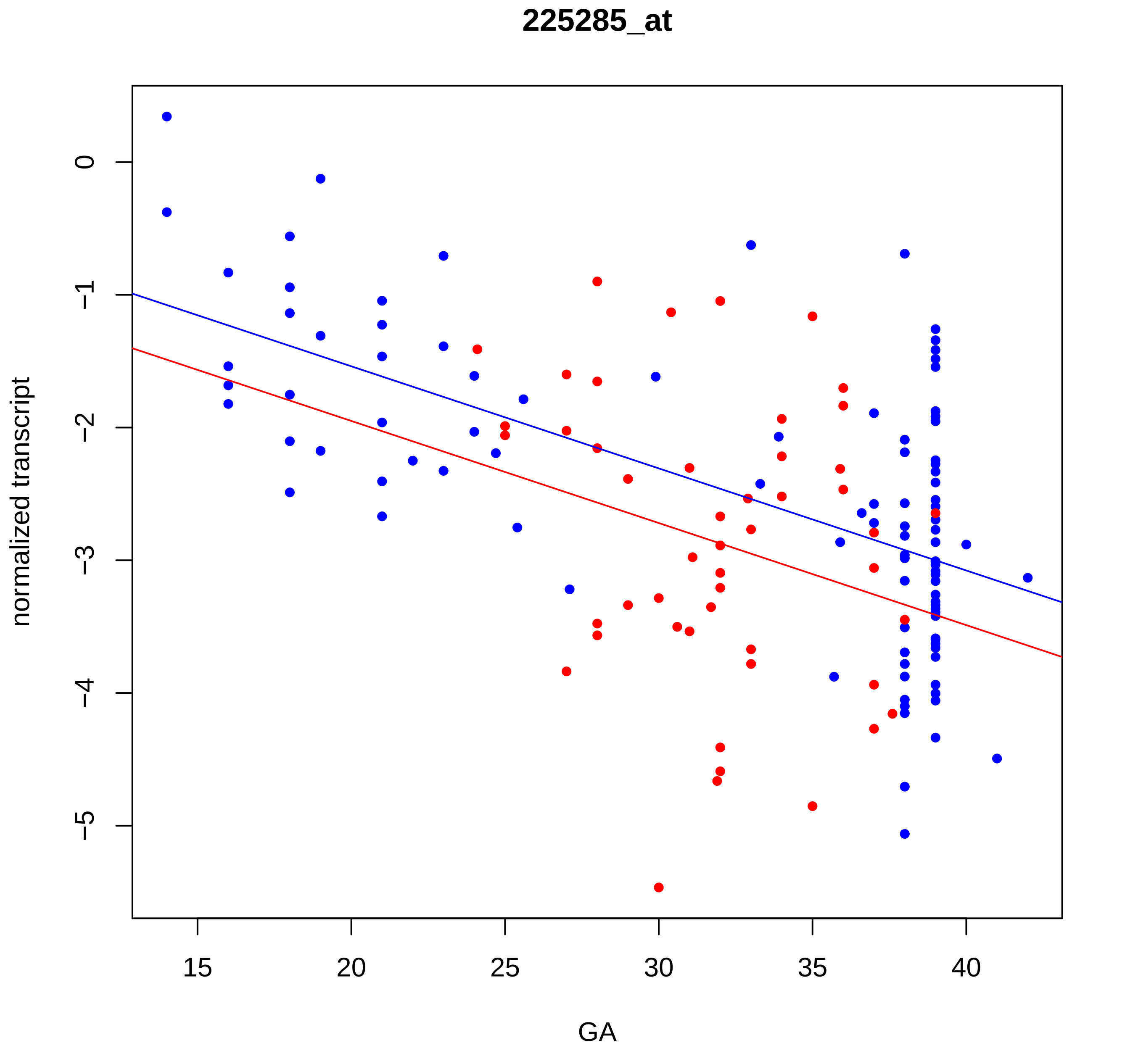

225285_at
normalized transcript
GA
0
−1
−2
−3
−4
−5
15
20
25
30
35
40

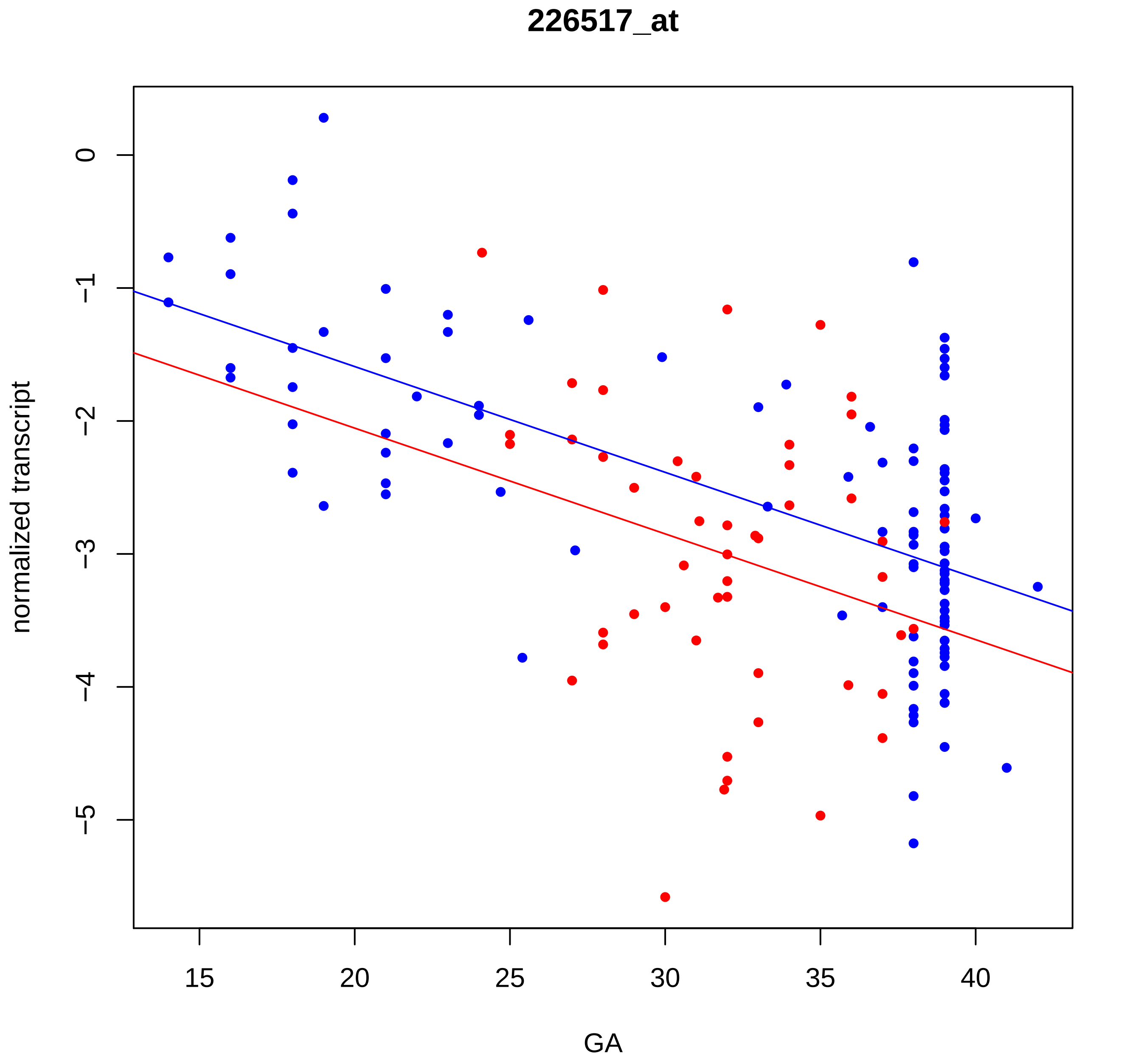

226517_at
0
−1
−2
−3
−4
−5
normalized transcript
15
20
25
30
35
40
GA

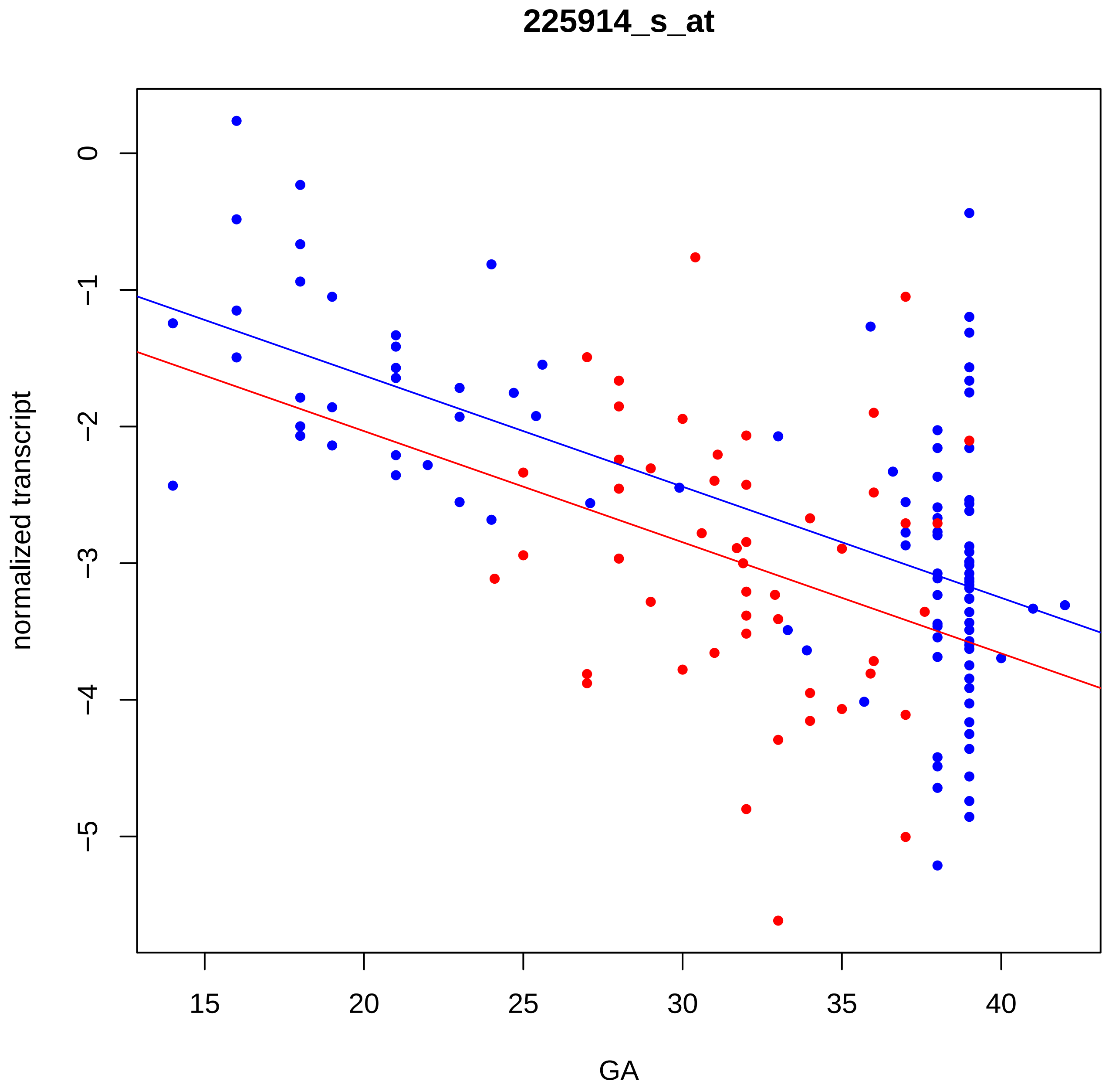

225914_s_at
normalized transcript
0
−1
−2
−3
−4
−5
15
20
25
30
35
40
GA

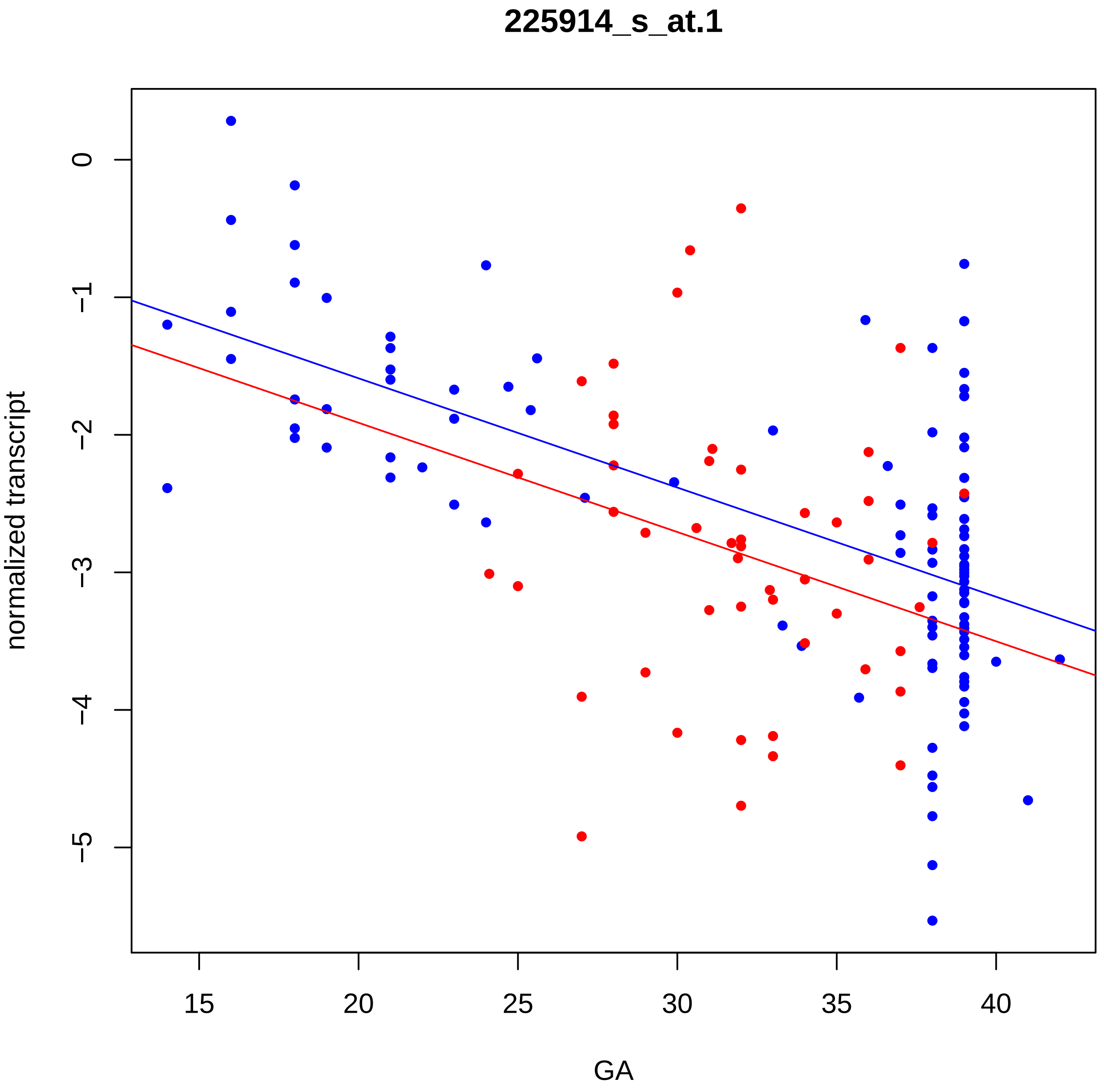
225914_s_at.1
normalized transcript
GA
0
−1
−2
−3
−4
−5
15
20
25
30
35
40

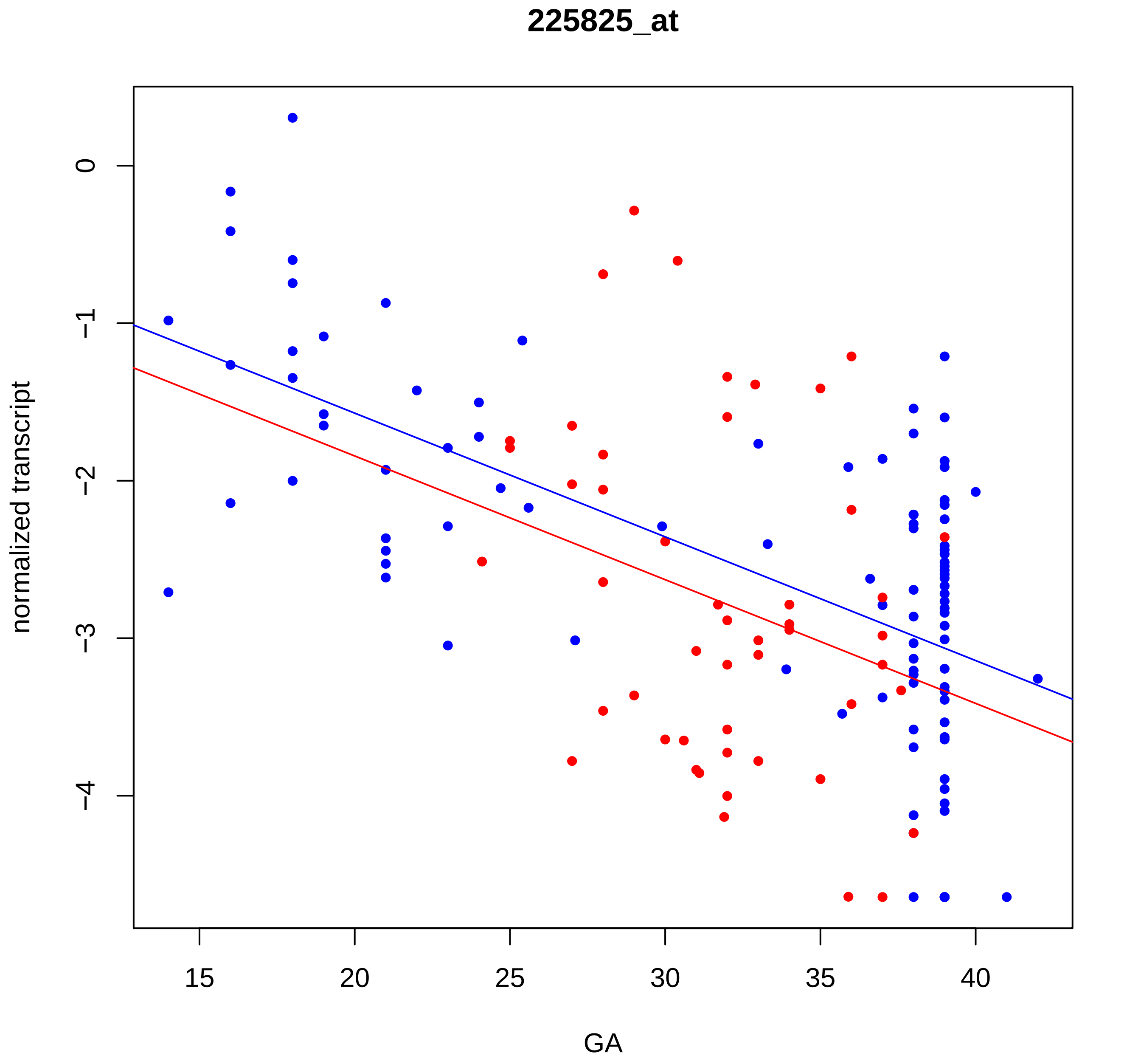

225825_at
normalized transcript
0
−1
−2
−3
−4
15
20
25
30
35
40
GA

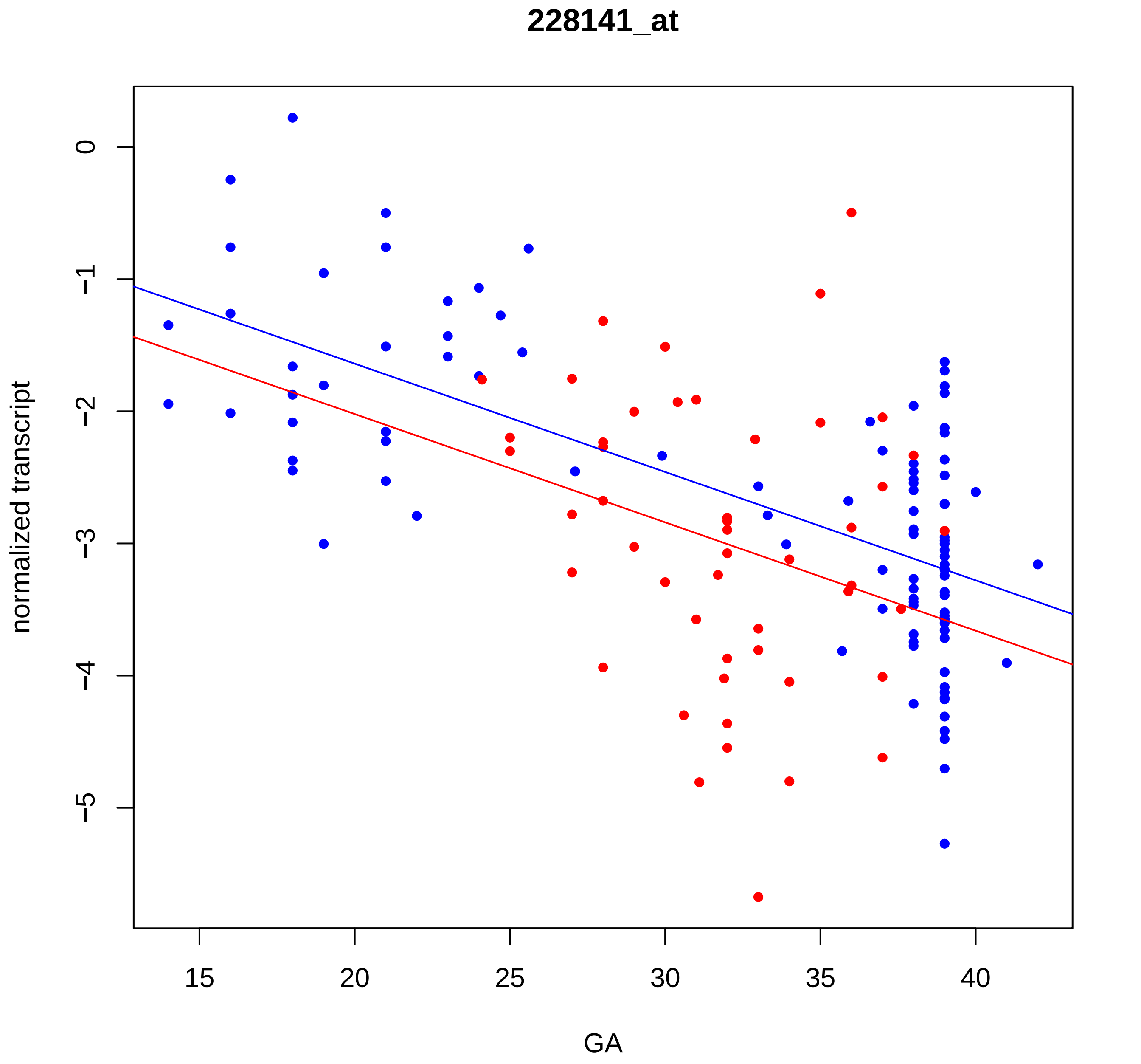

228141_at
0
−1
−2
−3
−4
−5
normalized transcript
15
20
25
30
35
40
GA

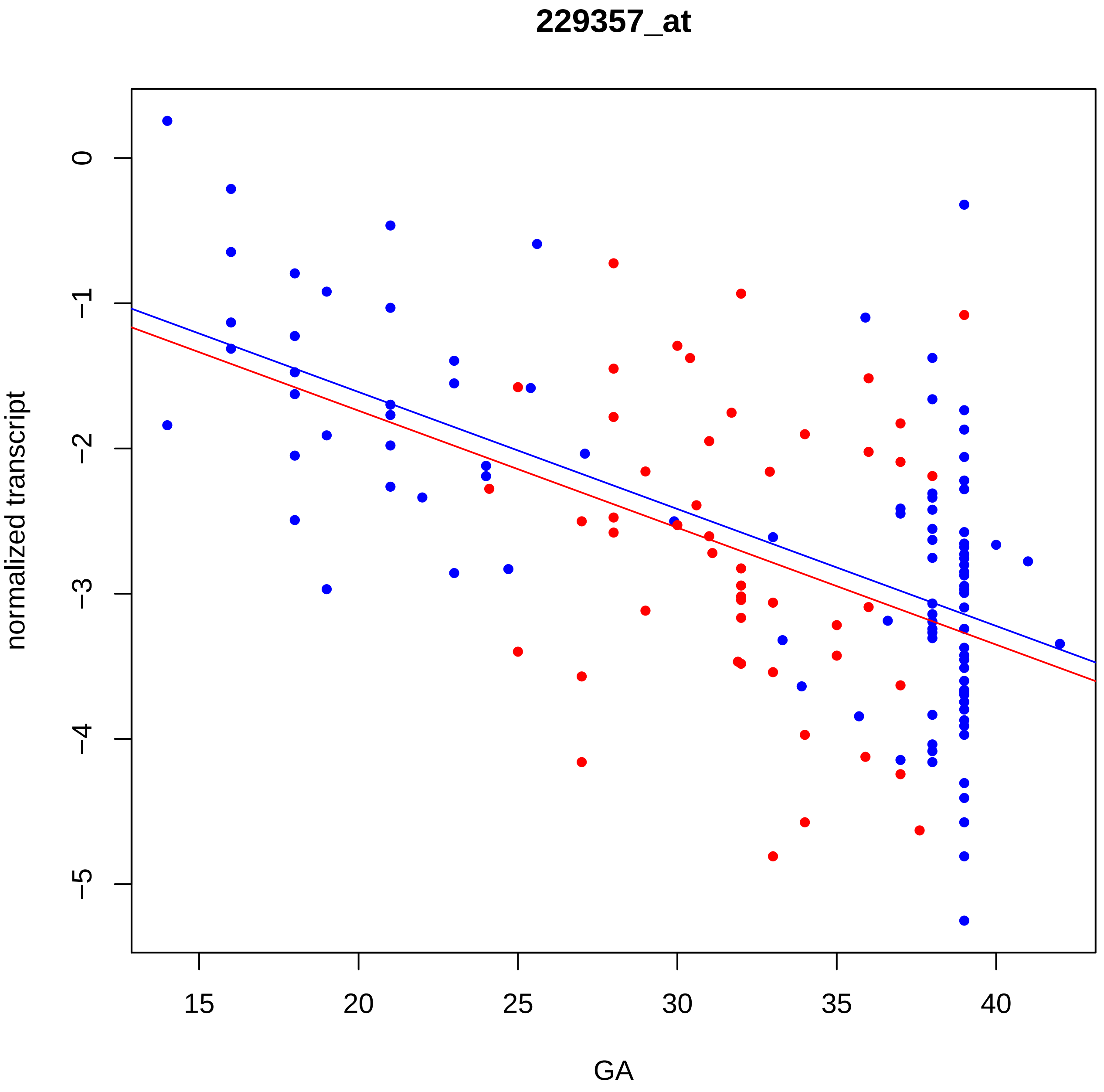

229357_at
normalized transcript
0
−1
−2
−3
−4
−5
15
20
25
30
35
40
GA

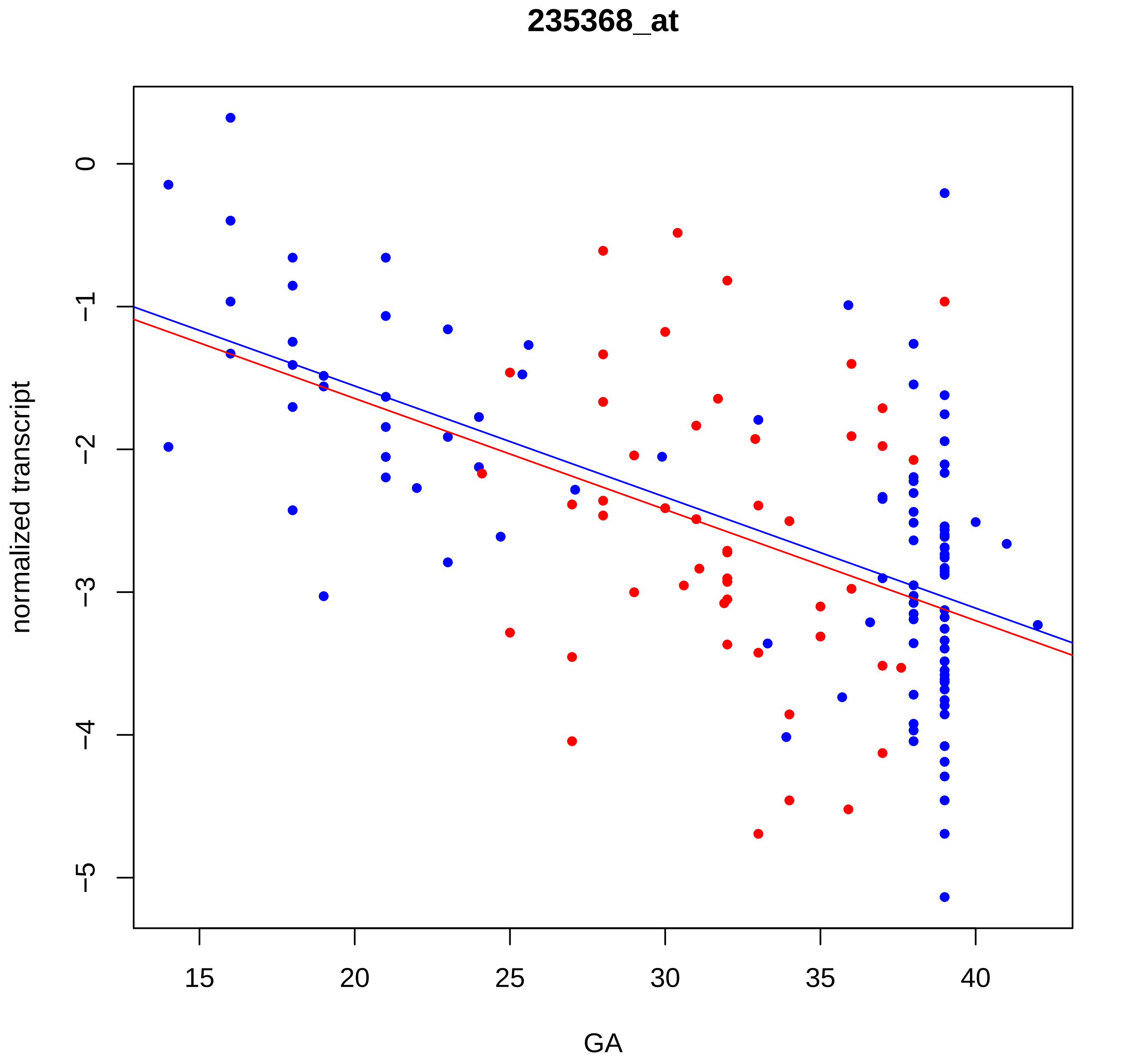
235368_at
normalized transcript
GA
0
−1
−2
−3
−4
−5
15
20
25
30
35
40

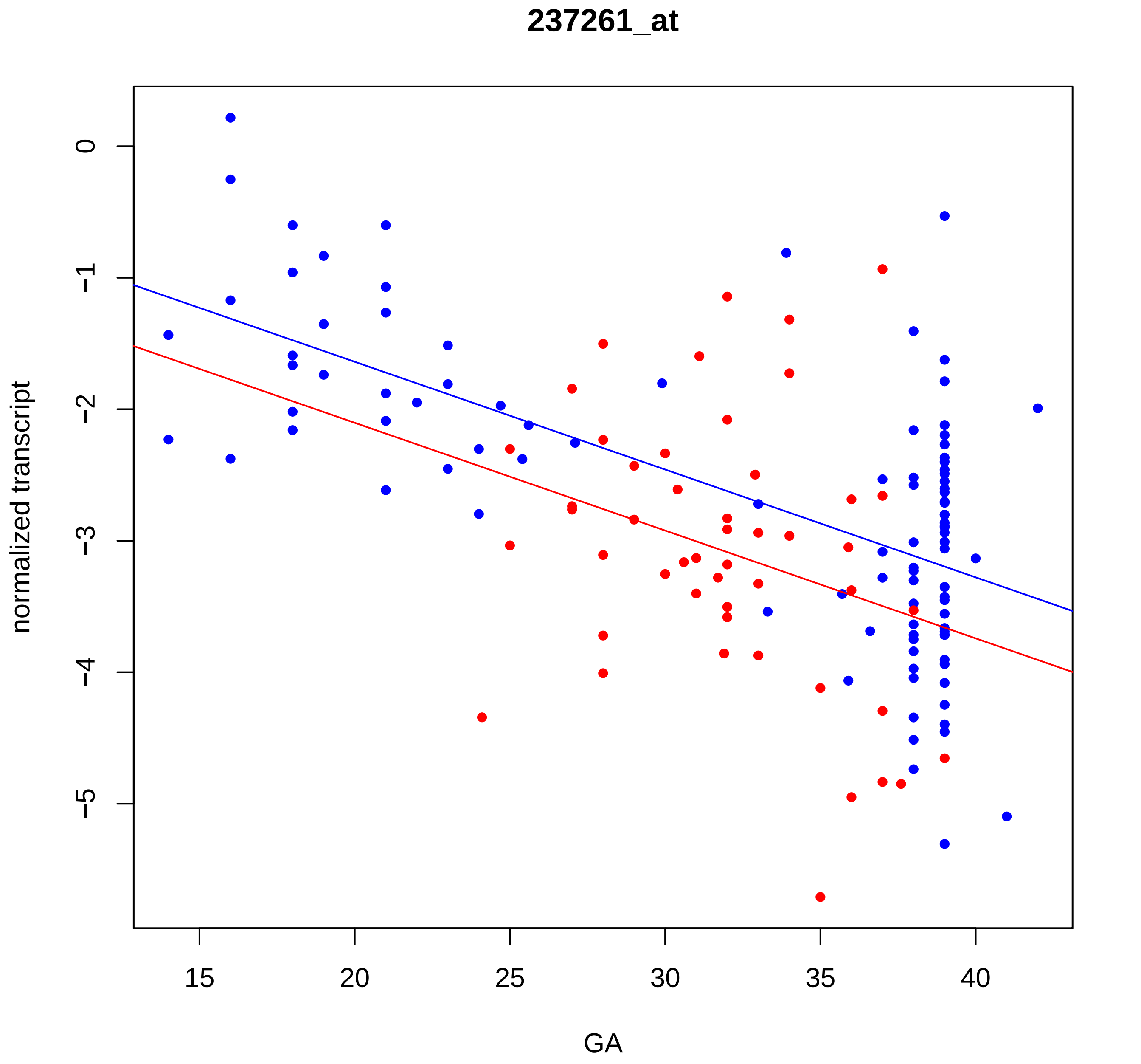

237261_at
normalized transcript
0
−1
−2
−3
−4
−5
15
20
25
30
35
40
GA

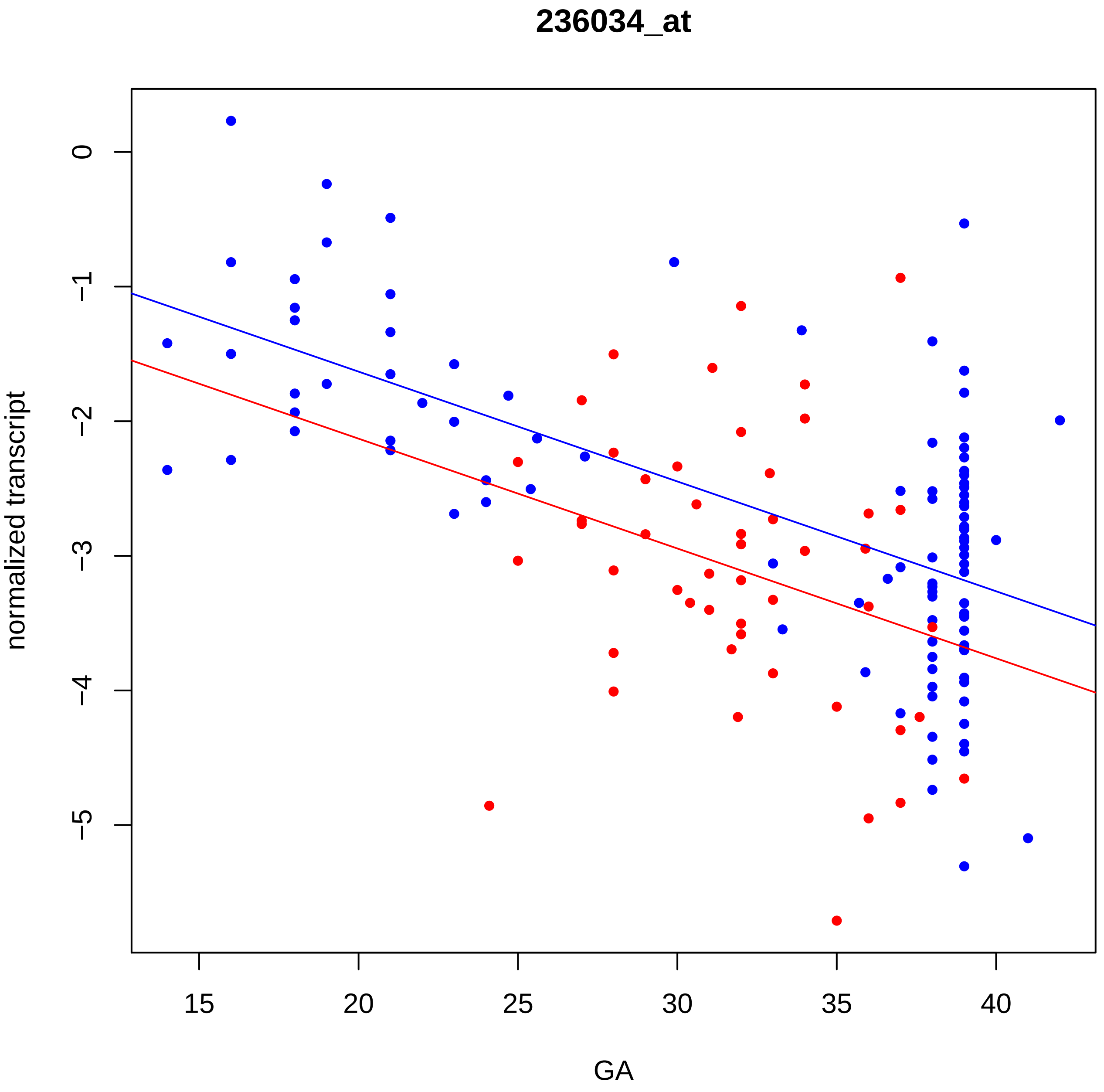

236034_at
0
−1
−2
−3
−4
−5
normalized transcript
15
20
25
30
35
40
GA
